# Supplementary material for: Discovery of N-(6-Methoxypyridin-3-yl)quinoline-2-amine Derivatives for Imaging Aggregated α-Synuclein in Parkinson’s Disease with Positron Emission Tomography
Source: Cells. 2025 Jul 18;14(14):1108. doi: 10.3390/cells14141108 (PMC12293736; doi:10.3390/cells14141108)

Supplemental Information

Discovery of *N*-(6-Methoxypyridin-3-yl)quinoline-2-amine Derivatives for Imaging Aggregated α-Synuclein in Parkinson's Disease with Positron Emission Tomography

Haiyang Zhao[a], Tianyu Huang[a], Dhruva Dhavale[b], Jennifer Y. O'Shea[b], Zsofia Lengyel-Zhand[c], Dinahlee Saturnino Guarino[c], Jiwei Gu[a], Xuyi Yue[a], Ying-Hwey Nai[a], Hao Jiang[a], Marshall G. Lougee[d], Vinayak V. Pagar[d], Hee Jong Kim[e], Benjamin A. Garcia[e], E. James Petersson[d], Chester A. Mathis[f], Paul Kotzbauer[b], Joel S. Perlmutter[b], Robert H. Mach[c], Zhude Tu[a,*]

[a.] Department of Radiology, Washington University School of Medicine, St Louis, MO, 63110, USA
[b.] Department of Neurology, Washington University School of Medicine, St Louis, MO, 63110, USA
[c.] Department of Radiology, University of Pennsylvania, Perelman School of Medicine, Philadelphia, PA, 19104, USA
[d.] Department of Chemistry, University of Pennsylvania, Philadelphia, PA, 19104, USA
[e.] Department of Biochemistry and Biophysics, University of Pennsylvania, Perelman School of Medicine, Philadelphia, PA 19104, USA
[f.] Department of Radiology, University of Pittsburgh School of Medicine, Pittsburgh, PA, 15213, USA
[*]Corresponding author. Tel.: +1-314-362-8487; Fax: +1-314-362-8555; E-mail: zhudetu@wustl.edu

Table of Contents

1. General Information and Materials………………………………………………..………. 3

2. General Buchwald-Hartwig Amination Procedure A…………….……………………………… 4

3. General Buchwald-Hartwig Amination Procedure B…………………………………………… 4

4. Characterization Data for Compounds **3**,**5**,7,**8**,**9** and **10** ……………..………………………….… 4

5. TZ-CLX Synthesis and Characterization……..………………..…..……………………………. 19

6. Alpha-Synuclein Binding Affinity Studies……..……………..……………………………… 21

7. Radiochemistry……..……………..…..…………………………..…………………………… 22

8. PET brain imaging studies in nonhuman primates………………..…………………………… 40

9. Copies of ^{1}H NMR, and ^{13}C NMR Spectra.…………………..………..…………..…..……….41

1. General Information and Materials

General Information: All dry reactions were conducted under a nitrogen atmosphere in oven dried glass apparatus using anhydrous solvents. Yields refer to chromatographically homogeneous materials, unless otherwise stated. Reactions were monitored by thin-layer chromatography (TLC) carried out on precoated glass plates of silica gel (0.25 mm) 60 F254 from EMD Chemicals Inc. Visualization was accomplished with ultraviolet light (UV 254 nm), or by shaking the plate in a sealed jar containing silica gel and iodine. Flash column chromatography was performed using Silica Flash® P60 40-63μm (230-400 mesh) from Silicycle Inc.. Melting points were determined on a MEL-TEMP 3.0 apparatus. ^{1}H NMR and ^{13}C NMR spectra were recorded on Varian 400 MHz (operating at 400 MHz for ^{1}H and 100 MHz for ^{13}C NMR) spectrometer. Rotamers are denoted by an asterisk (*). Chemical shifts are reported in parts per million (ppm) and coupling constants J are given in Hz (Hertz). Chemical shifts are reported relative to TMS (δ = 0.0) as an internal standard. (Abbreviations used in spectra: s = singlet, d = doublet, t = triplet, q = quartet, m = multiplet, br = broad, dd = double of doublets, dt = doublet of triplets, td = triplet of doublets, qd = quartet of doublets). High resolution mass spectra (HRMS) $[ESI]^{+}$ were recorded on a Bruker MaXis 4G Q-TOF mass spectrometer with electrospray ionization source.

Materials: All reagents were used as received from commercial sources unless otherwise stated, or prepared as described in the literature. All solvents used in the reaction were anhydrous and purchased from Sigma-Aldrich.

2. General Buchwald-Hartwig Amination Procedure A

Pd Catalyzed Cross-Coupling of Substituted 2-halo-(iso)quinoline 1/4/6 with 5-Amino-pyridine 2

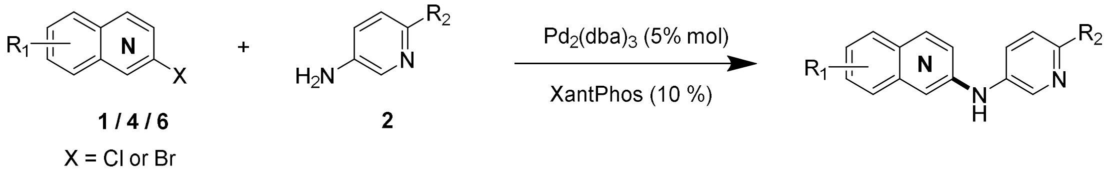

To a 20 mL sealed tube was added by reagent **2** (0.5 mmol, 1.0 equiv), reagent **1/4/6** (1.0 equiv), $Pd_2(dba)_3$ (0.05 mol%), Xantphos (0.1 mol%) and Cs_2CO_3 (2.0 equiv.). The vessel was evacuated and backfilled with nitrogen (three times), then anhydrous 1,4-dioxane (10 mL) was added. The sealed tube was screw capped and heated to 110 °C. After stirring for 12 hours, the reaction mixture was cooled to room temperature and diluted with ethyl acetate. The reaction mixture was filtered through a pad of Celite and washed with ethyl acetate (10 mL×3). The filtrate was concentrated. The residue was subjected to column chromatography on silica gel to afford the product.

3. General Buchwald-Hartwig Amination Procedure B

Pd Catalyzed Cross-Coupling of 6c with 2h

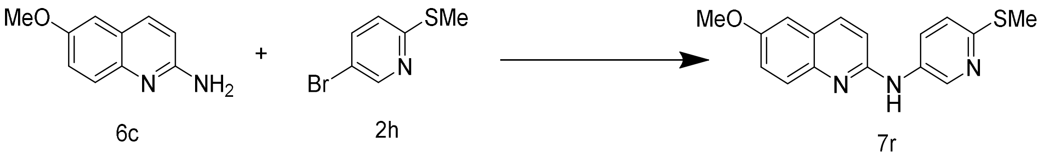

To a 20 mL sealed tube was added by 5-Bromo-2-methoxypyridine **2h** (1.0 equiv), 6-methoxy-quinolin-2-amine **6c** (1.0 equiv), $Pd_2(dba)_3$ (0.025 mol%), Xantphos (0.1 mol%) and Cs_2CO_3 (2.0 equiv.). The vessel was evacuated and backfilled with nitrogen (three times), then anhydrous 1,4-dioxane (10 mL) was added. The sealed tube was screw capped and heated to 110 °C. After stirring for 12 hours, the reaction mixture was cooled to room temperature and diluted with ethyl acetate. The reaction mixture was filtered through a pad of Celite and washed with ethyl acetate (10 mL×3). The filtrate was concentrated. The residue was subjected to column chromatography on silica gel to afford the product.

4. Characterization Data for Compounds 3, 3,5,7,8,9 and 10.

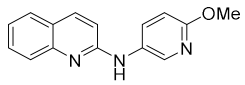

Chemical Formula: $C_{15}H_{13}N_3O$
Exact Mass: 251.1059

***N*-(6-methoxypyridin-3-yl)quinolin-2-amine (3a, TZ80-157).** Following general experimental procedure **A**, a light yellow solid (84 mg, 67% yield).

^{1}H NMR (400 MHz, $CDCl_3$) δ 8.37 (d, *J* = 2.6 Hz, 1 H), 7.96 – 7.84 (m, 2 H), 7.72 (d, *J* = 8.4 Hz, 1 H), 7.61 (d, *J* = 8.0 Hz, 1 H), 7.55 (t, *J* = 7.7 Hz, 1 H), 7.31 – 7.22 (m, 1 H), 6.83 (s, 1 H), 6.78 (dd, *J* = 8.8, 4.9 Hz, 2 H), 3.93 (s, 3 H). ^{13}C NMR (100 MHz, $CDCl_3$) δ 160.6, 154.7, 147.5, 139.9, 137.9, 133.6, 130.7, 129.9, 127.4, 126.6, 124.0, 123.1, 111.2, 110.8, 53.6. HRMS(ESI) calcd. for $C_{15}H_{14}N_3O$ $[M+H]^+$ 252.1131, found: 252.1135.

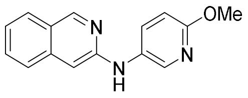

Chemical Formula: $C_{15}H_{13}N_3O$
Exact Mass: 251.1059

***N*-(6-methoxypyridin-3-yl)isoquinolin-3-amine (3b, TZ90-14).** Following general experimental procedure **A**, a light yellow solid (64 mg, 51% yield).

^{1}H NMR (400 MHz, $CDCl_3$) δ 8.91 (s, 1 H), 8.22 (s, 1 H), 7.78 (d, *J* = 8.1 Hz, 1 H), 7.65 (d, *J* = 10.0 Hz, 1 H), 7.49 (s, 2 H), 7.25 (s, 1 H), 6.96 – 6.66 (m, 3 H), 3.96 (s, 3 H). ^{13}C NMR (100 MHz, $CDCl_3$) δ 160.8, 153.4, 151.9, 141.1, 138.6, 134.4, 131.2, 130.6, 127.7, 125.0, 124.3, 123.4, 111.2, 97.8, 53.6. HRMS(ESI) calcd. for $C_{15}H_{14}N_3O$ $[M+H]^+$ 252.1131, found: 252.1131.

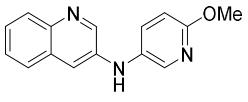

Chemical Formula: $C_{15}H_{13}N_3O$
Exact Mass: 251.1059

***N*-(6-methoxypyridin-3-yl)quinolin-3-amine (3c, TZ90-6).** Following general experimental procedure **A**, a light brown oil (52 mg, 42% yield).

^{1}H NMR (400 MHz, $CDCl_3$) δ 8.61 (s, 1 H), 8.11 (s, 1 H), 7.96 (d, *J* = 8.0 Hz, 1 H), 7.53 (dd, *J* = 15.6, 8.2 Hz, 2 H), 7.44 (p, *J* = 6.8 Hz, 2 H), 7.38 (s, 1 H), 6.78 (d, *J* = 8.8 Hz, 1 H), 6.14 (s, 1 H), 3.94 (s, 3 H).^{13}C NMR (100 MHz, $CDCl_3$) δ 160.8, 143.6, 143.0, 140.3, 138.8, 133.5, 131.5, 128.8, 127.2, 126.2, 126.1, 113.9, 111.4, 53.6. HRMS(ESI) calcd. for $C_{15}H_{14}N_3O$ $[M+H]^+$ 252.1131, found: 252.1130.

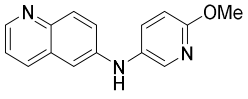

Chemical Formula: $C_{15}H_{13}N_3O$
Exact Mass: 251.1059

***N*-(6-Methoxypyridin-3-yl)quinolin-6-amine (3d, TZ64-29).** Following general experimental procedure **A**, a light brown solid (81.5 mg, 65% yield).

^{1}H NMR (400 MHz, $CDCl_3$) δ 8.65 (s, 1 H), 8.09 (s, 1 H), 7.93 (d, *J* = 9.0 Hz, 1 H), 7.85 (d, *J* = 8.3 Hz, 1 H), 7.52 (d, *J* = 8.8 Hz, 1 H), 7.26 (dt, *J* = 9.1, 3.1 Hz, 2 H), 7.01 (s, 1 H), 6.77 (d, *J* = 8.7 Hz, 1 H), 5.93 (s, 1 H), 3.93 (s, 3 H). ^{13}C NMR (100 MHz, $CDCl_3$) δ 160.8, 147.3, 143.9, 143.4, 140.9, 134.18, 134.15, 131.9, 130.7, 129.6, 121.7, 121.6, 111.3, 106.8, 53.6. HRMS(ESI) calcd. for $C_{15}H_{14}N_3O$ $[M+H]^+$ 252.1131, found: 252.1130.

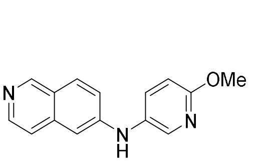

Chemical Formula: $C_{15}H_{13}N_3O$
Exact Mass: 251.1059

***N*-(6-Methoxypyridin-3-yl)isoquinolin-6-amine (3e, TZ64-27).** Following general experimental procedure **A**, a light brown solid (82.8 mg, 66% yield).

^{1}H NMR (400 MHz, $CDCl_3$) δ 8.97 (s, 1 H), 8.30 (d, *J* = 5.8 Hz, 1 H), 8.11 (d, *J* = 2.6 Hz, 1 H), 7.76 (d, *J* = 8.8 Hz, 1 H), 7.54 (dd, *J* = 8.7, 2.6 Hz, 1 H), 7.31 (d, *J* = 5.8 Hz, 1 H), 7.11 (dd, *J* = 8.8, 1.9 Hz, 1 H), 6.93 (s, 1 H), 6.79 (d, *J* = 8.7 Hz, 1 H), 6.33 (s, 1 H), 3.94 (s, 3 H). ^{13}C NMR (100 MHz, $CDCl_3$) δ 161.3, 151.4, 147.1, 143.4, 141.9, 137.8, 135.0, 131.0, 129.4, 123.8, 119.1, 119.0, 111.4, 104.5, 53.6. HRMS(ESI) calcd. for $C_{15}H_{14}N_3O$ $[M+H]^+$ 252.1131, found: 252.1135.

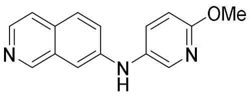

Chemical Formula: $C_{16}H_{14}N_2O$
Exact Mass: 250.1106

N-(6-Methoxypyridin-3-yl)isoquinolin-7-amine (3f, TZ90-15). Following general experimental procedure **A**, a light yellow solid (74 mg, 59% yield).

^{1}H NMR (400 MHz, $CDCl_3$) δ 8.98 (s, 1 H), 8.31 (d, J = 5.6 Hz, 1 H), 8.11 (s, 1 H), 7.68 (d, J = 8.8 Hz, 1 H), 7.50-7.55 (m, 2 H), 7.27 (d, J = 11.0 Hz, 1 H), 7.16 (s, 1 H), 6.80 (d, J = 8.7 Hz, 1 H), 5.93 (s, 1 H), 3.95 (s, 3 H). ^{13}C NMR (100 MHz, $CDCl_3$) δ 161.0, 150.6, 144.2, 141.0, 140.4, 134.3, 131.6, 130.7, 130.1, 128.0, 122.6, 120.2, 111.4, 106.5, 53.6. HRMS(ESI) calcd. for $C_{15}H_{14}N_3O$ $[M+H]^+$ 252.1132, found: 252.1131.

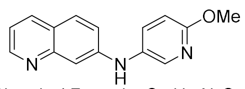

Chemical Formula: $C_{15}H_{13}N_3O$
Exact Mass: 251.1059

N-(6-Methoxypyridin-3-yl)quinolin-7-amine (3g, TZ64-33). Following general experimental procedure **A**, a light yellow solid (65.3 mg, 52% yield).

^{1}H NMR (400 MHz, $CDCl_3$) δ 8.77 – 8.69 (m, 1 H), 8.08 (s, 1 H), 7.98 (d, J = 8.1 Hz, 1 H), 7.64 (d, J = 8.8 Hz, 1 H), 7.58 (d, J = 8.8 Hz, 1 H), 7.32 (s, 1 H), 7.15 (dd, J = 8.1, 4.3 Hz, 1 H), 7.10 (d, J = 8.8 Hz, 1 H), 6.76 (d, J = 8.8 Hz, 1 H), 5.87 (s, 1 H), 3.93 (s, 3 H). ^{13}C NMR (100 MHz, $CDCl_3$) δ 161.1, 150.8, 149.9, 146.5, 141.5, 135.6, 134.6, 131.4, 129.0, 122.9, 118.5, 118.2, 111.3, 108.7, 53.6. HRMS(ESI) calcd. for $C_{15}H_{14}N_3O$ $[M+H]^+$ 252.1131, found: 252.1135.

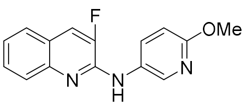

Chemical Formula: $C_{15}H_{12}FN_3O$
Exact Mass: 269.0964

3-Fluoro-N-(6-methoxypyridin-3-yl)quinolin-2-amine (5a, TZ64-14). Following general experimental procedure **A**, a light yellow solid (70 mg, 52% yield).

^{1}H NMR (400 MHz, $CDCl_3$) δ 8.65 (d, J = 2.2 Hz, 1 H), 8.21 (dd, J = 8.9, 2.5 Hz, 1 H), 7.80 (d, J = 8.4 Hz, 1 H), 7.67 – 7.50 (m, 3 H), 7.33 (t, J = 7.5 Hz, 1 H), 6.82 (d, J = 8.9 Hz, 2 H), 3.96 (s, 3 H). ^{13}C NMR (100 MHz, $CDCl_3$) δ 175.1, 160.9, 147.5 (d, J = 260.0 Hz), 145.6 (d, J = 12.0 Hz), 144.6 (d, J = 3.2 Hz), 138.7, 132.5, 130.8, 129.4 (d, J = 2.3 Hz), 127.6, 127.5, 124.7, 118.0 (d, J = 15.0 Hz), 111.2, 54.3. HRMS(ESI) calcd. for $C_{15}H_{13}N_3OF$ $[M+H]^+$ 270.1037, found: 270.1041.

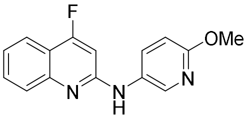

Chemical Formula: $C_{15}H_{12}FN_3O$
Exact Mass: 269.0964

4-Fluoro-N-(6-methoxypyridin-3-yl)quinolin-2-amine (5b, TZ64-66). Following general experimental procedure **A**, a light brown solid (63.2 mg, 47% yield).

^{1}H NMR (400 MHz, $CDCl_3$) δ 8.32 (d, J = 2.6 Hz, 1 H), 7.88 (d, J = 8.1 Hz, 1 H), 7.82 (dd, J = 8.8, 2.6 Hz, 1 H), 7.72 (d, J = 8.5 Hz, 1 H), 7.62 (t, J = 7.7 Hz, 1 H), 7.32 (t, J = 7.5 Hz, 1 H), 6.81 (d, J = 8.8 Hz, 2 H), 6.49 (d, J =

11.5 Hz, 1 H), 3.96 (s, 3 H). ^{13}C NMR (100 MHz, $CDCl_3$) δ 166.3 (d, *J* = 264.6 Hz), 161.2, 156.1 (d, *J* = 11.4 Hz), 149.3 (d, *J* = 6.2 Hz), 141.0, 134.5, 131.1, 129.9, 126.2 (d, *J* = 4.0 Hz), 123.2 (d, *J* = 0.6 Hz), 120.6 (d, *J* = 4.8 Hz), 116.0 (d, *J* = 14.8 Hz), 111.1, 94.6 (d, *J* = 19.6 Hz), 53.6. HRMS(ESI) calcd. for $C_{15}H_{13}N_3OF$ $[M+H]^+$ 270.1037, found: 270.1041.

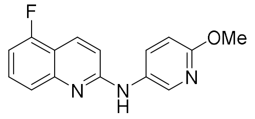

Chemical Formula: $C_{15}H_{12}FN_3O$
Exact Mass: 269.0964

5-Fluoro-*N*-(6-methoxypyridin-3-yl)quinolin-2-amine (5c, TZ64-15). Following general experimental procedure **A**, a light brown solid (76.7 mg, 57% yield).

^1H NMR (400 MHz, $CDCl_3$) δ 8.39 (d, *J* = 2.7 Hz, 1 H), 8.17 (d, *J* = 9.1 Hz, 1 H), 7.91 (dd, *J* = 8.8, 2.7 Hz, 1 H), 7.50 (dt, *J* = 14.7, 8.5 Hz, 2 H), 6.95 (dd, *J* = 12.8, 5.0 Hz, 1 H), 6.82 (dd, *J* = 11.4, 9.0 Hz, 2 H), 6.73 (s, 1 H), 3.96 (s, 3 H). ^{13}C NMR (100 MHz, $CDCl_3$) δ 160.9, 159.6, 157.1, 155.1, 140.2, 133.8, 131.1 (d, *J* = 4.7 Hz), 130.1, 129.6 (d, *J* = 9.6 Hz), 122.3 (d, *J* = 3.7 Hz), 114.1 (d, *J* = 16.2 Hz), 111.3 (d, *J* = 2.6 Hz), 110.9, 107.1 (d, *J* = 19.6 Hz), 53.6. HRMS(ESI) calcd. for $C_{15}H_{13}N_3OF$ $[M+H]^+$ 270.1037, found: 270.1038.

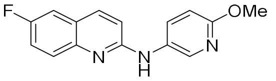

Chemical Formula: $C_{15}H_{12}FN_3O$
Exact Mass: 269.0964

6-Fluoro-*N*-(6-methoxypyridin-3-yl)quinolin-2-amine (5d, TZ64-13). Following general experimental procedure **A**, a light yellow solid (84.7 mg, 63% yield).

^1H NMR (400 MHz, $CDCl_3$) δ 8.38 (d, *J* = 2.7 Hz, 1 H), 7.91 (dd, *J* = 8.8, 2.8 Hz, 1 H), 7.86 (d, *J* = 9.0 Hz, 1 H), 7.73 (dd, *J* = 9.1, 5.1 Hz, 1 H), 7.35 (td, *J* = 8.8, 2.8 Hz, 1 H), 7.32 – 7.27 (m, 1 H), 6.82 (dd, *J* = 15.6, 8.9 Hz, 2 H), 6.65 (s, 1 H), 3.96 (s, 3 H). ^{13}C NMR (100 MHz, $CDCl_3$) δ 160.8, 158.6 (d, *J* = 243.1 Hz), 154.1 (d, *J* = 2.1 Hz), 144.0, 134.0, 137.3 (d, *J* = 4.6 Hz), 133.6, 130.4, 128.3 (d, *J* = 8.6 Hz), 124.1 (d, *J* = 9.6 Hz), 119.4 (d, *J* = 24.9 Hz), 112.1, 110.899 (d, *J* = 10.9 Hz), 110.896, 53.6. HRMS(ESI) calcd. for $C_{15}H_{13}N_3OF$ $[M+H]^+$ 270.1037, found: 270.1040.

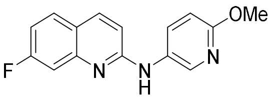

Chemical Formula: $C_{15}H_{12}FN_3O$
Exact Mass: 269.0964

7-Fluoro-*N*-(6-methoxypyridin-3-yl)quinolin-2-amine (5e, TZ64-68). Following general experimental procedure **A**, a light yellow solid (68.6 mg, 51% yield).

^1H NMR (400 MHz, $CDCl_3$) δ 8.36 (d, *J* = 2.6 Hz, 1 H), 7.91 (dd, *J* = 8.8, 2.7 Hz, 1 H), 7.87 (d, *J* = 8.9 Hz, 1 H), 7.59 (dd, *J* = 8.7, 6.3 Hz, 1 H), 7.36 (dd, *J* = 10.7, 2.2 Hz, 1 H), 7.05 (td, *J* = 8.6, 2.4 Hz, 1 H), 6.80 (d, *J* = 8.8 Hz, 1 H), 6.75 (d, *J* = 8.9 Hz, 2 H), 3.95 (s, 3 H). ^{13}C NMR (100 MHz, $CDCl_3$) δ 163.8 (d, *J* = 248.3 Hz), 160.8, 155.4, 148.8 (d, *J* = 13.2 Hz), 140.2, 137.7 (d, *J* = 0.5 Hz), 133.8, 130.3, 129.2 (d, *J* = 10.5 Hz), 120.9 (d, *J* = 1.2 Hz), 112.8 (d, *J* = 24.8 Hz), 110.9, 110.7 (d, *J* = 20.9 Hz), 110.3 (d, *J* = 2.7 Hz), 53.6. HRMS(ESI) calcd. for $C_{15}H_{13}N_3OF$ $[M+H]^+$ 270.1037, found: 270.1038.

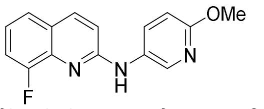

Chemical Formula: $C_{15}H_{12}FN_3O$
Exact Mass: 269.0964

8-Fluoro-*N*-(6-methoxypyridin-3-yl)quinolin-2-amine (5f, TZ64-16). Following general experimental procedure **A**, a light yellow solid (56.5 mg, 42% yield).

^{1}H NMR (400 MHz, $CDCl_3$) δ 8.29 (d, *J* = 2.7 Hz, 1 H), 7.97 (dd, *J* = 8.8, 2.8 Hz, 1 H), 7.92 (d, *J* = 9.0 Hz, 1 H), 7.43 (d, *J* = 8.0 Hz, 1 H), 7.26-7.33 (m, 1 H), 7.23-7.17 (m, 1 H), 6.87 (d, *J* = 9.0 Hz, 1 H), 6.83-6.79 (m, 2 H), 3.95 (s, 3 H). ^{13}C NMR (100 MHz, $CDCl_3$) δ 158.4, 156.92, 156.56, 141.2, 138.4 (d, *J* = 3.2 Hz), 138.1 (d, *J* = 11.5 Hz), 134.7, 130.9, 126.4 (d, *J* = 3.2 Hz), 123.7 (d, *J* = 4.2 Hz), 123.1 (d, *J* = 7.7 Hz), 115.0 (d, *J* = 19.0 Hz), 112.4, 111.8, 54.3. HRMS(ESI) calcd. for $C_{15}H_{13}N_3OF$ $[M+H]^+$ 270.1037, found: 270.1041.

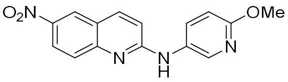

Chemical Formula: $C_{15}H_{12}N_4O_3$
Exact Mass: 296.0909

***N*-(6-Methoxypyridin-3-yl)-6-nitroquinolin-2-amine (7a, TZ90-9).** Following general experimental procedure **A**, a light yellow solid (109.5 mg, 74% yield).

^{1}H NMR (400 MHz, DMSO-d6) δ 9.92 (s, 1 H), 8.78 – 8.75 (m, 2 H), 8.37 – 8.19 (m, 3 H), 7.74 (d, *J* = 9.2 Hz, 1 H), 7.15 (d, *J* = 9.0 Hz, 1 H), 6.87 (d, *J* = 8.9 Hz, 1 H), 3.85 (s, 3 H). ^{13}C NMR (100 MHz, DMSO-d6) δ 159.1, 156.4, 150.8, 141.7, 138.4, 137.7, 131.7, 131.4, 127.1, 124.5, 123.4, 122.3, 115.8, 110.1, 53.2. HRMS(ESI) calcd. for $C_{15}H_{13}N_4O_3$ $[M+H]^+$ 297.0982, found: 297.0979.

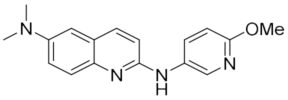

Chemical Formula: $C_{17}H_{18}N_4O$
Exact Mass: 294.1481

***N*²-(6-methoxypyridin-3-yl)-*N*⁶,*N*⁶-dimethylquinoline-2,6-diamine (7b, TZ90-11).** Following general experimental procedure **A**, a light yellow solid (110.3 mg, 75% yield).

^{1}H NMR (400 MHz, $CDCl_3$) δ 8.35 (s, 1 H), 7.88 (d, *J* = 8.8 Hz, 1 H), 7.76 (d, *J* = 8.9 Hz, 1 H), 7.64 (d, *J* = 9.2 Hz, 1 H), 7.26 (d, *J* = 7.2 Hz, 1 H), 6.76 (s, 2 H), 6.74 (s, 1 H), 6.57 (s, 1 H), 3.93 (s, 3 H), 2.99 (s, 6 H). ^{13}C NMR (100 MHz, $CDCl_3$) δ 160.2, 152.3, 147.0, 140.9, 139.2, 136.6, 133.0, 131.4, 127.2, 125.1, 119.8, 111.5, 110.7, 107.0, 53.5, 41.2. HRMS(ESI) calcd. for $C_{17}H_{19}N_4O$ $[M+H]^+$ 295.1553, found: 295.1559.

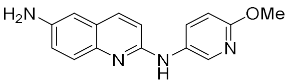

Chemical Formula: $C_{15}H_{14}N_4O$
Exact Mass: 266.1168

***N*²-(6-methoxypyridin-3-yl)quinoline-2,6-diamine (7c, TZ90-12).** To a 20 mL of round-bottom flask were added Pd/C (10 mg), **7a** (50 mg, 0.166 mmol) and 5 mL of MeOH. The reaction solution was stirred under 1 atm of H_2 at room temperature for 12 h. The reaction mixture was filtered through a pad of Celite and washed with ethyl acetate (10 mL×3). The filtrate was concentrated. The residue was subjected to column chromatography on silica gel to afford a light brown solid product (42 mg, 95% yield).

^{1}H NMR (400 MHz, $CDCl_3$) δ 8.35 (s, 1 H), 7.89 (d, *J* = 8.8 Hz, 1 H), 7.71 (d, *J* = 8.9 Hz, 1 H), 7.59 (d, *J* = 8.7 Hz,

1 H), 7.05 (d, $J = 8.7$ Hz, 1 H), 6.84 (s, 1 H), 6.76 (t, $J = 8.1$ Hz, 2 H), 6.49 (s, 1 H), 3.94 (s, 3 H), 3.74 (s, 2 H). ^{13}C NMR (100 MHz, $CDCl_3$) δ 160.3, 152.5, 142.0, 141.9, 139.3, 136.2, 133.1, 131.3, 127.7, 125.1, 121.4, 111.6, 110.7, 109.1, 53.5. HRMS(ESI) calcd. for $C_{15}H_{15}N_4O$ $[M+H]^+$ 267.1241, found: 267.1240.

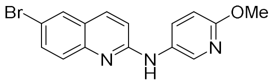

6-Bromo-N-(6-methoxypyridin-3-yl)quinolin-2-amine (7d, TZ64-105). To a 20 mL sealed tube was added by 5-Amino-2-Methoxypyridine (1.0 equiv), 2-chloro-6-Br-quinoline (1.0 equiv), $Pd_2(dba)_3$ (0.025 mol%), Xantphos (0.1 mol%) and Cs_2CO_3 (2.0 equiv.). The vessel was evacuated and backfilled with nitrogen (three times), then anhydrous 1,4-dioxane (10 mL) was added. The sealed tube was screw capped and heated to 100 ^{o}C. After stirring for 12 hours, the reaction mixture was cooled to room temperature and diluted with ethyl acetate. The reaction mixture was filtered through a pad of Celite and washed with ethyl acetate (10 mL×3). The filtrate was concentrated. The residue was subjected to column chromatography on silica gel to afford a yellow product (87 mg).
^{1}H NMR (400 MHz, $CDCl_3$) δ 8.38 (d, $J = 2.7$ Hz, 1 H), 7.91 (dd, $J = 8.8, 2.8$ Hz, 1 H), 7.86 (d, $J = 9.0$ Hz, 1 H), 7.73 (dd, $J = 9.1, 5.1$ Hz, 1 H), 7.35 (td, $J = 8.8, 2.8$ Hz, 1 H), 7.32 – 7.27 (m, 1 H), 6.82 (dd, $J = 15.6, 8.9$ Hz, 2 H), 6.65 (s, 1 H), 3.96 (s, 3 H). ^{13}C NMR (100 MHz, $CDCl_3$) δ 160.8, 158.6 (d, $J = 243.1$ Hz), 154.1 (d, $J = 2.1$ Hz), 144.0, 134.0, 137.3 (d, $J = 4.6$ Hz), 133.6, 130.4, 128.3 (d, $J = 8.6$ Hz), 124.1 (d, $J = 9.6$ Hz), 119.4 (d, $J = 24.9$ Hz), 112.1, 110.899 (d, $J = 10.9$ Hz), 110.896, 53.6. HRMS(ESI) calcd. for $C_{15}H_{13}N_3OBr$ $[M+H]^+$ 330.0164, found: 330.0166.

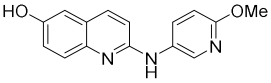

2-((6-Methoxypyridin-3-yl)amino)quinolin-6-ol (7e, TZ80-34). The synthesis procedure containing two steps;
Step 1:
Into a 20 mL sealed tube, the following were added: 5-amino-2-methoxypyridine (0.5 mmol, 1.0 equiv.), 2-chloro-6-(methoxymethoxy)quinoline (1.0 equiv.), tris(dibenzylideneacetone)dipalladium(0) (0.025 mol%), Xantphos (0.1 mol%), and caesium carbonate (2.0 equiv). The vessel was evacuated and backfilled with nitrogen three times before adding anhydrous 1,4-dioxane (10 mL). The tube was then tightly capped and heated to 110°C. After stirring continuously for 12 hours, the reaction mixture was cooled to room temperature and diluted with ethyl acetate, and then filtered with a pad of cellite. The filtrate was diluted with ethyl acetate and washed with brine. The combined organic layers were dried over Na_2SO_4 and concentrated.
Step 2:
The filtrate was concentrated, and then dichloromethane (DCM) (5.0 mL) was used as the solvent. Trifluoroacetic acid (5.0 mL) was added, and the solution was stirred for 10 hours. Subsequently, the solution was concentrated and diluted with ethyl acetate. It was then washed with aqueous sodium bicarbonate. The combined organic layers were further washed with brine, dried over sodium sulfate, filtered, and concentrated. The resultant residue underwent purification through silica gel chromatography, yielding a light green solid product (82.8 mg, 62% yield).
^{1}H NMR (400 MHz, d6-DMSO) δ 9.54 (s, 1 H), 9.31 (s, 1 H), 8.73 (s, 1 H), 8.18 (d, $J = 8.8$ Hz, 1 H), 7.92 (d, $J = 8.9$ Hz, 1 H), 7.54 (d, $J = 8.9$ Hz, 1 H), 7.13 (d, $J = 9.0$ Hz, 1 H), 7.01 (s, 1 H), 6.95 (d, $J = 8.9$ Hz, 1 H), 6.83 (d, $J = 8.9$ Hz, 1 H), 3.83 (s, 3 H). ^{13}C NMR (100 MHz, d6-DMSO) δ 158.4, 153.0, 152.1, 140.2, 136.5, 132.4, 130.9, 126.7,

124.2, 120.9, 113.8, 110.0, 109.6, 53.1. HRMS(ESI) calcd. for $C_{15}H_{14}N_3O_2$ $[M+H]^+$ 268.1081, found: 268.1083.

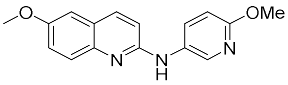

Chemical Formula: $C_{16}H_{15}N_3O_2$
Exact Mass: 281.1164

6-Methoxy-*N*-(6-methoxypyridin-3-yl)quinolin-2-amine (7f, TZ55-107). Following general experimental procedure **A**, a light brown solid (91.3 mg, 65% yield).
^{1}H NMR (400 MHz, $CDCl_3$) δ 8.34 (d, *J* = 2.6 Hz, 1 H), 7.87 (dd, *J* = 8.8, 2.7 Hz, 1 H), 7.83 (dd, *J* = 8.9, 3.2 Hz, 1 H), 7.67 (d, *J* = 9.1 Hz, 1 H), 7.29 – 7.20 (m, 1 H), 6.97 (d, *J* = 1.9 Hz, 1 H), 6.78 (t, *J* = 9.2 Hz, 2 H), 3.93 (s, 3 H), 3.87 (s, 3 H). ^{13}C NMR (100 MHz, $CDCl_3$) δ 161.3, 156.3, 153.9, 143.0, 140.5, 138.0, 134.2, 131.4, 128.3, 125.1, 122.4, 112.0, 111.6, 107.0, 56.2, 54.3. HRMS(ESI) calcd. for $C_{16}H_{16}N_3O_2$ $[M+H]^+$ 282.1237, found: 282.1238.

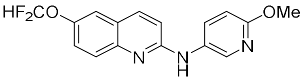

Chemical Formula: $C_{16}H_{13}F_2N_3O_2$
Exact Mass: 317.10

6-(Difluoromethoxy)-*N*-(6-methoxypyridin-3-yl)quinolin-2-amine (7g, TZ80-37). Following general experimental procedure **A**, a light yellow solid (99.8 mg, 63% yield).
^{1}H NMR (400 MHz, $CDCl_3$) δ 12.89 (s, 1 H), 8.17 (d, *J* = 2.6 Hz, 1 H), 8.04 (d, *J* = 9.5 Hz, 1 H), 7.95 (d, *J* = 9.1 Hz, 1 H), 7.53 (dt, *J* = 9.1, 2.7 Hz, 2 H), 7.46 (d, *J* = 2.3 Hz, 1 H), 6.96 (d, *J* = 9.5 Hz, 1 H), 6.84 (d, *J* = 8.8 Hz, 1 H), 6.58 (t, *J* = 72.6 Hz, 1 H), 3.95 (s, 3 H). ^{13}C NMR (100 MHz, $CDCl_3$) δ 164.1, 154.4, 148.4 (t, *J* = 2.9 Hz), 144.7, 143.7, 137.3, 135.8, 127.4, 126.7, 122.6, 121.2, 118.2, 116.1 (t, *J* = 262 Hz), 113.0, 112.0, 54.7. HRMS (ESI) calcd. for $C_{16}H_{14}N_3O_2F_2$ $[M+H]^+$ 318.1045, found: 318.1049.

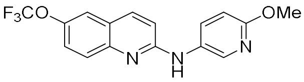

Chemical Formula: $C_{16}H_{12}F_3N_3O_2$
Exact Mass: 335.0882

***N*-(6-Methoxypyridin-3-yl)-6-(trifluoromethoxy)quinolin-2-amine (7h, TZ90-17).** Following general experimental procedure **A**, a light yellow solid (120.6 mg, 72% yield).
^{1}H NMR (400 MHz, $CDCl_3$) δ 8.40 (s, 1 H), 7.92-7.87 (m, 2 H), 7.74 (d, *J* = 9.0 Hz, 1 H), 7.47 (s, 1 H), 7.43 (d, *J* = 9.2 Hz, 1 H), 6.86-6.79 (m, 2 H), 6.70 (s, 1 H), 3.95 (s, 3 H). ^{13}C NMR (100 MHz, $CDCl_3$) δ 160.8, 154.9, 145.9, 144.4 (q, *J* = 1.9 Hz), 140.1, 137.5, 133.7, 130.3, 128.3, 123.8, 120.6 (q, *J* = 257.0 Hz), 118.3, 112.4, 110.9, 53.6. HRMS (ESI) calcd. for $C_{16}H_{13}N_3O_2F_3$ $[M+H]^+$ 336.0954, found: 336.0955.

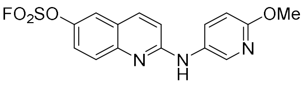

Chemical Formula: $C_{15}H_{12}FN_3O_4S$
Exact Mass: 349.05

2-((6-Methoxypyridin-3-yl)amino)quinolin-6-yl sulfurofluoridate (7i, TZ80-151). A solution of triethylamine (440 µL, 3.2 mmol) and the compound **7e** (0.5 mmol, 133 mg) in acetonitrile (1 mL) was stirred at room temperature for 10 min. A solution of fluorosulfuryl imidazolium salt (207 mg, 0.6 mmol) in acetonitrile (1 mL) was added and the mixture was stirred for 1 h, the reaction mixture was filtered through a pad of Celite and washed with ethyl acetate

(10 mL×3). The filtrate was concentrated. The residue was subjected to column chromatography on silica gel to afford a light-yellow solid product (156.5 mg, 90 % yield).
^{1}H NMR (400 MHz, $CDCl_3$) δ 8.41 (d, J = 2.6 Hz, 1 H), 7.90 (d, J = 9.0 Hz, 2 H), 7.79 (d, J = 9.2 Hz, 1 H), 7.61 (d, J = 2.4 Hz, 1 H), 7.50 (dd, J = 9.2, 2.5 Hz, 1 H), 6.88 (d, J = 9.0 Hz, 1 H), 6.81 (d, J = 8.8 Hz, 1 H), 6.76 (s, 1 H), 3.96 (s, 3 H). ^{13}C NMR (100 MHz, $CDCl_3$) δ 161.7, 156.1, 147.5, 145.7, 140.9, 138.2, 134.4, 130.7, 129.7, 124.4, 123.3, 119.4, 113.7, 111.7, 54.3. HRMS(ESI) calcd. for $C_{15}H_{13}N_3O_4FS$ [M+H]$^+$ 350.0605, found: 350.0608.

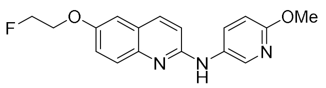

Chemical Formula: $C_{17}H_{16}FN_3O_2$
Exact Mass: 313.1227

6-(2-Fluoroethoxy)-*N*-(6-methoxypyridin-3-yl)quinolin-2-amine (7j, TZ90-3). Following general experimental procedure **A**, a light yellow solid (97.0 mg, 62% yield).
^{1}H NMR (400 MHz, $CDCl_3$) δ 8.36 (s, 1 H), 7.91 (d, J = 8.8 Hz, 1 H), 7.81 (d, J = 8.8 Hz, 1 H), 7.68 (d, J = 9.1 Hz, 1 H), 7.29 (d, J = 9.1 Hz, 1 H), 7.00 (s, 1 H), 6.79 (t, J = 8.1 Hz, 2 H), 6.60 (s, 1 H), 4.80 (d, J = 47.4 Hz, 2 H), 4.28 (d, J = 27.8 Hz, 2 H), 3.94 (s, 3 H). ^{13}C NMR (100 MHz, $CDCl_3$) δ 160.5, 154.2, 153.4, 143.2, 139.6, 136.9, 133.4, 130.9, 128.2, 124.4, 121.6, 111.6, 110.8, 107.5, 81.9 (d, J = 170.7 Hz), 67.4 (d, J = 20.5 Hz), 53.6. HRMS(ESI) calcd. for $C_{17}H_{17}N_3O_2F$ [M+H]$^+$ 314.1299, found: 314.1299.

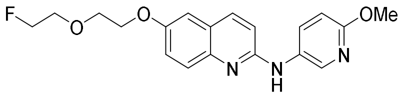

Chemical Formula: $C_{19}H_{20}FN_3O_3$
Exact Mass: 357.15

6-(2-(2-Fluoroethoxy)ethoxy)-*N*-(6-methoxypyridin-3-yl)quinolin-2-amine (7k, TZ80-14). Following general experimental procedure **A**, a light yellow solid (114.2 mg, 64% yield).
^{1}H NMR (400 MHz, $CDCl_3$) δ 8.36 (d, J = 2.7 Hz, 1 H), 7.92 (dd, J = 8.8, 2.8 Hz, 1 H), 7.82 (d, J = 8.9 Hz, 1 H), 7.67 (d, J = 9.1 Hz, 1 H), 7.35 – 7.22 (m, 1 H), 7.01 (d, J = 2.7 Hz, 1 H), 6.79 (dd, J = 8.8, 5.8 Hz, 2 H), 6.45 (s, 1 H), 4.71 – 4.63 (m, 1 H), 4.60 – 4.50 (m, 1 H), 4.27 – 4.17 (m, 2 H), 3.99 – 3.92 (m, 5 H), 3.92 – 3.86 (m, 1 H), 3.85 – 3.78 (m, 1 H). ^{13}C NMR (100 MHz, $CDCl_3$) δ 161.2 (s), 155.3 (s), 154.0 (s), 143.8 (s), 140.3 (s), 137.7 (s), 134.0 (s), 131.7 (s), 128.8 (s), 125.1 (s), 122.5 (s), 112.2 (s), 111.5 (s), 108.1 (s), 83.9 (d, J = 169.2 Hz), 71.3 (d, J = 19.6 Hz), 70.7 (s), 68.5 (s), 54.3 (s). HRMS(ESI) calcd. for $C_{19}H_{21}N_3O_3F$ [M+H]$^+$ 358.1561, found: 358.1562.

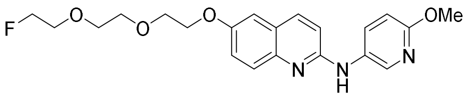

Chemical Formula: $C_{21}H_{24}FN_3O_4$
Exact Mass: 401.18

6-(2-(2-(2-Fluoroethoxy)ethoxy)ethoxy)-*N*-(6-methoxypyridin-3-yl)quinolin-2-amine (7l, TZ80-15). Following general experimental procedure **A**, a light brown solid (114.3 mg, 57% yield).
^{1}H NMR (400 MHz, $CDCl_3$) δ 8.38 (d, J = 2.7 Hz, 1 H), 7.92 (dd, J = 8.8, 2.7 Hz, 1 H), 7.76 (d, J = 8.8 Hz, 1 H), 7.65 (d, J = 9.1 Hz, 1 H), 7.32 – 7.19 (m, 1 H), 6.95 (s, 1 H), 6.78 (t, J = 9.7 Hz, 3 H), 4.68 – 4.56 (m, 1 H), 4.53 – 4.45 (m, 1 H), 4.18 (t, J = 4.3 Hz, 2 H), 3.93 (s, 3 H), 3.92 – 3.87 (m, 2 H), 3.82 – 3.67 (m, 6 H). ^{13}C NMR (100 MHz, $CDCl_3$) δ 161.0 (s), 155.3 (s), 153.9 (s), 143.5 (s), 140.0 (s), 137.6 (s), 133.8 (s), 131.8 (s), 128.5 (s), 125.0 (s), 122.5 (s), 112.3 (s), 111.4 (s), 108.0 (s), 83.8 (d, J = 168.9 Hz), 71.55, 71.54, 71.1 (d, J = 19.6 Hz), 70.5 (s), 68.4 (s), 54.2 (s). HRMS(ESI) calcd. for $C_{21}H_{25}N_3O_4F$ [M+H]$^+$ 402.1824, found: 402.1825.

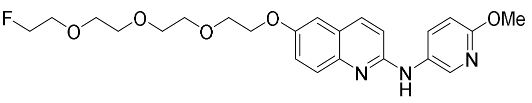

Chemical Formula: $C_{23}H_{28}FN_3O_5$
Exact Mass: 445.20

6-(2-(2-(2-(2-Fluoroethoxy)ethoxy)ethoxy)ethoxy)-*N*-(6-methoxypyridin-3-yl)quinolin-2-amine (7m, TZ80-16). Following general experimental procedure **A**, a light brown solid (113.5 mg, 51% yield).
^{1}H NMR (400 MHz, $CDCl_3$) δ 8.41 (d, *J* = 2.4 Hz, 1 H), 7.97 (dd, *J* = 8.8, 2.8 Hz, 1 H), 7.70 (d, *J* = 8.8 Hz, 1 H), 7.64 (d, *J* = 9.1 Hz, 1 H), 7.23 (dd, *J* = 9.1, 2.8 Hz, 1 H), 6.87 (d, *J* = 2.4 Hz, 2 H), 6.77 (t, *J* = 8.3 Hz, 2 H), 4.62 – 4.50 (m, 1 H), 4.50 – 4.36 (m, 1 H), 4.18 – 4.01 (m, 2 H), 3.93 (s, 3 H), 3.90 – 3.82 (m, 2 H), 3.79 – 3.59 (m, 10 H). ^{13}C NMR (100 MHz, $CDCl_3$) δ 160.1 (s), 154.5 (s), 153.2 (s), 142.9 (s), 139.0 (s), 136.8 (s), 132.8 (s), 131.3 (s), 127.9 (s), 124.4 (s), 121.7 (s), 111.9 (s), 110.6 (s), 107.2 (s), 83.1 (d, *J* = 168.9 Hz), 70.8 (s), 70.74 (s), 70.69 (s), 70.6 (s), 70.3 (d, *J* = 19.6 Hz), 69.7 (s), 67.6 (s), 53.5 (s). HRMS(ESI) calcd. for $C_{23}H_{29}N_3O_5F$ $[M+H]^+$ 446.2086, found: 446.2074.

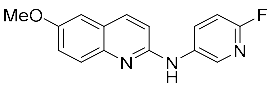

Chemical Formula: $C_{15}H_{12}FN_3O$
Exact Mass: 269.0964

***N*-(6-Fluoropyridin-3-yl)-6-methoxyquinolin-2-amine (7n, TZ77-107).** Following general experimental procedure **A**, a light brown solid (68.6 mg, 51% yield).
^{1}H NMR (400 MHz, d6-DMSO) δ 9.52 (s, 1 H), 8.75 (s, 1 H), 8.51 (td, *J* = 8.9, 2.8 Hz, 1 H), 8.01 (d, *J* = 8.9 Hz, 1 H), 7.61 (d, *J* = 8.9 Hz, 1 H), 7.21 (dt, *J* = 8.5, 2.6 Hz, 2 H), 7.11 (dd, *J* = 8.9, 3.3 Hz, 1 H), 7.00 (d, *J* = 8.9 Hz, 1 H), 3.81 (s, 3 H). ^{13}C NMR (100 MHz, d6-DMSO) δ 157.5 (d, *J* = 229.3 Hz), 155.5, 152.7, 142.3, 137.2 (d, *J* = 4.1 Hz), 137.0, 136.2 (d, *J* = 15.2 Hz), 131.3 (d, *J* = 7.1 Hz), 128.2, 124.7, 121.2, 114.3, 109.3 (d, *J* = 39.2 Hz), 107.2, 55.8. HRMS(ESI) calcd. for $C_{15}H_{13}N_3OF$ $[M+H]^+$ 270.1037, found: 270.1041.

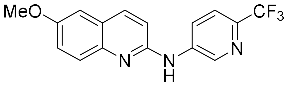

Chemical Formula: $C_{16}H_{12}F_3N_3O$
Exact Mass: 319.09

6-Methoxy-*N*-(6-(trifluoromethyl)pyridin-3-yl)quinolin-2-amine (7o, TZ80-3). Following general experimental procedure **A**, a light yellow solid (97.3 mg, 61% yield).
^{1}H NMR (400 MHz, $CDCl_3$) δ 8.87 (d, *J* = 2.4 Hz, 1 H), 8.71 (dd, *J* = 8.6, 2.3 Hz, 1 H), 7.95 (d, *J* = 8.8 Hz, 1 H), 7.79 (d, *J* = 9.1 Hz, 1 H), 7.68 (d, *J* = 8.7 Hz, 1 H), 7.33 (dd, *J* = 9.1, 2.8 Hz, 1 H), 7.04 (d, *J* = 2.8 Hz, 1 H), 6.90 (d, *J* = 8.8 Hz, 1 H), 6.79 (s, 1 H), 3.92 (s, 3 H). ^{13}C NMR (100 MHz, $CDCl_3$) δ 174.5 (s), 156.4 (s), 150.8 (s), 142.42 (s), 139.8 (s), 137.2 (s), 128.7 (s), 125.1 (s), 124.6 (s), 122.0 (s), 121.0 (q, *J* = 2.7 Hz), 112.9 (s), 106.1 (s), 55.6 (s). HRMS(ESI) calcd. for $C_{16}H_{13}N_3OF_3$ $[M+H]^+$ 320.1005, found: 320.1007.

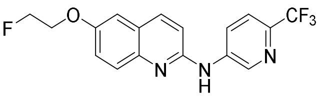

Chemical Formula: $C_{17}H_{13}F_4N_3O$
Exact Mass: 351.10

6-(2-Fluoroethoxy)-*N*-(6-(trifluoromethyl)pyridin-3-yl)quinolin-2-amine (7p, TZ80-4). Following general experimental procedure **A**, a light white solid (103.5 mg, 59% yield).

^{1}H NMR (400 MHz, CD_3OD) δ 9.06 (d, *J* = 2.3 Hz, 1 H), 8.79 (dd, *J* = 8.7, 2.3 Hz, 1 H), 7.99 (d, *J* = 8.9 Hz, 1 H), 7.72 (dd, *J* = 8.9, 5.5 Hz, 2 H), 7.30 (dd, *J* = 9.1, 2.7 Hz, 1 H), 7.17 (d, *J* = 2.7 Hz, 1 H), 7.01 (d, *J* = 8.8 Hz, 1 H), 4.84 – 4.77 (m, 1 H), 4.74 – 4.63 (m, 1 H), 4.35 – 4.29 (m, 1 H), 4.27 – 4.20 (m, 1 H). ^{13}C NMR (100 MHz, CD_3OD) δ 155.1, 152.0, 142.3, 141.5, 139.1, 138.2 (d, *J* = 34.8 Hz), 136.7, 128.5 (d, *J* = 12.3 Hz), 128.0, 125.0, 124.2, 121.2, 120.7 (q, *J* = 2.8 Hz), 113.5, 107.0, 81.71 (d, *J* = 169.0 Hz), 67.5 (d, *J* = 20.0 Hz). HRMS(ESI) calcd. for $C_{17}H_{14}N_3OF_4$ $[M+H]^+$ 352.1068, found: 352.1070.

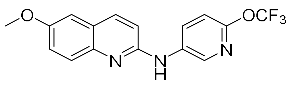

Chemical Formula: $C_{16}H_{12}F_3N_3O_2$
Exact Mass: 335.0882

6-methoxy-N-(6-(trifluoromethoxy)pyridin-3-yl)quinolin-2-amine (7q, TZ90-5). Following general experimental procedure **A**, a light white solid (85.4 mg, 51% yield).

^{1}H NMR (400 MHz, $CDCl_3$) δ 8.57 (d, *J* = 2.5 Hz, 1 H), 8.48 (dd, *J* = 8.8 Hz, 2.8 Hz, 1 H), 7.90 (d, *J* = 8.8 Hz, 1 H), 7.74 (d, *J* = 9.1 Hz, 1 H), 7.29 (dd, *J* = 9.1 Hz, 2.6 Hz, 1 H), 7.07 (d, *J* = 8.8 Hz, 1 H), 7.02 (d, *J* = 2.3 Hz, 1 H), 6.85 (d, *J* = 8.8 Hz, 1 H), 6.65 (s, 1 H), 3.90 (s, 3 H). ^{13}C NMR (100 MHz, $CDCl_3$) δ 156.0, 151.4, 142.5, 137.8, 137.1, 136.4, 130.3, 128.4, 124.9, 121.7, 118.2 (q, *J* = 152.5 Hz), 113.6 (q, *J* = 2.9 Hz), 112.5, 106.1, 55.5. HRMS(ESI) calcd. for $C_{16}H_{13}N_3O_2F_3$ $[M+H]^+$ 336.0954, found: 336.0954.

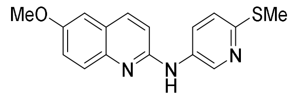

Chemical Formula: $C_{16}H_{15}N_3OS$
Exact Mass: 297.09

6-Methoxy-*N*-(6-(methylthio)pyridin-3-yl)quinolin-2-amine (7r, TZ80-98). Following general experimental procedure **B**, a light yellow solid (69.8 mg, 47% yield).

^{1}H NMR (400 MHz, $CDCl_3$) δ 8.68 (d, *J* = 2.6 Hz, 1 H), 8.06 (dd, *J* = 8.7, 2.8 Hz, 1 H), 7.84 (d, *J* = 8.8 Hz, 1 H), 7.70 (d, *J* = 9.1 Hz, 1 H), 7.30 – 7.22 (m, 1 H), 7.18 (d, *J* = 8.6 Hz, 1 H), 6.98 (d, *J* = 2.7 Hz, 1 H), 6.83 (d, *J* = 8.8 Hz, 1 H), 6.56 (s, 1 H), 3.88 (s, 3 H), 2.57 (s, 3 H). ^{13}C NMR (100 MHz, $CDCl_3$) δ 155.7, 152.3, 152.1, 142.8, 141.1, 136.9, 134.0, 129.5, 128.3, 127.7, 124.7, 121.6, 112.2, 106.2, 55.5, 13.9. HRMS(ESI) calcd. for $C_{16}H_{16}N_3OS$ $[M+H]^+$ 298.1009, found: 298.1012.

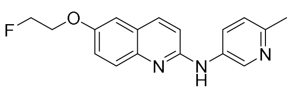

Chemical Formula: $C_{17}H_{16}FN_3O$
Exact Mass: 297.13

6-(2-Fluoroethoxy)-*N*-(6-methylpyridin-3-yl)quinolin-2-amine (7s, TZ80-55). Following general experimental procedure **A**, a light yellow solid (78.7 mg, 53% yield).

^{1}H NMR (400 MHz, $CDCl_3$) δ 8.64 (d, *J* = 2.4 Hz, 1 H), 8.14 (dd, *J* = 8.4, 2.6 Hz, 1 H), 7.83 (d, *J* = 8.9 Hz, 1 H), 7.71 (d, *J* = 9.1 Hz, 1 H), 7.30 (dd, *J* = 9.1, 2.7 Hz, 1 H), 7.14 (d, *J* = 8.4 Hz, 1 H), 6.99 (d, *J* = 2.7 Hz, 1 H), 6.86 (d, *J* = 8.9 Hz, 1 H), 5.05 – 4.59 (m, 2 H), 4.50 – 4.12 (m, 2 H). 2.52 (s, 3 H). ^{13}C NMR (100 MHz, $CDCl_3$) δ 154.5, 152.5, 152.0, 142.9, 140.7, 137.0, 134.7, 128.3, 127.6, 124.5, 123.2, 121.7, 112.3, 107.4, 81.9 (d, *J* = 170.8 Hz), 67.4 (d, *J* = 20.5 Hz), 23.6. HRMS(ESI) calcd. for $C_{17}H_{17}N_3OF$ $[M+H]^+$ 298.1350, found: 298.1351.

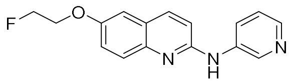

Chemical Formula: $C_{16}H_{14}FN_3O$
Exact Mass: 283.11

6-(2-Fluoroethoxy)-*N*-(pyridin-3-yl)quinolin-2-amine (7t, TZ80-53). Following general experimental procedure **A**, a light yellow solid (59.4 mg, 42% yield).
^{1}H NMR (400 MHz, $CDCl_3$) δ 8.77 (d, *J* = 2.5 Hz, 1 H), 8.37 (d, *J* = 8.3 Hz, 1 H), 8.28 (d, *J* = 4.7 Hz, 1 H), 7.89 (d, *J* = 8.8 Hz, 1 H), 7.77 (d, *J* = 9.1 Hz, 1 H), 7.37 – 7.26 (m, 2 H), 7.03 (d, *J* = 2.6 Hz, 1 H), 6.90 (d, *J* = 8.8 Hz, 1 H), 6.68 (s, 1 H), 4.92 – 4.84 (m, 1 H), 4.80 – 4.72 (m, 1 H), 4.39 – 4.32 (m, 1 H), 4.31 – 4.24 (m, 1 H). ^{13}C NMR (100 MHz, $CDCl_3$) δ 154.7, 152.0, 143.2, 143.0, 141.0, 137.3, 137.0, 128.6, 125.8, 124.6, 123.6, 121.8, 112.6, 107.4, 81.9 (d, *J* = 170.8 Hz), 67.4 (d, *J* = 20.5 Hz). HRMS(ESI) calcd. for $C_{16}H_{15}N_3OF$ $[M+H]^+$ 284.1194, found: 284.1189.

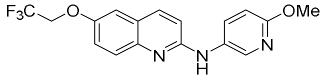

Chemical Formula: $C_{17}H_{14}F_3N_3O_2$
Exact Mass: 349.10

***N*-(6-Methoxypyridin-3-yl)-6-(2,2,2-trifluoroethoxy)quinolin-2-amine (7u, TZ80-58).** Following general experimental procedure **A**, a light yellow solid (130.9 mg, 75% yield).
^{1}H NMR (400 MHz, $CDCl_3$) δ 8.36 (d, *J* = 2.7 Hz, 1 H), 7.90 (dd, *J* = 8.8, 2.7 Hz, 1 H), 7.81 (d, *J* = 8.9 Hz, 1 H), 7.70 (d, *J* = 9.1 Hz, 1 H), 7.29 (dd, *J* = 9.1, 2.8 Hz, 1 H), 7.01 (d, *J* = 2.6 Hz, 1 H), 6.79 (dd, *J* = 11.9, 8.9 Hz, 2 H), 6.54 (s, 1 H), 4.41 (q, *J* = 8.1 Hz, 2 H), 3.93 (s, 3 H). ^{13}C NMR (100 MHz, $CDCl_3$) δ 161.3, 154.5, 153.9, 144.5, 140.5, 137.7, 134.2, 131.4, 129.2, 125.4 (q, *J* = 277 Hz), 124.9, 121.9, 112.6, 111.6, 109.2, 66.9 (q, *J* = 35.6 Hz), 54.3. HRMS(ESI) calcd. for $C_{17}H_{15}N_3O_2F_3$ $[M+H]^+$ 350.1111, found: 350.1110.

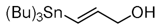

Chemical Formula: $C_{15}H_{32}OSn$
Exact Mass: 348.1475

(*E*)-3-(tributylstannyl)prop-2-en-1-ol (7v, TZ61-83). To a solution of propargyl alcohol (0.84 g, 15 mmol) in toluene (30 mL) was added Bu_3SnH (4.36 g, 15 mmol), azobisisobutyronitrile (0.25 g, 1.5 mmol) under nitrogen, respectively. The mixture was stirred at 120 ºC for 10 h. The reaction mixture was concentrated and the residue was subjected to silica gel chromatography (hexane to hexane/EtOAc 6/1) to afford product **7v** (**TZ61-83**) as a light yellow oil (2.16 g, 42% yield).
^{1}H NMR (400 MHz, $CDCl_3$) δ ^{1}H NMR (400 MHz, Chloroform-d) δ 6.35 – 6.10 (m, 2 H), 4.15 (m, 2 H), 1.90-1.60 (brs, 1 H), 1.53-1.45 (m, 6 H), 1.25-1.34 (m, 6 H), 0.95 – 0.79 (m, 15 H). ^{13}C NMR (100 MHz, $CDCl_3$) δ 147.0, 128.2, 66.3, 29.0, 27.3, 13.6, 9.4.

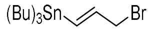

Chemical Formula: $C_{15}H_{31}BrSn$
Exact Mass: 410.0631

(*E*)-(3-bromoprop-1-en-1-yl)tributylstannane (7w, TZ61-79). To a solution of **7v** (**TZ61-83**) (140 mg, 0.40 mmol) in CH_2Cl_2 (2 mL) was added CBr_4 (159 mg, 0.48 mmol), then Ph_3P in CH_2Cl_2 (126 mg, 0.48 mmol) was added dropwise at 0 ºC. The mixture was stirred at 0 ºC for 1 h, then continued to stir at room temperature for 1 h. The reaction mixture was concentrated in vacuo and the residue was subjected to silica gel chromatography (hexane) to afford product **7w** (**TZ61-79**) as a light yellow oil (141 mg, 85% yield).
^{1}H NMR (400 MHz, $CDCl_3$) δ 6.29 - 6.12 (m, 2 H), 3.95 (dd, *J* = 6.7 Hz, 1.0 Hz, 2 H), 1.60-1.41 (m, 6 H), 1.39 –

1.19 (m, 6 H), 0.98 – 0.80 (m, 15H). ^{13}C NMR (100 MHz, $CDCl_3$) δ 143.0, 135.1, 35.9, 29.0, 27.2, 13.7, 9.5.

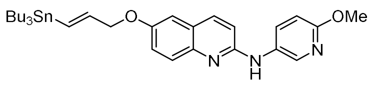

Chemical Formula: $C_{30}H_{43}N_3O_2Sn$
Exact Mass: 597.2377

(*E*)-N-(6-methoxypyridin-3-yl)-6-((3-(tributylstannyl)allyl)oxy)quinolin-2-amine (7x, TZ61-80). To a solution of **7e (TZ80-34)** (20 mg, 0.075 mmol) in anhydrous DMF (2 mL) was added K_2CO_3 (32 mg, 0.098 mmol), (*E*)-(3-bromoprop-1-en-1-yl)tributylstannane **7w** (37 mg, 0.090 mmol) successively. The mixture was stirred at 80 °C in a sealed tube overnight. After cooling down to room temperature, the reaction mixture was concentrated in vacuo and the residue was subjected to silica gel chromatography (hexane/EtOAc 6/1) to afford product **7x (TZ61-80)** as a yellow solid (18 mg, 40% yield).

^{1}H NMR (400 MHz, $CDCl_3$) δ 8.35 (d, *J* = 2.8 Hz, 1 H), 7.89 (dd, *J* = 8.8, 2.8 Hz, 1 H), 7.78 (d, *J* = 8.9 Hz, 1 H), 7.66 (d, *J* = 9.1 Hz, 1 H), 7.43 – 7.21 (m, 1 H), 6.98 (d, *J* = 2.8 Hz, 1 H), 6.79 – 6.75 (m, 3 H), 6.40 (dt, J = 19.2 Hz, 1.5 Hz, 1 H), 6.22 (dt, J = 19.2 Hz, 4.8 Hz, 1H), 4.63 (dd, *J* = 4.8 Hz, 1.6 Hz, 2 H), 3.93 (s, 3 H), 1.56 – 1.43 (m, 6 H), 1.38 – 1.25 (m, 6 H), 1.0 – 0.83 (m, 15 H). ^{13}C NMR (100 MHz, $CDCl_3$) δ 160.4, 154.6, 153.3, 142.8, 142.6, 139.5, 136.9, 133.3, 132.2, 131.1, 127.8, 124.4, 121.9, 111.4, 110.7, 107.8, 71.7, 53.5, 29.0, 27.2, 13.7, 9.4. HRMS(ESI) calcd. for $C_{30}H_{44}N_3O_2Sn$ $[M+H]^+$ 598.2377, found: 598.2450.

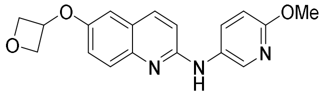

Chemical Formula: $C_{18}H_{17}N_3O_3$
Exact Mass: 323.13

***N*-(6-Methoxypyridin-3-yl)-6-(oxetan-3-yloxy)quinolin-2-amine (8a, TZ80-54).** Following general experimental procedure **A**, a light yellow solid (98.5 mg, 61% yield).

^{1}H NMR (400 MHz, $CDCl_3$) δ 8.37 (d, *J* = 2.7 Hz, 1 H), 7.90 (dd, *J* = 8.8, 2.7 Hz, 1 H), 7.79 (d, *J* = 8.9 Hz, 1 H), 7.70 (d, *J* = 9.1 Hz, 1 H), 7.20 (dd, *J* = 9.1, 2.7 Hz, 1 H), 6.86 – 6.74 (m, 2 H), 6.63 (d, *J* = 2.7 Hz, 1 H), 6.51 (s, 1 H), 5.29 (p, *J* = 5.6 Hz, 1 H), 5.03 (t, *J* = 6.7 Hz, 2 H), 4.88 – 4.75 (m, 2 H), 3.95 (s, 3 H). ^{13}C NMR (100 MHz, $CDCl_3$) δ 174.4, 160.6, 153.4, 152.4, 139.7, 136.9, 133.4, 130.8, 128.5, 124.3, 121.4, 111.7, 110.8, 107.3, 78.0, 70.4, 53.6. HRMS(ESI) calcd. for $C_{18}H_{18}N_3O_3$ $[M+H]^+$ 324.1343, found: 324.1344.

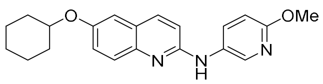

Chemical Formula: $C_{21}H_{23}N_3O_2$
Exact Mass: 349.18

6-Cyclohexyloxy-*N*-(6-methoxypyridin-3-yl)quinolin-2-amine (8b, TZ80-52). Following general experimental procedure **A**, a light brown solid (97.7 mg, 56% yield).

^{1}H NMR (400 MHz, $CDCl_3$) δ 8.34 (d, *J* = 2.4 Hz, 1 H), 7.89 (dd, *J* = 8.8, 2.7 Hz, 1 H), 7.79 (d, *J* = 8.9 Hz, 1 H), 7.65 (d, *J* = 9.1 Hz, 1 H), 7.23 (d, *J* = 2.6 Hz, 2 H), 7.00 (d, *J* = 2.4 Hz, 1 H), 6.77 (dd, *J* = 8.8, 2.5 Hz, 2 H), 6.43 (s, 1 H), 4.36 – 4.23 (m, 1 H), 3.93 (s, 3 H), 2.11 – 1.94 (m, 2 H), 1.87 – 1.73 (m, 2 H), 1.65 – 1.47 (m, 4 H), 1.45 – 1.25 (m, 4 H). ^{13}C NMR (100 MHz, $CDCl_3$) δ 160.4, 153.5, 153.1, 142.8, 139.5, 136.9, 133.3, 131.1, 128.0, 124.6, 122.8, 111.3, 110.8, 109.5, 75.9, 53.5, 31.8, 25.6, 23.8. HRMS(ESI) calcd. for $C_{21}H_{24}N_3O_2$ $[M+H]^+$ 350.1863, found: 350.1862.

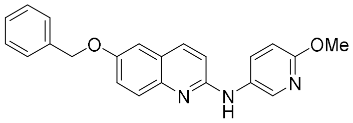

Chemical Formula: $C_{22}H_{19}N_3O_2$
Exact Mass: 357.15

6-(Benzyloxy)-*N*-(6-methoxypyridin-3-yl)quinolin-2-amine (8c, TZ80-40). Following general experimental procedure **A**, a light brown solid (112.5 mg, 63% yield).

^{1}H NMR (400 MHz, $CDCl_3$) δ 8.37 (d, *J* = 2.6 Hz, 1 H), 7.91 (dd, *J* = 8.8, 2.8 Hz, 1 H), 7.81 (d, *J* = 8.9 Hz, 1 H), 7.69 (d, *J* = 9.1 Hz, 1 H), 7.48 (d, *J* = 7.3 Hz, 2 H), 7.41 (t, *J* = 7.3 Hz, 2 H), 7.37 – 7.31 (m, 2 H), 7.07 (d, *J* = 2.6 Hz, 1 H), 6.79 (dd, *J* = 8.8, 3.4 Hz, 2 H), 6.54 (s, 1 H), 5.14 (s, 2 H), 3.95 (s, 3 H). ^{13}C NMR (100 MHz, $CDCl_3$) δ 160.4 (s), 154.7 (s), 153.3 (s), 143.1 (s), 139.5 (s), 136.9 (s), 136.8 (s), 133.3 (s), 131.0 (s), 128.6 (s), 128.1 (s), 128.0 (s), 127.5 (s), 124.5 (s), 121.9 (s), 111.5 (s), 110.8 (s), 107.7 (s), 70.3 (s), 53.6 (s). HRMS(ESI) calcd. for $C_{22}H_{20}N_3O_2$ $[M+H]^+$ 358.1550, found: 358.1545.

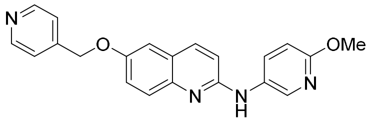

Chemical Formula: $C_{21}H_{18}N_4O_2$
Exact Mass: 358.14

***N*-(6-Methoxypyridin-3-yl)-6-(pyridin-4-ylmethoxy)quinolin-2-amine (8d, TZ80-42).** Following general experimental procedure **A**, a light yellow solid (91.3 mg, 51% yield).

^{1}H NMR (400 MHz, $CDCl_3$) δ 8.64 (d, *J* = 5.9 Hz, 2 H), 8.38 (d, *J* = 2.7 Hz, 1 H), 7.91 (dd, *J* = 9.0, 2.8 Hz, 1 H), 7.80 (d, *J* = 8.9 Hz, 1 H), 7.71 (d, *J* = 9.1 Hz, 1 H), 7.40 (d, *J* = 5.5 Hz, 2 H), 7.35 (dd, *J* = 9.0, 2.8 Hz, 1 H), 7.01 (d, *J* = 2.7 Hz, 1 H), 6.89 – 6.68 (m, 2 H), 6.52 (s, 1 H), 5.17 (s, 2 H), 3.95 (s, 3 H). ^{13}C NMR (100 MHz, $CDCl_3$) δ 161.2 (s), 154.7 (s), 154.2 (s), 150.8 (s), 146.8 (s), 144.0 (s), 140.3 (s), 137.6 (s), 134.0 (s), 131.6 (s), 129.0 (s), 125.1 (s), 122.3 (s), 122.2 (s), 112.4 (s), 111.5 (s), 108.5 (s), 69.1 (s), 54.3 (s). HRMS(ESI) calcd. for $C_{21}H_{19}N_4O_2$ $[M+H]^+$ 359.1503, found: 359.1498.

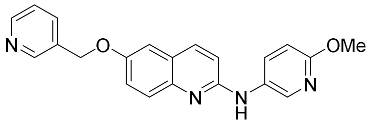

Chemical Formula: $C_{21}H_{18}N_4O_2$
Exact Mass: 358.1430

***N*-(6-Methoxypyridin-3-yl)-6-(pyridin-3-ylmethoxy)quinolin-2-amine (8e, TZ80-66-B).** Following general experimental procedure **A**, a light yellow solid (93.1 mg, 51% yield).

^{1}H NMR (400 MHz, $CDCl_3$) δ 8.73 (s, 1 H), 8.60 (d, *J* = 4.7 Hz, 1 H), 8.37 (d, *J* = 2.6 Hz, 1 H), 7.92 (dd, *J* = 8.8, 2.1 Hz, 1 H), 7.81 (d, *J* = 8.8 Hz, 2 H), 7.70 (d, *J* = 9.1 Hz, 1 H), 7.33 (td, *J* = 8.1, 3.6 Hz, 2 H), 7.06 (d, *J* = 2.6 Hz, 1 H), 6.80 (t, *J* = 9.4 Hz, 2 H), 6.63 (s, 1 H), 5.15 (s, 2 H), 3.94 (s, 3 H). ^{13}C NMR (100 MHz, $CDCl_3$) δ 161.2, 154.9, 154.1, 150.2, 149.7, 143.9, 140.3, 137.6, 136.0, 134.0, 133.1, 131.6, 128.9, 125.1, 124.3, 122.4, 112.4, 111.5, 108.5, 68.5, 54.3. HRMS(ESI) calcd. for $C_{21}H_{19}N_4O_2$ $[M+H]^+$ 359.1503, found: 359.1505.

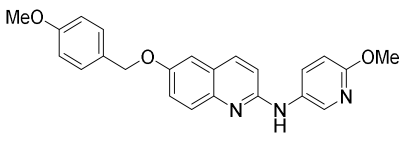

Chemical Formula: $C_{23}H_{21}N_3O_3$
Exact Mass: 387.16

6-((4-Methoxybenzyl)oxy)-*N*-(6-methoxypyridin-3-yl)quinolin-2-amine (8f, TZ80-76). Following general experimental procedure **A**, a light yellow solid (123.8 mg, 64% yield).
^{1}H NMR (400 MHz, $CDCl_3$) δ 8.34 (d, *J* = 2.7 Hz, 1 H), 7.94 – 7.86 (m, 1 H), 7.81 (dd, *J* = 8.8, 1.7 Hz, 1 H), 7.66 (d, *J* = 9.1 Hz, 1 H), 7.38 (d, *J* = 7.8 Hz, 2 H), 7.34 – 7.26 (m, 1 H), 7.05 (s, 1 H), 6.92 (d, *J* = 8.4 Hz, 2 H), 6.78 (ddd, *J* = 8.7, 4.7, 1.3 Hz, 2 H), 6.41 (d, *J* = 11.0 Hz, 1 H), 5.05 (s, 2 H), 3.93 (s, 3 H), 3.81 (s, 3 H). ^{13}C NMR (100 MHz, $CDCl_3$) δ 160.5, 159.5, 154.7, 153.2, 143.1, 139.5, 136.9, 133.3, 131.0, 129.3, 128.8, 128.1, 124.5, 122.0, 114.0, 111.4, 110.8, 107.6, 70.1, 55.3, 53.5. HRMS(ESI) calcd. for $C_{23}H_{22}N_3O_3$ $[M+H]^+$ 388.1656, found: 388.1656.

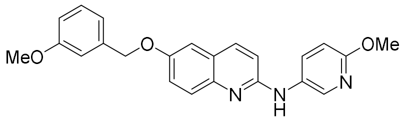

Chemical Formula: $C_{23}H_{21}N_3O_3$
Exact Mass: 387.16

6-((3-Methoxybenzyl)oxy)-*N*-(6-methoxypyridin-3-yl)quinolin-2-amine (8g, TZ80-77). Following general experimental procedure **A**, a light brown solid (116.1 mg, 60% yield).
^{1}H NMR (400 MHz, $CDCl_3$) δ 8.36 (d, *J* = 2.4 Hz, 1 H), 7.91 (dd, *J* = 8.8, 2.7 Hz, 1 H), 7.81 (d, *J* = 8.9 Hz, 1 H), 7.69 (d, *J* = 9.1 Hz, 1 H), 7.40 – 7.28 (m, 2 H), 7.05 (d, *J* = 9.7 Hz, 3 H), 6.88 (d, *J* = 8.3 Hz, 1 H), 6.79 (dd, *J* = 8.8, 3.6 Hz, 2 H), 6.48 (d, *J* = 7.8 Hz, 1 H), 5.12 (s, 2 H), 3.95 (s, 3 H), 3.83 (s, 3 H). ^{13}C NMR (100 MHz, $CDCl_3$) δ 161.2, 160.5, 155.3, 154.0, 143.8, 140.3, 139.2, 137.7, 134.0, 131.7, 130.4, 128.8, 125.2, 122.6, 120.4, 114.2, 113.6, 112.1, 111.5, 108.4, 70.9, 56.0, 54.3. HRMS(ESI) calcd. for $C_{23}H_{22}N_3O_3$ $[M+H]^+$ 388.1656, found: 388.1658.

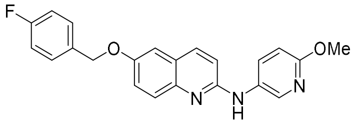

Chemical Formula: $C_{22}H_{18}FN_3O_2$
Exact Mass: 375.1383

6-((4-Fluorobenzyl)oxy)-*N*-(6-methoxypyridin-3-yl)quinolin-2-amine (8h, TZ80-44). Following general experimental procedure **A**, a light yellow solid (125.6 mg, 67% yield).
^{1}H NMR (400 MHz, $CDCl_3$) δ 8.37 (d, *J* = 2.7 Hz, 1 H), 7.91 (dd, *J* = 8.8, 2.8 Hz, 1 H), 7.82 (d, *J* = 8.9 Hz, 1 H), 7.69 (d, *J* = 9.1 Hz, 1 H), 7.45 (dd, *J* = 8.4, 5.5 Hz, 2 H), 7.32 (dd, *J* = 9.1, 2.8 Hz, 1 H), 7.15 – 7.01 (m, 3 H), 6.80 (dd, *J* = 8.8, 6.5 Hz, 2 H), 6.46 (s, 1 H), 5.10 (s, 2 H), 3.95 (s, 3 H). ^{13}C NMR (100 MHz, $cdcl_3$) δ 164.4(s), 162.0 (s), 161.2 (s), 155.2 (s), 154.0 (s), 143.8 (s), 140.3 (s), 137.7 (s), 134.0 (s), 133.3 (d, *J* = 3.2 Hz), 131.6 (s), 130.1 (d, *J* = 8.2 Hz), 128.8 (s), 125.1 (s), 122.6 (s), 116.2 (d, *J* = 21.5 Hz), 112.2 (s), 111.5 (s), 108.4 (s), 70.4 (s), 54.3 (s). HRMS(ESI) calcd. for $C_{22}H_{19}N_3O_2F$ $[M+H]^+$ 376.1456, found: 376.1452.

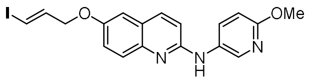

Chemical Formula: $C_{18}H_{16}IN_3O_2$
Exact Mass: 433.0287

(*E*)-6-((3-Iodoallyl)oxy)-*N*-(6-methoxypyridin-3-yl)quinolin-2-amine (8i, TZ90-13). To a 20 mL sealed tube was added by Cs_2CO_3 (1.2 equiv.), the compound **7e** (0.5 mmol, 133 mg), (*E*)-3-bromo-1-iodoprop-1-ene (**10**) (0.5 mmol, 1.0 equiv.), and DMSO (2.0 mL) was stirred at 80 °C for 24 h. And then, the reaction mixture was filtered through a pad of Celite and washed with ethyl acetate (10 mL×3). The filtrate was concentrated. The residue was subjected to column chromatography on silica gel to afford a yellow solid (80.0 mg, 37%).

^{1}H NMR (400 MHz, $CDCl_3$) δ 8.36 (d, *J* = 2.2 Hz, 1 H), 7.90 (dd, *J* = 8.8 Hz, 1.9 Hz, 1 H), 7.81 (d, *J* = 8.9 Hz, 1 H), 7.67 (d, *J* = 9.1 Hz, 1 H), 7.25 (d, *J* = 8.2 Hz, 1 H), 6.96 (s, 1 H), 6.87 – 6.75 (m, 3 H), 6.60-6.55 (m, 2 H), 4.52 (d, *J* = 5.3 Hz, 2 H), 3.94 (s, 3 H). ^{13}C NMR (100 MHz, $CDCl_3$) δ 160.5, 153.9, 153.4, 143.2, 140.5, 139.6, 136.9, 133.4, 130.9, 128.2, 124.4, 121.6, 111.6, 110.8, 107.6, 79.7, 69.9, 53.6. HRMS(ESI) calcd. for $C_{18}H_{17}N_3O_2I$ $[M+H]^+$ 434.0360, found: 434.0354.

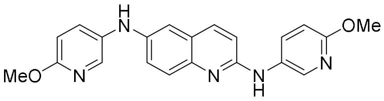

Chemical Formula: $C_{21}H_{19}N_5O_2$
Exact Mass: 373.15

***N*^{2},*N*^{6}-bis(6-methoxypyridin-3-yl)quinoline-2,6-diamine (8j, TZ80-102).**

To a 20 mL sealed tube was added by 5-NH_2-2-methoxypyridine (3.0 equiv), 2-Chloro-6-Br-quinoline (1.0 equiv), $Pd_2(dba)_3$ (0.025 mol%), Xantphos (0.1 mol%) and Cs_2CO_3 (4.0 equiv.). The vessel was evacuated and backfilled with nitrogen (three times), then anhydrous 1,4-dioxane (10 mL) was added. The sealed tube was screw capped and heated to 110 °C. After stirring for 24 hours, the reaction mixture was cooled to room temperature and diluted with ethyl acetate. The reaction mixture was filtered through a pad of Celite and washed with ethyl acetate (10 mL×3). The filtrate was concentrated. The residue was subjected to column chromatography on silica gel to afford a dark green solid (110.0 mg, 59% yield).

^{1}H NMR (400 MHz, $CDCl_3$) δ 8.35 (d, *J* = 2.5 Hz, 1 H), 8.04 (d, *J* = 1.7 Hz, 1 H), 7.88 (dd, *J* = 8.8, 2.5 Hz, 1 H), 7.69 (d, *J* = 8.9 Hz, 1 H), 7.63 (d, *J* = 8.9 Hz, 1 H), 7.46 (d, *J* = 9.9 Hz, 1 H), 7.20 (d, *J* = 8.9 Hz, 1 H), 7.00 (s, 1 H), 6.75 (t, *J* = 8.4 Hz, 3 H), 6.46 (s, 1 H), 5.51 (s, 1 H), 3.92 (s, 6 H). ^{13}C NMR (100 MHz, $CDCl_3$) δ 160.4, 160.2, 152.9, 142.8, 140.4, 139.5, 139.3, 136.6, 133.2, 133.1, 132.9, 131.0, 127.9, 124.9, 122.1, 111.7, 111.2, 110.8, 109.7, 53.54, 53.53. HRMS(ESI) calcd. for $C_{21}H_{20}N_5O_2$ $[M+H]^+$ 374.1612, found: 374.1615.

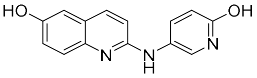

Chemical Formula: $C_{14}H_{11}N_3O_2$
Exact Mass: 253.0851

2-((6-Hydroxypyridin-3-yl)amino)quinolin-6-ol (9, TZ90-23).

To a 20 mL of round-bottom flask were added **7e** (133 mg, 0.5 mmol) and 10 mL of HBr (40%, H_2O). The reaction solution was refluxed for 24 h. And then, the reaction mixture was diluted with ethyl acetate, washed with aqueous sodium bicarbonate. At last, the combined organic layers were washed with brine, dried over Na_2SO_4, filtered, and concentrated. The residue was purified with silica gel chromatography to give a light green solid product (82.8 mg, 62%).

^{1}H NMR (400 MHz, d6-DMSO) δ 11.27 (s, 1 H), 9.46 (s, 1 H), 9.07 (s, 1 H), 8.53 (d, *J* = 2.7 Hz, 1 H), 7.82 (d, *J* = 8.9 Hz, 1 H), 7.66 (d, *J* = 9.7 Hz, 1 H), 7.42 (d, *J* = 8.9 Hz, 1 H), 7.11 (d, *J* = 8.9 Hz, 1 H), 6.97-6.95 (m, 2 H), 6.37 (d, *J* = 9.6 Hz, 1 H). ^{13}C NMR (100 MHz, d6-DMSO) δ 160.1, 152.7, 152.3, 141.2, 136.5, 135.5, 127.1, 124.1, 122.9, 122.8, 120.6, 119.5, 113.8, 109.5.

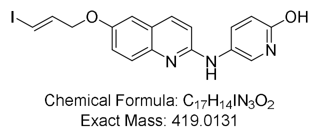

(*E*)-5-((6-((3-iodoallyl)oxy)quinolin-2-yl)amino)pyridin-2-ol (10).

To a solution of **9** (126 mg, 0.50 mmol) in anhydrous DMF (5 mL) was added K_2CO_3 (244 mg, 0.75 mmol), (*E*)-3-bromo-1-iodoprop-1-ene (123 mg, 0.5 mmol) successively. The mixture was stirred at 80 °C in a sealed tube overnight. After cooling down to room temperature, the reaction mixture was concentrated *in vacuo* and the residue was subjected to silica gel chromatography (hexane/EtOAc 2/1 to pure ethyl acetate) to afford product **10** as a light-green solid (43 mg, 21% yield).

^{1}H NMR (400 MHz, Methanol-d_4) δ 8.55 (d, *J* = 2.7 Hz, 1 H), 8.09 (dd, *J* = 8.9 Hz, 2.8 Hz, 1 H), 7.81 (d, *J* = 9.0 Hz, 1 H), 7.54 (d, *J* = 9.0 Hz, 1 H), 7.13 (dd, *J* = 9.0 Hz, 2.7 Hz, 1 H), 6.97 (d, *J* = 2.7 Hz, 1 H), 6.94 – 6.74 (m, 3 H), 6.62 – 6.58 (m, 1 H), 4.69 (dd, *J* = 5.7 Hz, 1.5 Hz, 2 H). ^{13}C NMR (100 MHz, Methanol-d4) δ 158.2, 153.0, 152.8, 141.4, 140.9, 137.2, 136.5, 132.6, 131.8, 126.5, 124.7, 120.5, 113.1, 110.1, 109.3, 78.4, 67.1. HRMS(ESI) calcd. for $C_{17}H_{15}N_3O_2I$ $[M+H]^+$ 420.0203, found: 420.0205.

5. TZ-CLX Synthesis and Characterization

6-(2-(3-(but-3-yn-1-yl)-3H-diazirin-3-yl)ethoxy)-N-(6-methoxypyridin-3-yl)quinolin-2-amine (TZ-CLX).

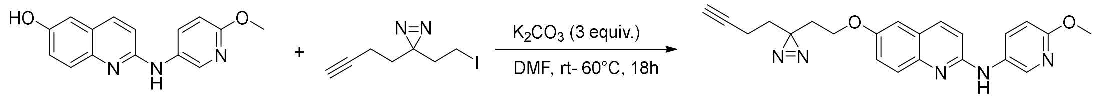

To a solution of 2-((6-methoxypyridin-3-yl)amino)quinolin-6-ol (25 mg, 0.093 mmol, 1 equiv) in DMF (1.5 mL) was added Potassium carbonate (38.8 mg, 0.280 mmol, 3 equiv) and 3-(But-3-yn-1-yl)-3-(2-iodoethyl)-3H-diazirine (46.4 mg, 0.187 mmol, 2 equiv) at room temperature (5 minute). The reaction temperature was increased to 60°C and stirred for 18 h. The reaction was quenched with water (10 mL) and DCM (20 mL) and then layers were separated. The aqueous layer was further extracted 2 times with DCM (20 mL). The combined organic layer was washed with saturated brine solution (50 mL), the organic layer was dried over Na_2SO_4 and concentrated under vacuum. The crude compound was purified by flash column chromatography (gradient of 0-6% MeOH/DCM) to obtain the desired product TZ-CLX in 61% yield. TLC (DCM:MeOH, 95:5 v/v): Rf = 0.20; Yellow Solid; ^{1}H NMR (400 MHz, $CDCl_3$): δ 8.35 (d, *J* = 1.7 Hz, 1 H), 7.90 (dd, *J* = 8.7, 2.1 Hz, 1 H), 7.80 (d, *J* = 8.8 Hz, 1 H), 7.67 (d, *J* = 9.1 Hz, 1 H), 7.25-7.24 (m, 1 H), 6.95 (d, *J* = 1.8 Hz, 1 H), 6.78 (t, *J* = 7.8 Hz, 2 H), 6.53 (bs, 1 H), 3.94 (s, 3 H), 3.89 (t, *J* = 6.1 Hz, 2 H), 2.10-2.06 (m, 2 H), 1.99 (s, 1 H), 1.93 (t, *J* = 6.1 Hz, 2 H), 1.76 (t, *J* = 7.5 Hz, 2 H); ^{13}C NMR (100 MHz, $CDCl_3$): δ 160.5, 154.4, 153.4, 143.2, 139.7, 136.9, 133.4, 131.0, 128.1, 124.5, 121.7, 111.5, 110.8, 107.4, 82.8, 69.2, 62.9, 53.6, 32.9, 32.7, 26.7, 13.3; HRMS-(ESI-TOF) (m/z): calcd for $C_{22}H_{21}N_5O_2$ $[M+H]^+$ 388.1774; found 388.1777.

TZ-CLX Photoaffinity Labeling and Digestion. α-Synuclein fibrils (50 µl, 100 µM final conc.) were added to a 1.5 ml Eppendorf followed by 0.5 µl of TZ-CLX in dimethyl sulfoxide (DMSO) (50 µM final conc.) or 0.5 µl of a DMSO vehicle control. The samples were incubated at 37°C for 1 hr. and irradiated for 30 minutes with 365 nm light (UVP Multiple Ray Lamp, 254 nm, 8 watt; Analytik Jena; Jena, Germany). After irradiation, the crosslinked material was disaggregated via boiling in SDS (100 µl total vol., 25 mM final conc.) and were precipitated using a chloroform

and methanol precipitation.1 The resulting protein pellet was then resolubilized by heating in 20 µl of 8 M urea at 50 °C for 30 minutes. The resulting solution was then diluted by adding either a) 20 µl of 500 mM ammonium bicarbonate and 160 µl of water for trypsin digests or b) 20 µl of a 10X PBS solution and 160 µl of water for GluC digests. The digestion was then allowed to proceed overnight at 37 °C. After digestion, the peptide solutions were acidified by addition of 2 µl of 100%TFA (1% final conc.) and were cleaned up using C18 spin columns (Thermo Fisher Scientific; Waltham, MA). The final solution was concentrated under speed vacuum, yielding a lyophilized peptide pellet.

Bottom-up LC-MS/MS Analysis. Peptide mixtures resulting from labeling and digestion were resolubilized in 25 µl of water with 0.1%TFA and analyzed by a Q-Exactive HF mass spectrometer (Thermo Fisher Scientific; Waltham, MA) coupled to a Dionex Ultimate 3000 UHPLC system (Thermo Fisher Scientific; Waltham, MA) equipped with an in-house made 15 cm long fused silica capillary column (75 µm ID), packed with reversed phase Repro-Sil Pure C18-AQ 2.4 µm resin (Dr. Maisch GmbH, Ammerbuch, Germany). Elution was performed by the following method: a linear gradient from 4 to 38% buffer B (90 min), followed by 95% buffer B (5 min), and re-equilibration from 95 to 4% buffer B (5 min) with a flow rate of 300 nL/min (buffer A: 0.1% formic acid in water; buffer B: 80% acetonitrile with 0.1% formic acid). Data were acquired in data-dependent MS/MS mode. Full scan MS settings were as follows: mass range 200–1600 m/z, resolution 120,000; MS1 AGC target 3E6; MS1 Maximum IT 100. MS/MS settings were: resolution 30,000; AGC target 5E5; MS2 Maximum IT 100 ms; fragmentation was enforced by higher-energy collisional dissociation with stepped collision energy of 25, 27, 30; loop count top 20; isolation window 1.4; MS2 Minimum AGC target 800; charge exclusion: unassigned, 1, 8 and >8; peptide match preferred; exclude isotope on; dynamic exclusion 45 s. Data was searched using Proteome Discoverer (Thermo Fisher Scientific; Waltham, MA) with the mass of the photo-crosslinker supplemented as a variable modification on all amino acids. Peptides identified as crosslinked in DMSO controls (false positives) were removed from the final dataset, and the resulting peptides were normalized for intensity and compared internally for each digest.

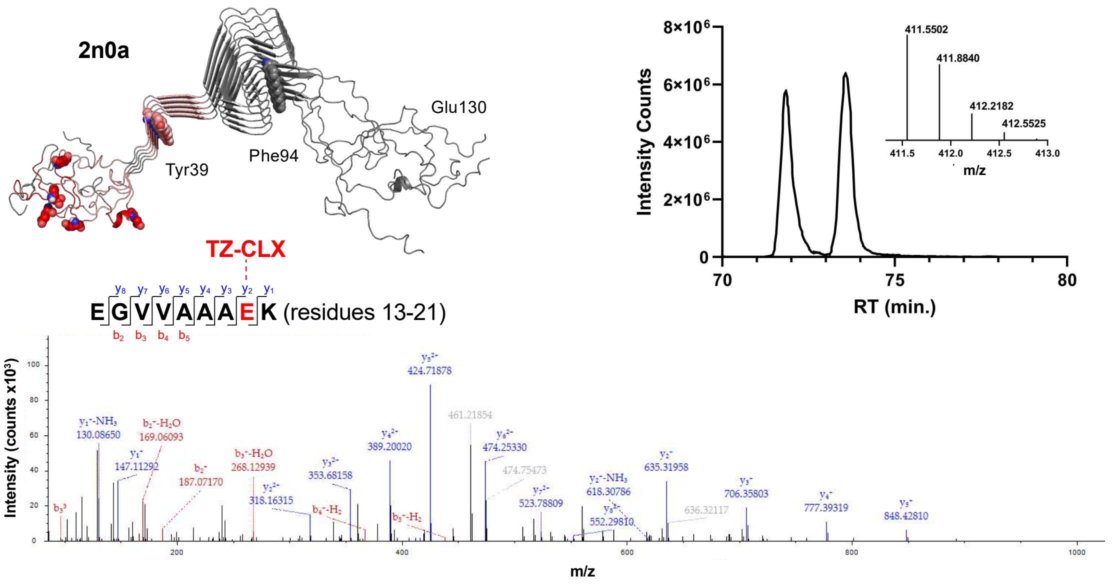

Figure S1. N-terminal Crosslinking of TZ-CLX. Top Left: Top view of crosslinked α-synuclein15-22 peptide (red) in 2n0a ssNMR structure. Crosslinked residue(s), Tyr39 (Site 2), and Phe94 (Site 9) residues are shown in sphere representation. Top Right: α-Synuclein13-21 fragment crosslinked to **TZ-CLX** identified via Orbitrap LC-MS/MS (extracted and parent ion chromatograms.) Bottom: Fragment (MS/MS) spectrum of **TZ-CLX** crosslinked α-synuclein13-21 peptide with annotated b and y ion series.

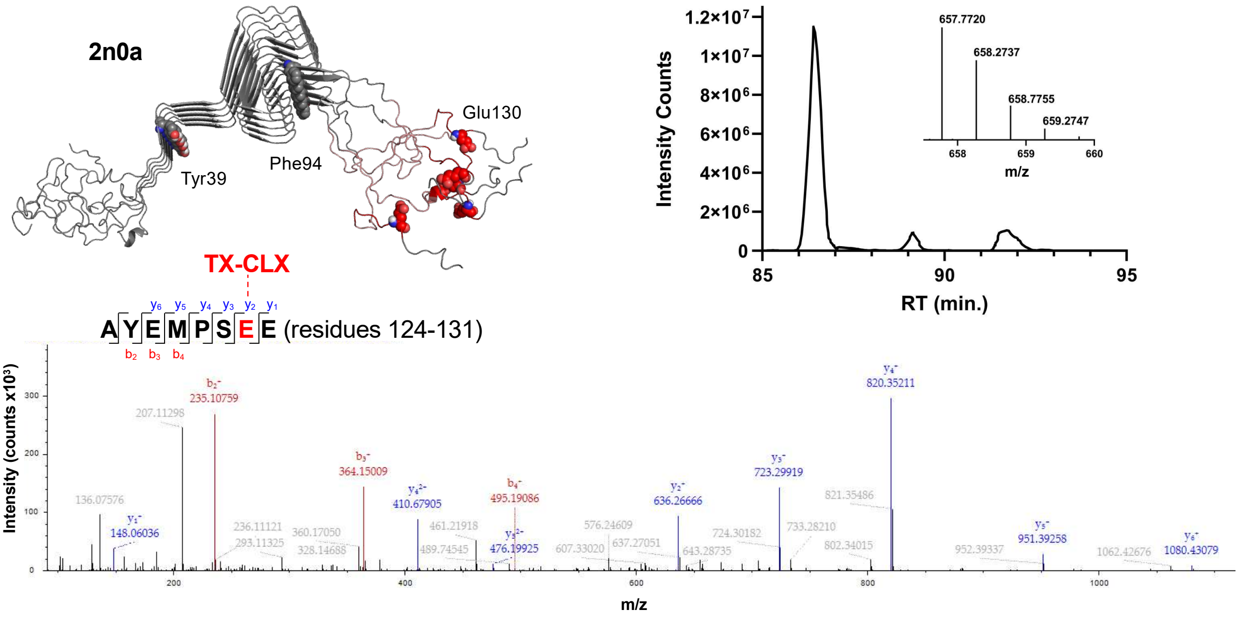

Figure S2. C-terminal Crosslinking of TZ-CLX. Top Left: Top view of crosslinked α-synuclein$_{124-131}$ peptide (red) in 2n0a ssNMR structure. Crosslinked residue(s), Tyr39 (Site 2), and Phe94 (Site 9) residues are shown in sphere representation. Top Right: α-Synuclein$_{124-131}$ fragment crosslinked to **TZ-CLX** identified via Orbitrap LC-MS/MS (extracted and parent ion chromatograms.) Bottom: Fragment (MS/MS) spectrum of **TZ-CLX** crosslinked α-synuclein$_{124-131}$ peptide with annotated b and y ion series.

6. Alpha-Synuclein Binding Affinity Studies

a). Determination of α-synuclein binding activity using radioactive competitive assay [^{3}H]BF2846

Three concentrations of test compounds (10nM, 100nM, and 1μM) were selected and individually mixed with [3H]BF2846 (~4 nM). Each mixture was then separately added to 50 nM of recombinant α-syn fibrils in a working buffer (50 mM Tris-HCl, 0.01% bovine serum albumin (BSA)). The mixture (150μL) was gently mixed by agitation, covered, and then incubated at 37°C for 1.5 h in a nonbinding 96 well plate (Corning, 3605). The mixture was filtered through a Unifilter – 96 harvesting system (PerkinElmer) and then washed three times with 250μL of ice-cold buffer containing 10 mM Tris – HCl (pH7.4), 15mM NaCl, and 20% EtOH. 50μL of scintillation cocktail (MicroScint-20, PerkinElmer) was added to the collected filtrate and counted on a Microbeta system (PerkinElmer). Total binding was measured in the absence of tested compounds, and nonspecific binding was defined by the presence of 100 nM unlabeled BF2846 in the working buffer. The data analysis was carried out in the following equation and graphically presented with a heatmap.

$$Percentage\ inhibition\ (\%) = \left(1 - \frac{Competitor\ Binding\ Counts - Nonspecific\ Binding\ Counts}{Total\ Binding\ Counts - Nonspecific\ BindingCounts}\right) * 100$$

For the full curve of α-syn binding affinity, [^{3}H]BF2846 (~4 nM) was incubated with fixed concentrations of 50 nM α-syn fibrils and ten concentrations of tested compounds (0.05−1000nM). All data points were collected in three individual experiments. The equilibrium dissociation constants,Ki values, were obtained from EC$_{50}$ using thee

quation Ki = EC_{50} / (1 + [radioligand] / KD) by nonlinear regression from GraphPad Prism v.9.3.1

b). Determination of binding activity for AD tissues using [^{3}H]PiB

Three concentrations of test compounds (10nM, 100nM, and 1μM) were selected and individually mixed with [^{3}H]BF2846 (~32 nM). Each mixture was then separately added to 0.5μg/μL of AD tissue homogenate (Tissue ID: 05-215) in Dulbecco's phosphate-buffered saline (DPBS). The mixture (150μL) was gently mixed by agitation, covered, and then incubated at 37°C for 1.5 h in a nonbinding 96 well plate (Corning, 3605). Nonspecific binding was defined by 1μM of unlabeled PiB in DPBS. The following procedures were performed in the same procedure as the α-syn assay using DPBS instead of the working buffer.

c) Direct radioactive competitive binding assay *of radiotracers* [^{11}C]7f, [^{18}F]7j, [^{11}C]8i and [^{125}I]8i and [^{3}H]8i

Affinity determination (Kd) using direct radioactive competitive binding assay with recombinant α-synuclein fibrils, Banner PD tissues, AD brain tissue homogenates. Brain tissue from PD and AD cases was obtained from the Banner Sun Health Research Institute Brain and Body Donation Program of Sun City, Arizona. https://pubmed.ncbi.nlm.nih.gov/25619230/

These direct binding assays used a fixed concentration of either α-synuclein fibrils or AD tissue and the radioligand (**[^{11}C]7f, [^{18}F]7j, [^{11}C]8i and [^{125}I]8i, [^{3}H]8i**) and varying concentration ranges of corresponding homologous non-radiolabeled standard reference compound. In brief, the standard homologous reference compound was diluted in 30 mM Tris-HCl pH 7.4, 0.1% BSA. Reactions were incubated at 37 °C for 1 h before quantifying bound radioligand. Bound and free radioligands were separated by vacuum filtration through 1.0 μm glass fiber filters in 96-well filter plates (Millipore), followed by three 200 μL washes with ice-cold assay buffer. Filtrates containing the bound ligand were mixed with 150 μL of Optiphase Supermix scintillation cocktail (PerkinElmer) and counted immediately. All data points were performed in triplicate. The dissociation constant (Kd) and the maximal number of binding sites (Bmax) values were determined by fitting the data to the equation. Bound = (Bmax*[Radioligand])/([Radioligand] + [Unlabeled compound] + Kd) + Bottom by nonlinear regression using GraphPad Prism software (version 4.0), where (Bottom) is nonspecific binding and [Radioligand], [Unlabeled compound], and Kd is expressed in nM

7. Radiochemistry

Production of [^{11}C]CH_3I followed the reported method. Briefly, [^{11}C]CH_3I was produced on-site from [^{11}C]CO_2 using a GE PETtrace MeI Microlab. Up to 1.4 Ci of [^{11}C]carbon dioxide was produced from the JSW BC-16/8 cyclotron by irradiating a gas target of 0.5% O_2 in N_2 for 15 – 30 min with a 40 μA beam of 16 MeV protons in the Barnard Cyclotron Facility of Washington University School of Medicine. After the [^{11}C]CO_2 was converted to [^{11}C]CH_4 using a nickel catalyst [Shimalite-Ni (reduced), Shimadzu, Japan P.N.221-27719] in the presence of hydrogen gas at 360 °C; the [^{11}C]CH_4 was further converted to [^{11}C]CH_3I by reaction with iodine in the gas phase at 690 °C. Approximately 12 min following the end-of-bombardment (EOB), several hundred millicuries of [^{11}C]CH_3I were delivered in the gas phase to the hot cell where the radiosynthesis was accomplished. Radioligand [^{3}H]**8i** was custom synthesized by Novandi Chemistry AB Forskargatan 20J, Södertälje, SE-151 36, Sweden via a subcontract.

Radiosynthesis of [^{11}C]7f ([^{11}C]TZ55-107)

Approximately 1.0 mg of precursor **7e** was placed in a V-shape reaction vessel with an aqueous NaOH solution (5 M) in MeCN (300 µL). [^{11}C]CH_3I was bubbled into the reaction vessel and the reaction mixture was heated to 80 ^{o}C for 5 min. After quenching wit 1.8 mL of HPLC mobile phase (48% acetonitrile in 0.1 M ammonium formate buffer, pH 4.5). The reactive mixture was loaded onto a C18 column (Agilent Zorbax SB-C18, 5 µm, 250×9.6 mm), then eluted from the column using above-mentioned HPLC mobile phase at a flow rate of 4.0 mL/min. A 100 mL of glass vial prefilled with 60 mL sterile water was used to collect the radioactive product from 12-13 min and then passed through a C18 Sep-Pak Plus cartridge with nitrogen gas assistance. The trapped product was eluted using 0.6 mL of ethanol and 5.4 mL of saline to formulate the injection dose. The product was authenticated using an analytical HPLC system (Agilent SB-C18 analytic column, 250 mm×4.6 mm; mobile phase of 75% acetonitrile in 0.1 M ammonium formate buffer, pH 4.5; flow rate of 1.0 mL/min; UV wavelength of 254 nm; t_R = 3.9 min) by co-injecting with standard reference compound **7f**. The radiochemical yield was about 45%, the radiochemical purity was >99%, and the specific activity was >74 GBq/µmol (decay corrected to EOB).

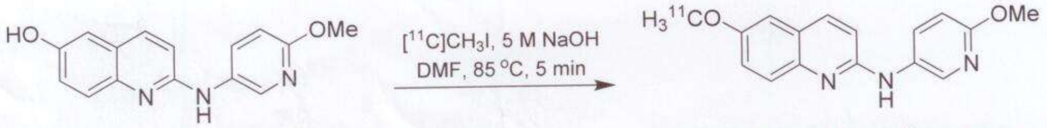

Chemical Formula: $C_{15}H_{13}N_3O_2$
Molecular Weight: 267.28800
TZ61-23

Chemical Formula: $C_{15}{}^{11}CH_{15}N_3O_2$
Molecular Weight: 280.31543
[^{11}C]**TZ55-107**

Title: [^{11}C]**TZ55-107**			
Batch# [^{11}C]**TZ55-107**	Precursor 1.3 mg		Date: 08 / 17 /2016
Cyclotron: JSW	Units: 800/1000 (800 circled)		EOB 12:30 AM/PM (PM circled)
Chemist: XY		PI: Tu	
Detector Reading HPLC 1200-1400 psi	Ending of Trapping: 22.5 mV 12:51:32		
	Ending of heating: 18.2 mV 12:57:40		
	After Injection: 8.3 mV 12:59:43		
Yield Before Filtration:		Reaction time:	
Delivery for study: 3.6 mCi	Time: 13:24	Vol: ________	
Radiocemical Purity: ______________%			
Specific Activity: 646 Ci/mmol ______mCi in ______μL at______			
Comments: collected 64.8 mci @ 13:18:16<br>delivered 3.64 mci @ 13:24:30 in 150 uL<br>QC 5.76 mci @ 13:25:20 in 0.20 mL Mass 12.50. SA 646 ci/mmol			

Procedures:

1 – 2 mg (3.7 - 7.5 μmol) of precursor was dissolved in 200 μL anhydrous DMF, 3 μL of 5 M aqueous solution (15 μmol) was added 5 min prior to [^{11}C]MeI trapping, vortexed. [^{11}C]MeI was bubbled into the solution and the reaction was heated at **85 °C for 5 min**. Shaking was applied to the reaction vessel two times with the aid of a long clamp. 1.7 mL mobile phase (**48% acetonitrile in 52% 0.1 M ammonium formate, pH 4.5**) was added to quench the reaction and the mixture was load to HPLC column for purification (**Agilent Zorbax C18 column, 250 × 9.6 mm, 5 μ, UV at 254 nm, flow rate 4 mL/min**). The retention time for the precursor was 6-7 min, for the standard was 11-12 min.

The desired fraction was collected into a water bottle prefilled with 50 mL water and the solution was passed to a C18 Sep-Pak, the Sep-Pak was rinsed with additional 20 mL water. The product was eluted out with EtOH to make a solution at a concentration of 2 – 3 mCi/0.2 – 0.3 mL for the assays (7 – 15 mCi/mL).

1

Lab name: SRI Instruments
Client: Valued Customer
Analysis date: 08/17/2016 12:53:30
Method: Syringe Injection
Description: Radioactive channel
Column: RESTEK 15METER MXT-1
Carrier: HELIUM AT 5 PSI
Data file: 11C-TZ55-107-0.CHR ()
Sample: RUN1
Comments: TYPE YOUR COMMENTS HERE

Temperature program:

Init temp	Hold	Ramp	Final temp

Events:

Time	Event
0.000	ZERO
0.000	SOUND

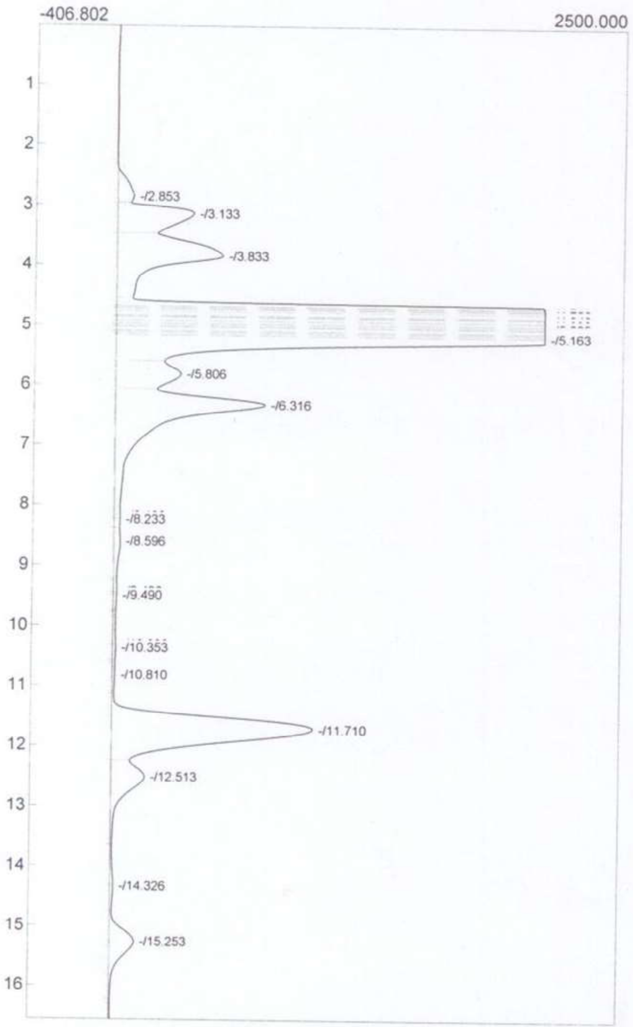

Component	Retention	Area	Height	External	Units
		0.0000		0.0000	

Lab name: SRI Instruments
Client: Valued Customer
Analysis date: 08/17/2016 12:53:30
Method: Syringe Injection
Description: Radioactive channel
Column: RESTEK 15METER MXT-1
Carrier: HELIUM AT 5 PSI
Data file: 11C-TZ55-107-0.48ACN-4.5-RAD01-081716.chr ()
Sample: RUN1
Comments: TYPE YOUR COMMENTS HERE

Temperature program:

Init temp	Hold	Ramp	Final temp

Events:

Time	Event
0.000	ZERO

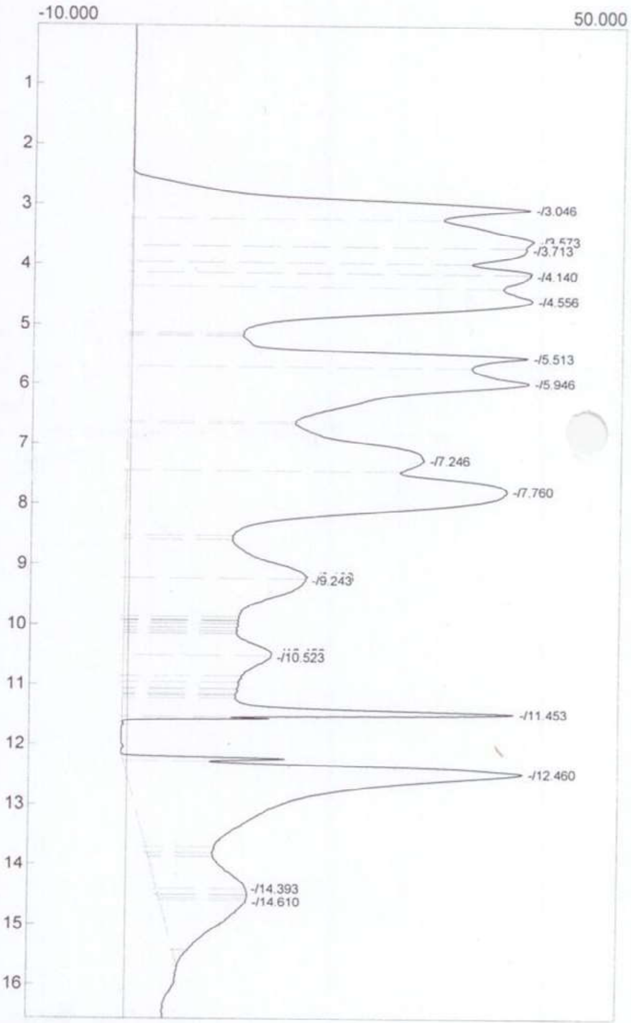

Component	Retention	Area	Height	External	Units
		0.0000		0.0000	

[11C]TZ55-107

Sample Name:	[11C]TZ55-107	*Column*	Agilent USCL034814
Flow Rate(mL/min):	**1.00**		
Sample Type:	**unknown**	*Mobile Phase:*	**70% ACN**
Control Program:	**C-TZ55-107**		**30% 0.1M Formate buffer pH=6.5**
Quantif. Method:	**C-TZ55-107**		
Recording Time:	**8/17/2016 13:46**		
Run Time (min):	**8.00**		

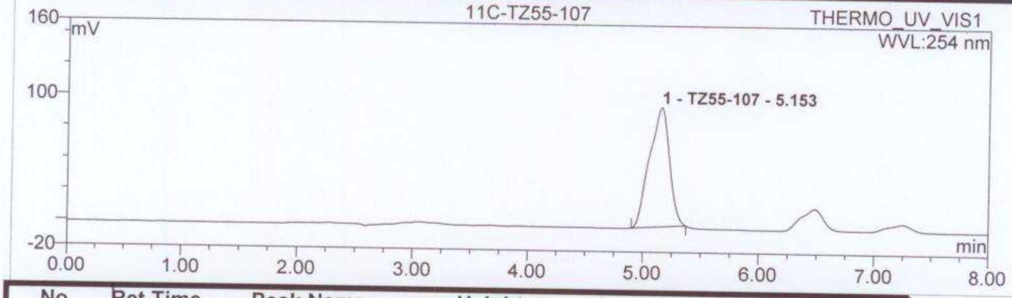

No.	Ret.Time min	Peak Name	Height mV	Area mV*min	Amount ug/mL	Rel.Area %
1	5.15	TZ55-107	94.688	19.02923	12.5008	100.00
Total:			94.688	19.029		100.00

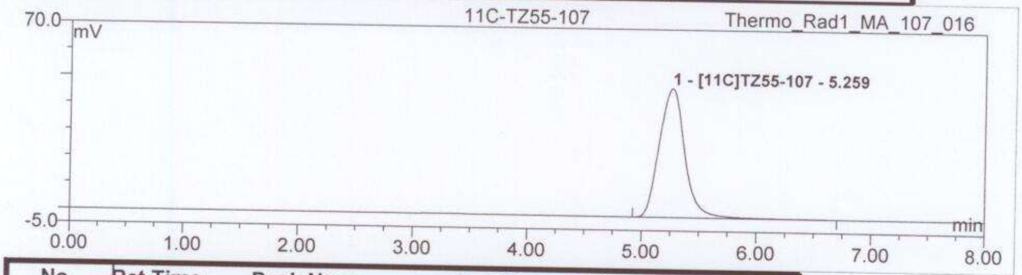

No.	Ret.Time min	Peak Name	Height mV	Area mV*min	Rel.Area %
1	5.26	[11C]TZ55-107	47.988	12.436	100.00
Total:			47.988	12.436	100.00

Reported by Initials / Date XY, 08/17/2016

Signoff by Signature / Date Xiuyi Yue, 08/17/2016

F-TZ19-66B/Integration

Chromeleon (c) Dionex 1996-2001
Version 6.80 SR12 Build 3578 (207169)

[11C]TZ55-107 COINJ

Sample Name:	[11C]TZ55-107	*Column*	Agilent USCL034814
Flow Rate(mL/min):	**1.00**		
Sample Type:	**unknown**	*Mobile Phase:*	**70% ACN**
Control Program:	**C-TZ55-107**		**30% 0.1M Formate buffer pH=6.5**
Quantif. Method:	**C-TZ55-107**		
Recording Time:	**8/17/2016 13:56**		
Run Time (min):	**8.00**		

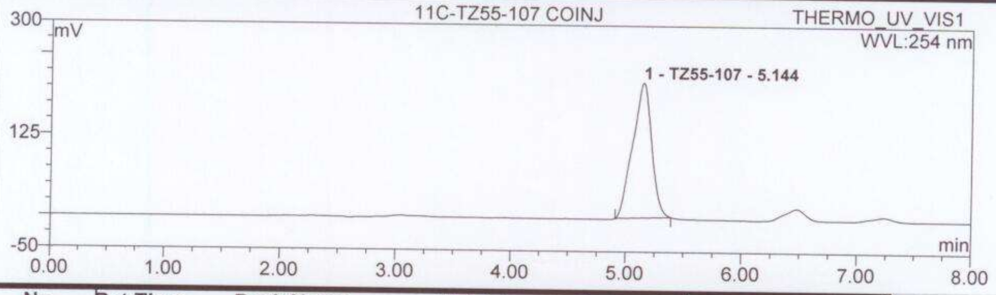

No.	Ret.Time min	Peak Name	Height mV	Area mV*min	Amount ug/mL	Rel.Area %
1	5.14	TZ55-107	209.029	39.74716	26.1109	100.00
Total:			209.029	39.747		100.00

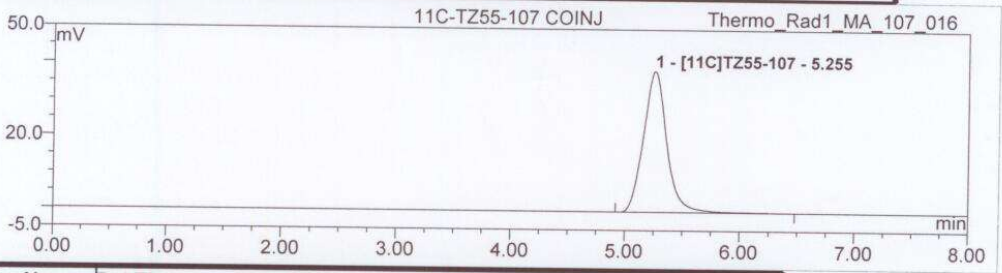

No.	Ret.Time min	Peak Name	Height mV	Area mV*min	Rel.Area %
1	5.26	[11C]TZ55-107	38.601	9.163	100.00
Total:			38.601	9.163	100.00

Reported by Initials / Date Xy , 08/17/2016

Signoff by Signature / Date Xuyi Yue, 08/17/2016

F-TZ19-66B/Integration

Chromeleon (c) Dionex 1996-2001
Version 6.80 SR12 Build 3578 (207169)

Radionuclidic Identity Analysis on 8/17/2016 at 13:58

mCi Reading	Time(min)
1.145	0.495
1.125	1.004
1.107	1.512
1.087	2.021
1.069	2.530
1.051	3.039
1.031	3.548
1.014	4.057
0.996	4.566
0.980	5.075
0.964	5.584
0.946	6.094
0.929	6.603
0.914	7.112
0.898	7.621
0.883	8.130
0.867	8.640
0.852	9.149
0.838	9.658
0.824	10.168

Calculated Halflife(sec)	1219.293
Theoretical Halflife(sec)	1223.100
Calculated Halflife(min)	20.322
Theoretical Halflife(min)	20.385
Percent Difference	0.311

11C-TZ55-107

Batch ID 11C-TZ55-107-0817

Initials Xy

Date 8/17/2016

Sign Off Full Name: Xiuyi Yue

Sign Off Date: 08/17/2016

End of Decay: 13:53

Decay performed on: CRC712MH

Radiosynthesis of [^{11}C]8i ([^{11}C]TZ61-84)

Approximately 1.0 mg of precursor (**10**) was placed in a V-shape reaction vessel with KOH solid (1 mg) in DMF (300 µL). Then, the mixture was shaken for 1 minute using a Vertx mixer. [^{11}C]CH_3OTf was bubbled into the reaction vessel and the reaction mixture was heated to 90 °C for 5 min. After quenching with 1.8 mL of HPLC mobile phase (65% acetonitrile in 0.1 M ammonium formate, buffer, pH 4.5). The reactive mixture was loaded onto a C18 column (Agilent Zorbax SB-C18, 5 µm, 250×9.6 mm), then eluted from the column using above-mentioned HPLC mobile phase at a flow rate of 4.0 mL/min. A 100 mL of glass vial prefilled with 60 mL sterile water was used to collect the radioactive product from 18 -19 min and then passed through a C18 Sep-Pak Plus cartridge with nitrogen gas assistance. The trapped product was eluted using 0.6 mL of ethanol and 5.4 mL of saline to formulate the injection dose. The product was authenticated using an analytical HPLC system (Agilent SB-C18 analytic column, 250 mm×4.6 mm; mobile phase of 70%acetonitrile in 0.1M ammonium formate buffer, pH 4.5; flow rate of 1.0 mL/min; UV wavelength of 254 nm; tR =5.0 min) by co-injecting with standard reference compound **8i (TZ61-84)**. The radiochemical yield was about 16%, the radiochemical purity was >99%, and the specific activity was >74 GBq/µmol. (decay corrected to EOS).

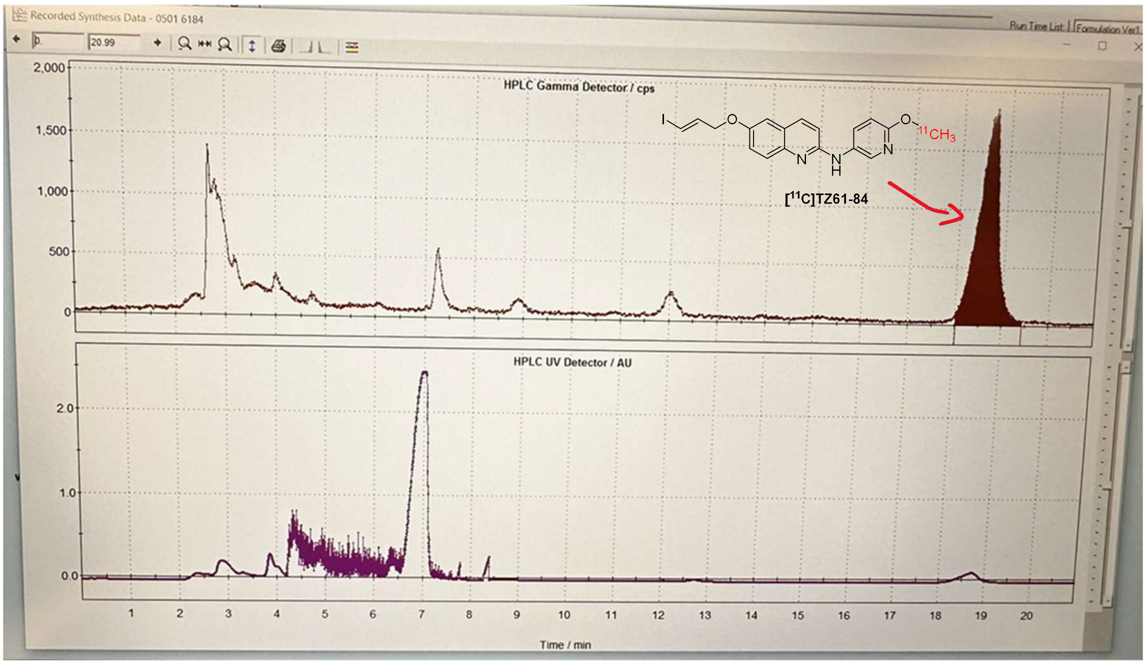

HPLC FOR 11C-11C-TZ6184

Sample Name:	11C-TZ6184		
Flow Rate(mL/min):	**1.00**	Column SN:	USCL034814
Sample Type:	**standard**	*Mobile Phase:*	**70% ACN**
Control Program:	11C-TZ6184		**30% 0.1M Formate buffer pH=4.5**
Quantif. Method:	11C-TZ6184		
Recording Time:	**2/19/2020 9:26**		
Run Time (min):	**8.00**		

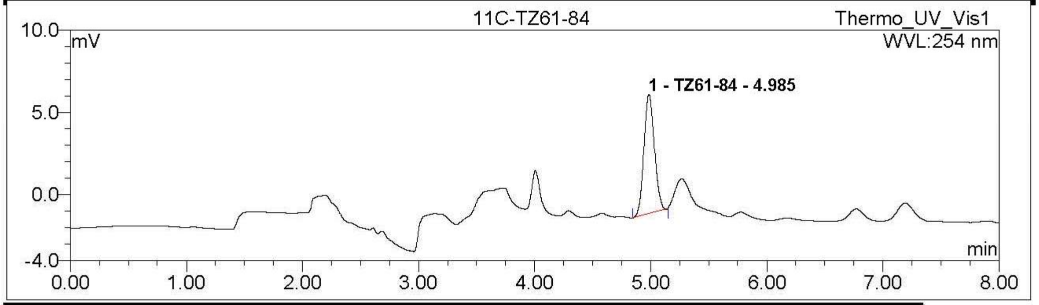

No.	Ret.Time min	Peak Name	Height mV	Area mV*min	Amount ug/mL	Rel.Area %
1	4.98	TZ61-84	7.200	0.74790	0.5865	100.00
**Total:**			7.200	0.748		100.00

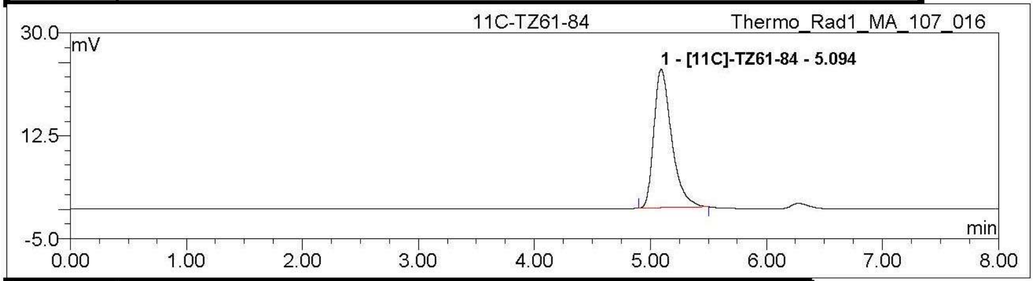

No.	Ret.Time min	Peak Name	Height mV	Area mV*min	Rel.Area %
1	5.09	[11C]-TZ61-84	23.590	4.188	100.00
**Total:**			23.590	4.188	100.00

Reported by Initials / Date __________ / __________

Signoff by Signature / Date ____________________ / __________

HPLC FOR 11C-11C-TZ6184 COINJ

Sample Name:	11C-TZ6184		
Flow Rate(mL/min):	**1.00**	Column SN: USCL034814	
Sample Type:	**standard**	*Mobile Phase:*	**70% ACN**
Control Program:	11C-TZ6184		**30% 0.1M Formate buffer pH=4.5**
Quantif. Method:	11C-TZ6184		
Recording Time:	**2/19/2020 9:36**		
Run Time (min):	**8.00**		

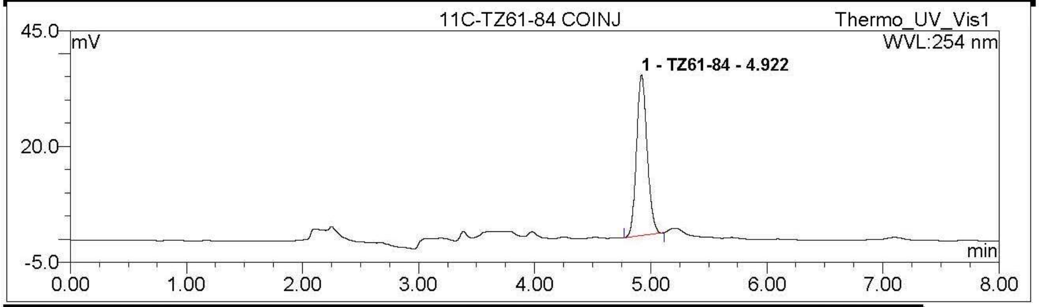

No.	Ret.Time min	Peak Name	Height mV	Area mV*min	Amount ug/mL	Rel.Area %
1	4.92	TZ61-84	34.648	3.65533	2.8662	100.00
**Total:**			34.648	3.655		100.00

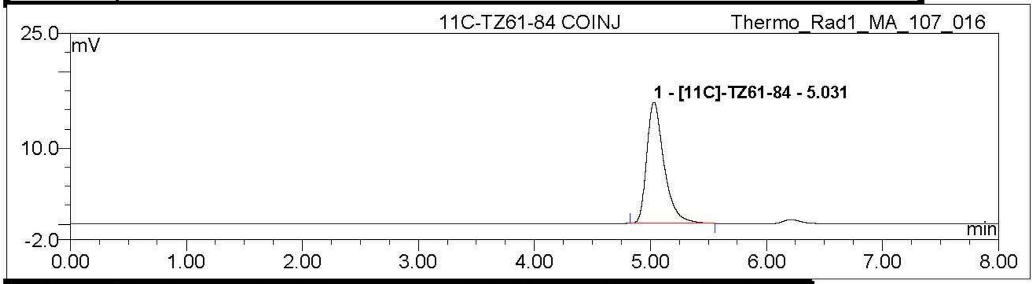

No.	Ret.Time min	Peak Name	Height mV	Area mV*min	Rel.Area %
1	5.03	[11C]-TZ61-84	15.861	2.747	100.00
**Total:**			15.861	2.747	100.00

Reported by Initials / Date __________ / __________

Signoff by Signature / Date ____________________ / __________

F-TZ19-66B/Integration

Chromeleon (c) Dionex 1996-2001
Version 6.80 SR12 Build 3578 (207169)

Radionuclidic Identity Analysis on 2/19/2020 at 10:01

mCi Reading	Time(min)
0.043	0.495
0.042	1.004
0.042	1.512
0.041	2.021
0.040	2.530
0.039	3.038
0.038	3.547
0.038	4.056
0.037	4.565
0.037	5.074
0.036	5.582
0.035	6.091
0.035	6.600
0.034	7.109
0.034	7.618
0.033	8.126
0.033	8.635
0.032	9.144
0.031	9.653
0.031	10.161

**Calculated Halflife(sec)**	1188.946
**Theoretical Halflife(sec)**	1223.100
**Calculated Halflife(min)**	19.816
**Theoretical Halflife(min)**	20.385
**Percent Difference**	2.792

11C-TZ6184

**Batch ID**	JG02192020
**Initials**	
**Date**	2/19/2020
**Sign Off Full Name:**	
**Sign Off Date:**	
**End of Decay:**	9:38

Decay performed on: CRC712MH

Radiosynthesis of [^{18}F]7j ([^{18}F]TZ61-84)

A solution of precursor **7e** (2 mg) and Cs_2CO_3 (2.0 mg) in DMSO was added to reaction vessel containing **[^{18}F]12**. (**[^{18}F]12** was prepared according to the reference: X. Yue, D. D. Dhavale, J. Li, Z. Luo, J. Liu, H. Yang, R. H. Mach, P. T. Kotzbauer, Z. Tu. *Bioorg. Med. Chem. Lett.* **2018**, *28*, 1011–1019). The vessel was capped and heated at 100 °C for 15 min. Subsequently, the reaction mixture was diluted with 2.7 mL of HPLC mobile phase (38% acetonitrile in 0.1m ammonium formate buffer, pH 4.5) and loaded onto a C18 column (Agilent SB-C18, 250 mm×10 mm), then eluted from the column using above mentioned HPLC mobile phase at a flow rate of 4.0 mL/min. A 100 mL of glass vial that has 50 mL of sterile water was used to collect the radioactive product from 25–26 min and then passed through a C18 Sep-PakPlus cartridge with nitrogen gas assistance. The trapped product was eluted using 0.6 mL of ethanol and 5.4 mL of saline to formulate the injection dose. The product was authenticated using an analytical HPLC system (Agilent SB-C18 analytic column, 250 mm×4.6 mm; mobile phase of 70%acetonitrile in 0.1m ammonium formate buffer, pH 4.5; flow rate of 1.0 mL/min; UV wavelength of 254 nm; t_R =4.8 min) by co-injecting with standard reference compound **7j**. The radiochemical yield was about 35%, the radiochemical purity was >98%, and the specific activity was >55 GBq/μmol(decay corrected to EOB).

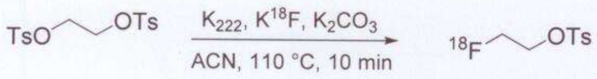

Chemical Formula: $C_9H_{11}{}^{18}FO_3S$
Molecular Weight: 217.24

Procedure			
Precursor: 5.0 mg<br>Measured 7.2 mg	ACN: 0.2 mL	K_2CO_3:<br>from **^{18}F Treated**	K_{222}: 6 - 8 mg<br>Measured 8.2 mg

Batch #:	Date: 5 / 19 / 2017
Cyclotron: ECF	EOB: 10:20 AM
Chemist: XY	PI: Zhude Tu

Activity received: 225 mCi @ 10:45 in 0.4 mL

Transferred to rxn: 201.5 mCi @ 10:58

Azeotropic drying 3× 1.0 mL acetonitrile, heated in 110 °C oil bath.

After dry: 171.4 mCi @ 11:23

Reaction heated at 110 °C; time: 11:24 to 11:34

HPLC column: Agilent SB-C18

Mobile phase: 50:50 ; Flow rate: 4.0 mL/min; Pressure: 1500-1600 psi

sep-pak 60.6 12:05

Activity washed off by 2 + 1 mL ether; 36.2 mCi @ 12:04 before dry.

31.0 mCi @ 12:25 after dried with stacked Na_2SO_4 sep-Pak.

Comments: Quench solvent 3 mL.

After Et_2O phase is transferred out, added another 1.5 mL to extract.

HPLC condition: Agilent Zorbax SB-C18, 5 μ, 250 × 9.4 mm; 50% ACN in 0.1M AMF pH = 6.5 buffer, 4 mL/min, UV = 254 nm. Retention time for product: ~ 10 min, precursor: ~ 23 - 24 min.

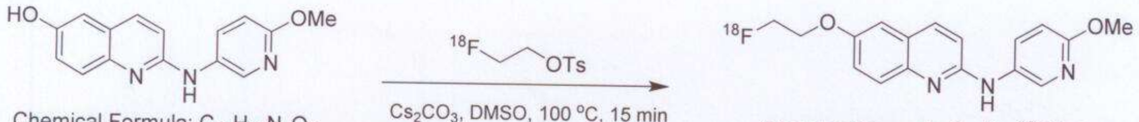

Chemical Formula: $C_{15}H_{13}N_3O_2$
Molecular Weight: 267.29
TZ61-23

Chemical Formula: $C_{17}H_{16}{}^{18}FN_3O_2$
Molecular Weight: 312.33
Log P: 3.62
[^{18}F]**TZ61-44**

Title: [^{18}F]TZ61-44		
Batch# [^{18}F]TZ61-44 pre lib[illegible]		Date: 5/10/17
Cyclotron: TR19	Activity Requested: 200 mCi	EOB: 10:30
Chemist: XY	PI: Tu	
Detector Reading:	[^{18}F]Treated KF:	mCi @
HPLC, 700-800 [illegible] Transferred to rxn: ______ mCi @ ______		
[^{18}F]FEtOTs was used for the second step reaction.		
[^{18}F]FEtOTs after drying: 31.0 mCi @ 12:25		
Radiochemical Purity: ______ %		
Specific Activity: ~~[illegible]~~ 1586 Ci/mmol(3.01 mCi in 200 μL at 13:40 ; μg/mL ______)		

Note: Collected 12.40 mci @ 13:18
Eluted out 3.94 mci @ 13:20 in 0.8 ml EtOH
delivered 2.40 mci @ 13:23 in 150 uL
QC 3.01 mci @ 13:40 in 200 uL
Mass 2.96. SA 1586 Ci/mmol

Procedure:

To the reaction vial containing dried 2-[^{18}F]fluoroethyl tosylate was added 2-3 mg of precursor, 3 – 4 mg of Cs_2CO_3 in a solution of 300 μL DMSO. The reaction was placed in a 100 °C oil-bath and heated for 15 min, shaking occasionally. The reaction vessel was removed from the oil-bath, quenched with 3.0 mL of the HPLC mobile phase (**38% MeCN in 62% 0.1 M ammonium formate buffer, pH = 4.5**), and injected onto the HPLC column (Agilent SB-C18 250 x 9.6 mm, 5 μ, UV = 254 nm, 4.0 mL/min). The desired fraction was collected into a water bottle prefilled with 50 mL steric water. The dilute fraction was passed through a C18 Sep-pak and rinsed with another 20 mL steric water. The product was eluted with USP grade ethanol and delivered for competitive binding assay.

The retention time for the target fraction is ________ min.

Lab name: SRI Instruments
Client: Valued Customer
Analysis date: 05/10/2017 12:51:54
Method: Syringe Injection
Description: UV detector
Column: RESTEK 15METER MXT-1
Carrier: HELIUM AT 5 PSI
Data file: F-TZ61-44-0.CHR ()
Sample: RUN1
Comments: TYPE YOUR COMMENTS HERE

Temperature program:

Init temp	Hold	Ramp	Final temp

Events:

Time	Event
0.000	ZERO
0.000	SOUND

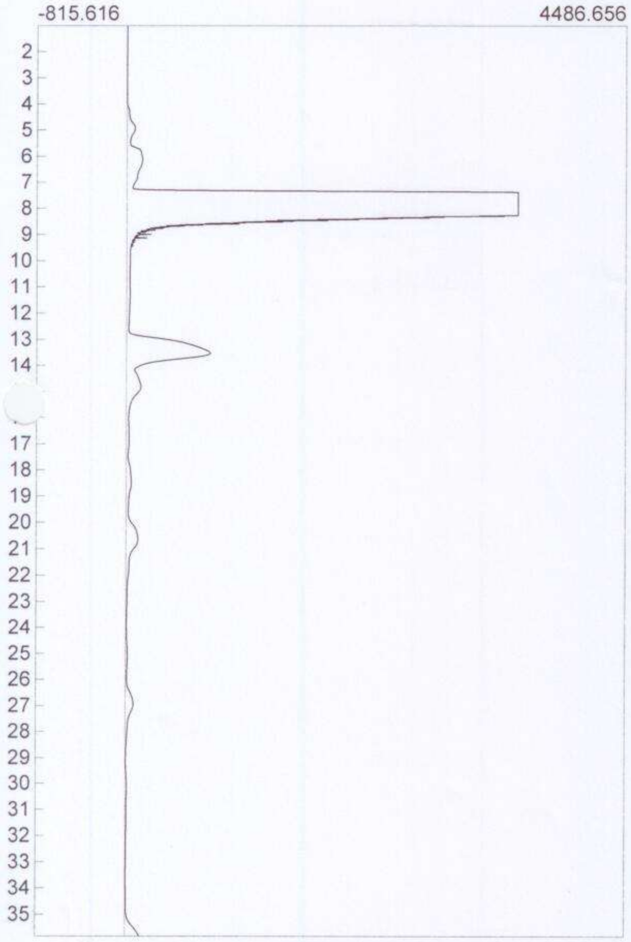

Component	Retention	Area	Height	External	Units
		0.0000		0.0000	

Lab name: SRI Instruments
Client: Valued Customer
Analysis date: 05/10/2017 12:51:54
Method: Syringe Injection
Description: Radioactivity detector
Column: RESTEK 15METER MXT-1
Carrier: HELIUM AT 5 PSI
Data file: F-TZ61-44-0.42ACN-pH4.5-RAD04-03102017.chr ()
Sample: RUN1
Comments: TYPE YOUR COMMENTS HERE

Temperature program:

Init temp	Hold	Ramp	Final temp

Events:

Time	Event
0.000	ZERO

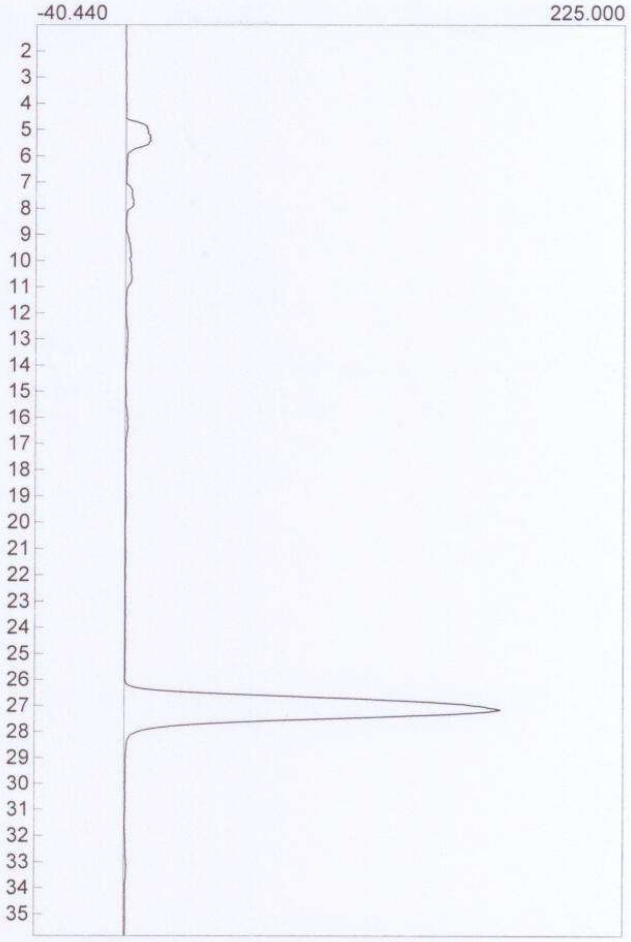

Component	Retention	Area	Height	External	Units
		0.0000		0.0000	

Lab name: SRI Instruments
Client: Valued Customer
Analysis date: 05/10/2017 11:45:09
Method: Syringe Injection
Description: UV detector
Column: RESTEK 15METER MXT-1
Carrier: HELIUM AT 5 PSI
Data file: FEOTs-uv41-05102017.CHR ()
Sample: RUN1
Comments: TYPE YOUR COMMENTS HERE

Temperature program:

Init temp	Hold	Ramp	Final temp

Events:

Time	Event
0.000	ZERO
0.000	SOUND

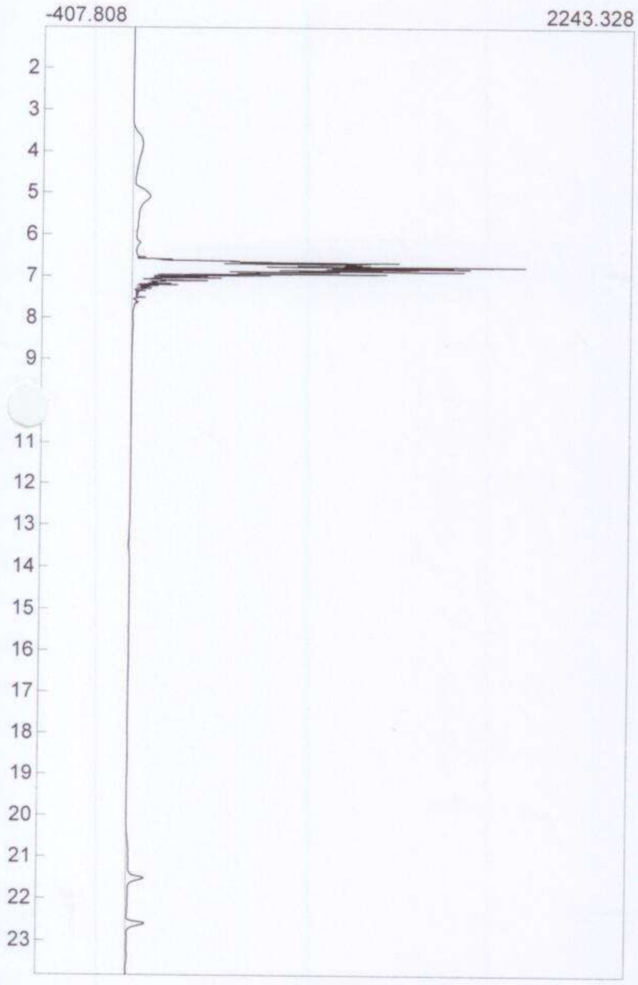

Component	Retention	Area	Height	External	Units
		0.0000		0.0000	

Lab name: SRI Instruments
Client: Valued Customer
Analysis date: 05/10/2017 11:45:09
Method: Syringe Injection
Description: Radioactivity detector
Column: RESTEK 15METER MXT-1
Carrier: HELIUM AT 5 PSI
Data file: FEtOTs-rad41-05102017.chr ()
Sample: RUN1
Comments: TYPE YOUR COMMENTS HERE

Temperature program:

Init temp	Hold	Ramp	Final temp

Events:

Time	Event
0.000	ZERO

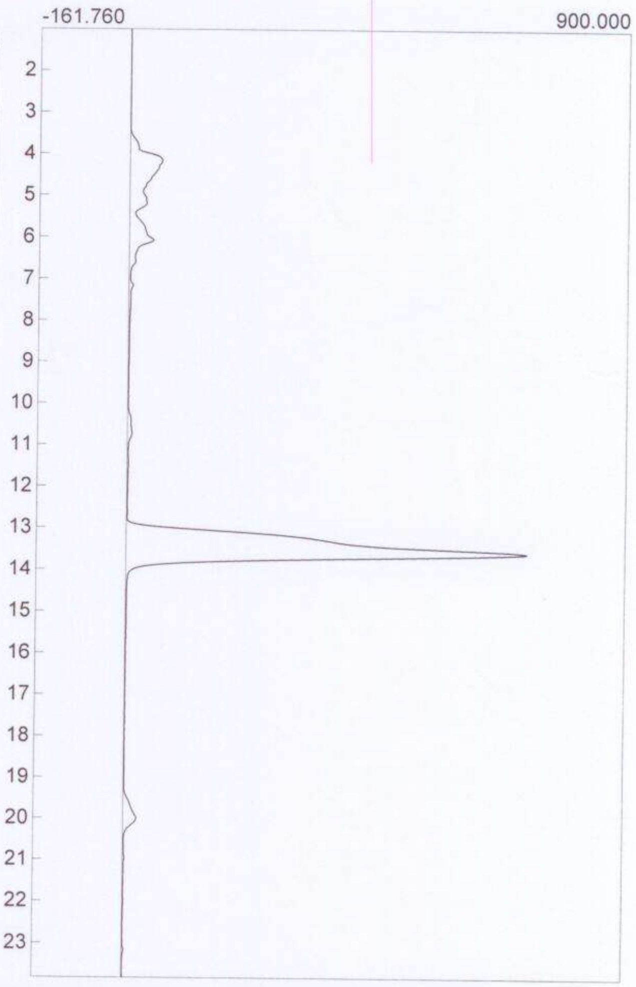

Component	Retention	Area	Height	External	Units
		0.0000		0.0000	

TZ61-44

Sample Name:	TZ61-84	*Column*	Agilent USCL0314814
Flow Rate(mL/min):	**1.00**		
Sample Type:	**standard**	*Mobile Phase:*	**70% ACN**
Control Program:	**F-TZ61-44**		**30% 0.1M Formate buffer pH=4.5**
Quantif. Method:	**F-TZ61-44**		
Recording Time:	**5/10/2017 14:04**		
Run Time (min):	**8.00**		

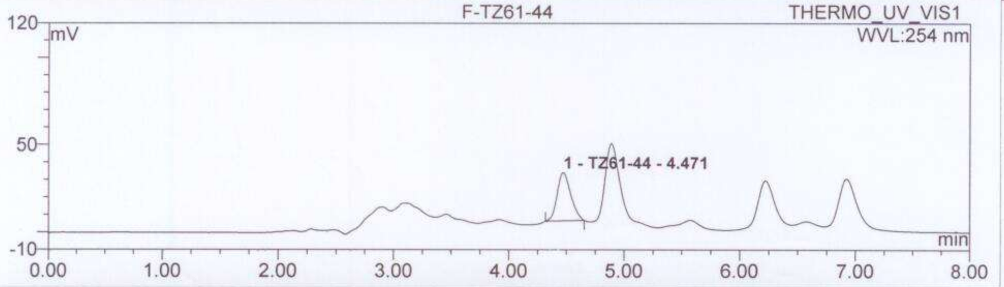

No.	Ret.Time min	Peak Name	Height mV	Area mV*min	Amount ug/mL	Rel.Area %
1	4.47	TZ61-44	27.716	3.78513	2.9680	100.00
Total:			27.716	3.785		100.00

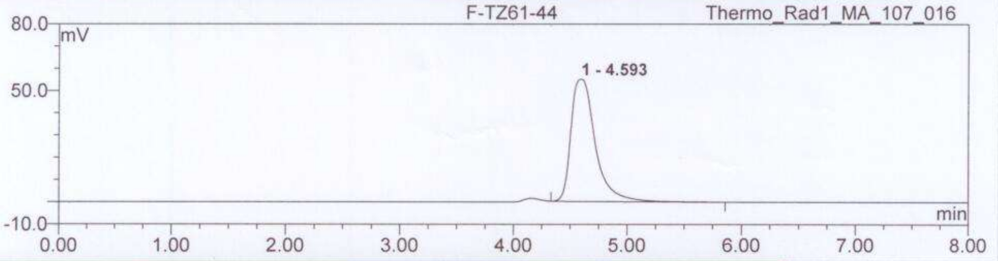

No.	Ret.Time min	Peak Name	Height mV	Area mV*min	Rel.Area %
1	4.59	n.a.	55.041	13.596	100.00
Total:			55.041	13.596	100.00

Reported by Initials / Date XY / 05/10/2017

Signoff by Signature / Date Yuyi Yue / 05/10/2017

F-TZ19-66B/Integration

Chromeleon (c) Dionex 1996-2001
Version 6.80 SR12 Build 3578 (207169)

TZ61-44 COINJ

Sample Name:	TZ61-84	*Column*	Agilent USCL0314814
Flow Rate(mL/min):	**1.00**		
Sample Type:	**standard**	*Mobile Phase:*	**70% ACN**
Control Program:	**F-TZ61-44**		**30% 0.1M Formate buffer pH=4.5**
Quantif. Method:	**F-TZ61-44**		
Recording Time:	**5/10/2017 14:15**		
Run Time (min):	**8.00**		

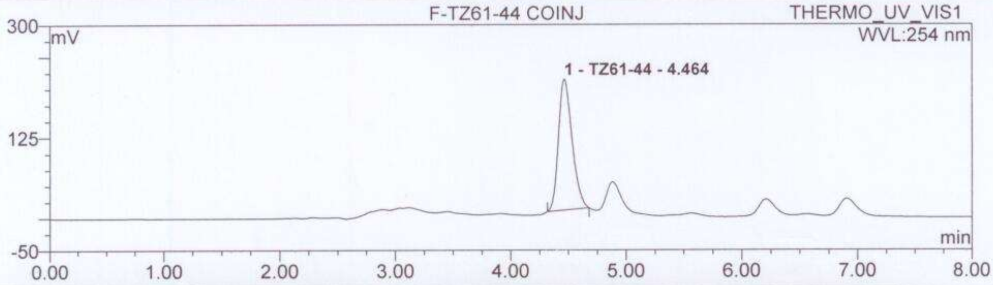

No.	Ret.Time min	Peak Name	Height mV	Area mV*min	Amount ug/mL	Rel.Area %
1	4.46	TZ61-44	202.891	27.86125	21.8467	100.00
Total:			202.891	27.861		100.00

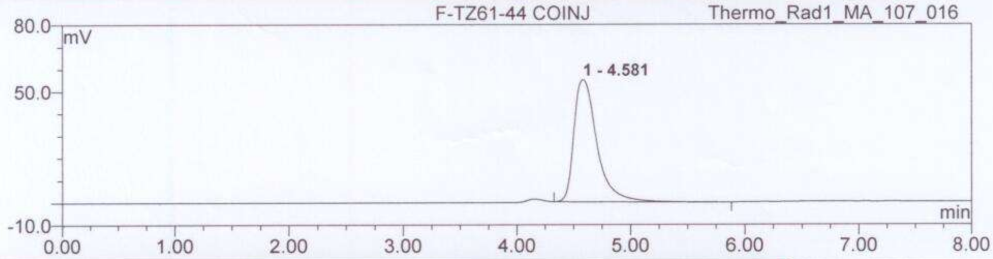

No.	Ret.Time min	Peak Name	Height mV	Area mV*min	Rel.Area %
1	4.58	n.a.	54.888	12.807	100.00
Total:			54.888	12.807	100.00

Reported by Initials / Date XY / 05/10/2017

Signoff by Signature / Date Xiyi Yue / 05/10/2017

F-TZ19-66B/Integration

Chromeleon (c) Dionex 1996-2001
Version 6.80 SR12 Build 3578 (207169)

Radionuclidic Identity Analysis on 5/10/2017 at 14:13

mCi Reading	Time(min)
0.696	0.497
0.694	1.008
0.691	1.519
0.688	2.029
0.687	2.540
0.685	3.051
0.683	3.562
0.680	4.073
0.679	4.584
0.676	5.095
0.674	5.605
0.672	6.116
0.670	6.626
0.668	7.137
0.665	7.648
0.663	8.159
0.661	8.669
0.659	9.180
0.656	9.691
0.654	10.202

**Calculated Halflife(sec)**	6580.902
**Theoretical Halflife(sec)**	6586.200
**Calculated Halflife(min)**	109.682
**Theoretical Halflife(min)**	109.770
**Percent Difference**	0.080

18F-TZ61-44

Batch ID 18F-TZ61-44-05101

Initials XY

Date 5/10/2017

Sign Off Full Name: Xuyi Yue

Sign Off Date: 05/10/2017

End of Decay: 14:03

Decay performed on: CRC712MH

Radiosynthesis of [^{125}I]8i ([^{125}I]TZ61-68)

To a 2 mL low-retention centrifuge tube was added 50 µL tributylstannane precursor (4 mg/mL) and 50 µL freshly prepared 5% sodium acetate in glacial acetic acid. 10 µL of Na[^{125}I]I was added to the vial. After the addition of 50 µL of freshly prepared hydrogen peroxide/acetic acid, the mixture was stirring at room temperature for 15 mins and vortexed for 3 times. Reaction mixture was loaded onto a semi-preparative HPLC system. The retention time for the target compound was 17 min. The collected HPLC fraction was diluted with 50 mL of sterile water, the product was trapped on a C-18 Sep-Pak Plus cartridge and washed with 20 mL of water. Before the C-18 Sep-Pak was washed with ethanol. The purity was determined to be 95% by radio TLC (Hexane/Ethyl Acetate: 2/1).

Cold TZ61-84

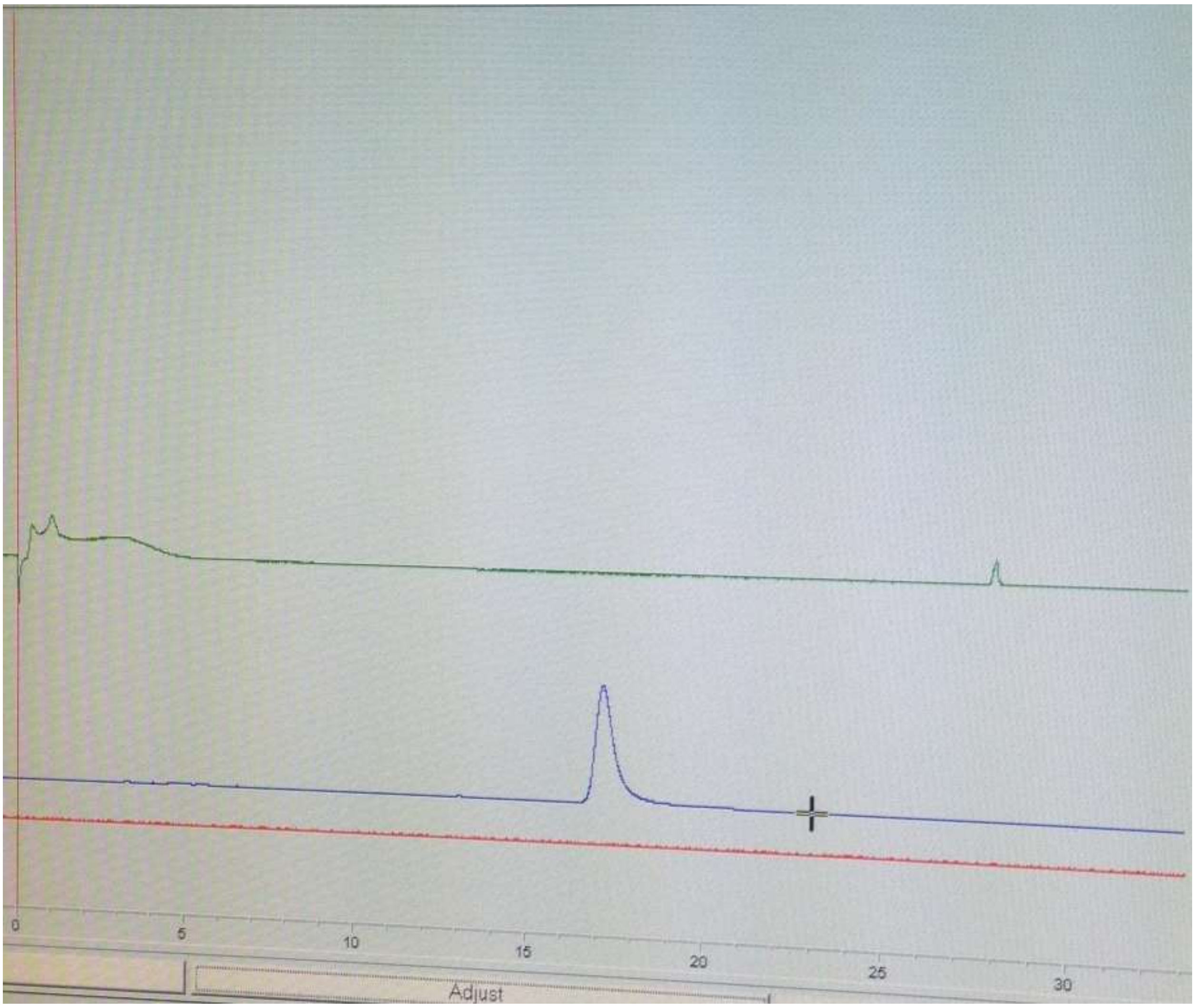

Hot ^{125}I-TZ61-84

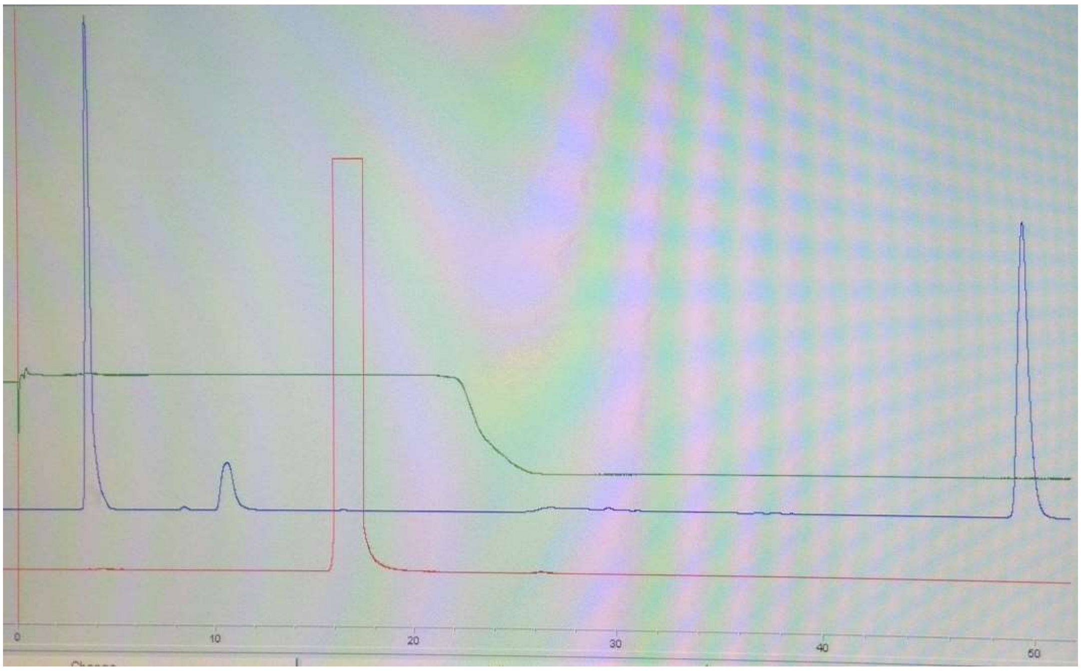

^{125}I-TZ61-84 and TZ61-84 co-injection

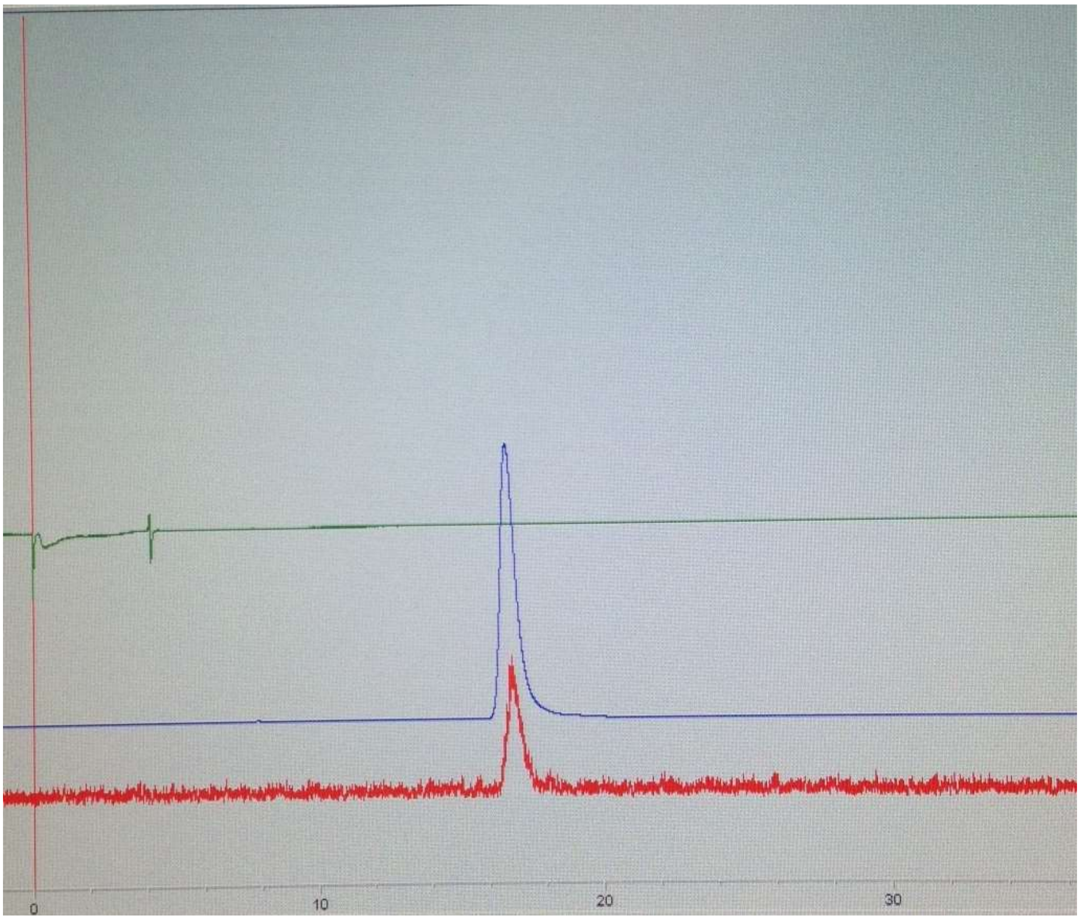

8. PET brain imaging studies in nonhuman primates

All animal experiments were conducted by following the Guidelines for the Care and Use of Research Animals under a research protocol approved by the Washington University Institutional Animal Care and Use Committee (IACUC). The NHP study was conducted in the NHP microPET facility at the Washington University School of Medicine in St. Louis. Three adult male cynomolgus macaques (9.73 ± 0.71 kg) were used in this study. The animal was fasted for 12 h prior to PET scans. Anesthesia was induced with an intramuscular injection of ketamine (10–20 mg kg^{-1}) and glycopyrrolate (0.013–0.017 mg kg^{-1}) to reduce salivary gland and respiratory secretions. The animal was intubated, and anesthesia was maintained at 40-50% N_2O and 1.4–2.0% isoflurane/oxygen throughout the procedure. A percutaneous venous catheter was placed for radiotracer injection. During the PET scanning session, the head was positioned supine in an adjustable head holder with the brain in the center of the field of view. PET scans were performed with a microPET Focus 220 scanner (Concorde/CTI/Siemens Microsystems, Knoxville, TN, USA). A 10 min transmission scan was performed to check positioning; once confirmed, a 45 min transmission scan was obtained for attenuation correction. Subsequently, a 2 h dynamic emission scan was acquired after administration of 365.44 ± 26.69 MBq of [^{11}C]TZ61-84, [^{11}C]TZ55-107 or [^{18}F]TZ61-44 *via* the venous catheter. PET data were collected from 0–120 min with the following time frames: 3 × 1 min, 4 × 2 min, 3 × 3 min and 20 × 5 min. The PET images were reconstructed using a filtered back projection method into a volume size of 128 × 128 × 95 and a voxel size of 1.85 × 1.85 × 0.796 mm^3, with all corrections (scatter, decay, randoms, normalizations, etc.) applied. The dynamic PET images were motion-corrected and then co-registered to their structural MPRAGE MR images. The INIA19 primate brain and atlas (https://www.nitrc.org/projects/inia19/) were then co-registered to the MR images and to obtain the regional time−activity curves (TACs) in the regions of interests (ROIs). The radioactivity uptake was normalized using the body weight and the injected dose to obtain the standardized uptake values (SUVs). Voxel-wise reference Logan was performed using cerebellum as the reference region to obtain the distribution volume ratio (DVR). Image processing was performed using MIAKAT in Matlab (R2023a, The MathWorks, Inc.).

(MR data were acquired prior to PET image acquisition with a Siemens 3T Trio scanner, with an extremity head coil in the coronal direction and the following spin echo sequence (TE = 3.34 ms, TR = 2530 ms, fip angle = 7°, thickness = 0.50 mm, feld-of-view = 140 mm, image matrix = 256 × 256 × 176, voxel size = 0.547 × 0.547 × 0.500 mm).)

9. Copies of ^{1}H NMR, and ^{13}C NMR Spectra

_N_-(6-methoxypyridin-3-yl)quinolin-2-amine (3a, TZ80-157).

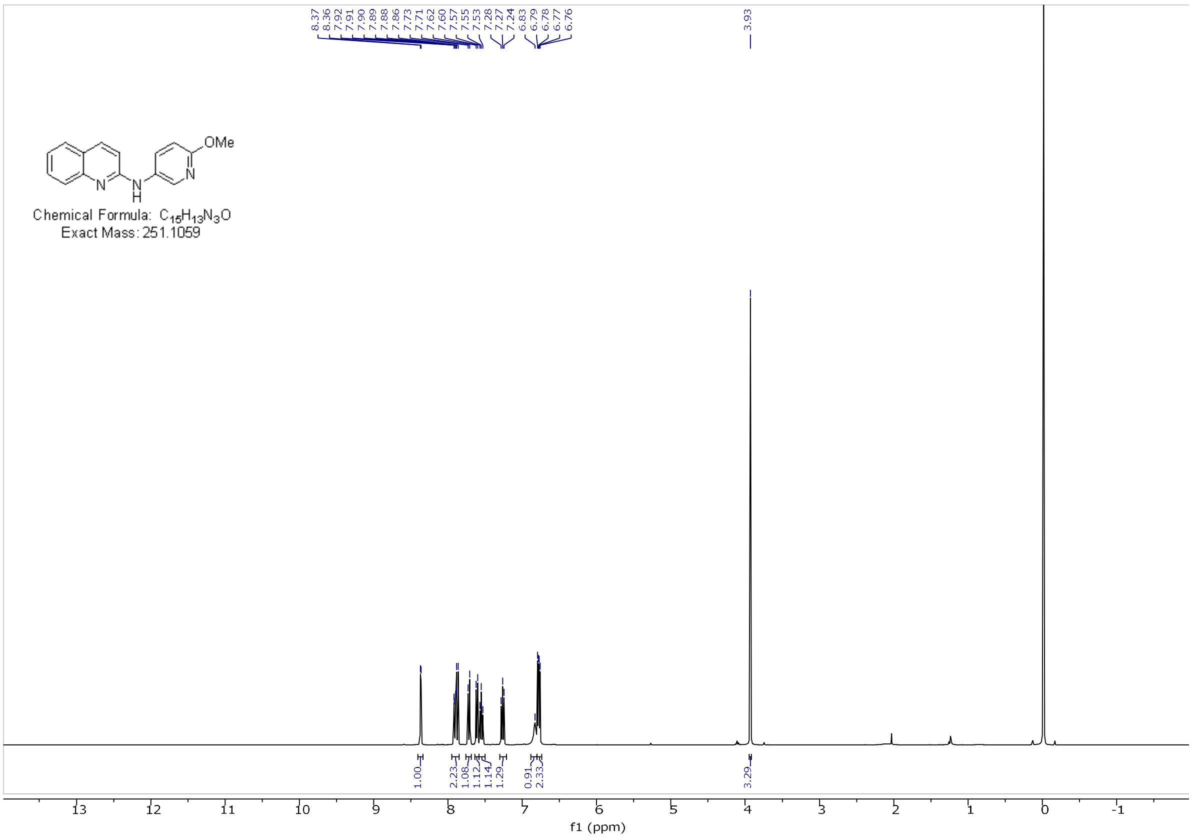

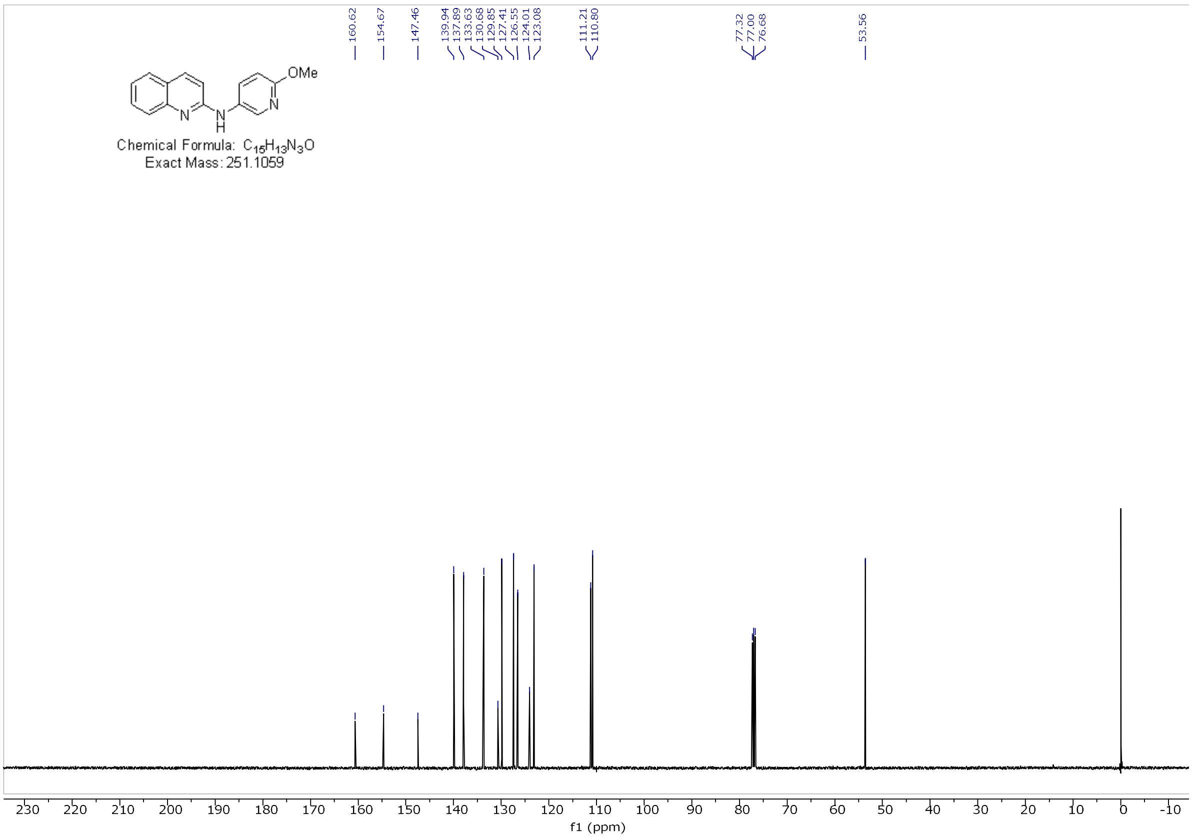

***N*-(6-methoxypyridin-3-yl)isoquinolin-3-amine (3b, TZ90-14).**

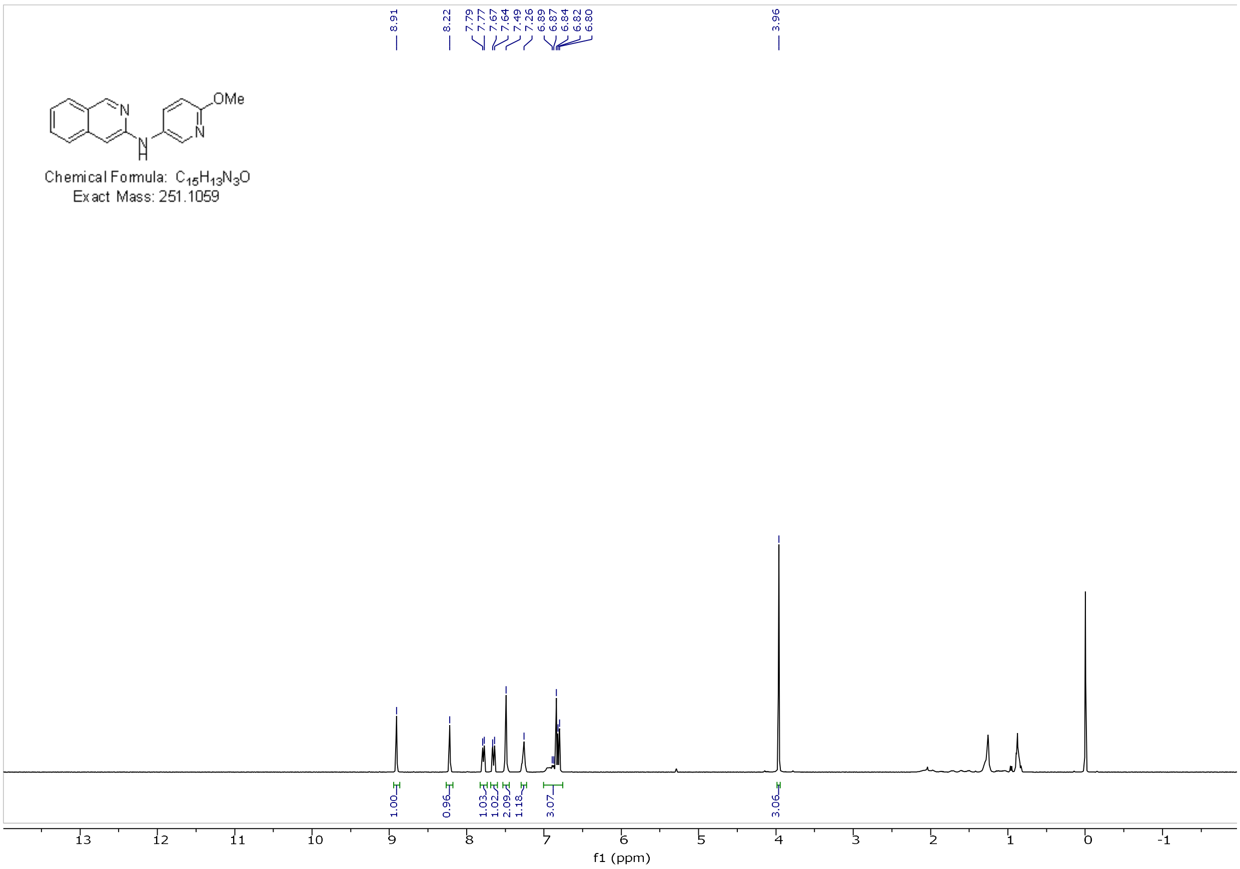

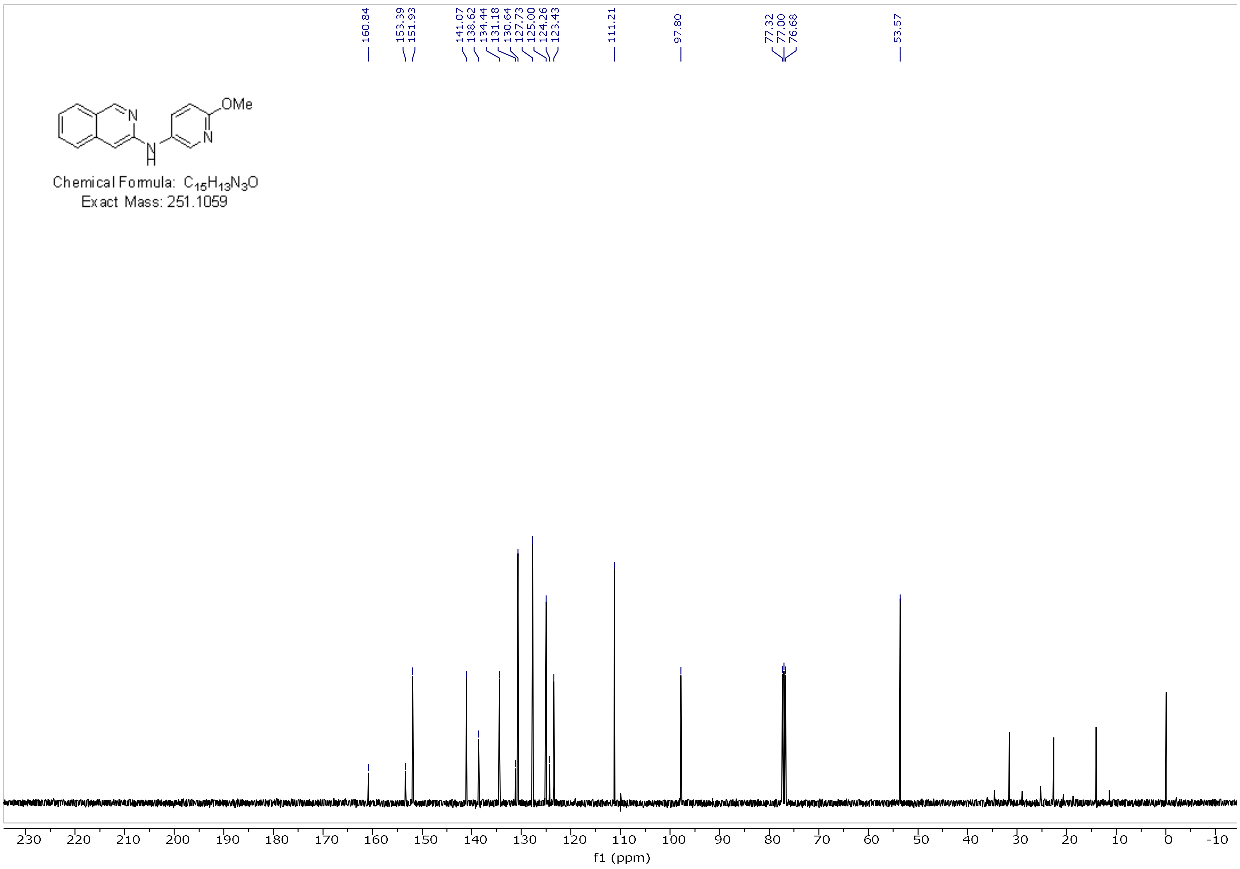

***N*-(6-methoxypyridin-3-yl)quinolin-3-amine (3c, TZ90-6).**

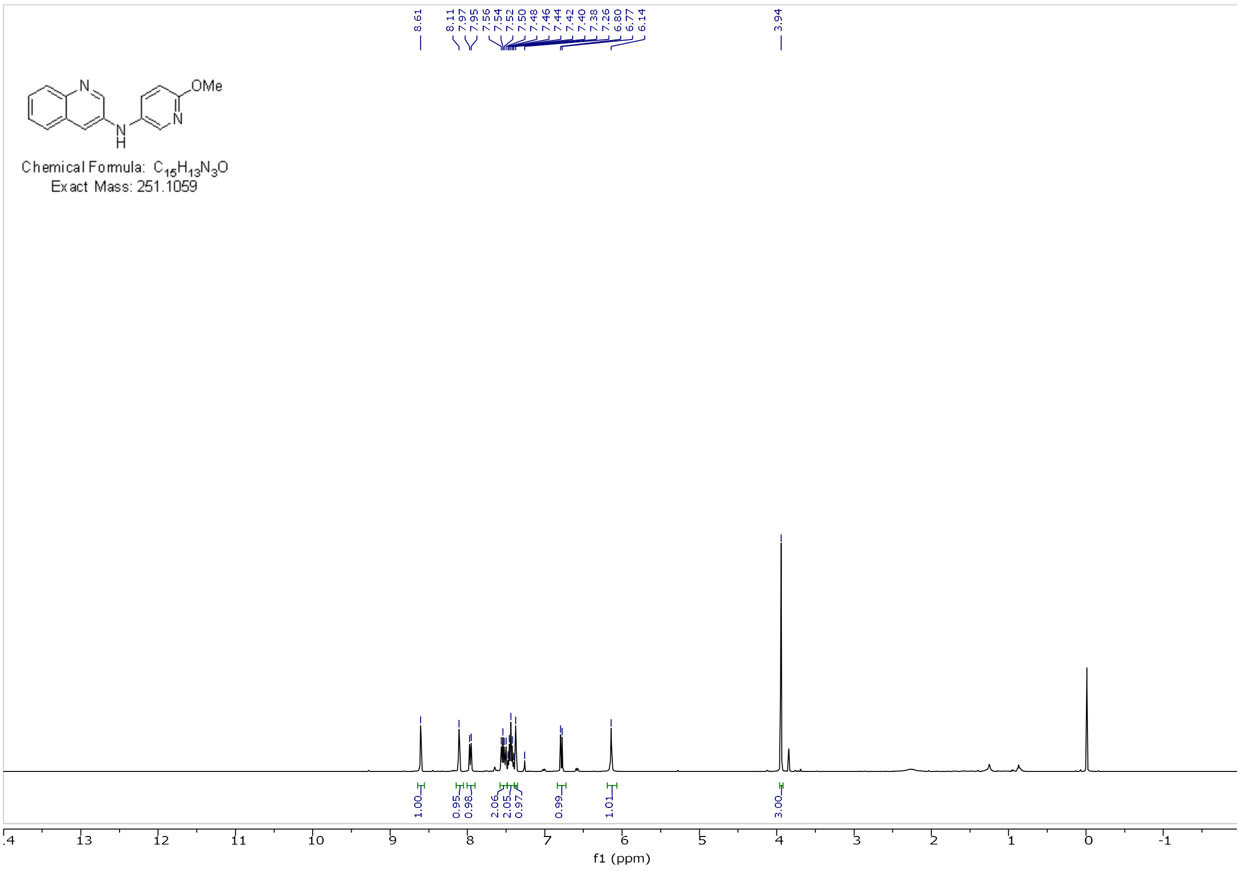

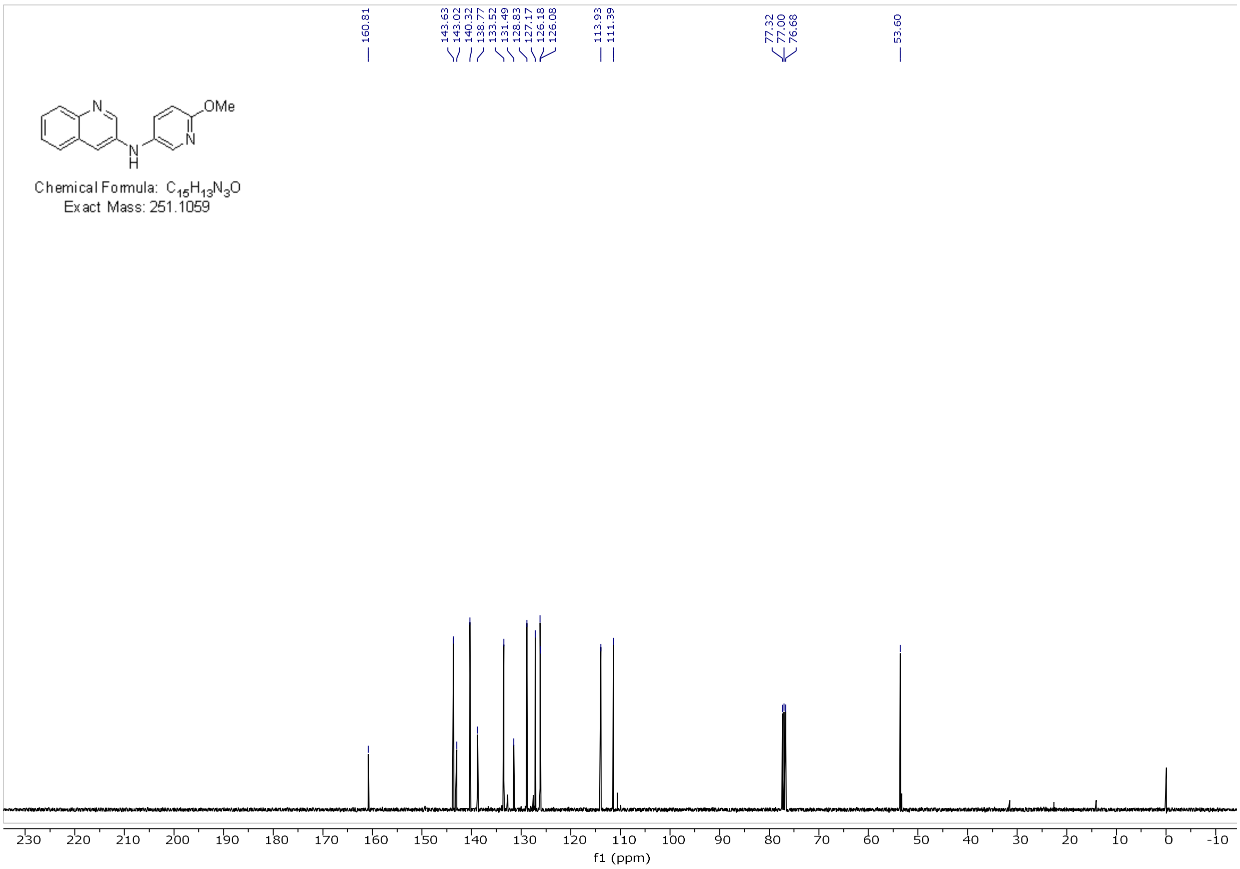

N-(6-Methoxypyridin-3-yl)quinolin-6-amine (3d, TZ64-29).

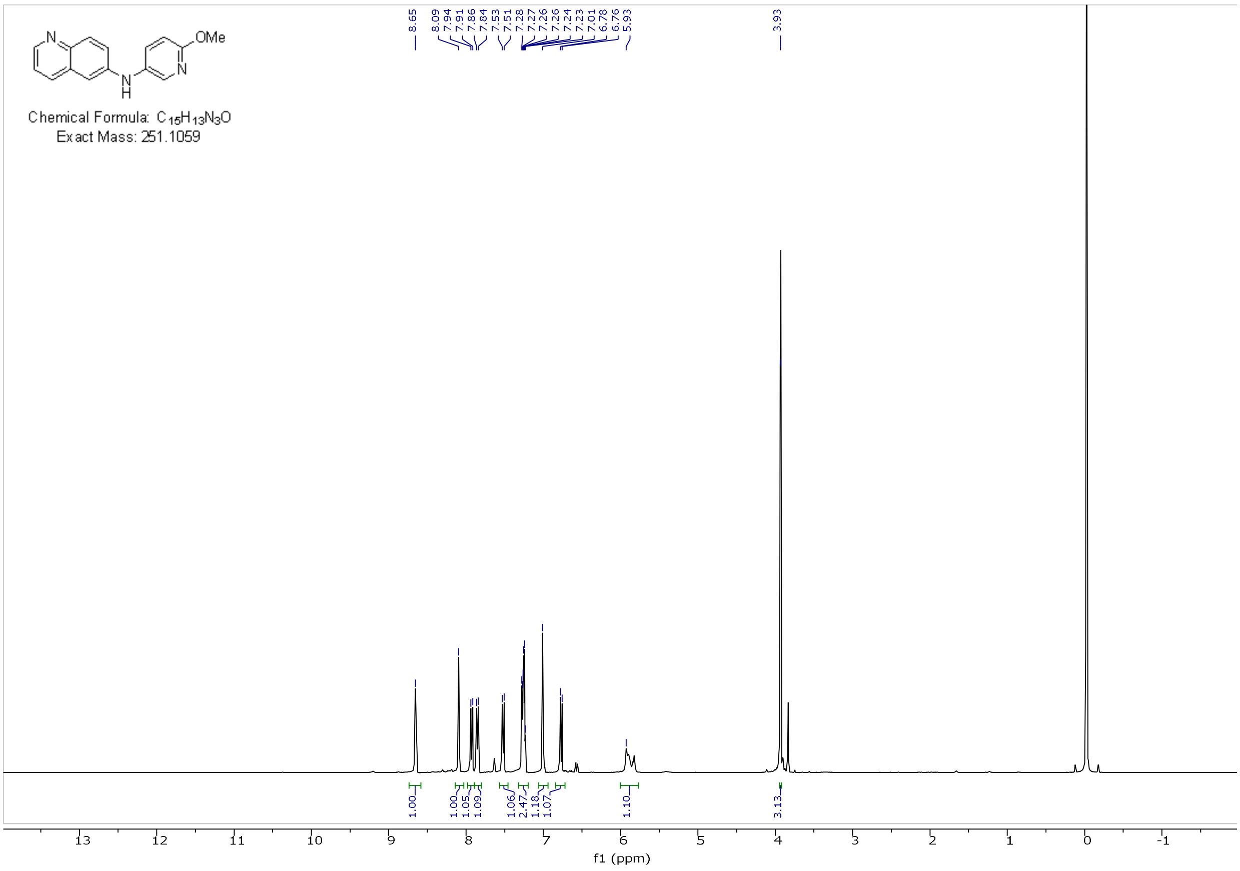

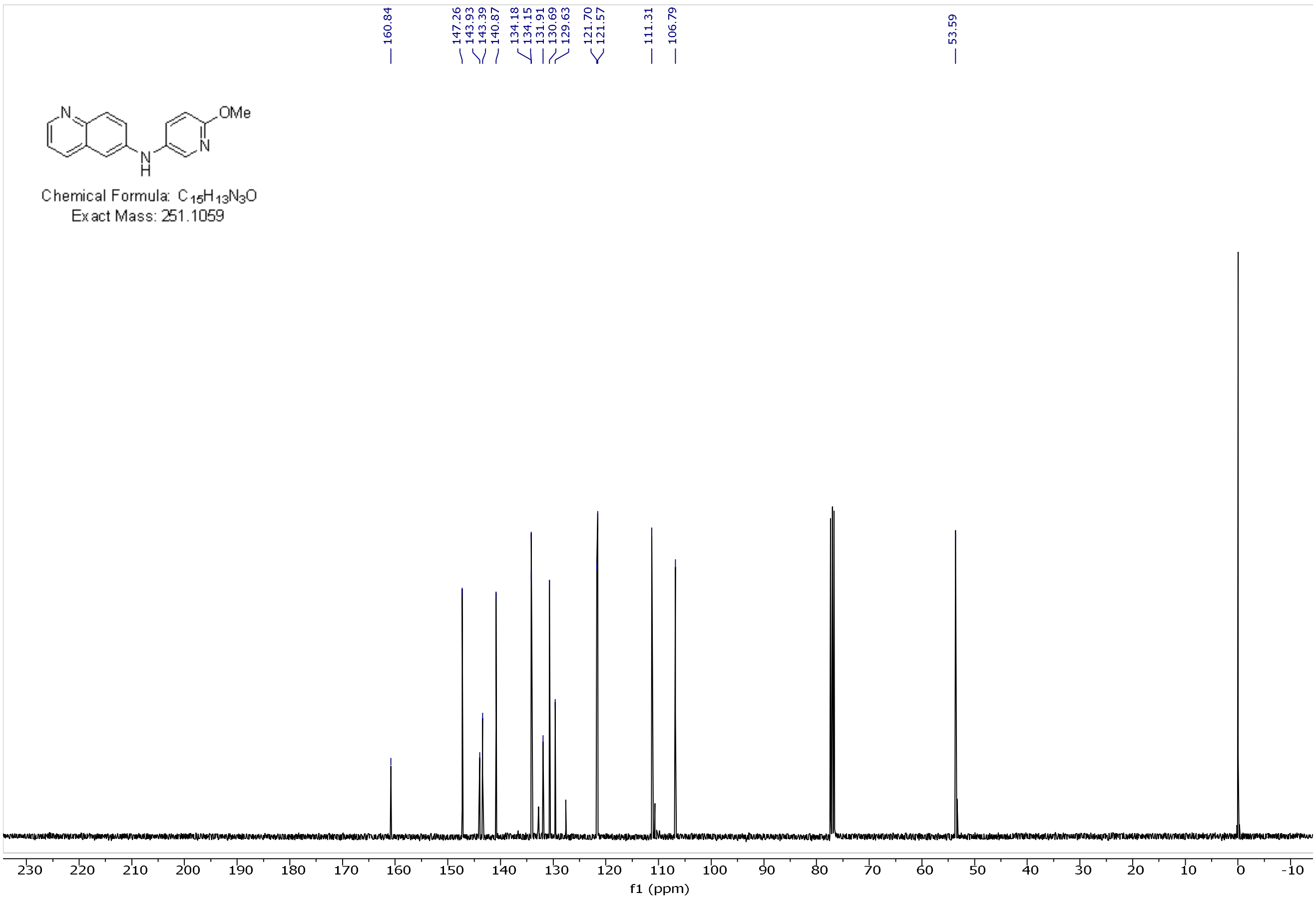

N-(6-Methoxypyridin-3-yl)isoquinolin-6-amine (3e, TZ64-27).

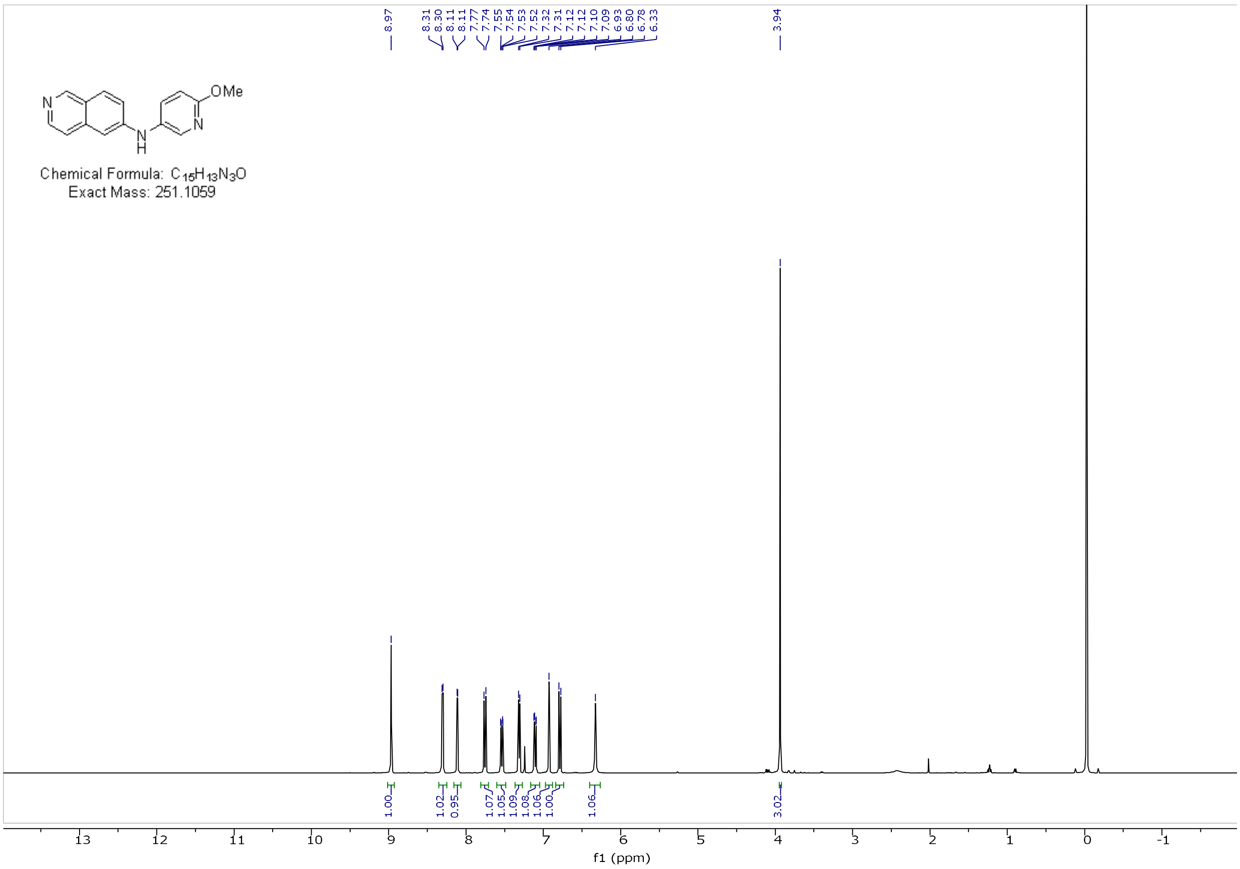

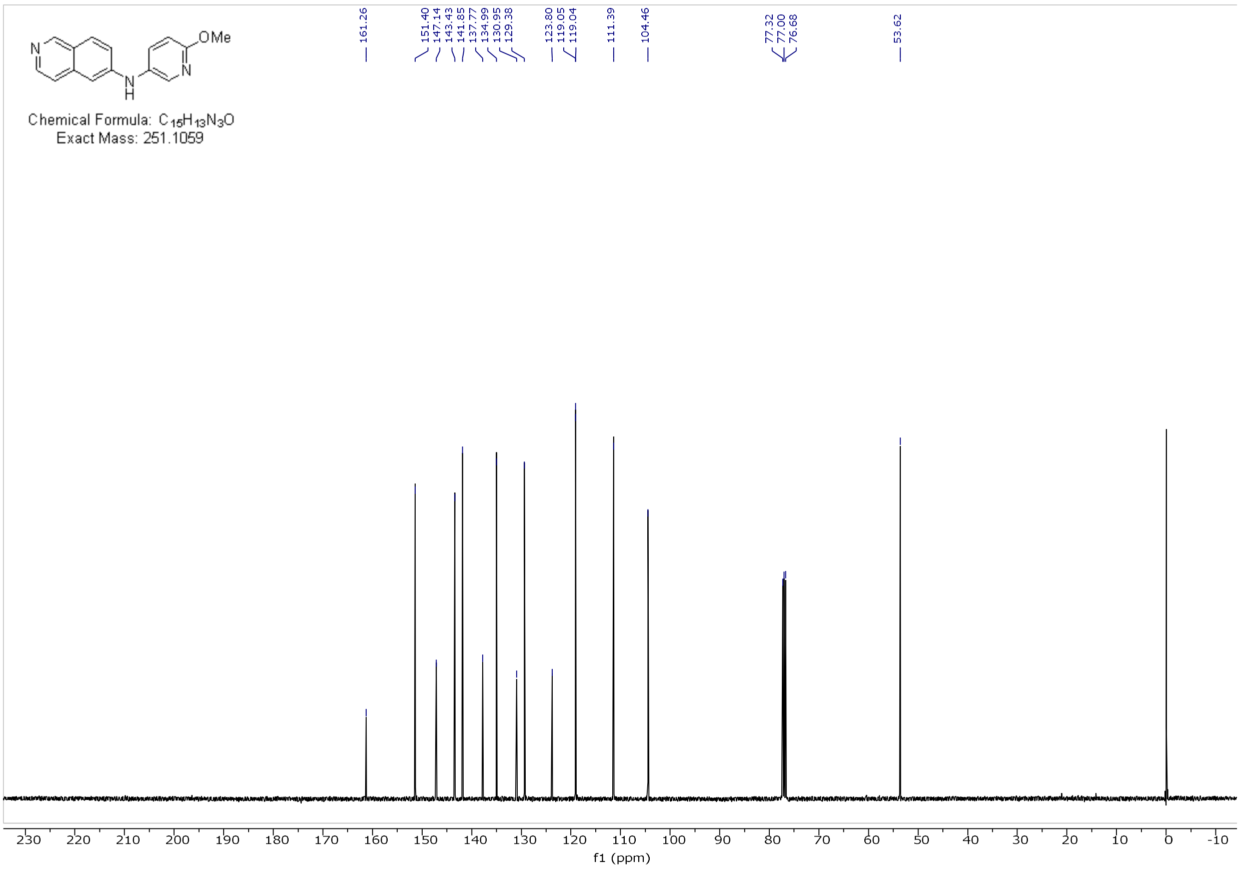

N-(6-Methoxypyridin-3-yl)isoquinolin-7-amine (3f, TZ90-15).

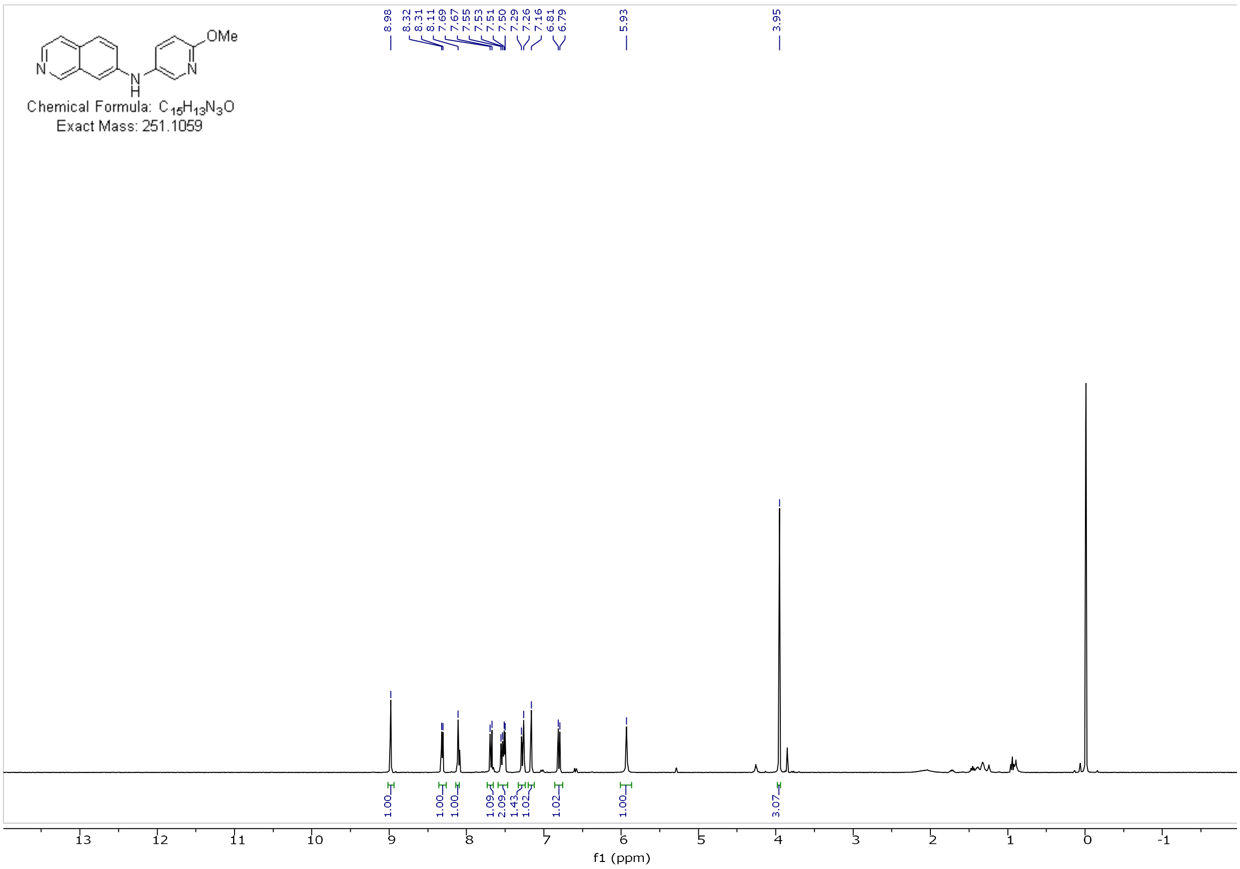

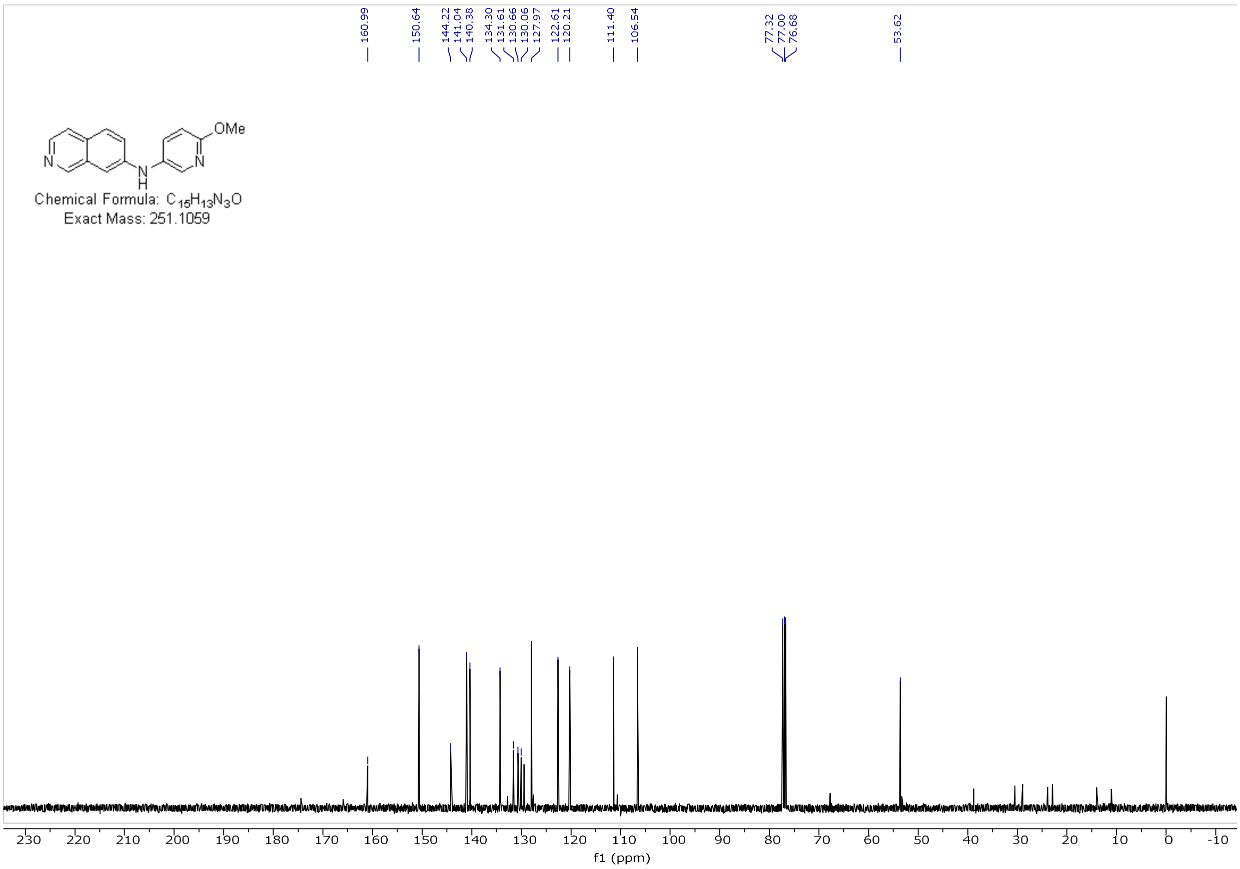

N-(6-Methoxypyridin-3-yl)quinolin-7-amine (3g, TZ64-33).

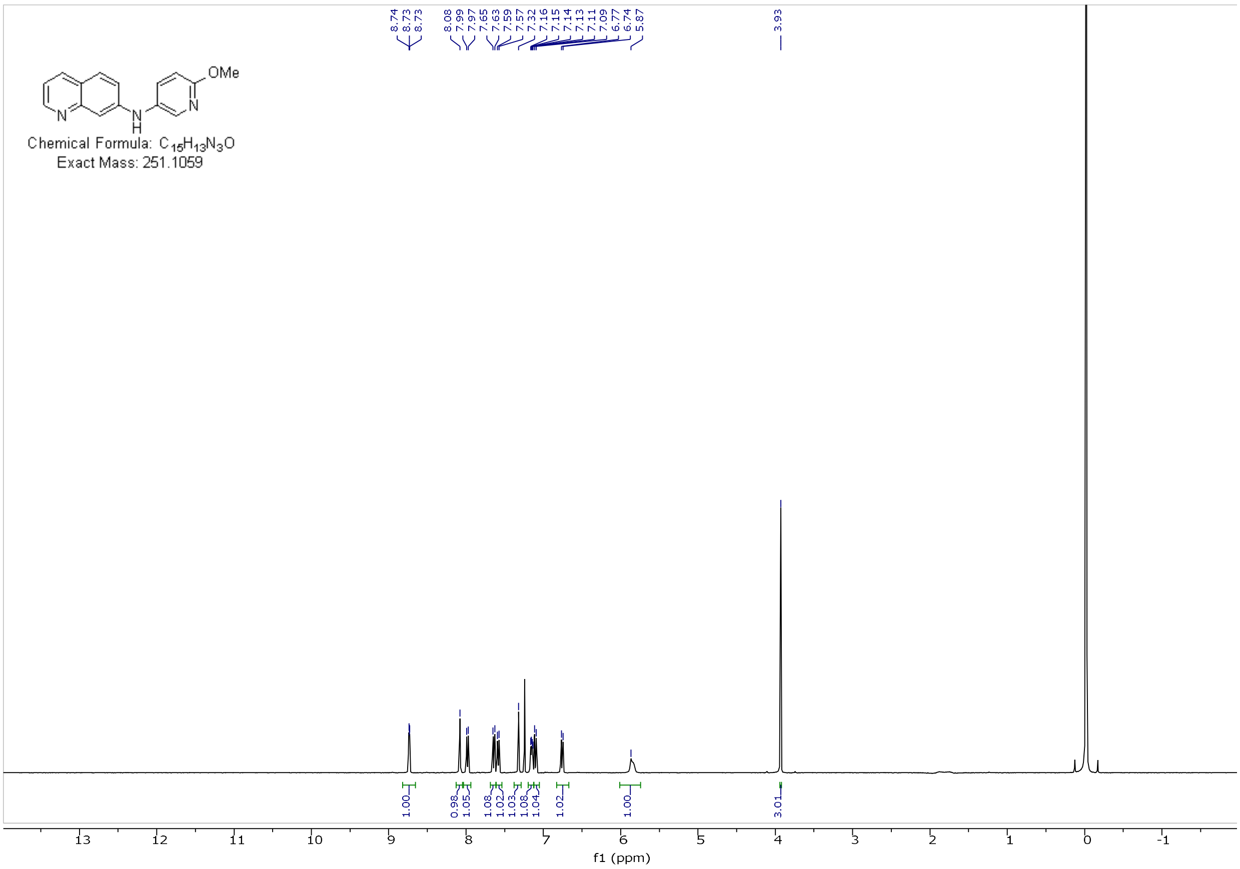

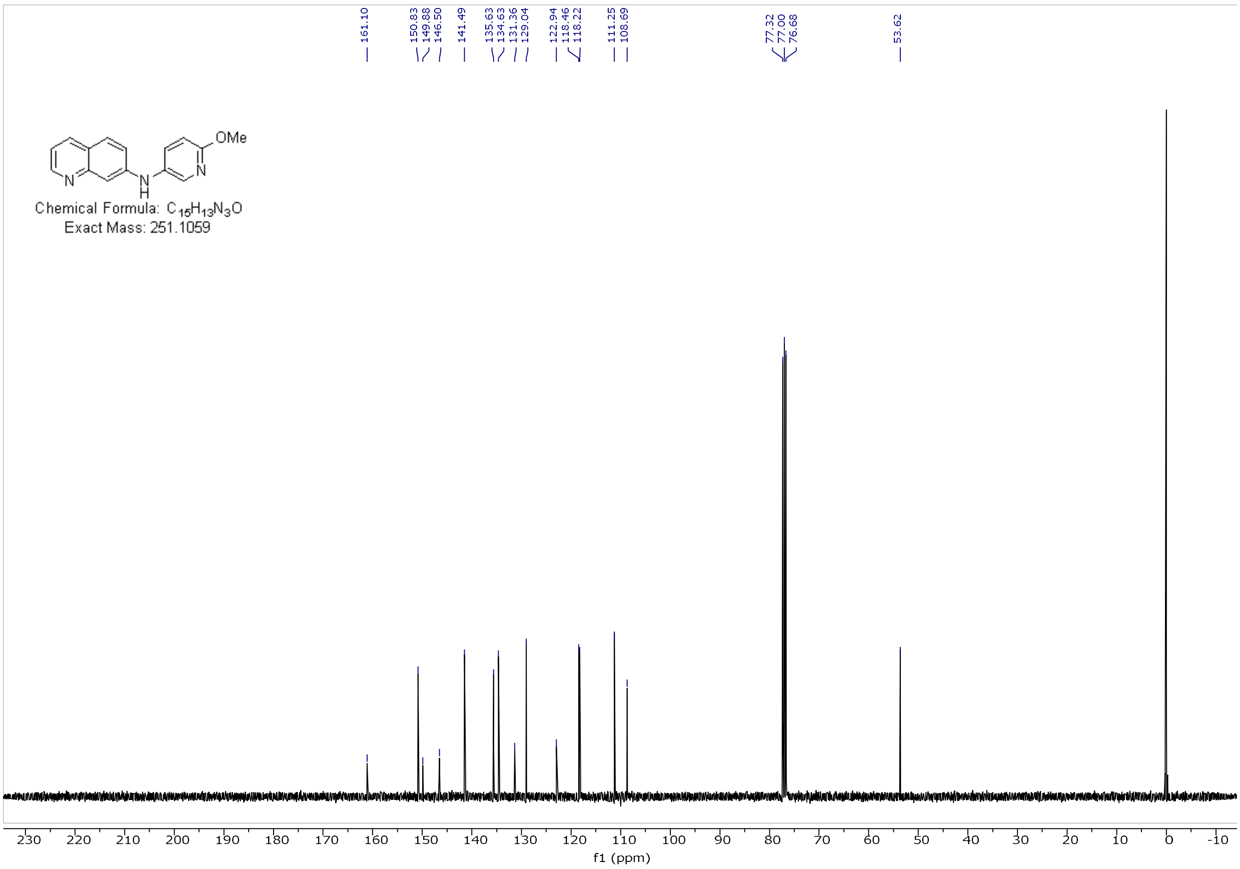

3-Fluoro-*N*-(6-methoxypyridin-3-yl)quinolin-2-amine (5a, TZ64-14).

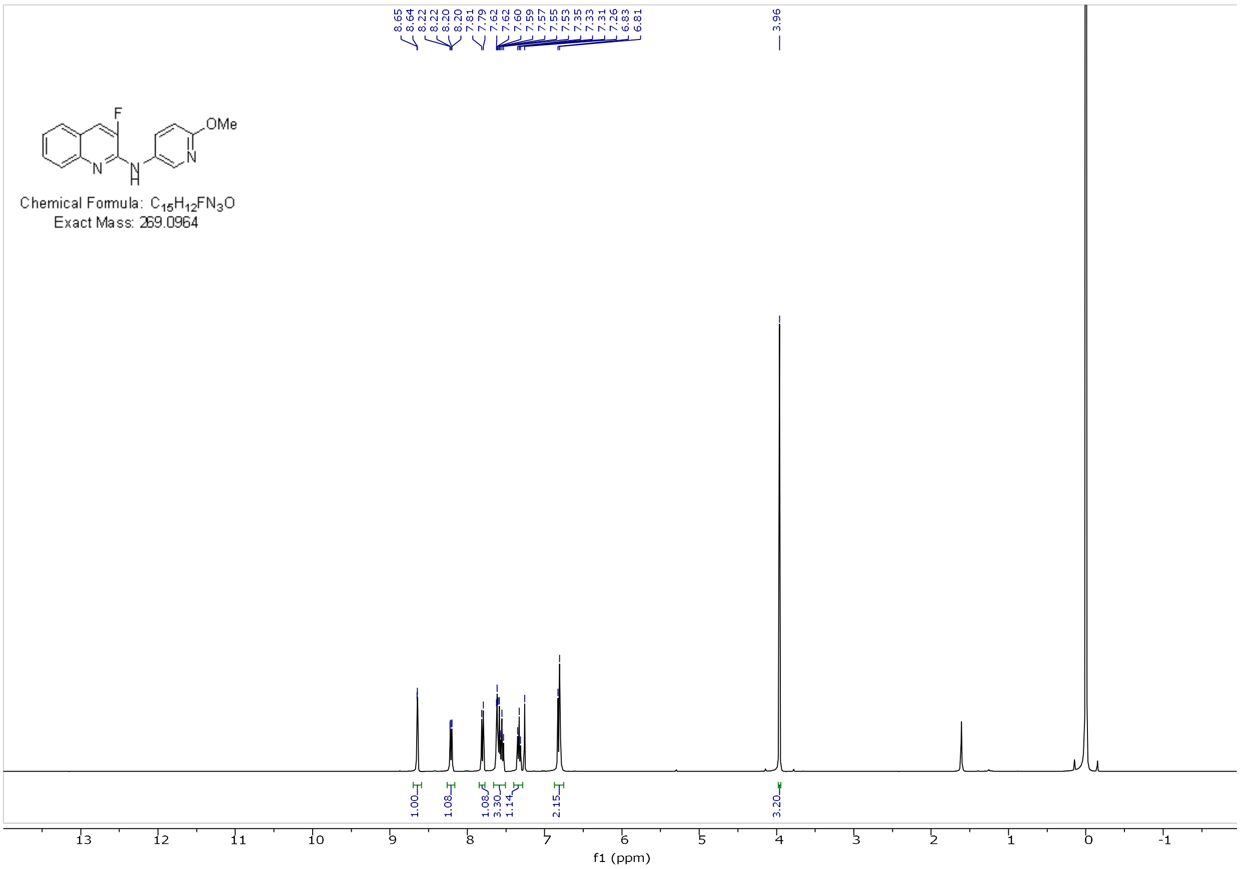

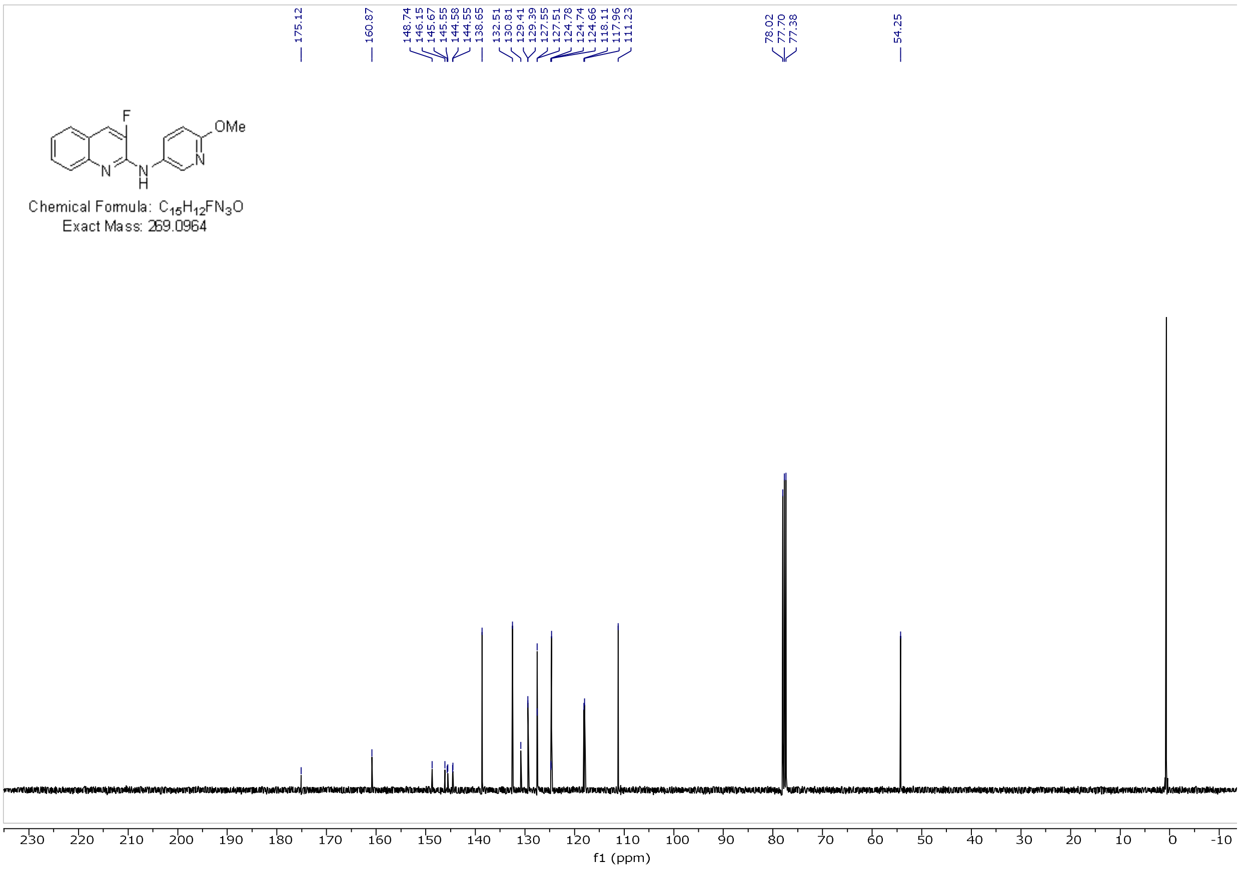

4-Fluoro-*N*-(6-methoxypyridin-3-yl)quinolin-2-amine (5b, TZ64-66).

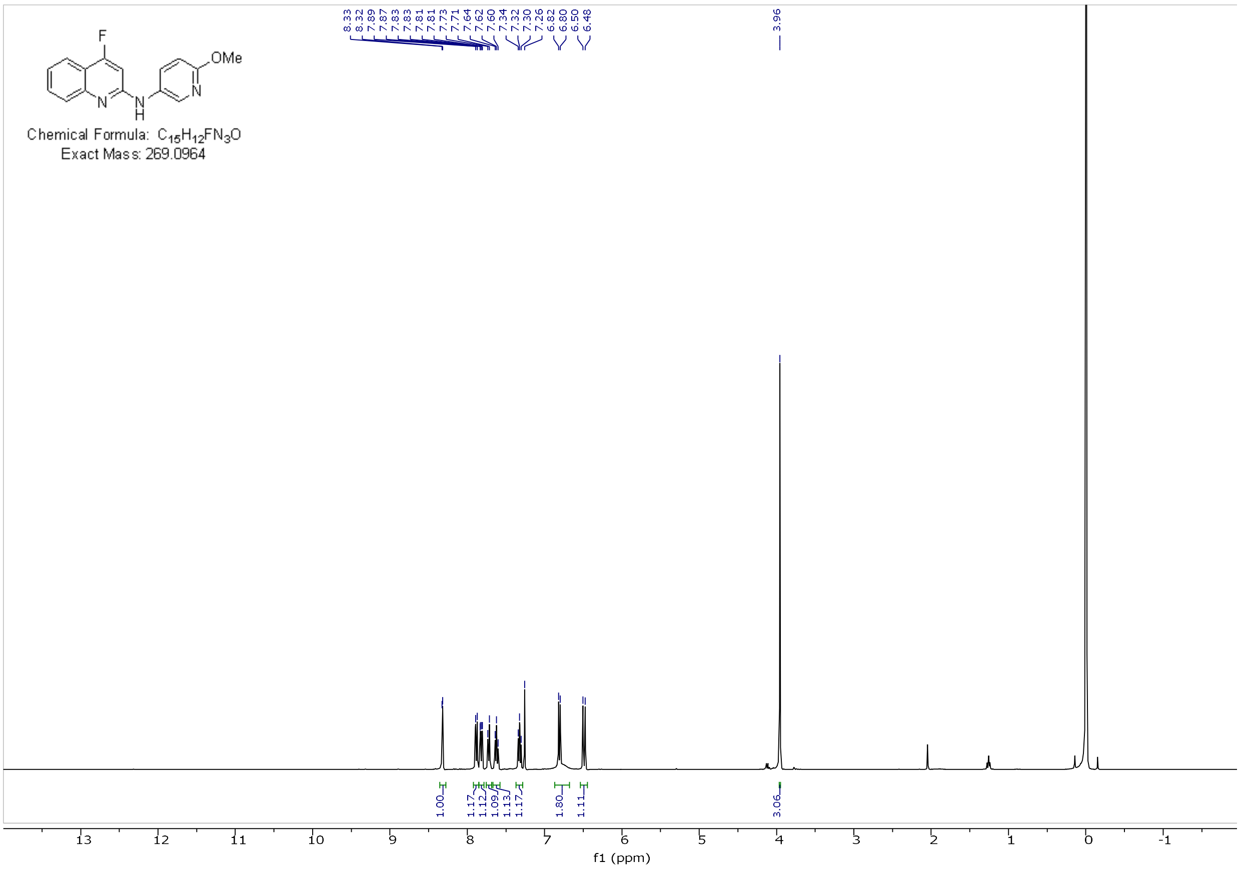

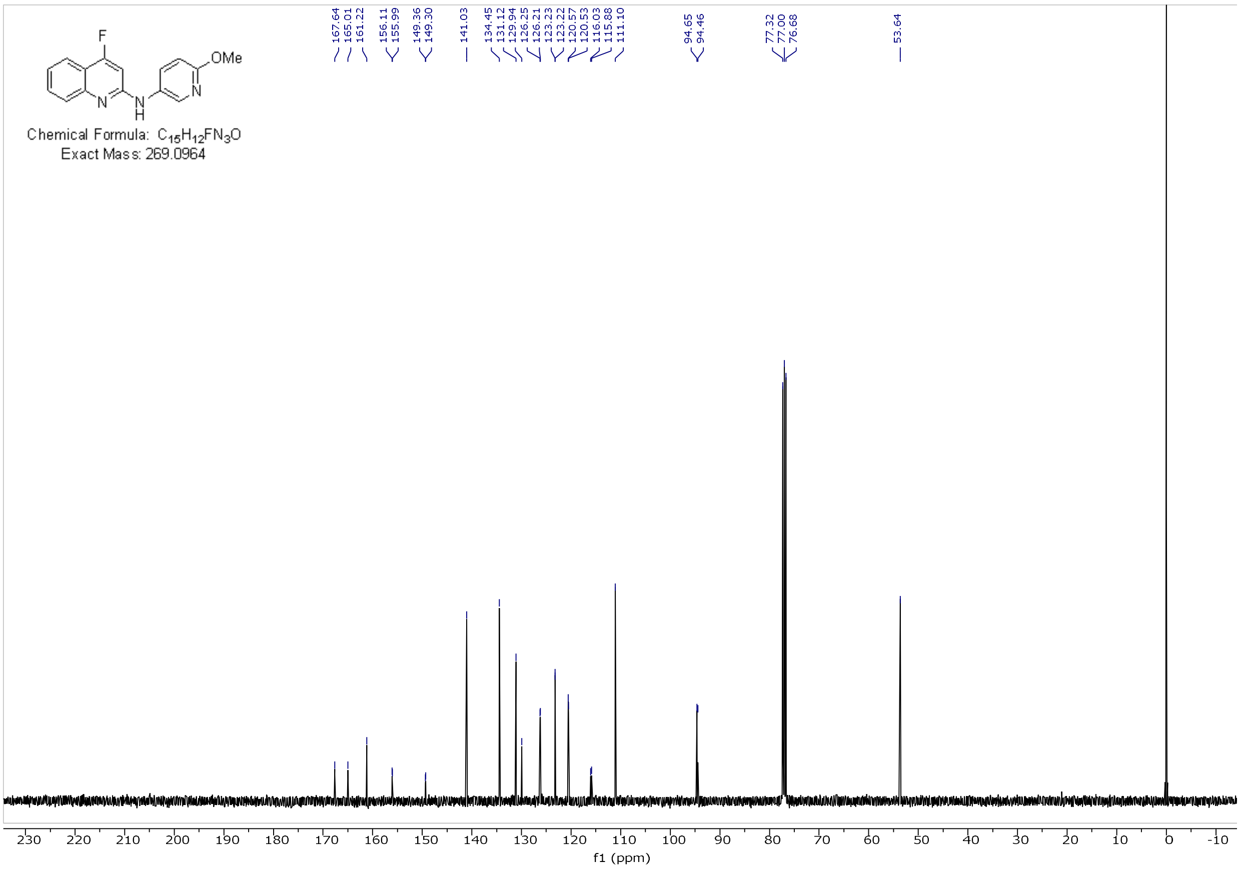

5-Fluoro-*N*-(6-methoxypyridin-3-yl)quinolin-2-amine (5c, TZ64-15).

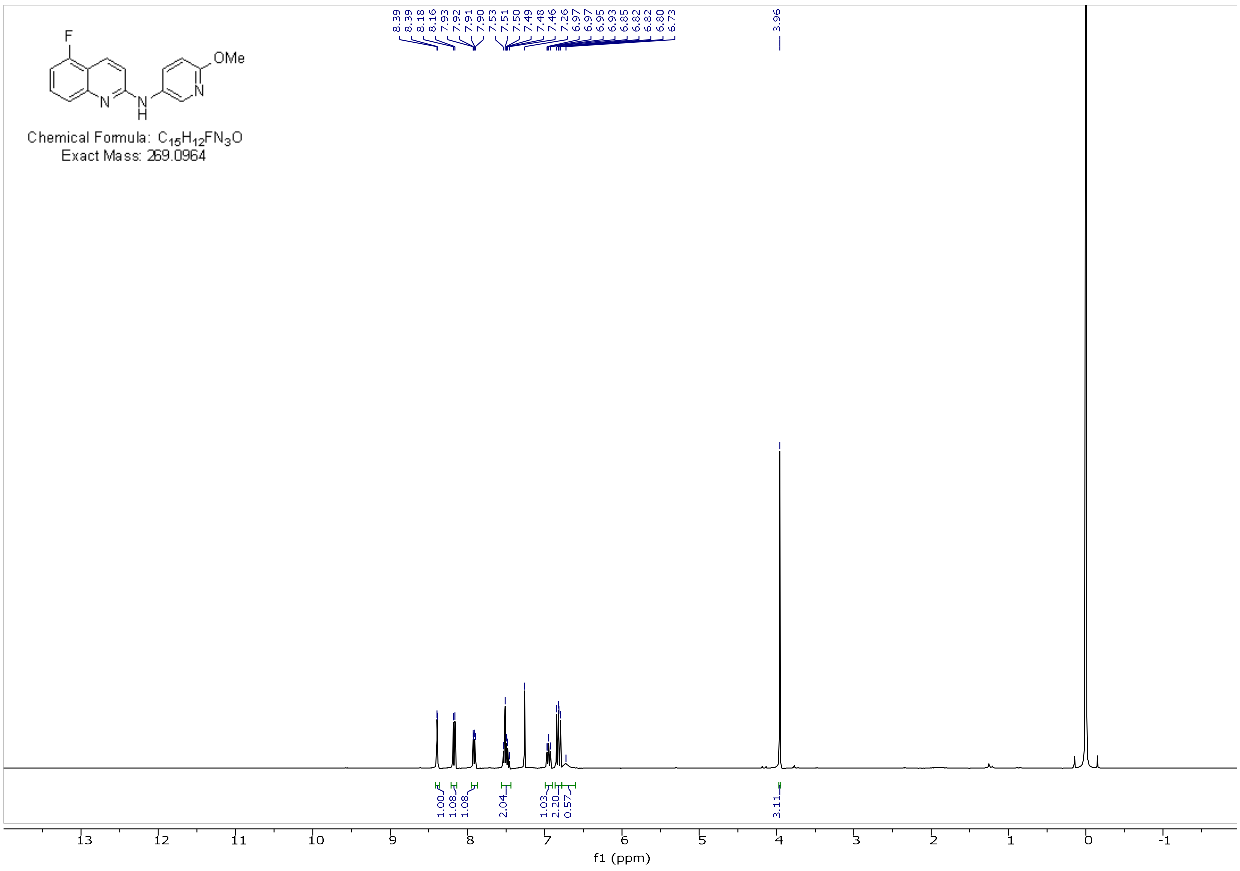

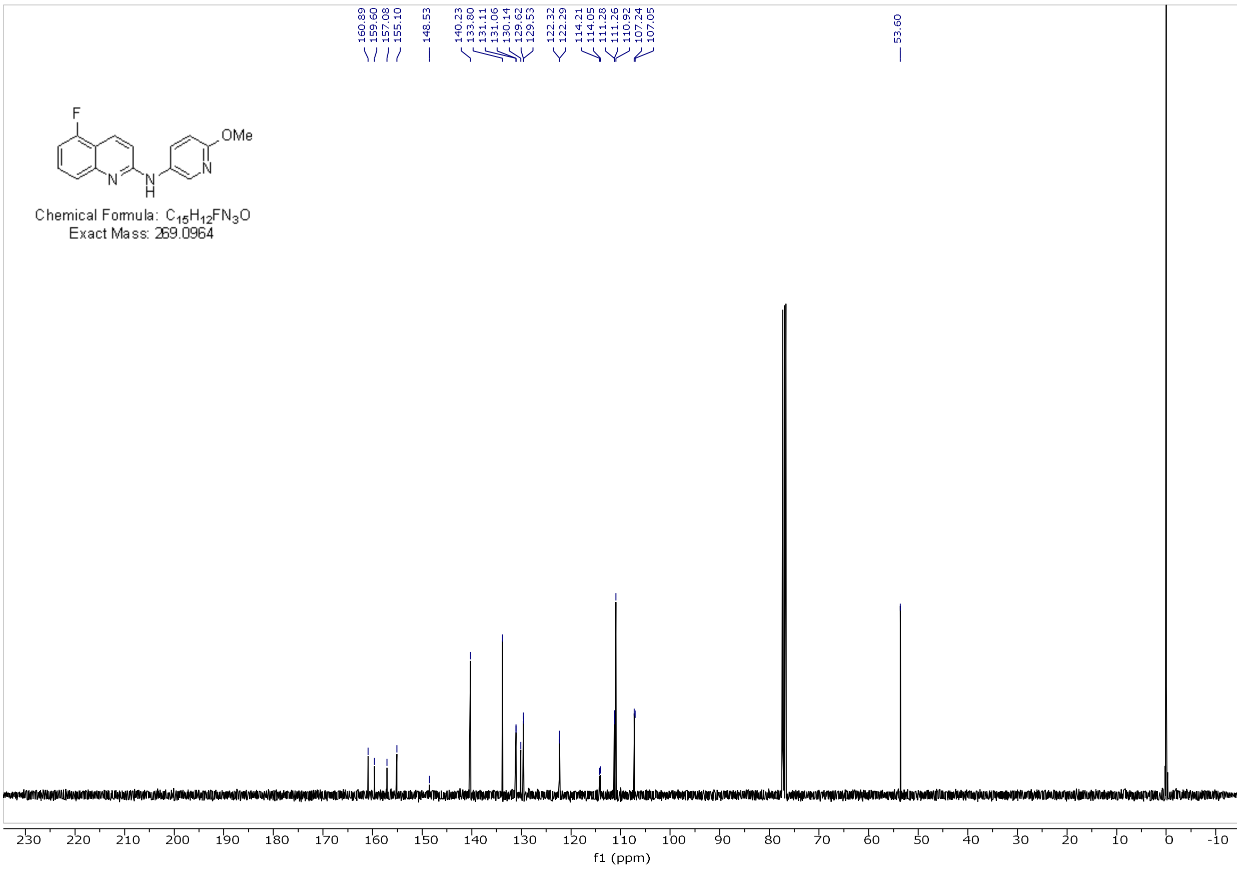

6-Fluoro-*N*-(6-methoxypyridin-3-yl)quinolin-2-amine (5d, TZ64-13).

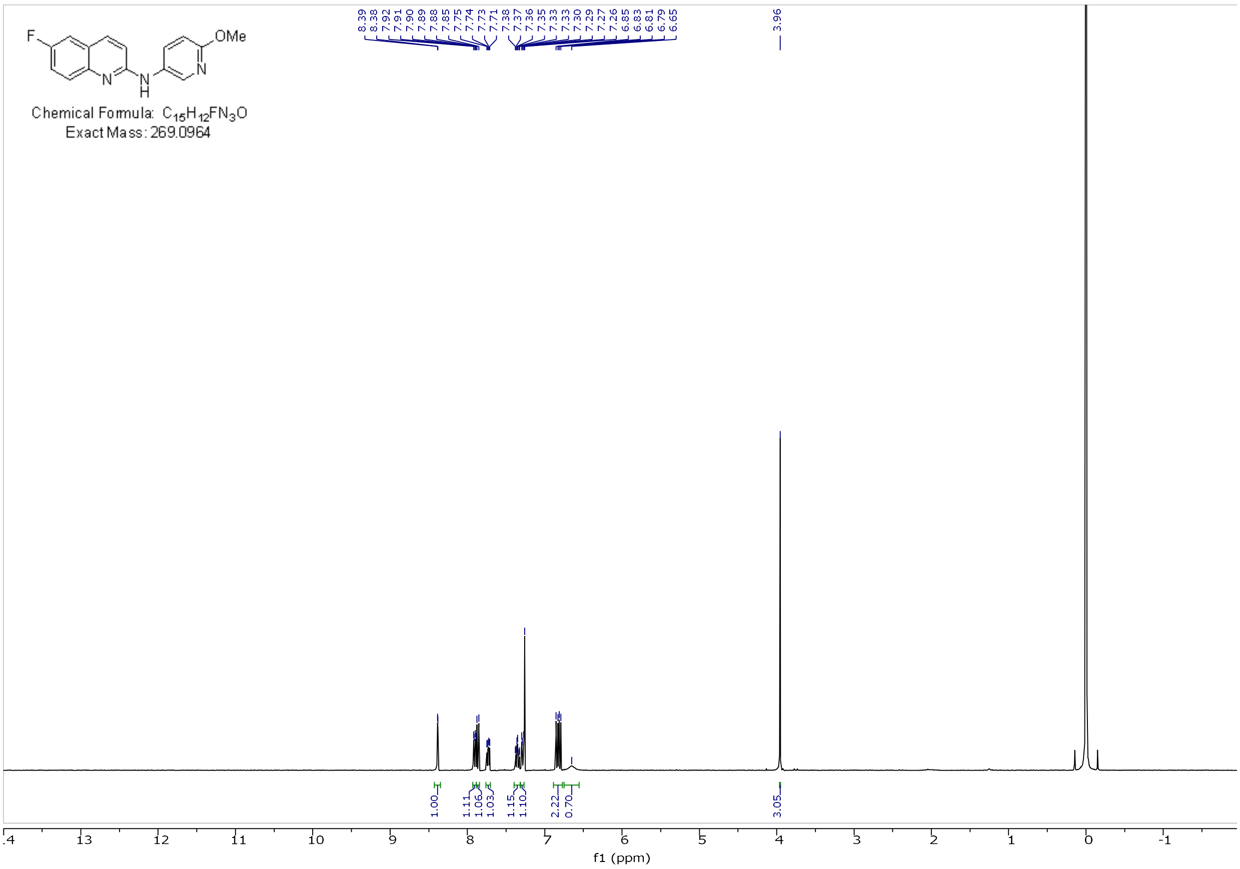

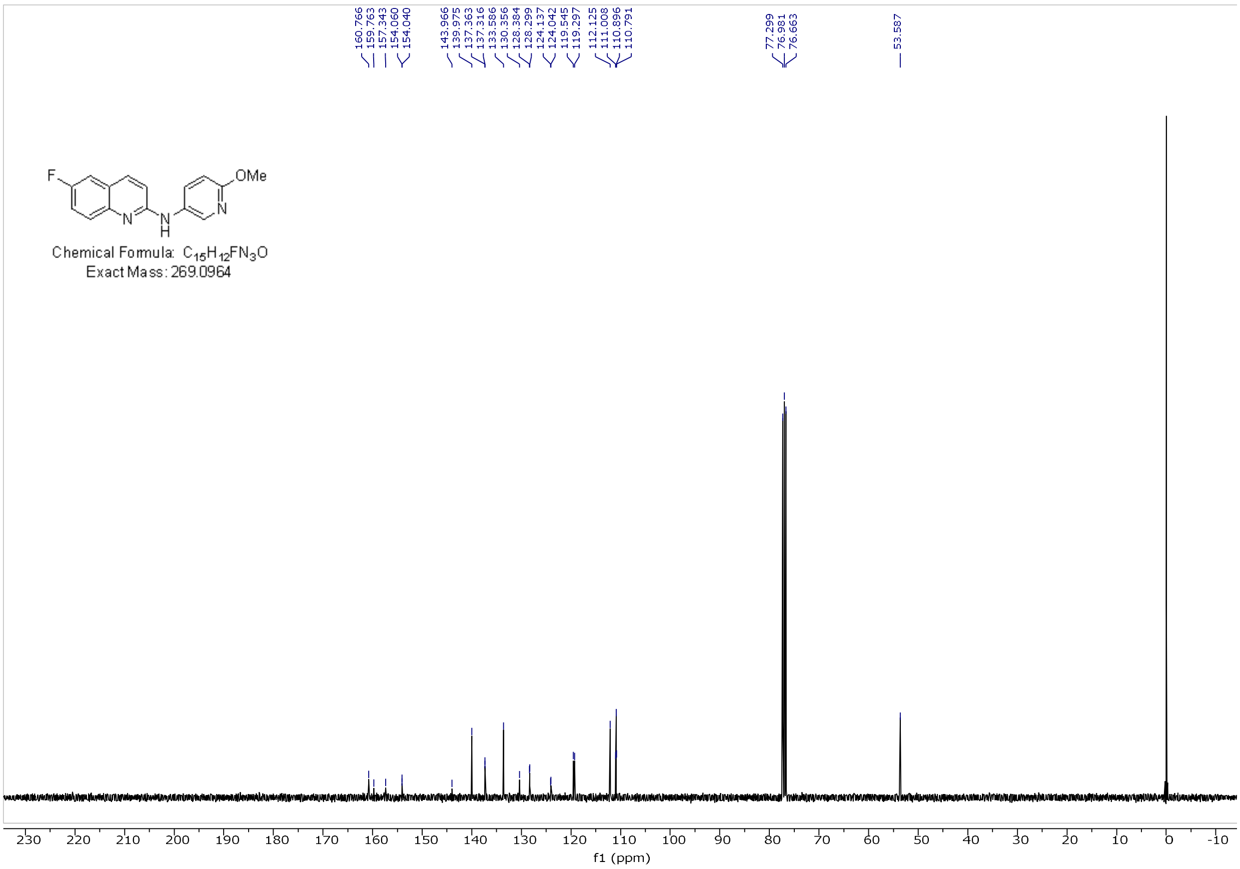

7-Fluoro-*N*-(6-methoxypyridin-3-yl)quinolin-2-amine (5e, TZ64-68).

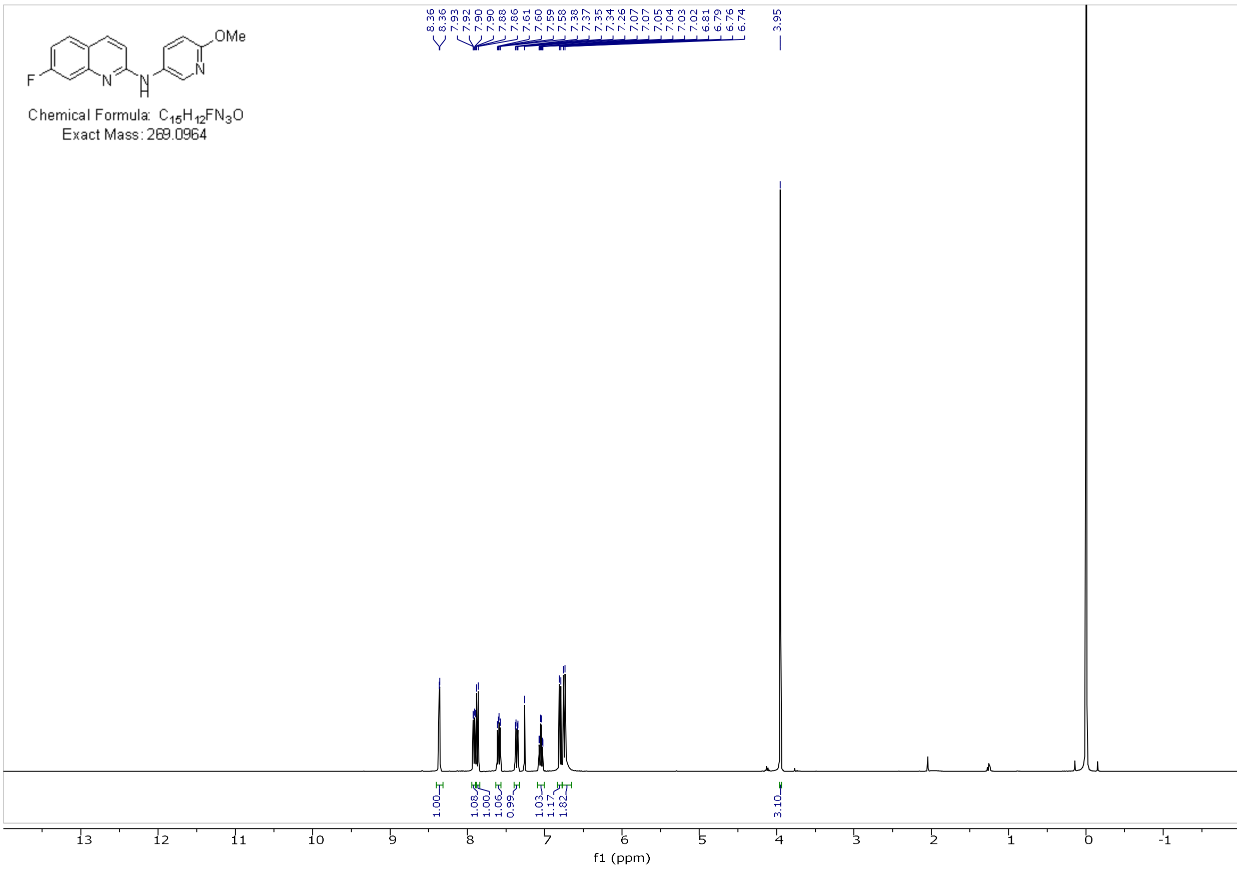

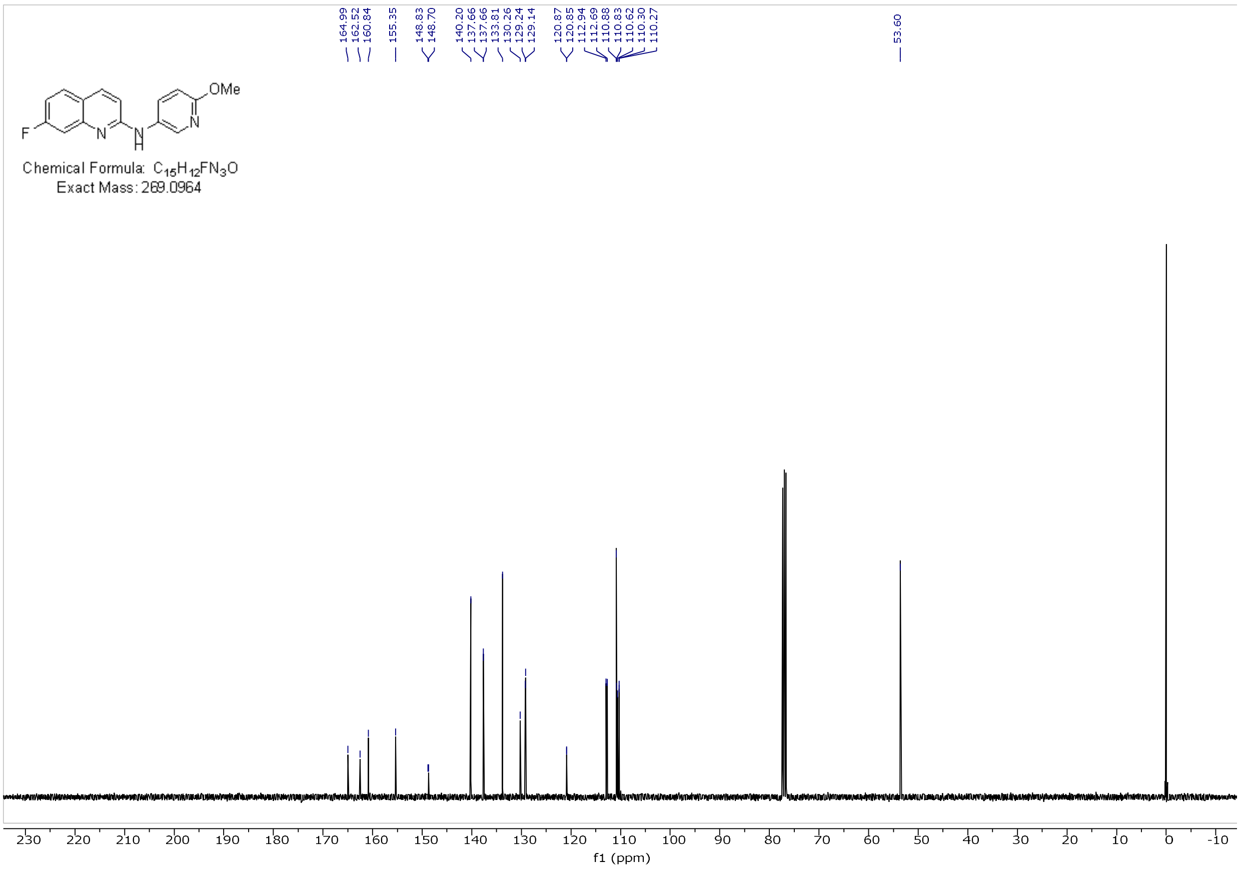

8-Fluoro-*N*-(6-methoxypyridin-3-yl)quinolin-2-amine (5f, TZ64-16).

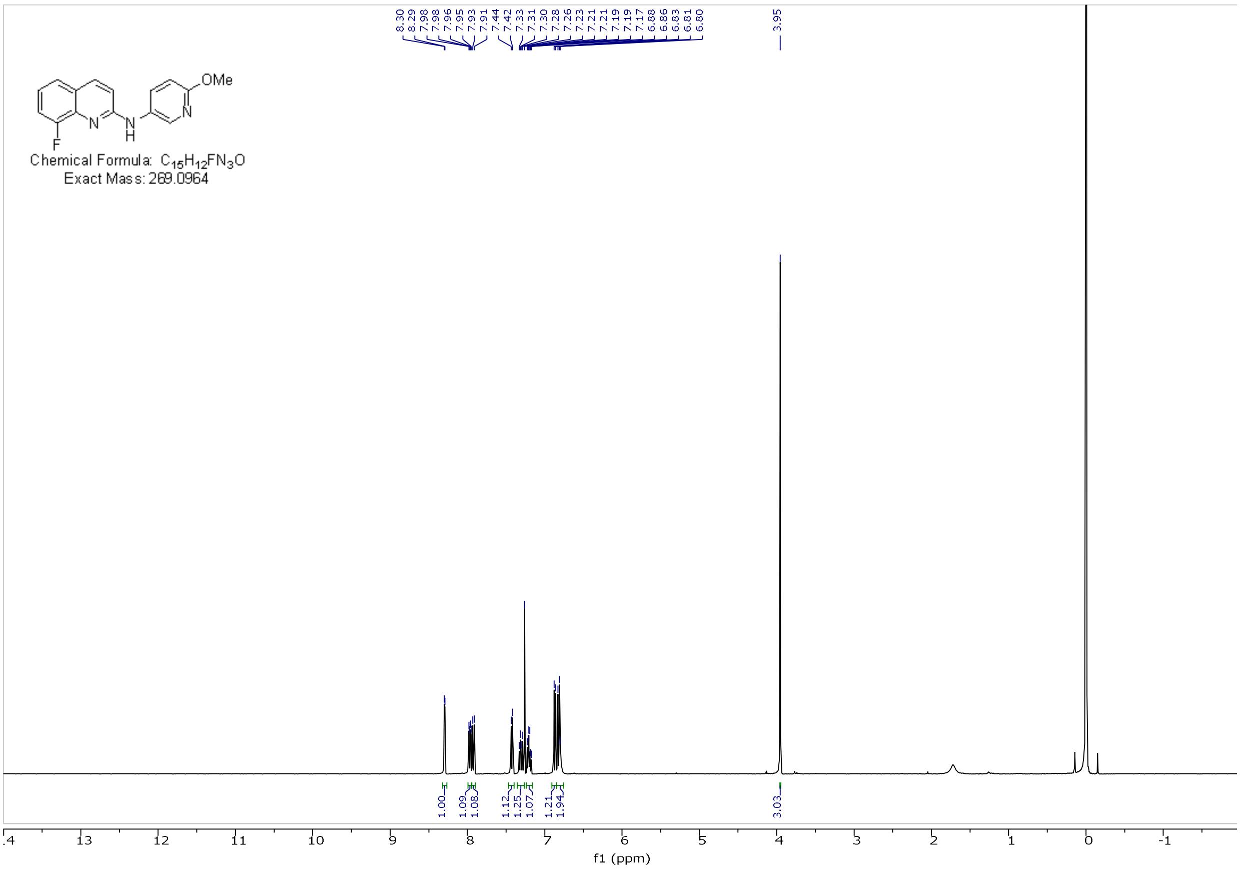

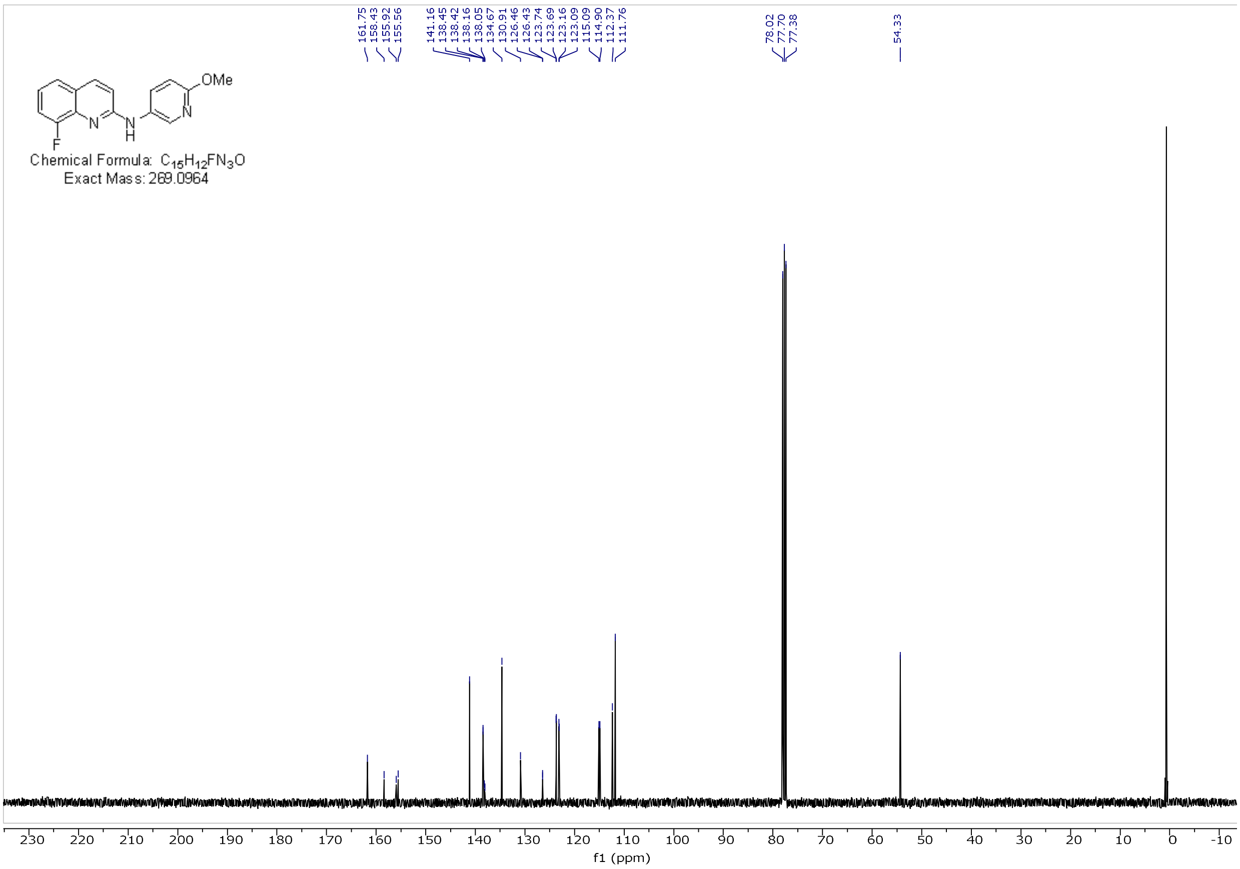

***N*-(6-Methoxypyridin-3-yl)-6-nitroquinolin-2-amine (7a, TZ90-9).**

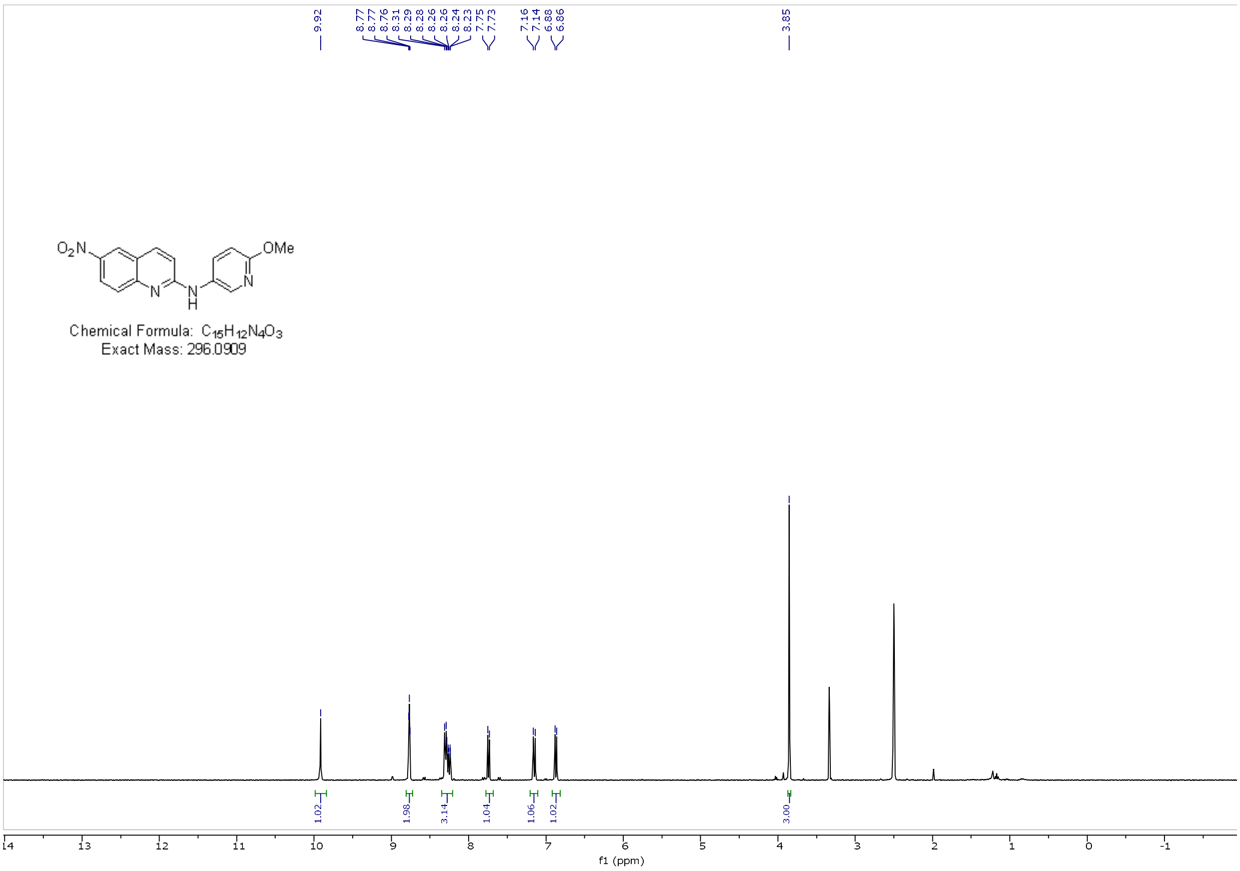

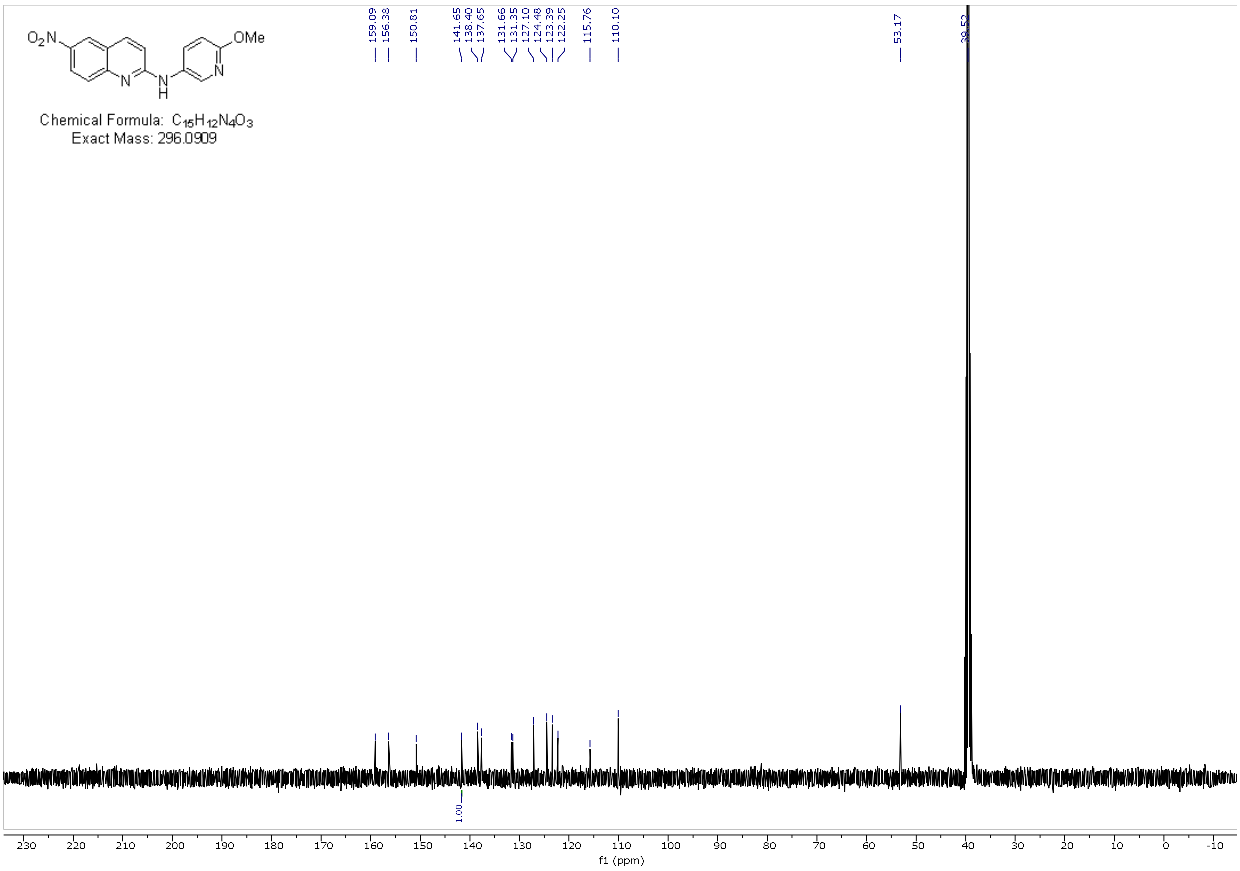

N^2-(6-methoxypyridin-3-yl)-N^6,N^6-dimethylquinoline-2,6-diamine (7b, TZ90-11).

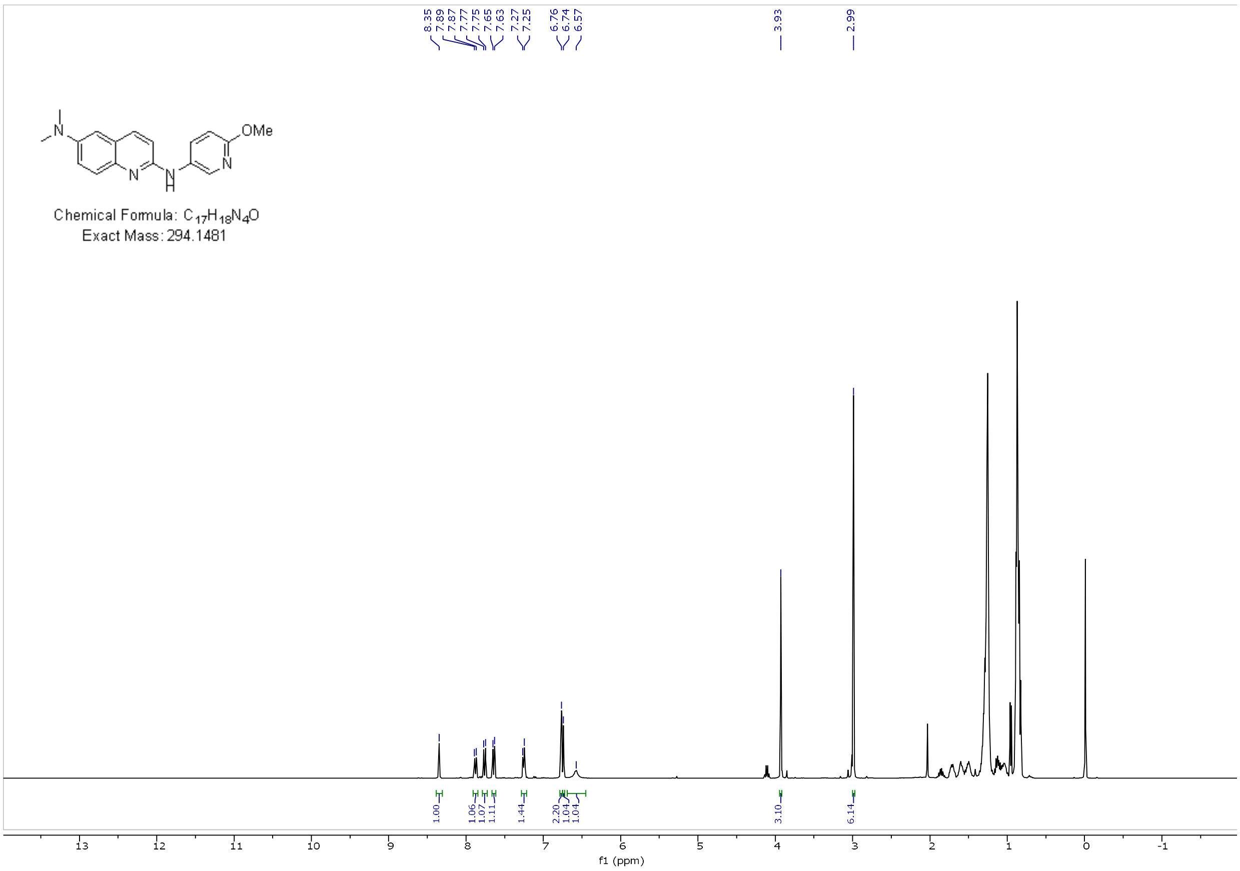

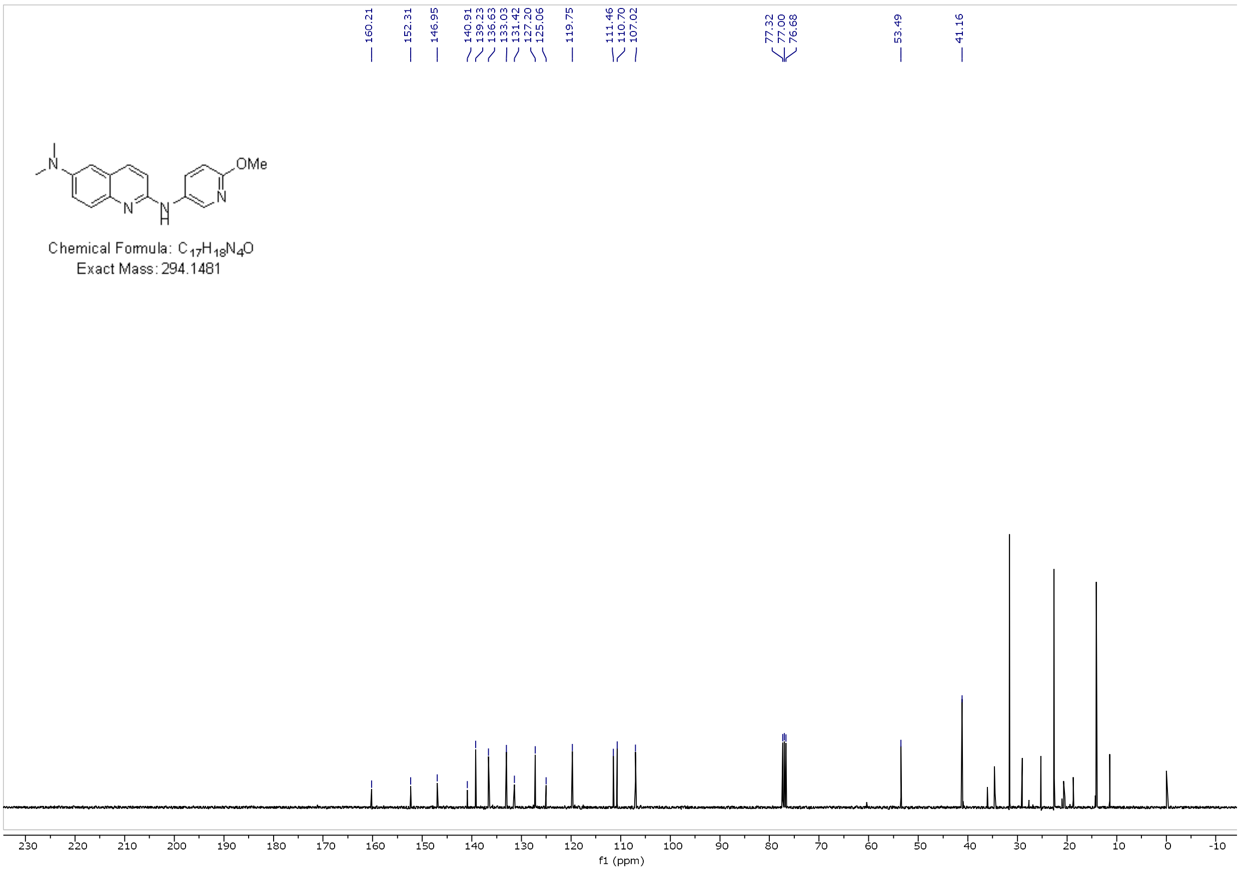

N^2-(6-methoxypyridin-3-yl)quinoline-2,6-diamine (7c, TZ90-12).

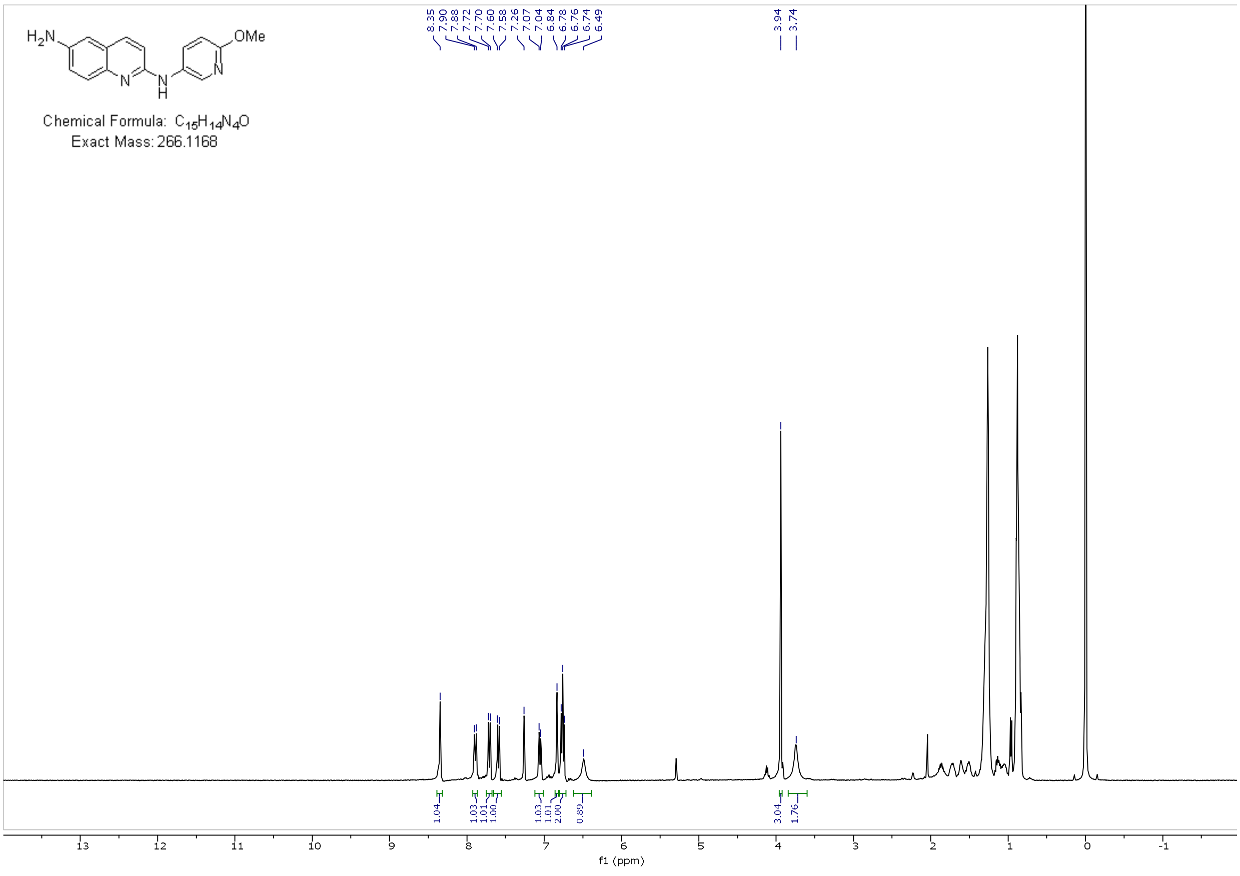

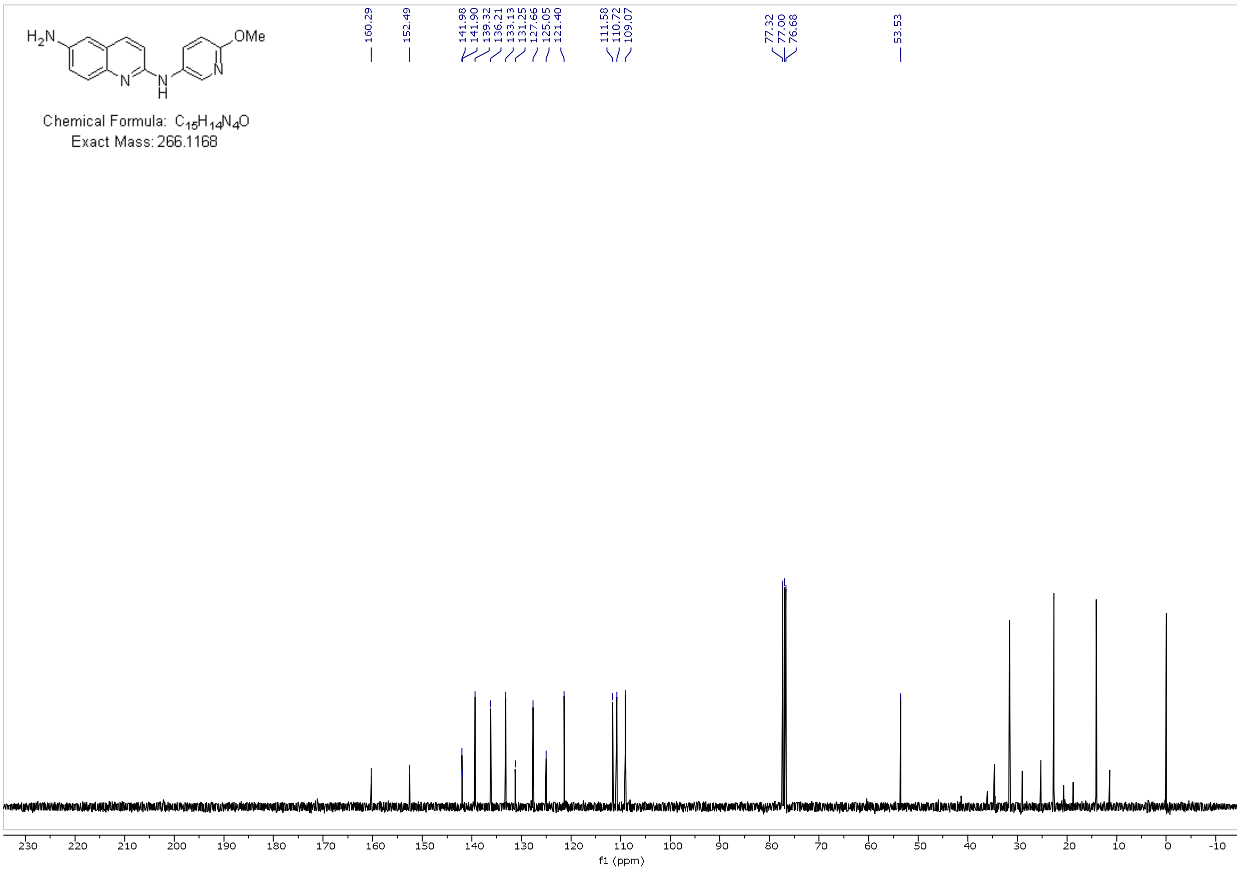

6-Bromo-N-(6-methoxypyridin-3-yl)quinolin-2-amine (7d, TZ64-105).

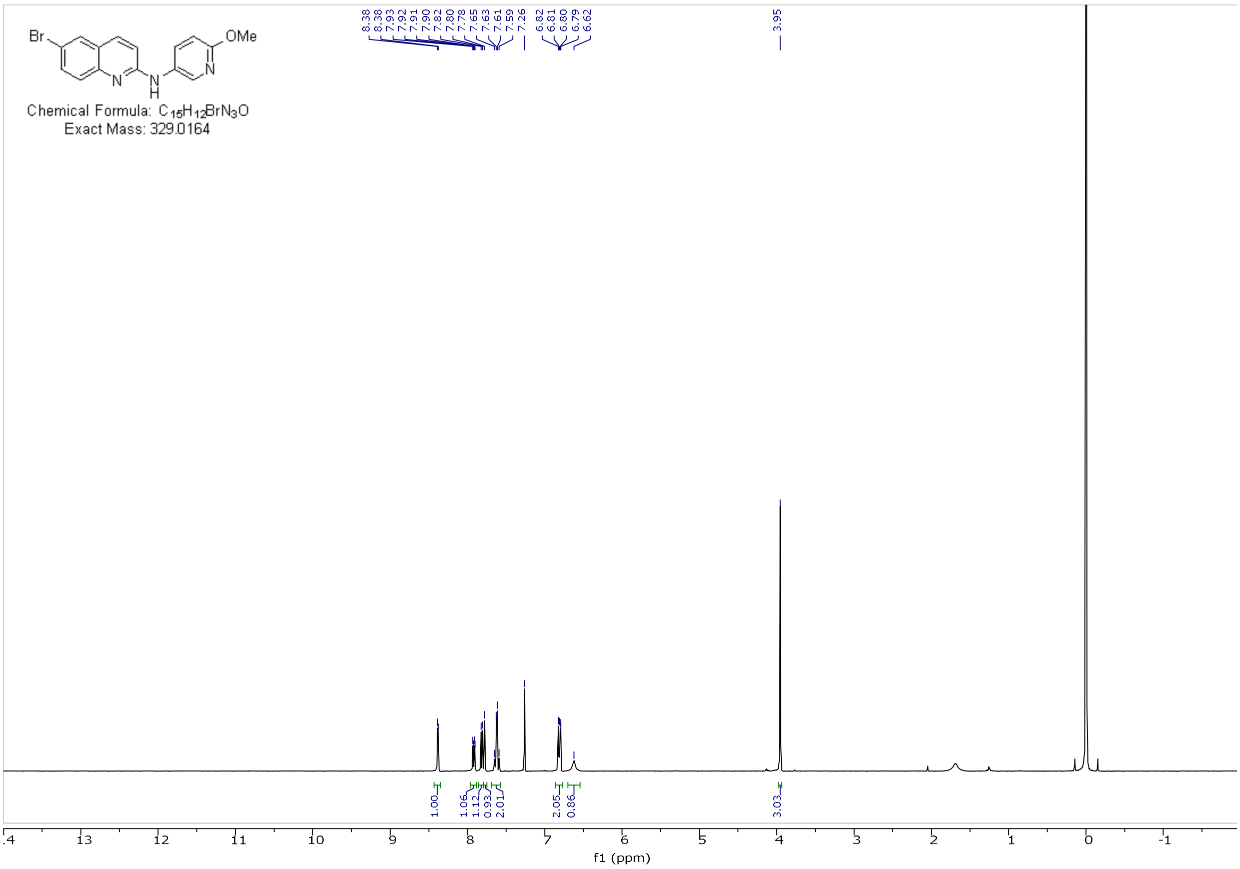

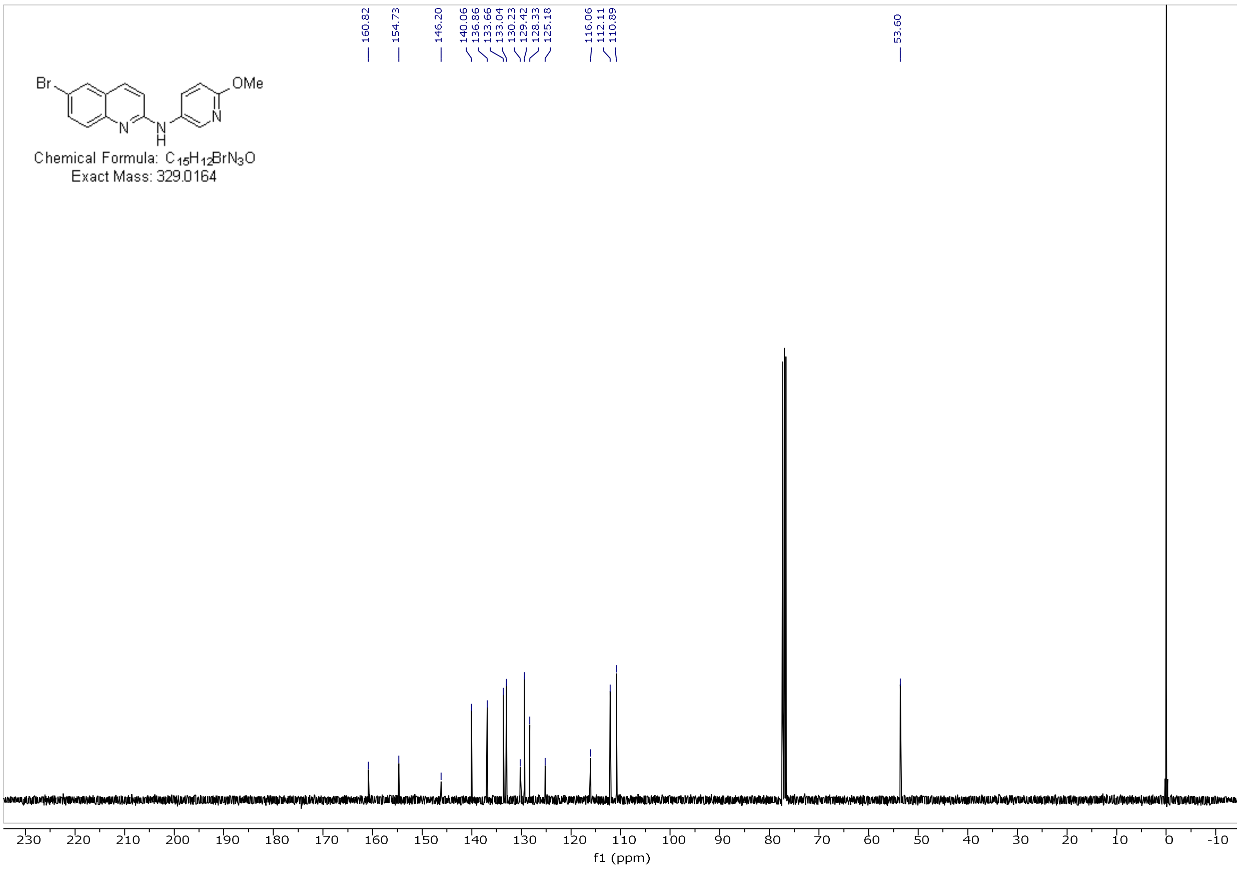

2-((6-Methoxypyridin-3-yl)amino)quinolin-6-ol (7e, TZ80-34).

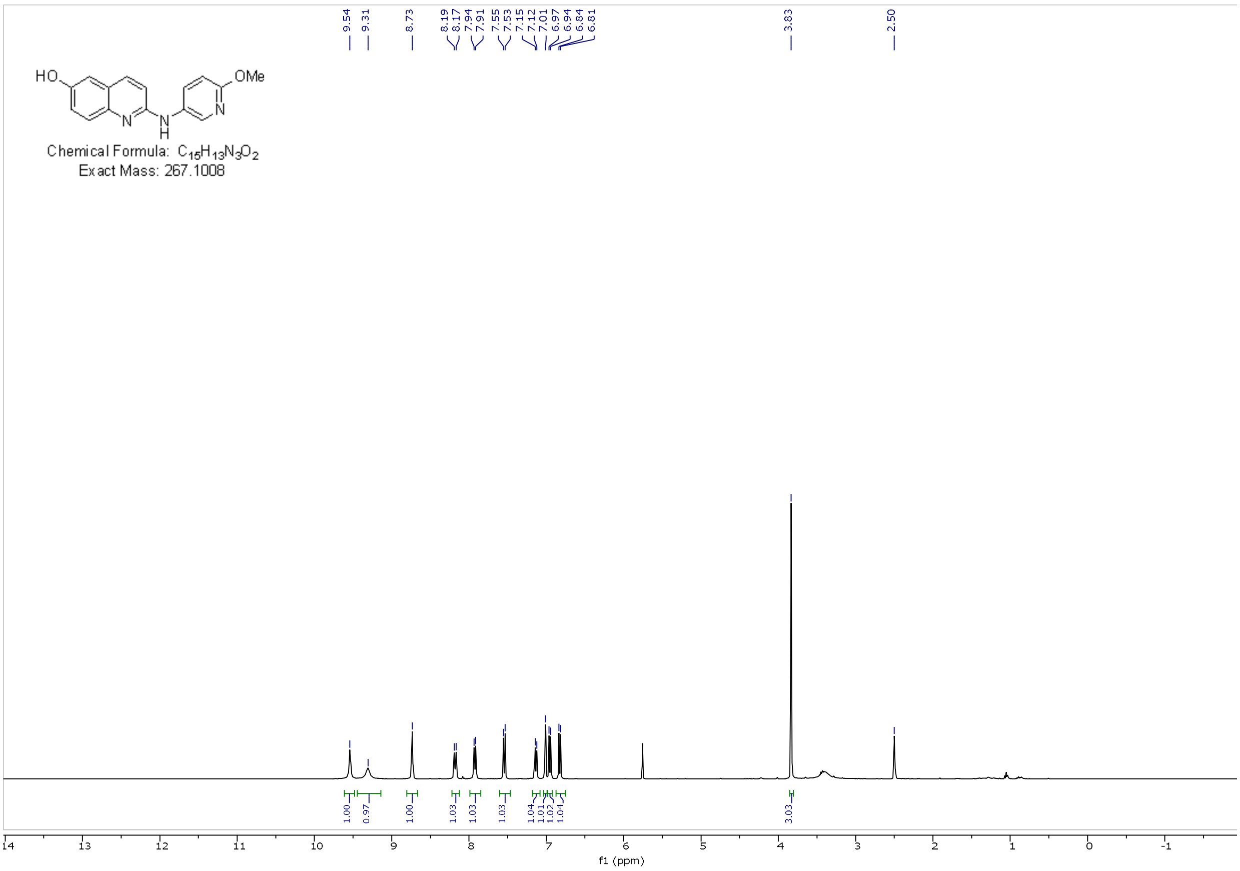

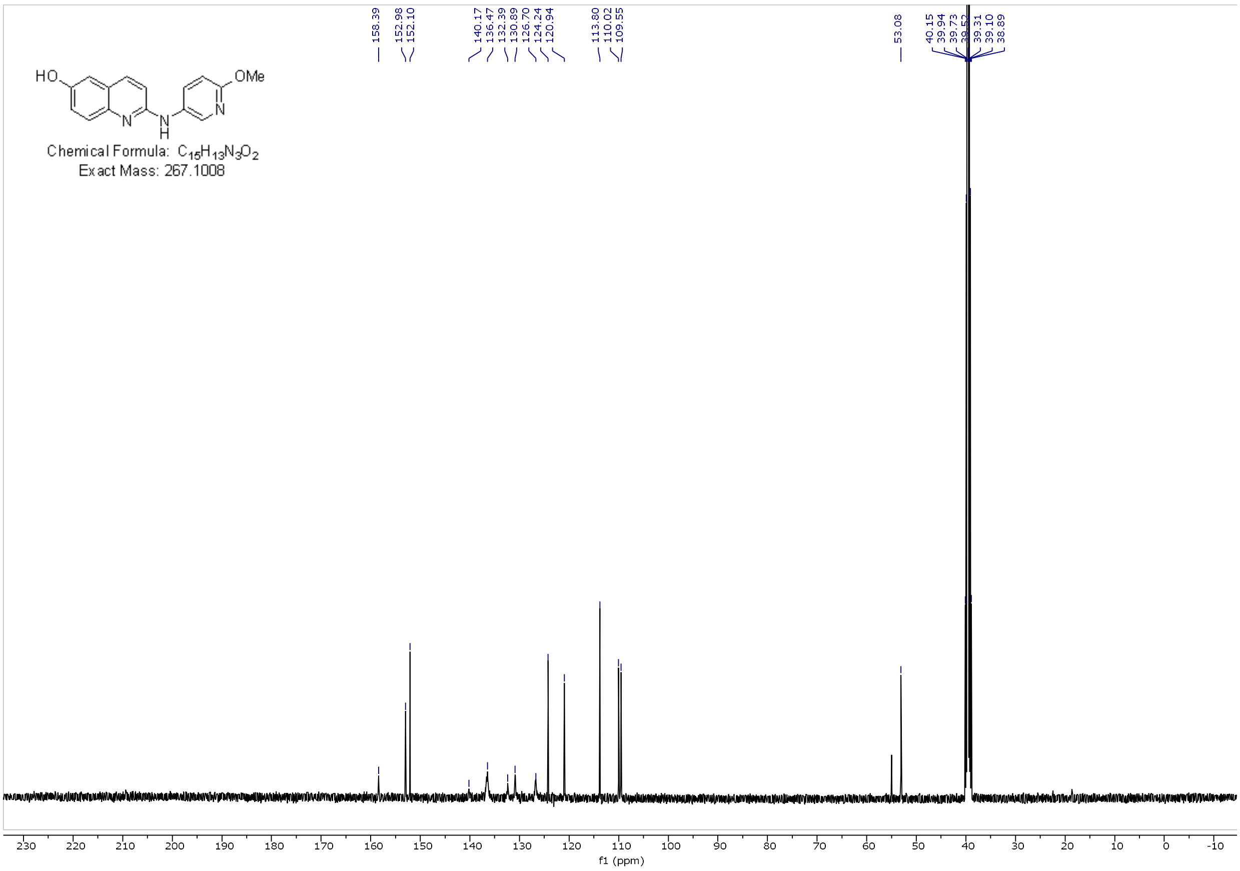

6-Methoxy-*N*-(6-methoxypyridin-3-yl)quinolin-2-amine (7f, TZ55-107).

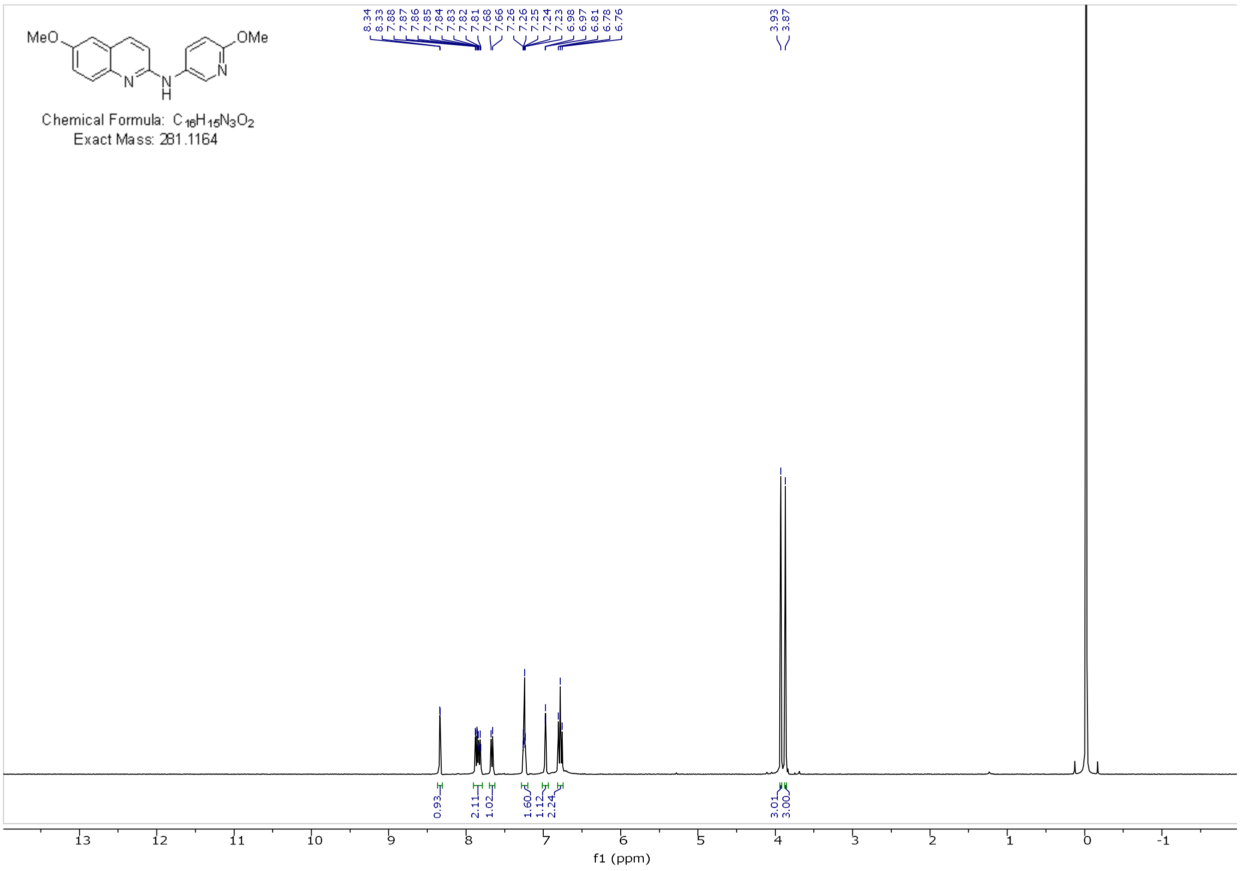

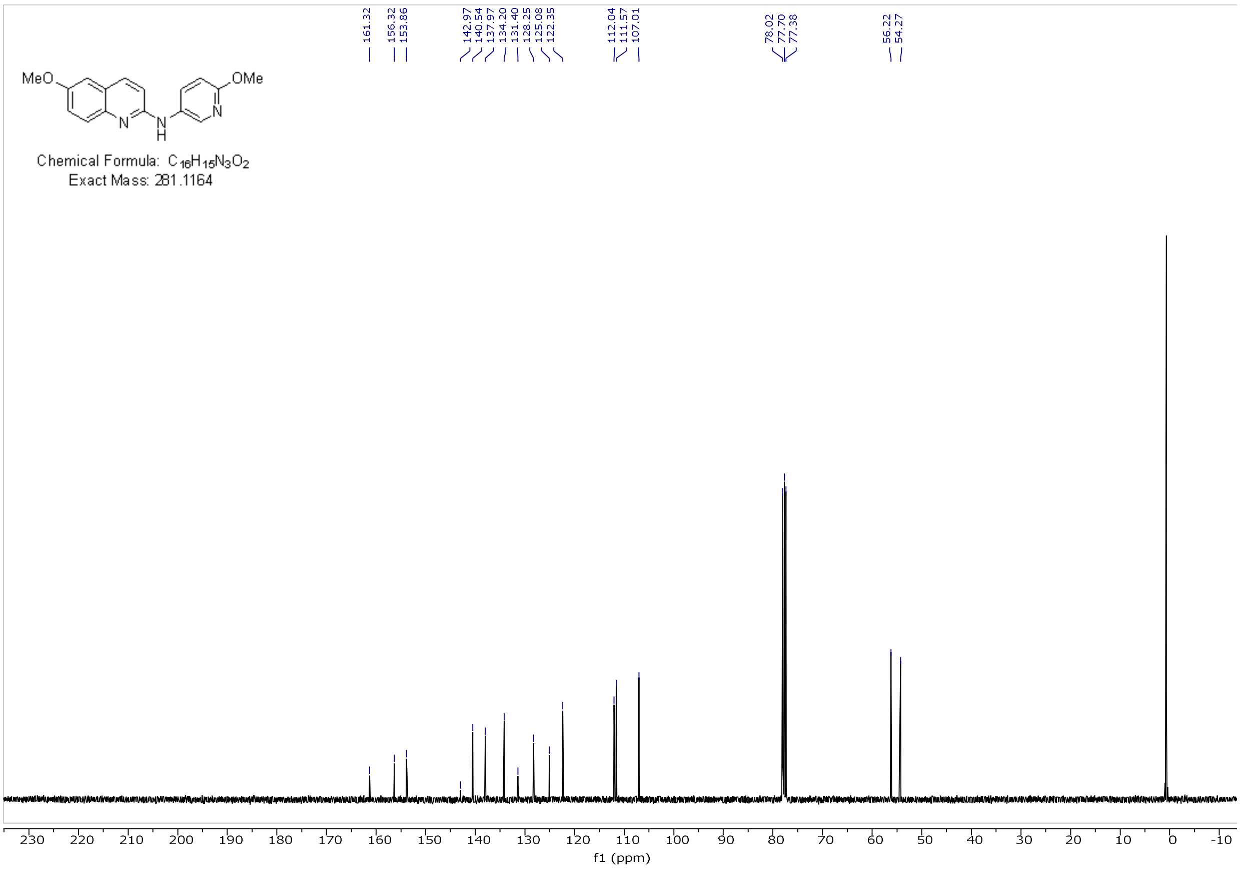

6-(Difluoromethoxy)-*N*-(6-methoxypyridin-3-yl)quinolin-2-amine (7g, TZ80-37).

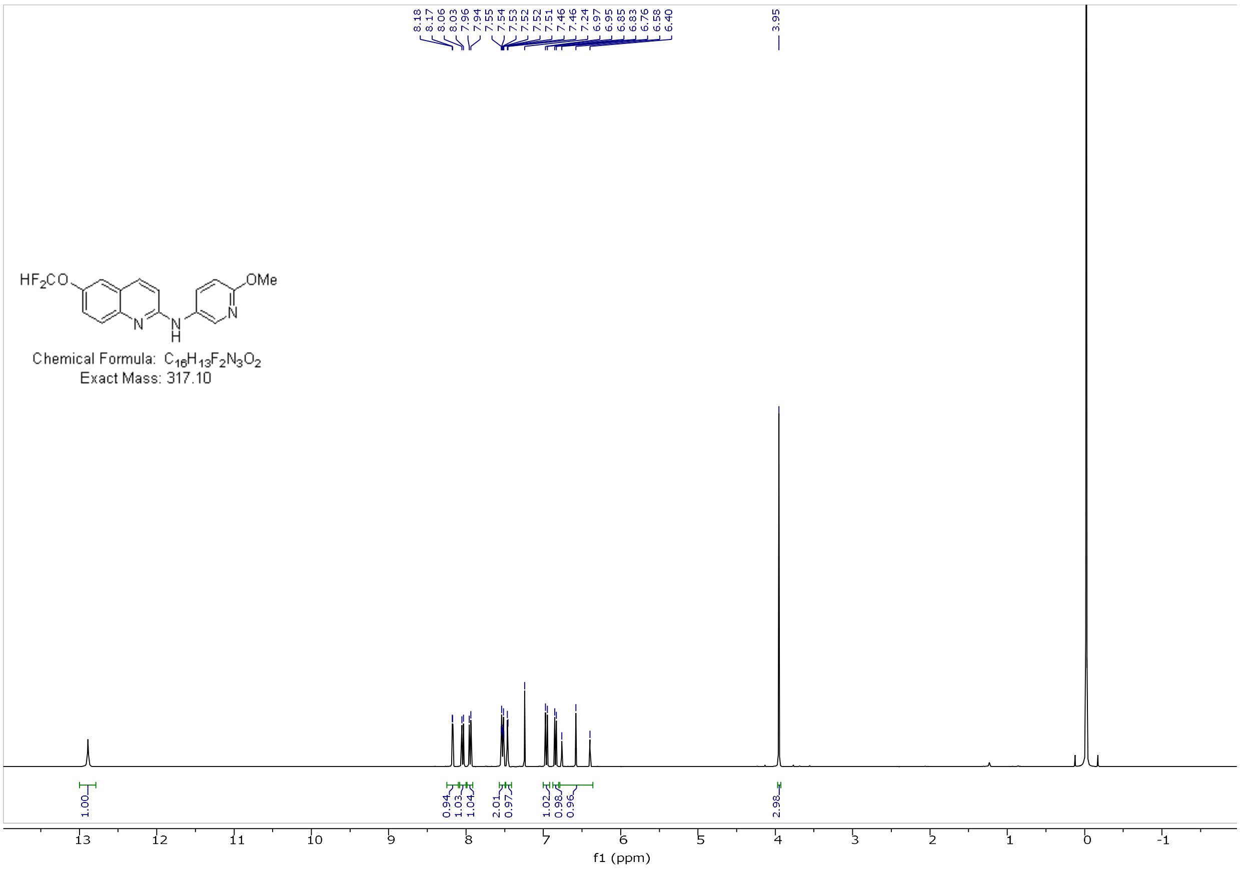

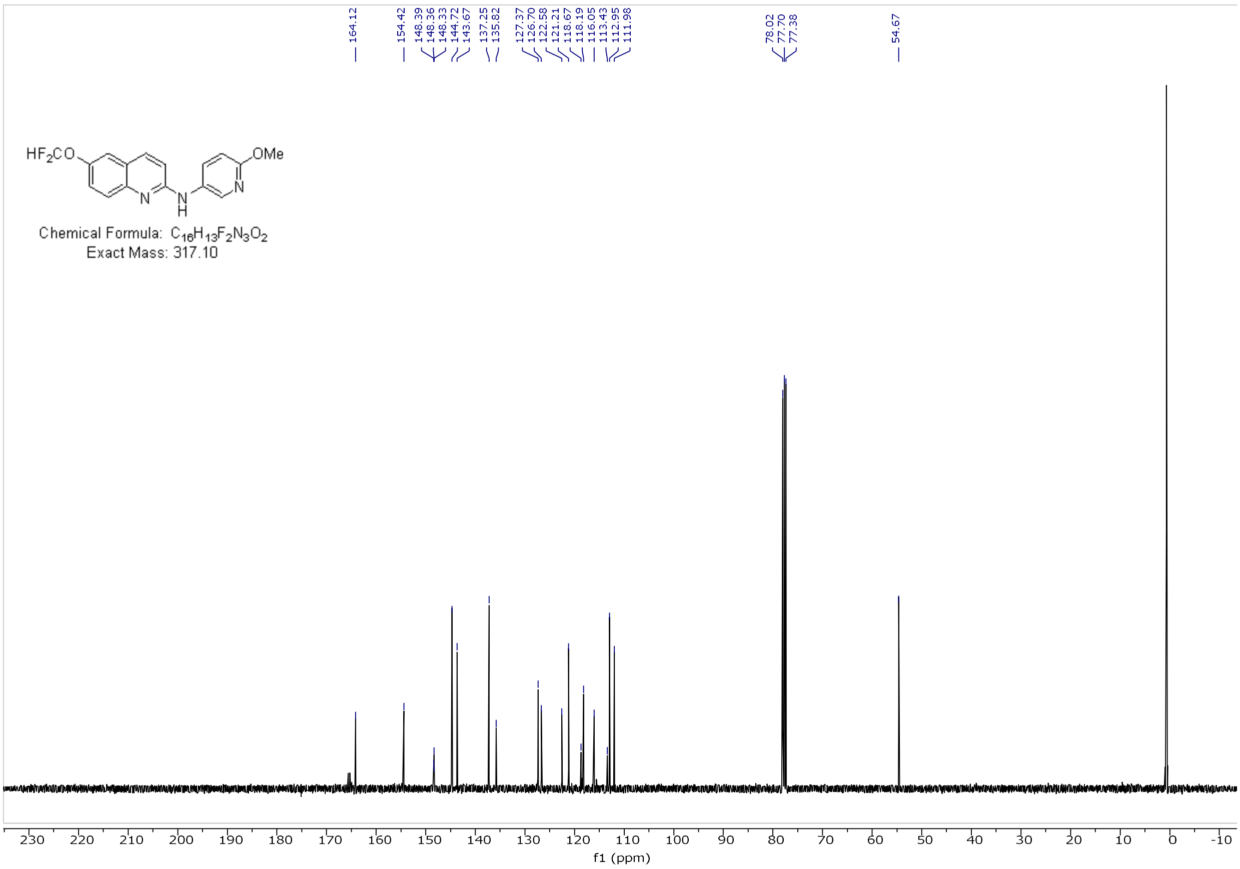

N-(6-Methoxypyridin-3-yl)-6-(trifluoromethoxy)quinolin-2-amine (7h, TZ90-17).

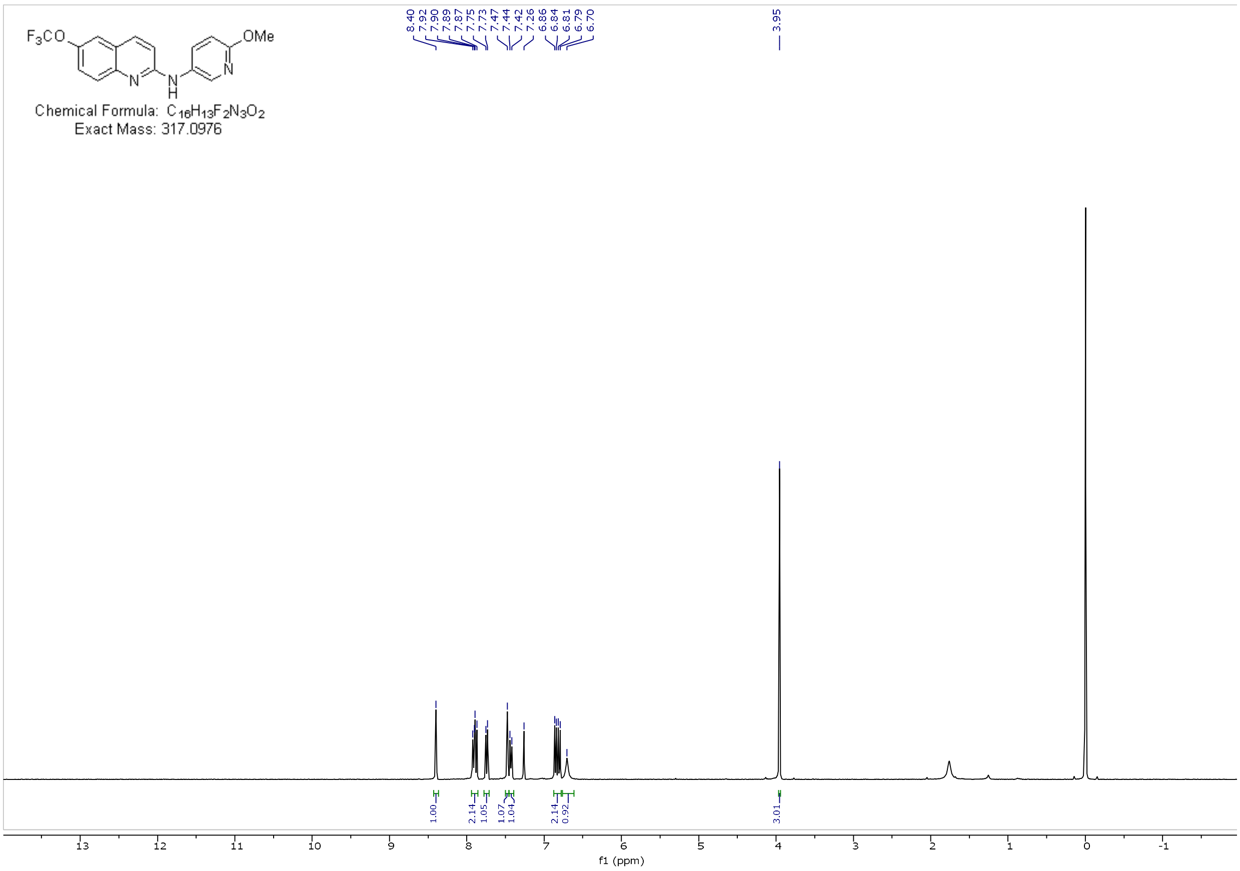

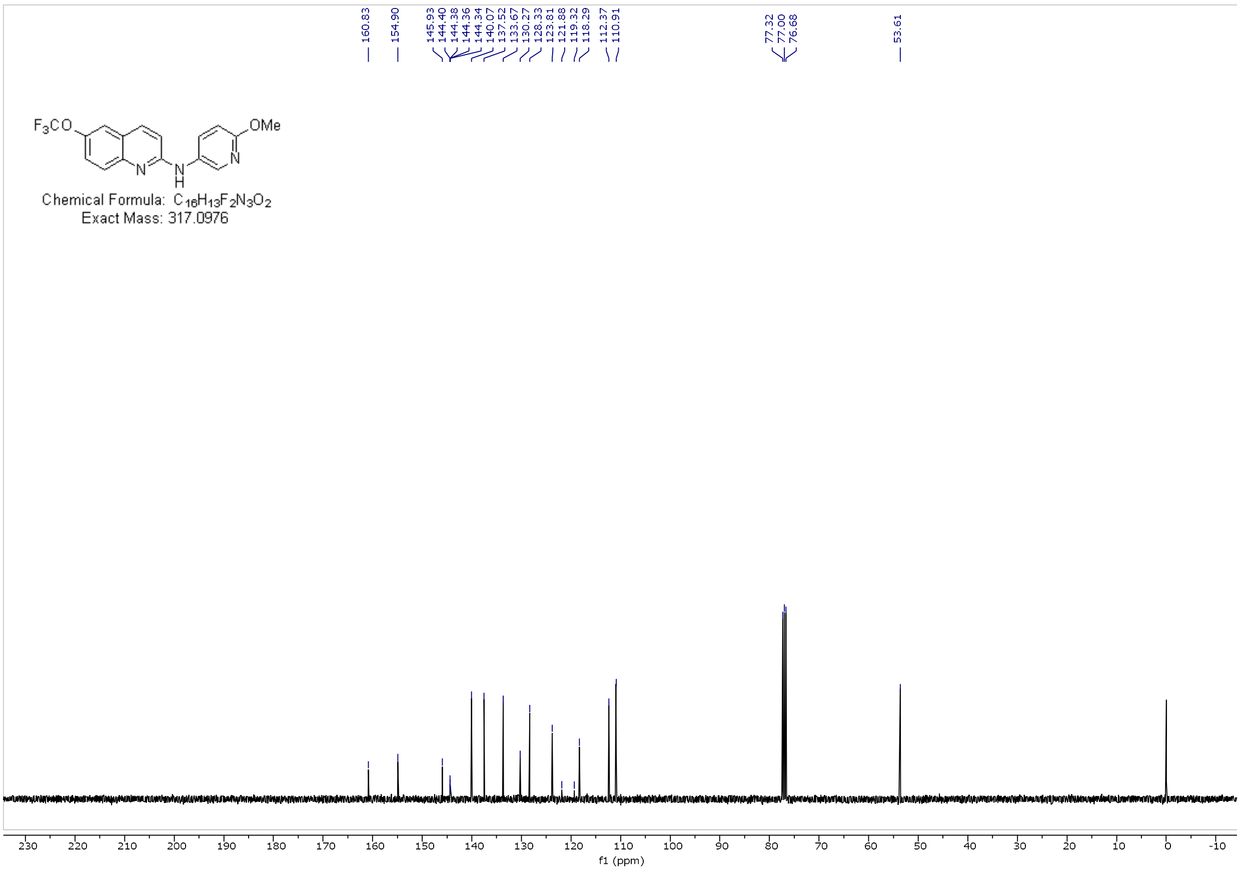

2-((6-Methoxypyridin-3-yl)amino)quinolin-6-yl sulfurofluoridate (7i, TZ80-151).

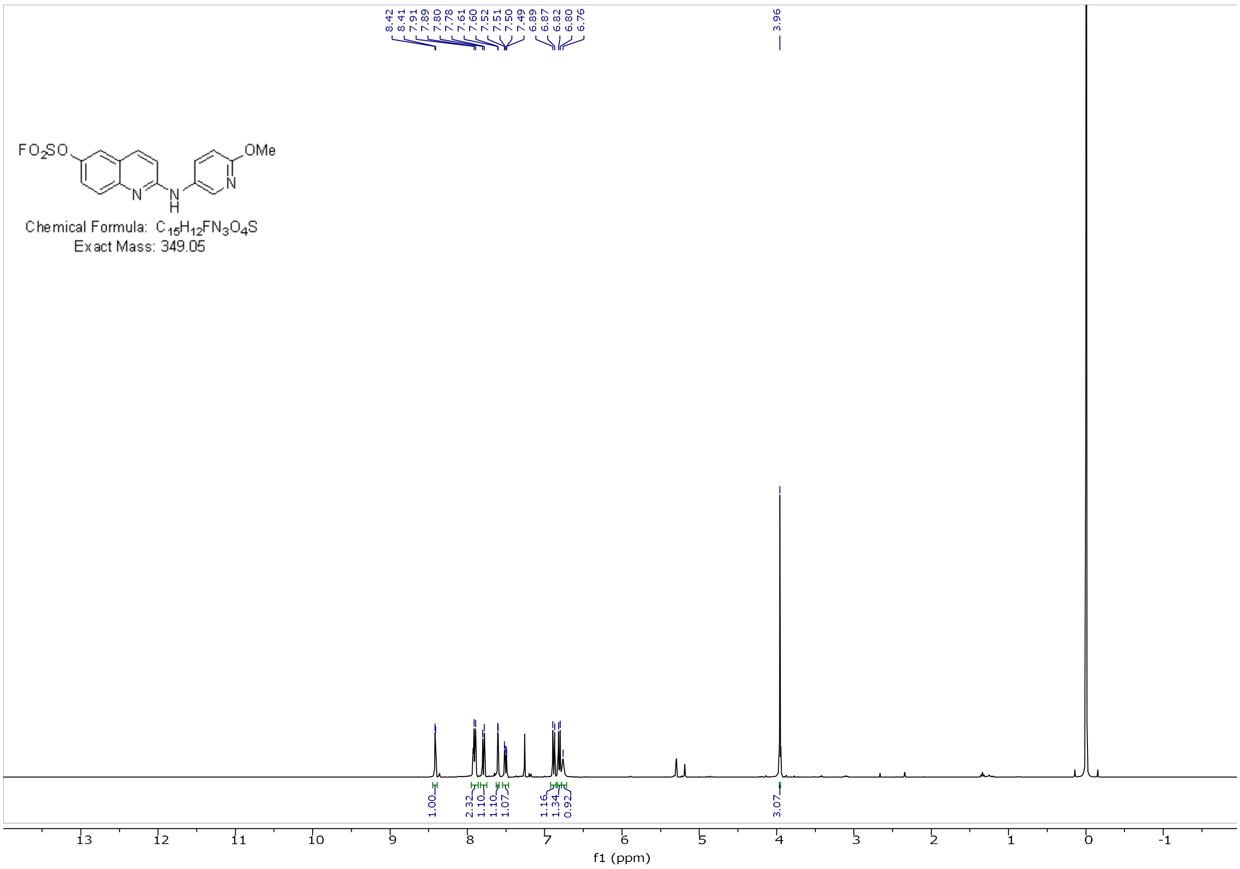

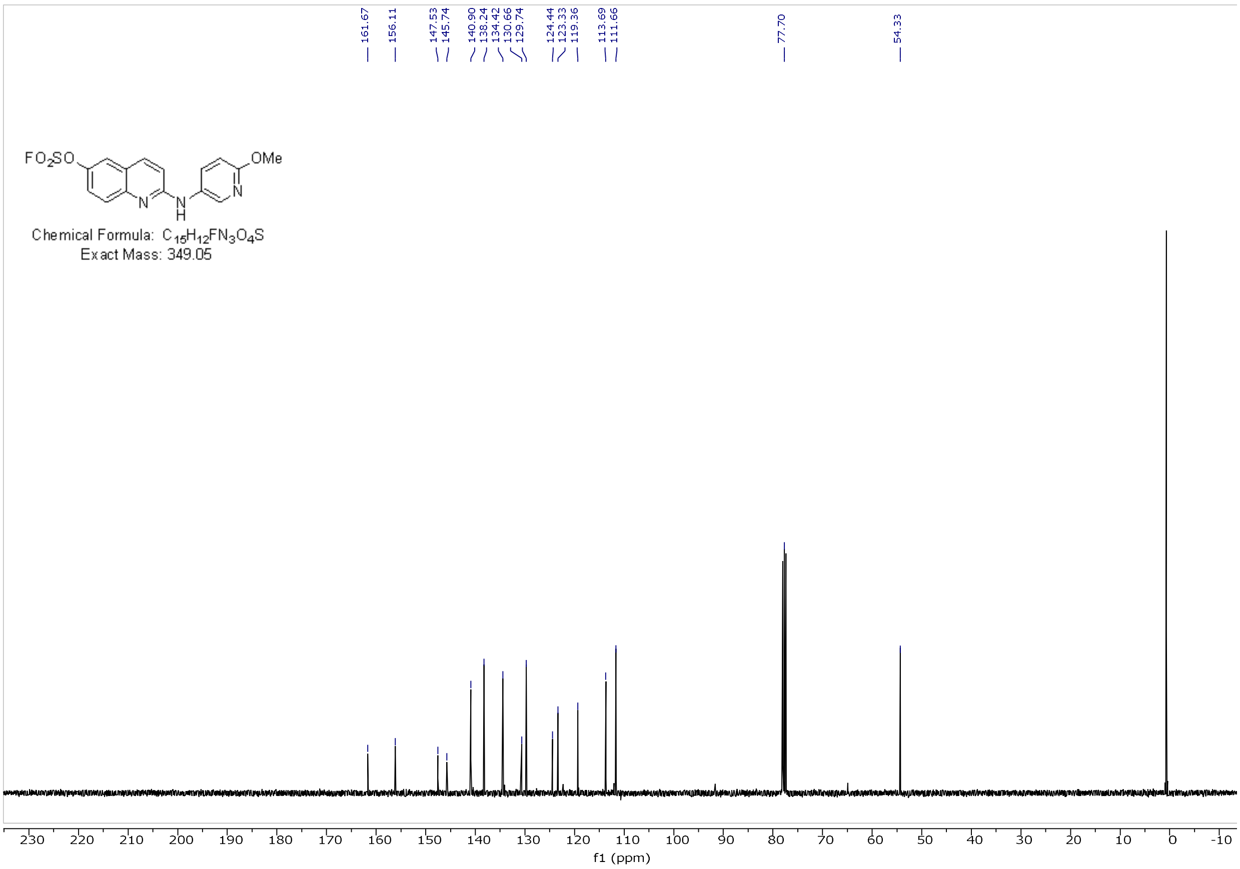

6-(2-Fluoroethoxy)-*N*-(6-methoxypyridin-3-yl)quinolin-2-amine (7j, TZ90-3).

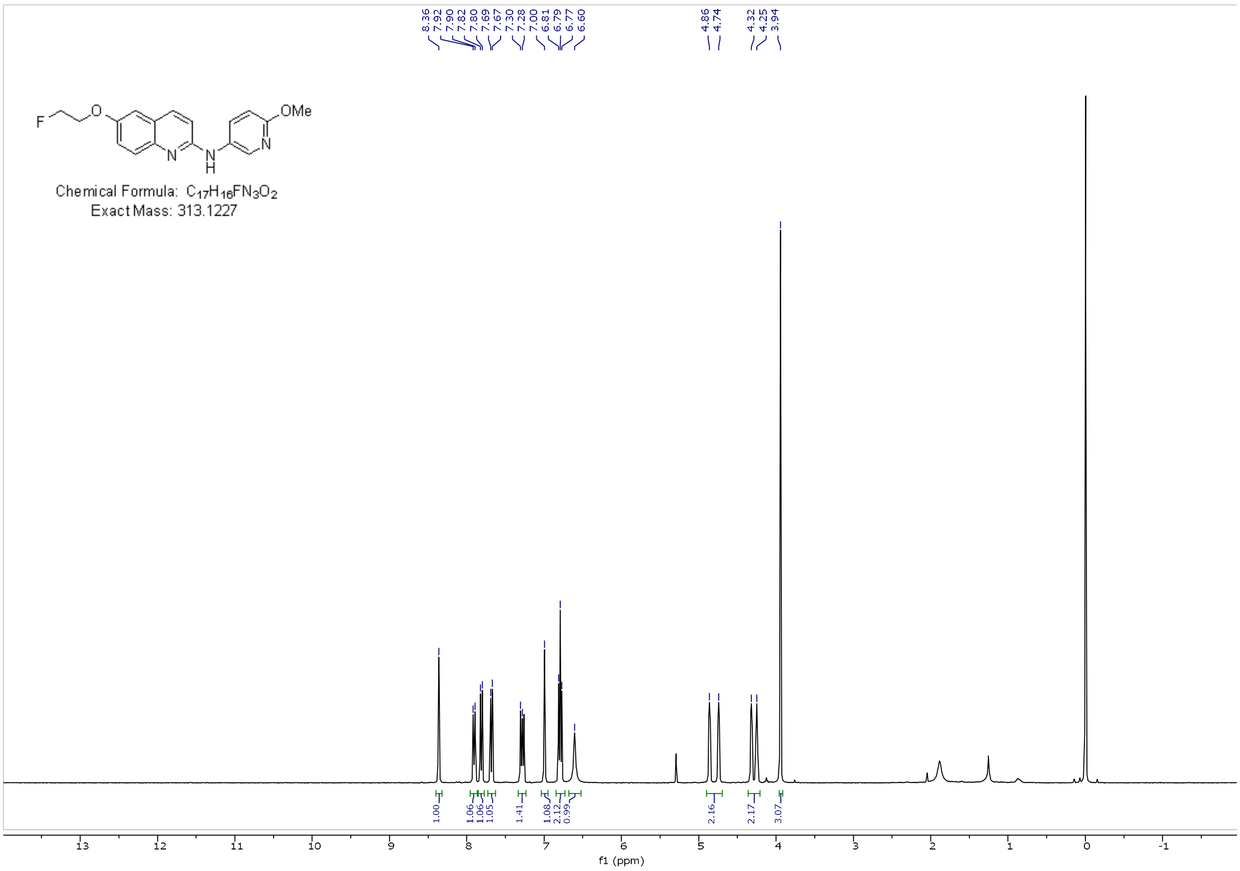

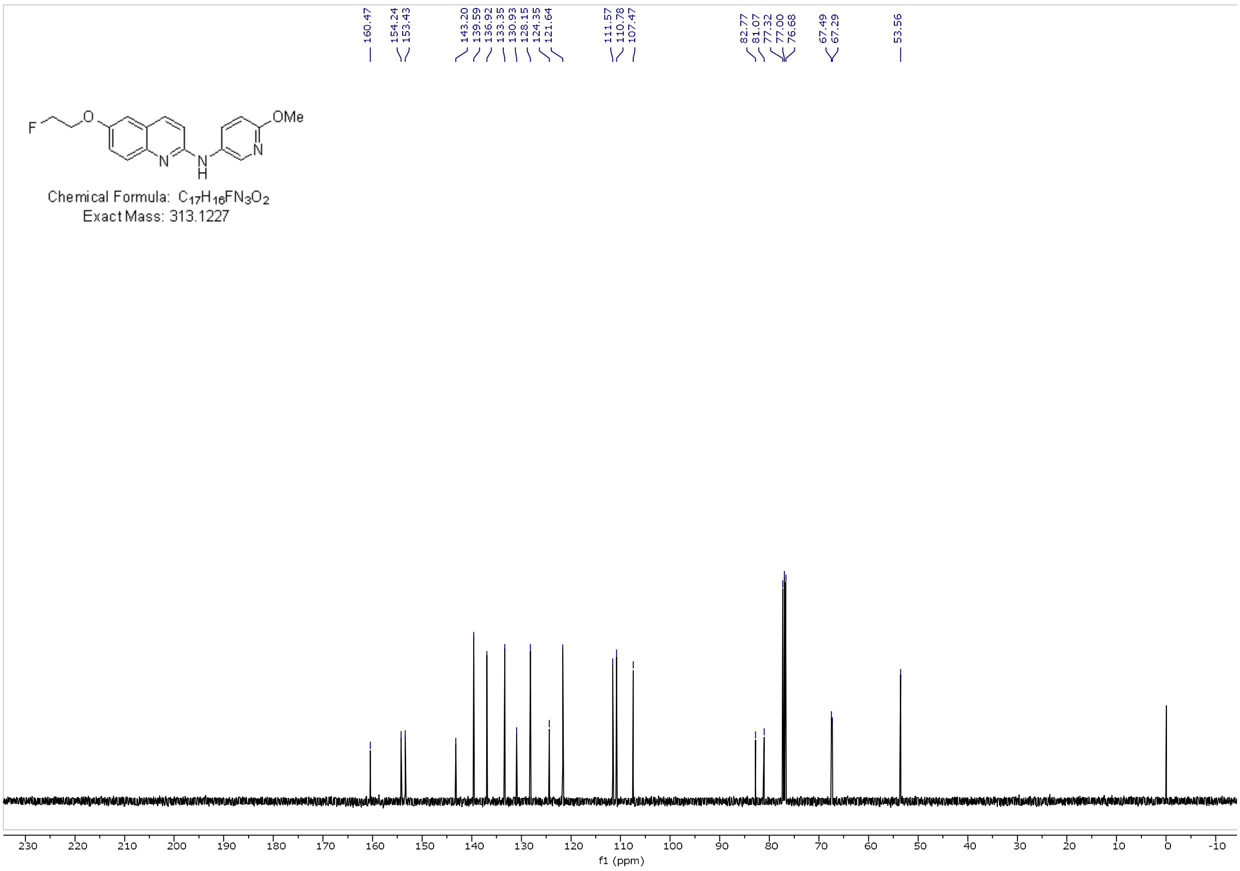

6-(2-(2-Fluoroethoxy)ethoxy)-*N*-(6-methoxypyridin-3-yl)quinolin-2-amine (7k, TZ80-14).

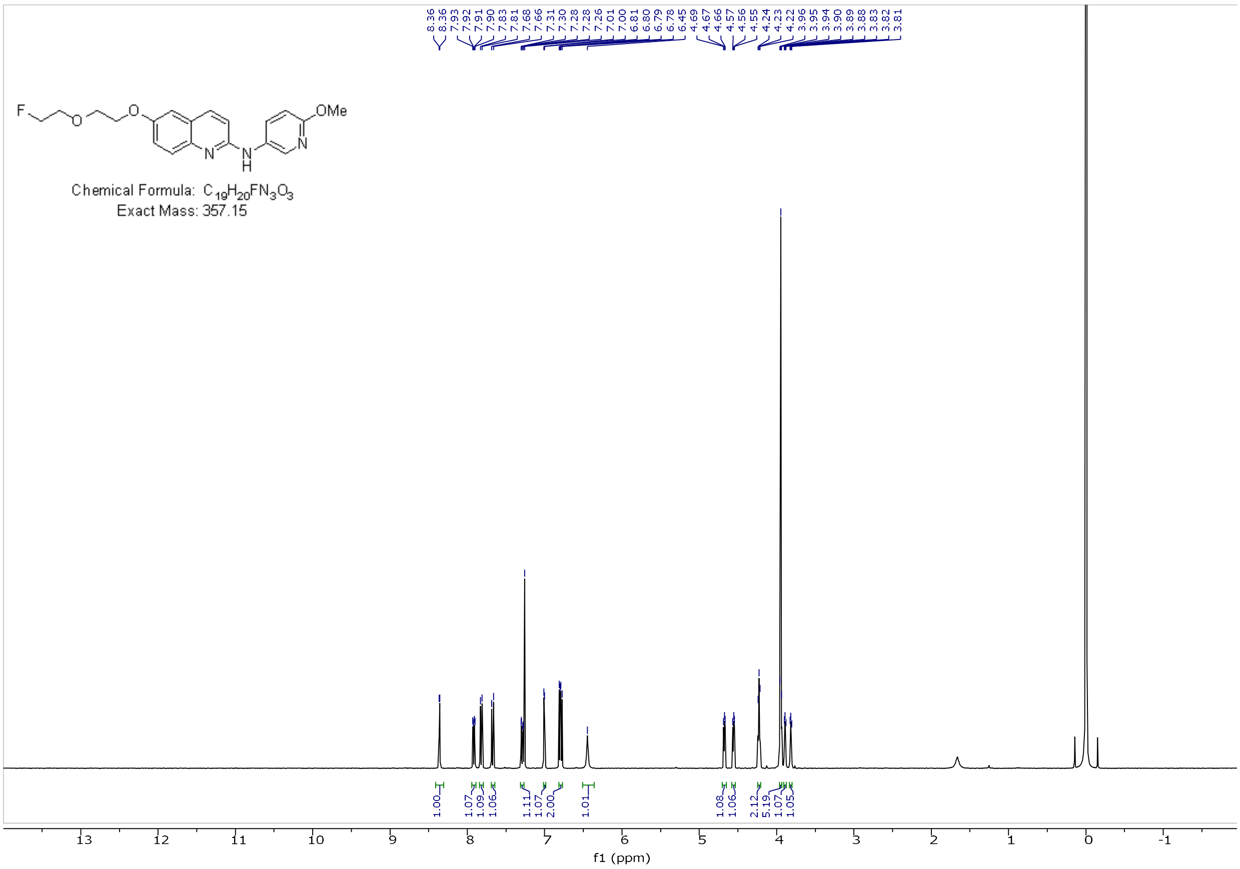

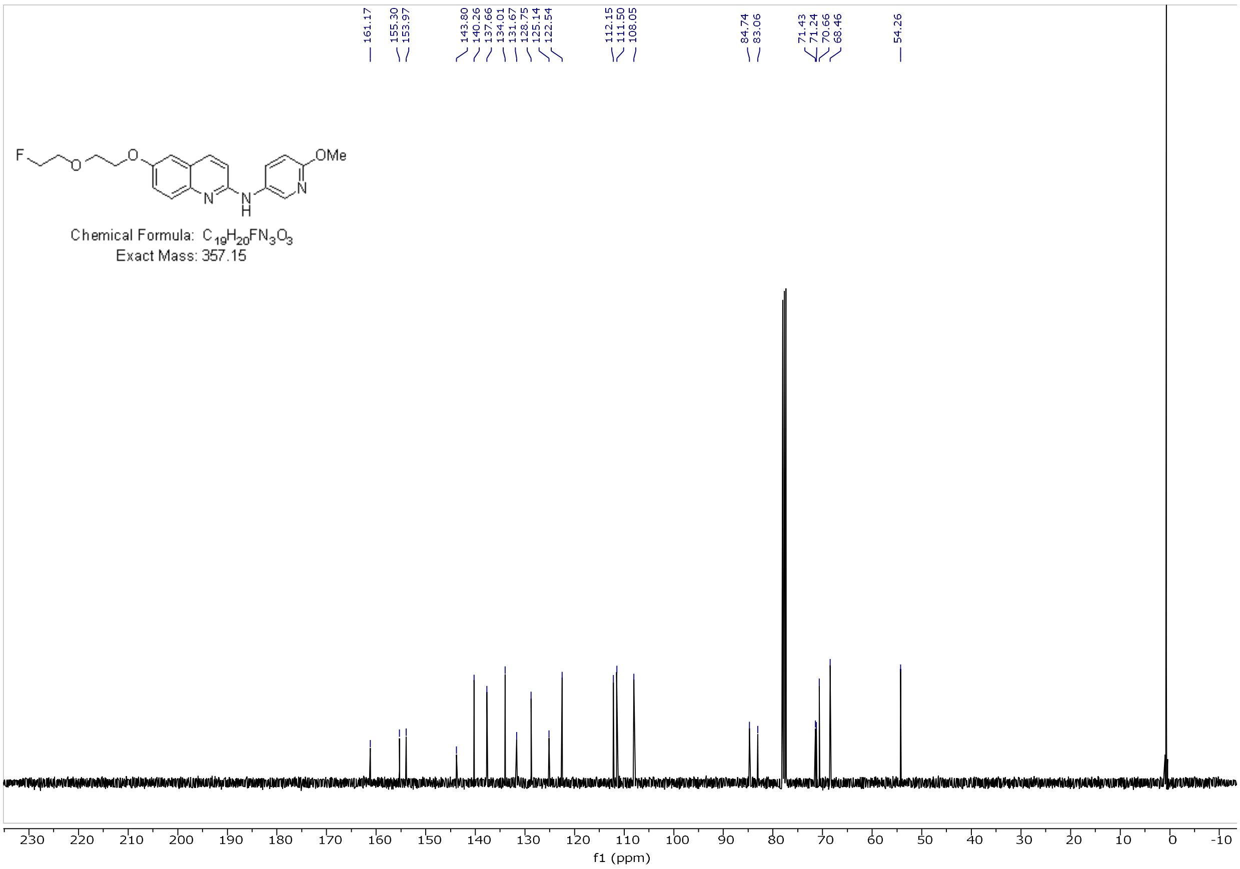

6-(2-(2-(2-Fluoroethoxy)ethoxy)ethoxy)-*N*-(6-methoxypyridin-3-yl)quinolin-2-amine (7l, TZ80-15).

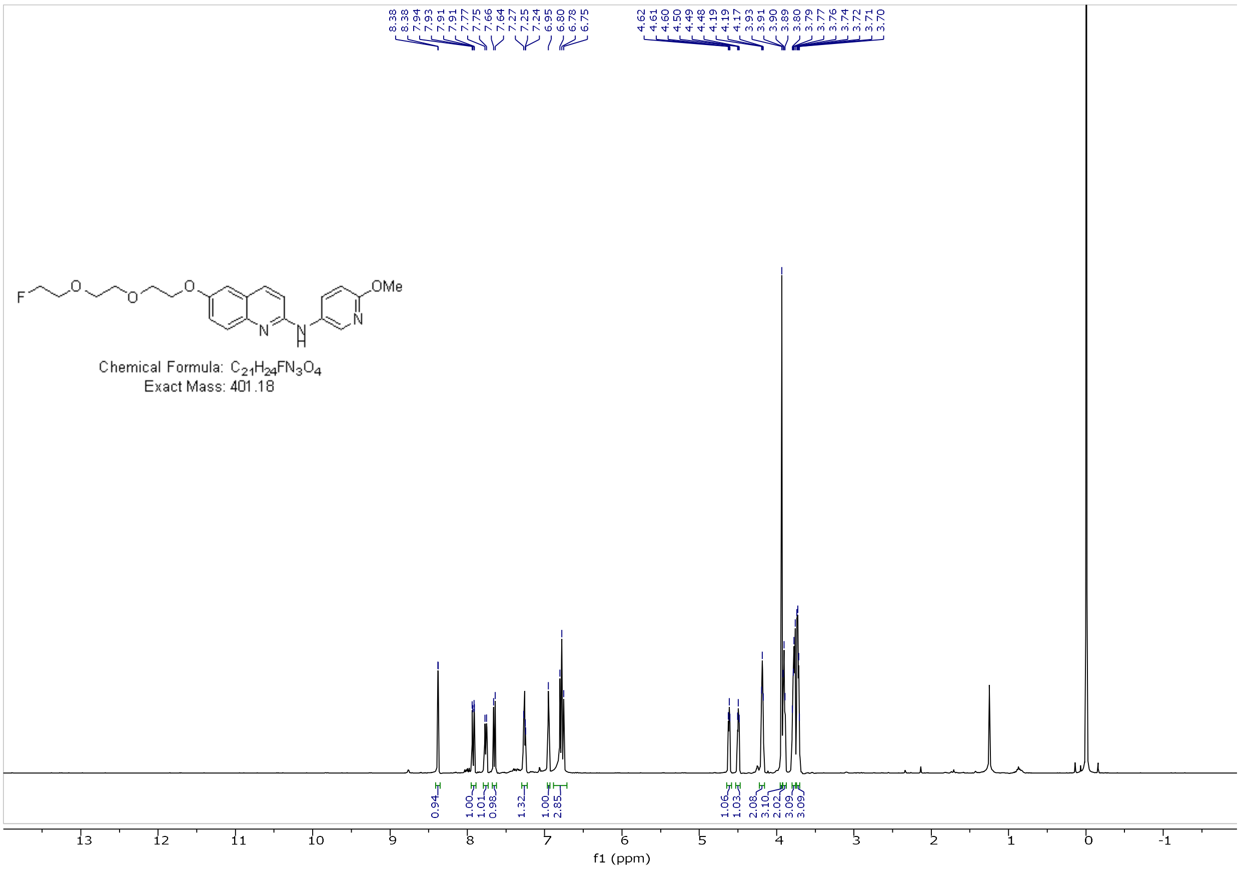

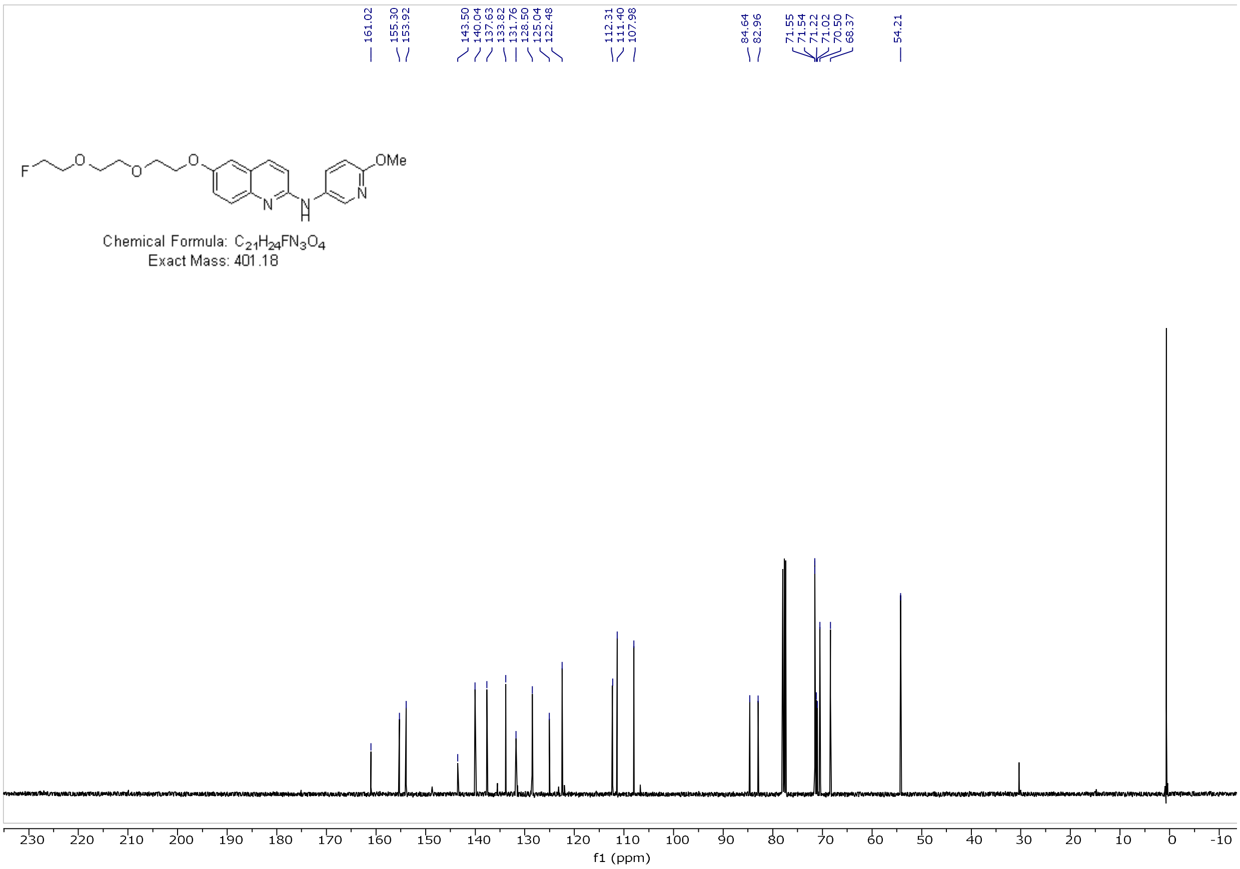

6-(2-(2-(2-(2-Fluoroethoxy)ethoxy)ethoxy)ethoxy)-*N*-(6-methoxypyridin-3-yl)quinolin-2-amine (7m, TZ80-16).

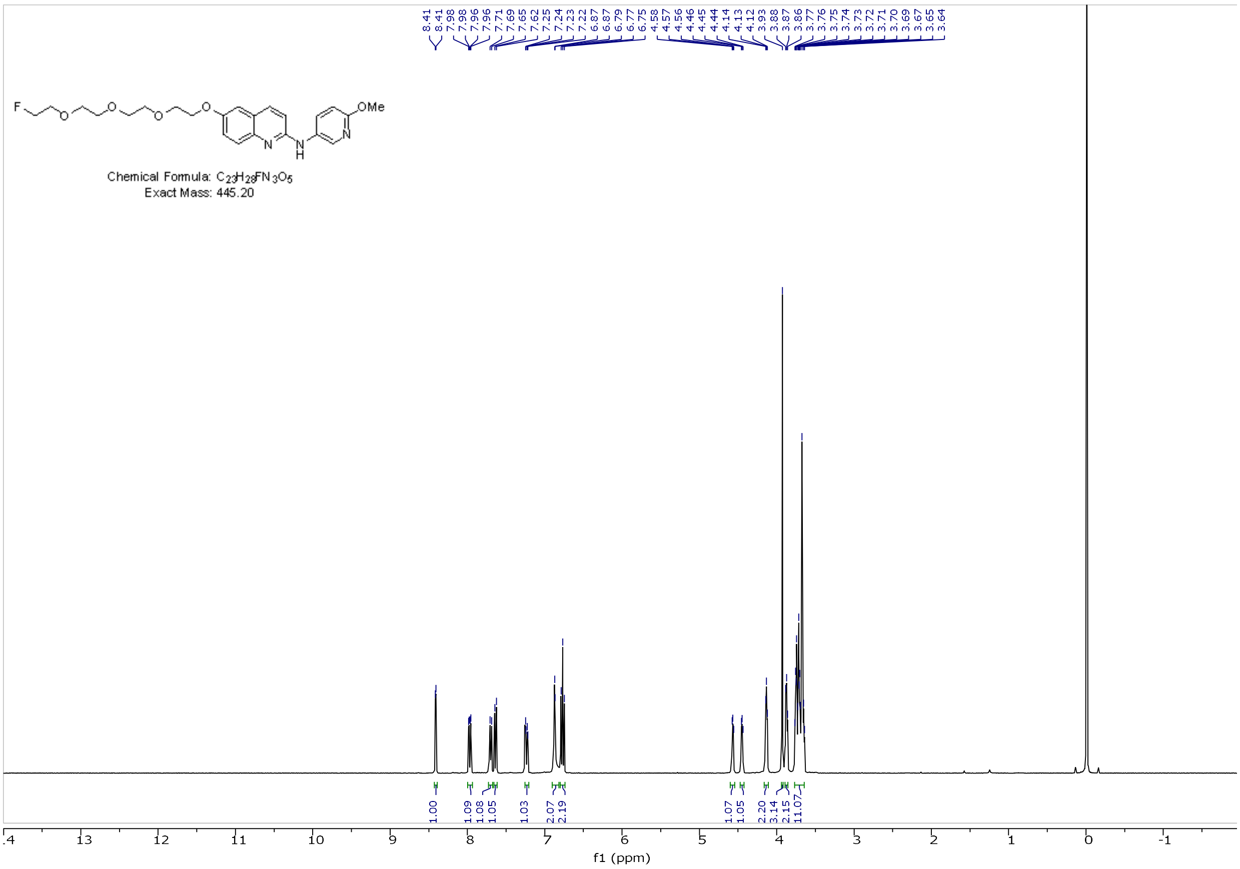

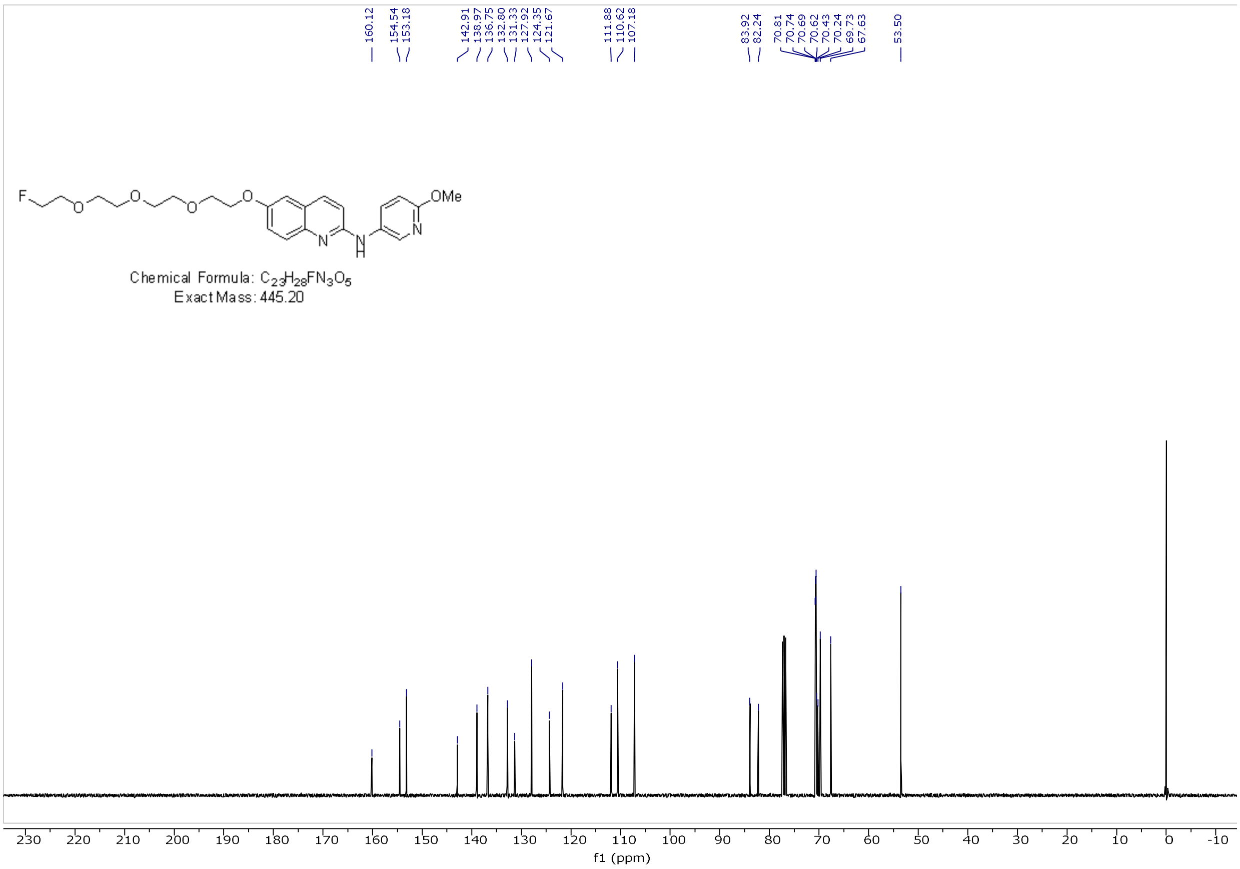

N-(6-Fluoropyridin-3-yl)-6-methoxyquinolin-2-amine (7n, TZ77-107).

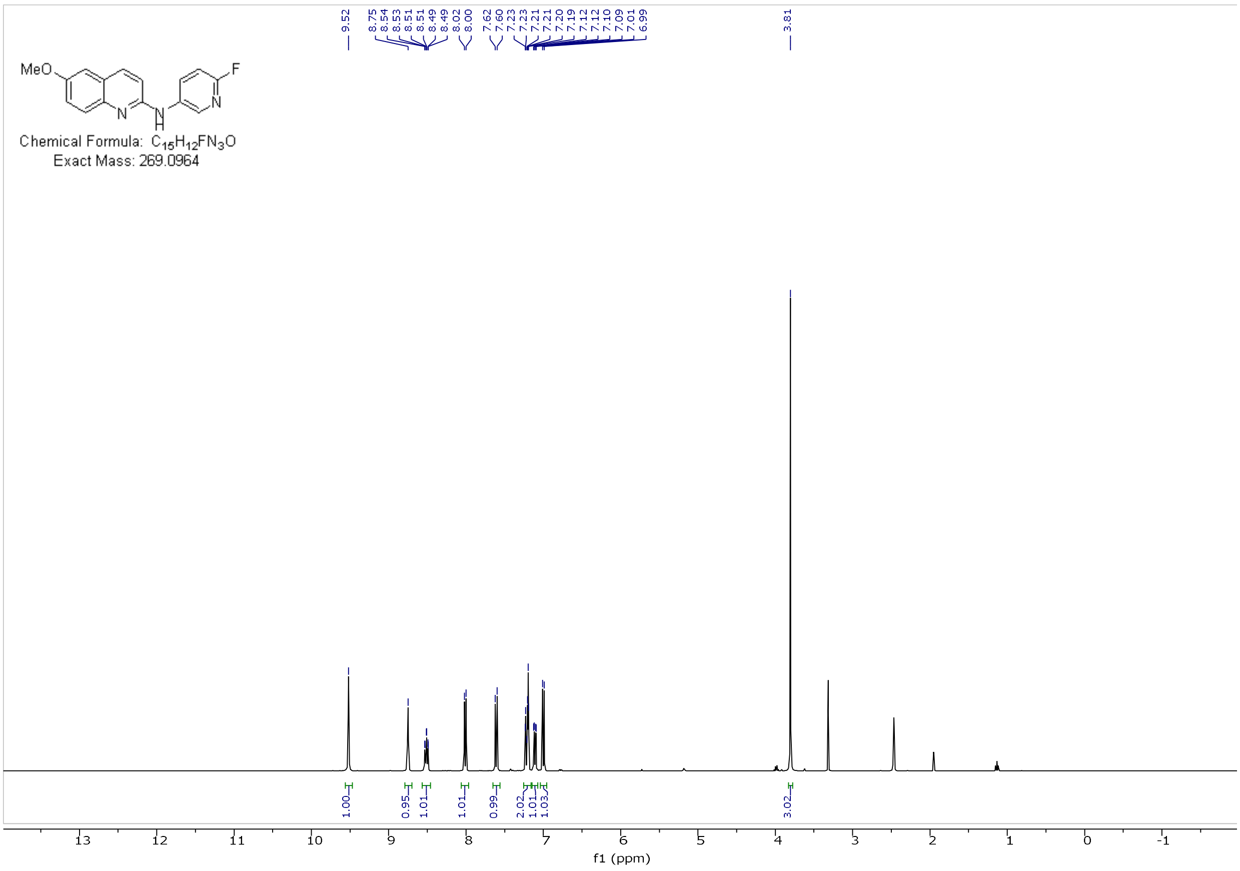

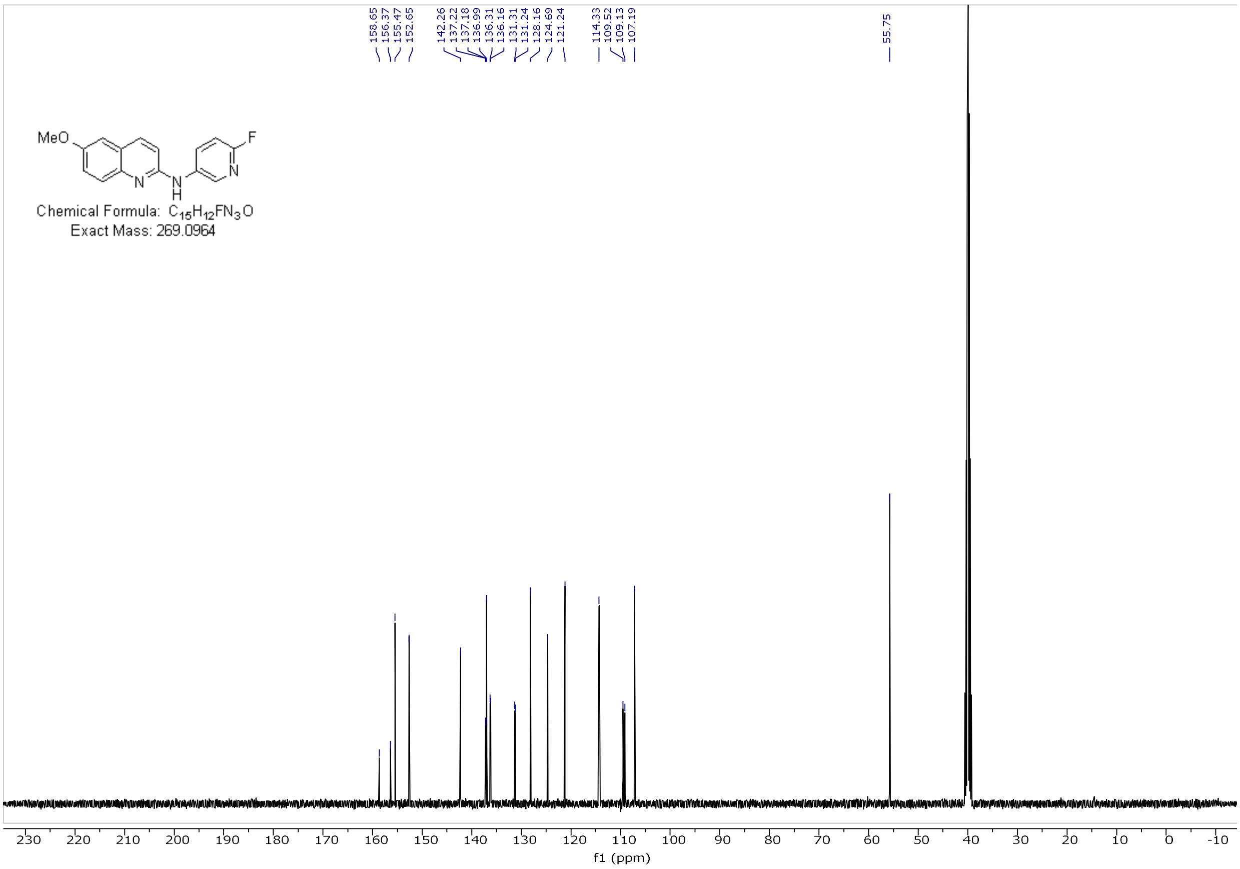

6-Methoxy-*N*-(6-(trifluoromethyl)pyridin-3-yl)quinolin-2-amine (7o, TZ80-3).

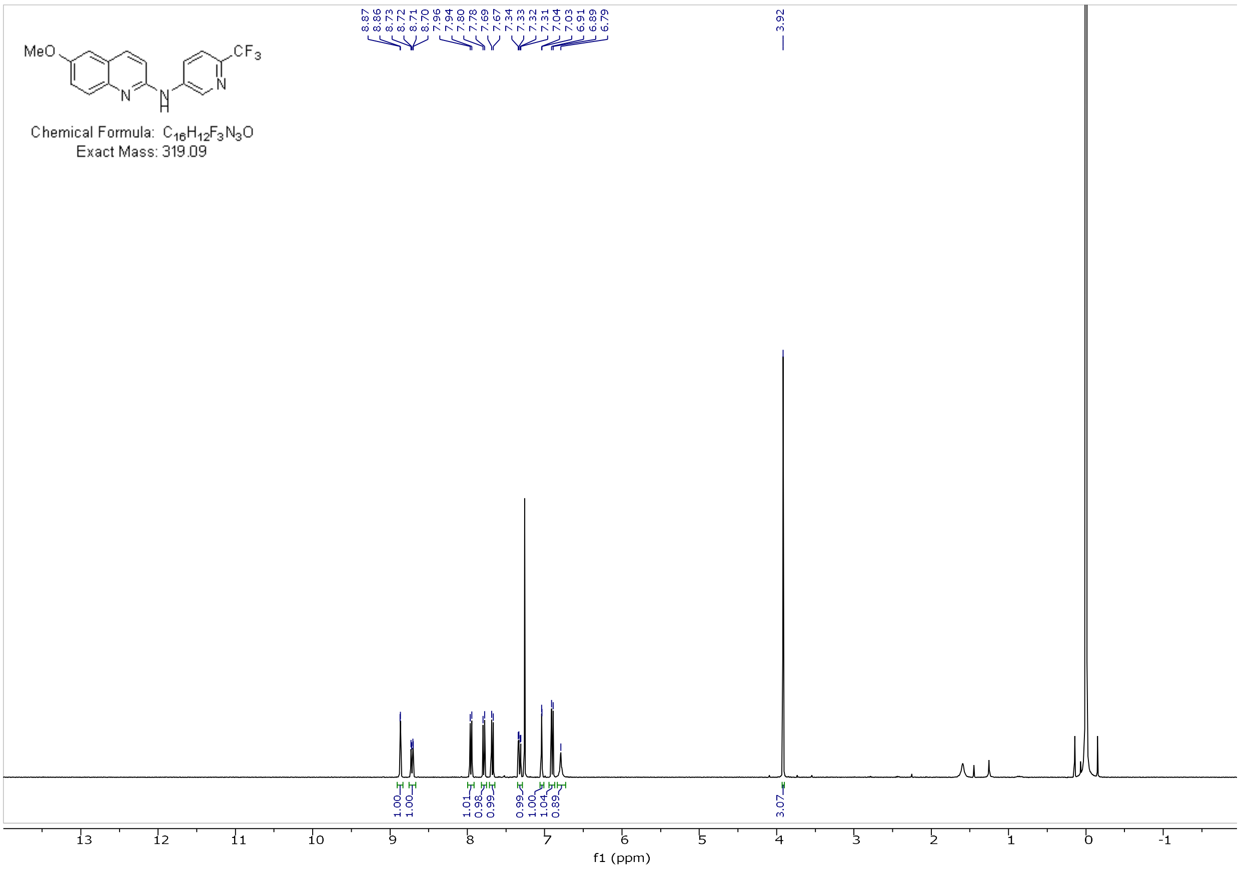

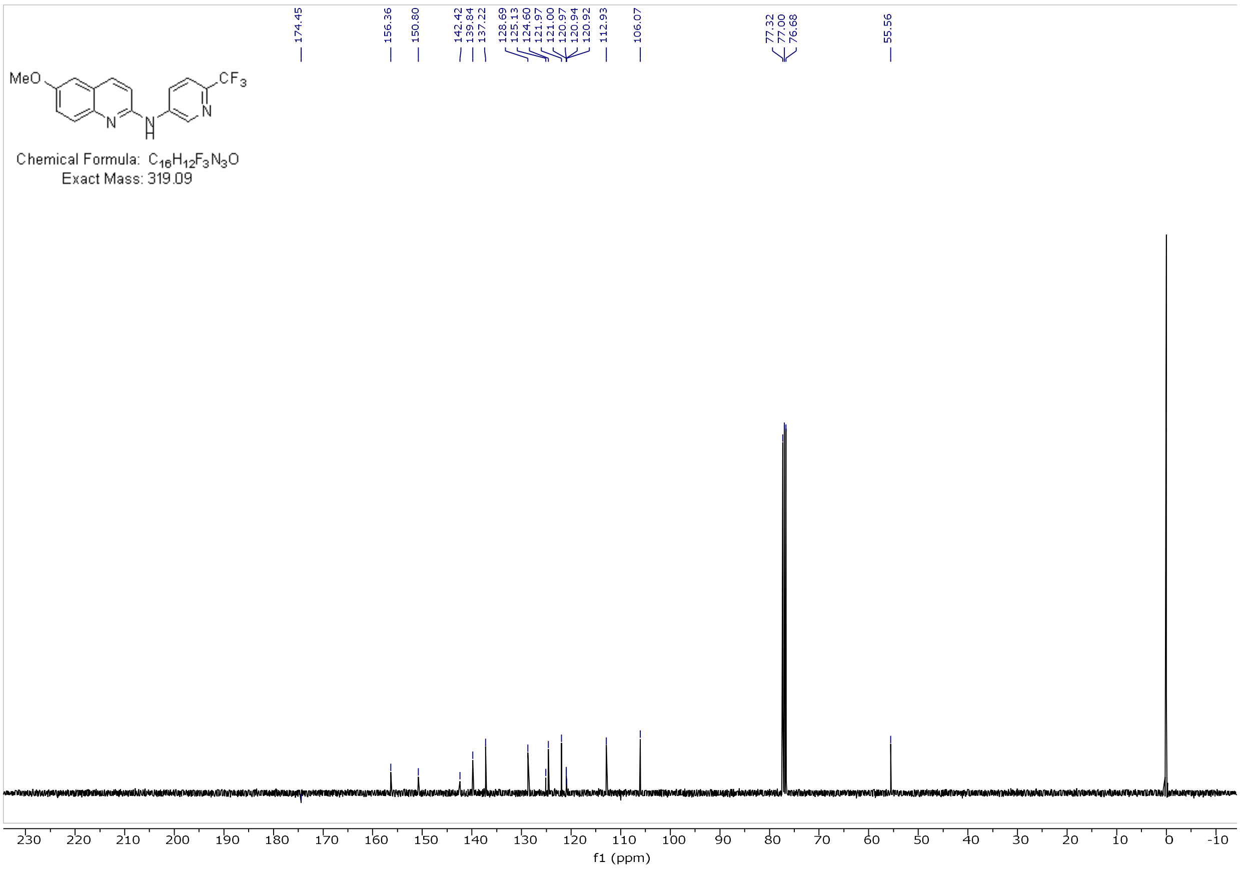

6-(2-Fluoroethoxy)-*N*-(6-(trifluoromethyl)pyridin-3-yl)quinolin-2-amine (7p, TZ80-4).

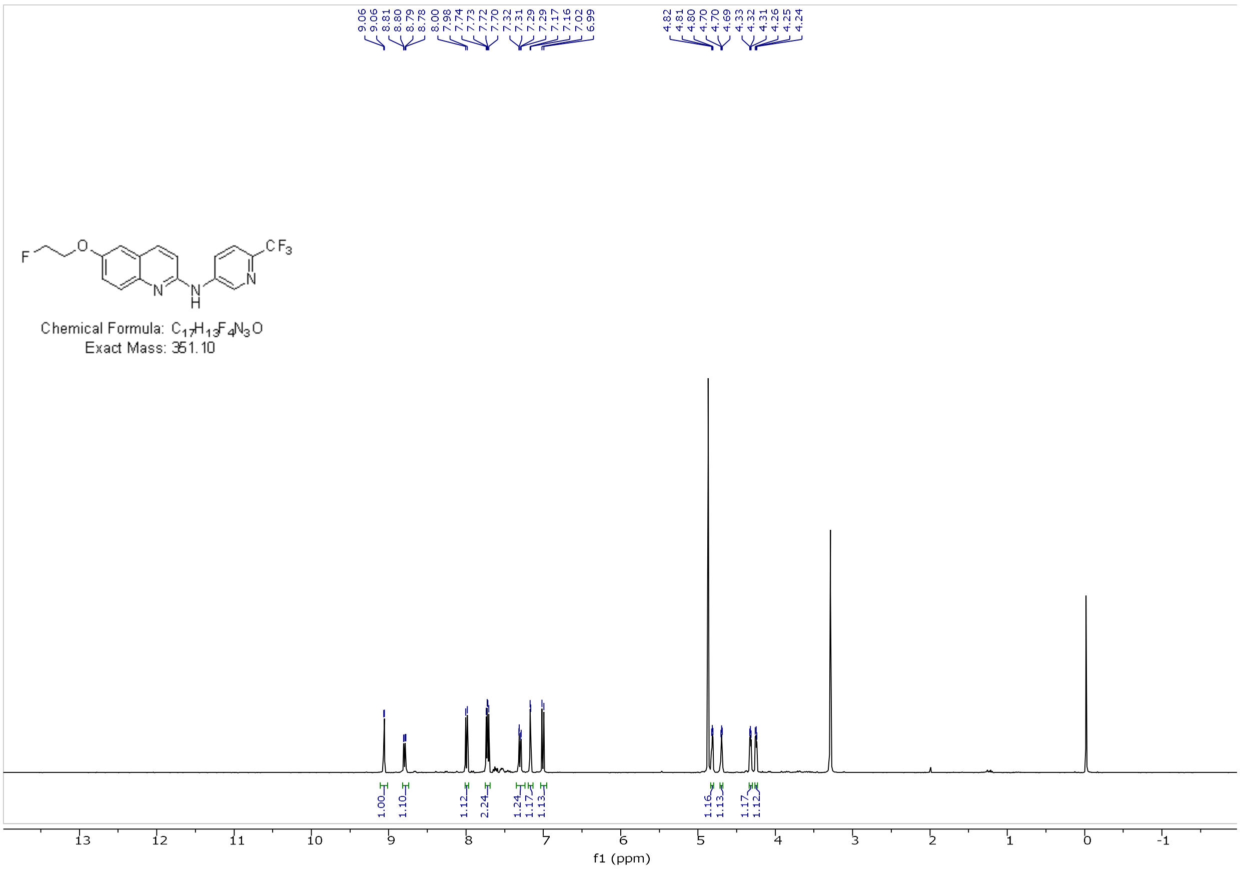

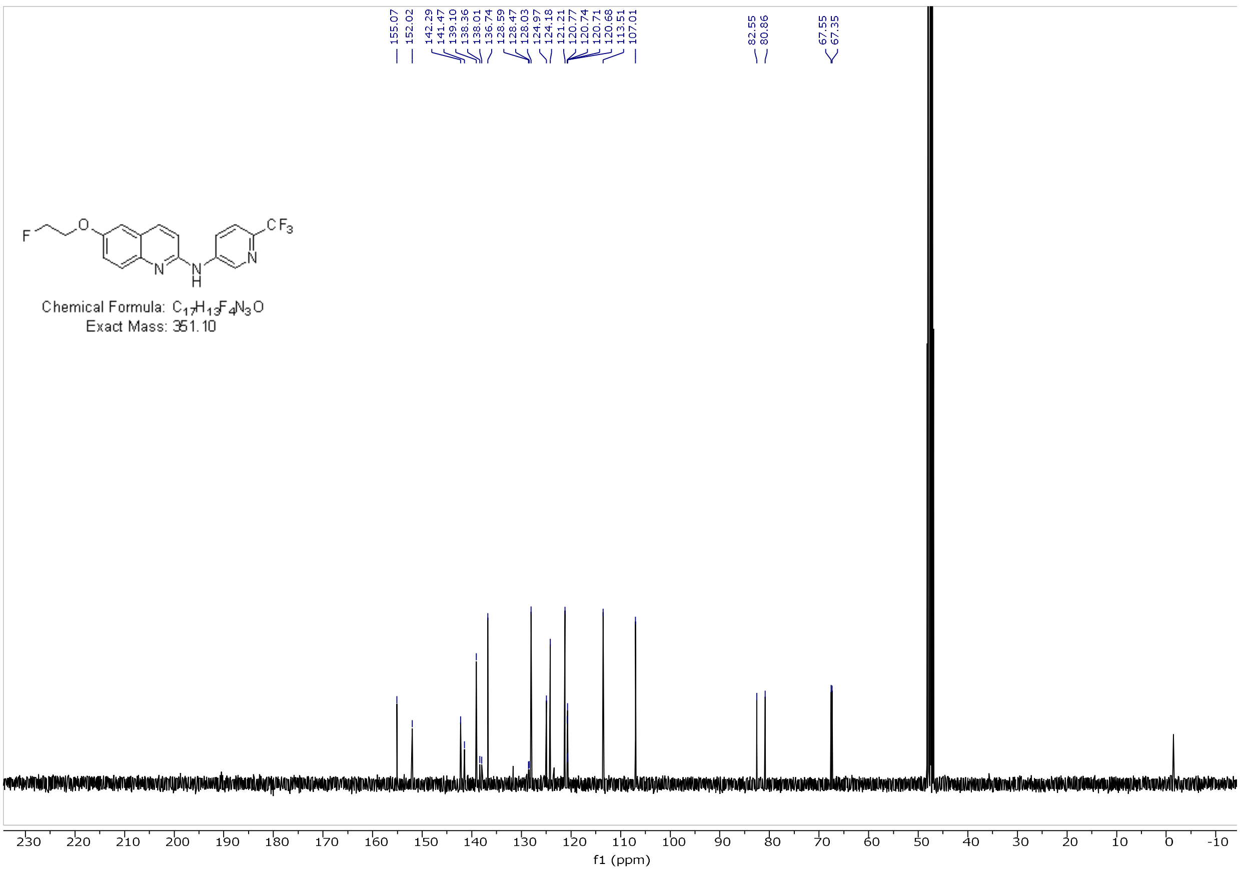

6-methoxy-N-(6-(trifluoromethoxy)pyridin-3-yl)quinolin-2-amine (7q, TZ90-5).

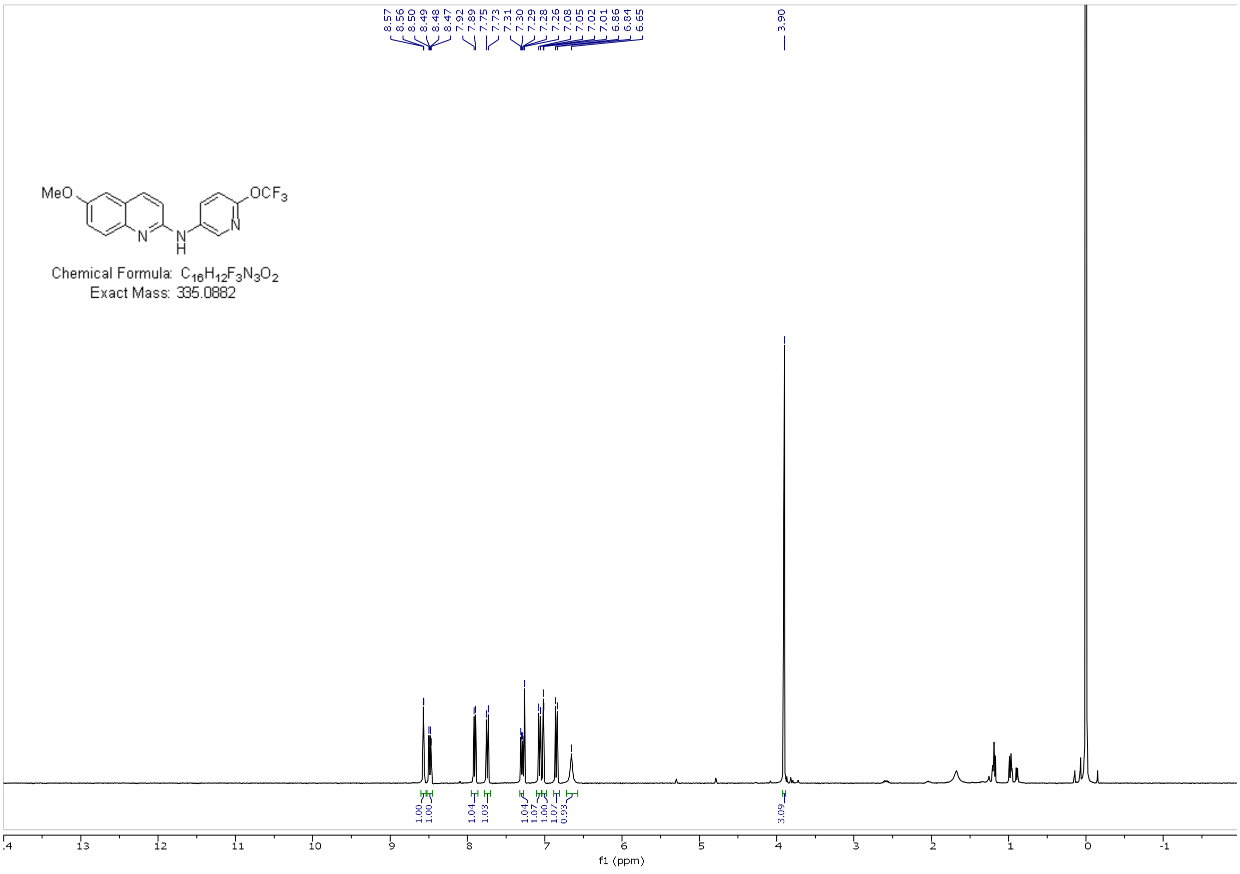

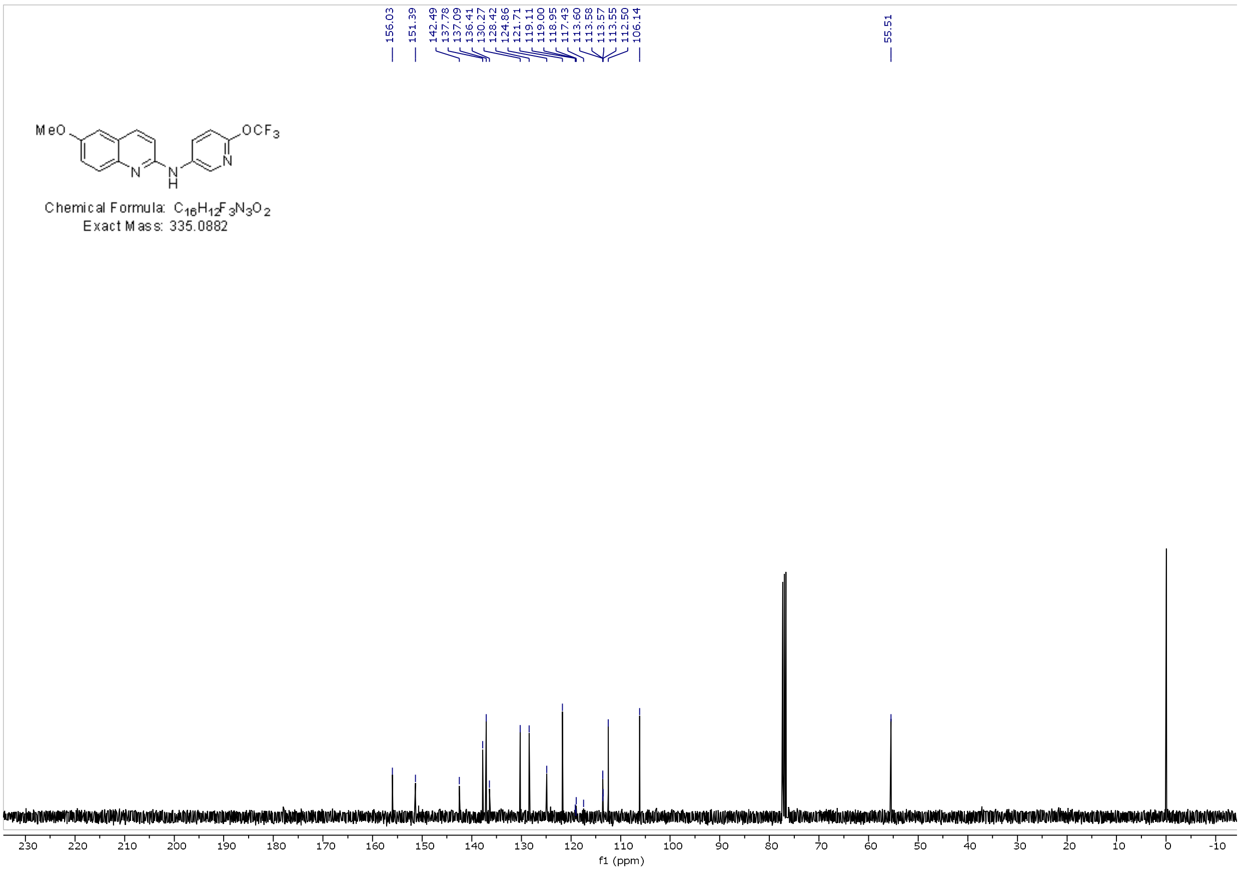

6-Methoxy-*N*-(6-(methylthio)pyridin-3-yl)quinolin-2-amine (7r, TZ80-98).

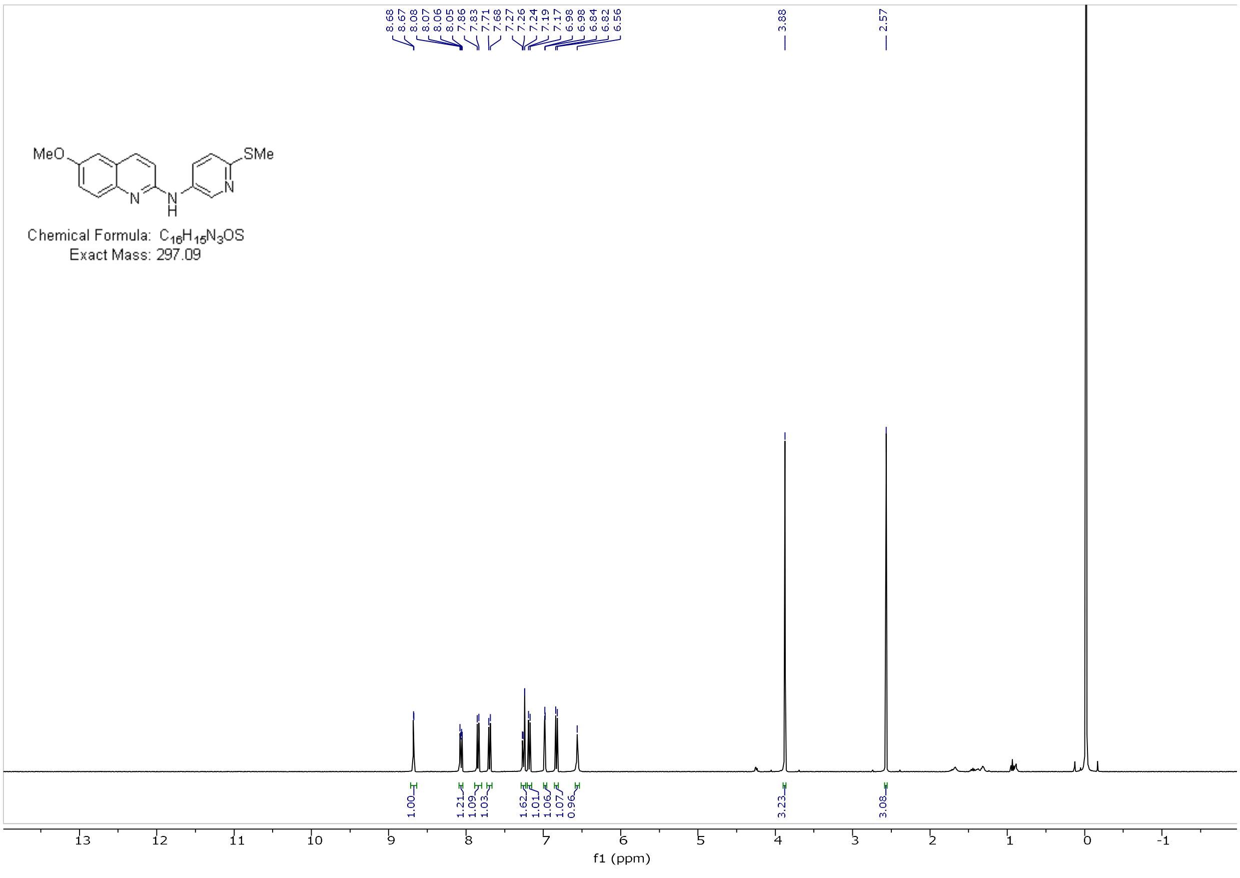

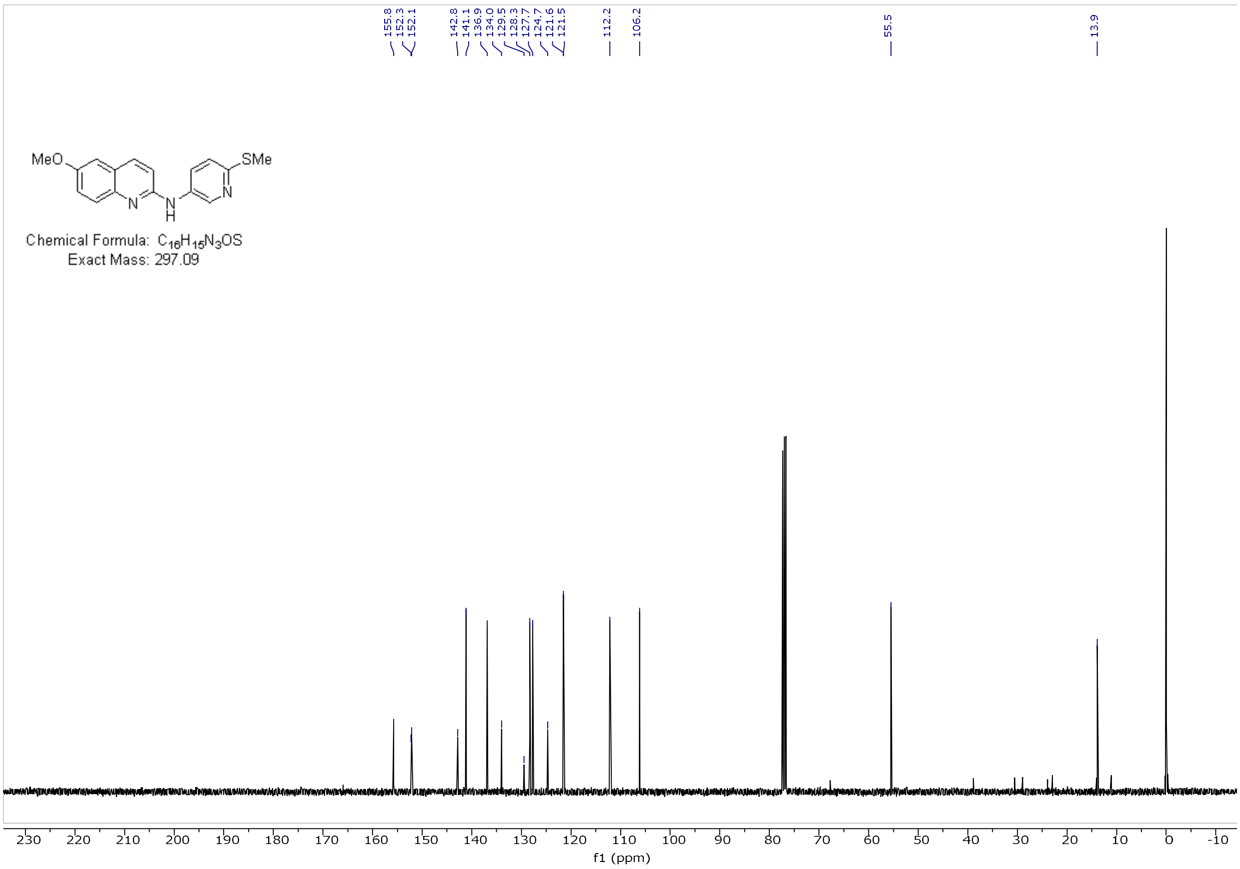

6-(2-Fluoroethoxy)-*N*-(6-methylpyridin-3-yl)quinolin-2-amine (7s, TZ80-55).

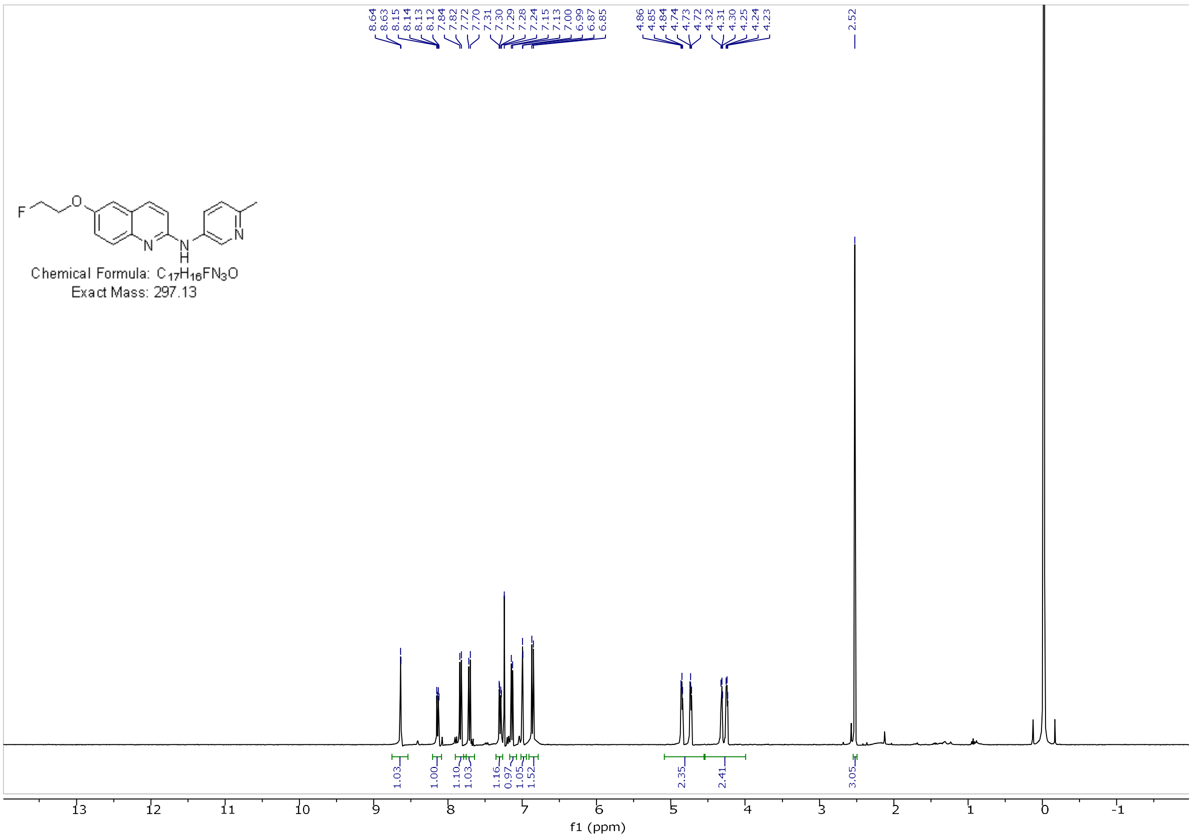

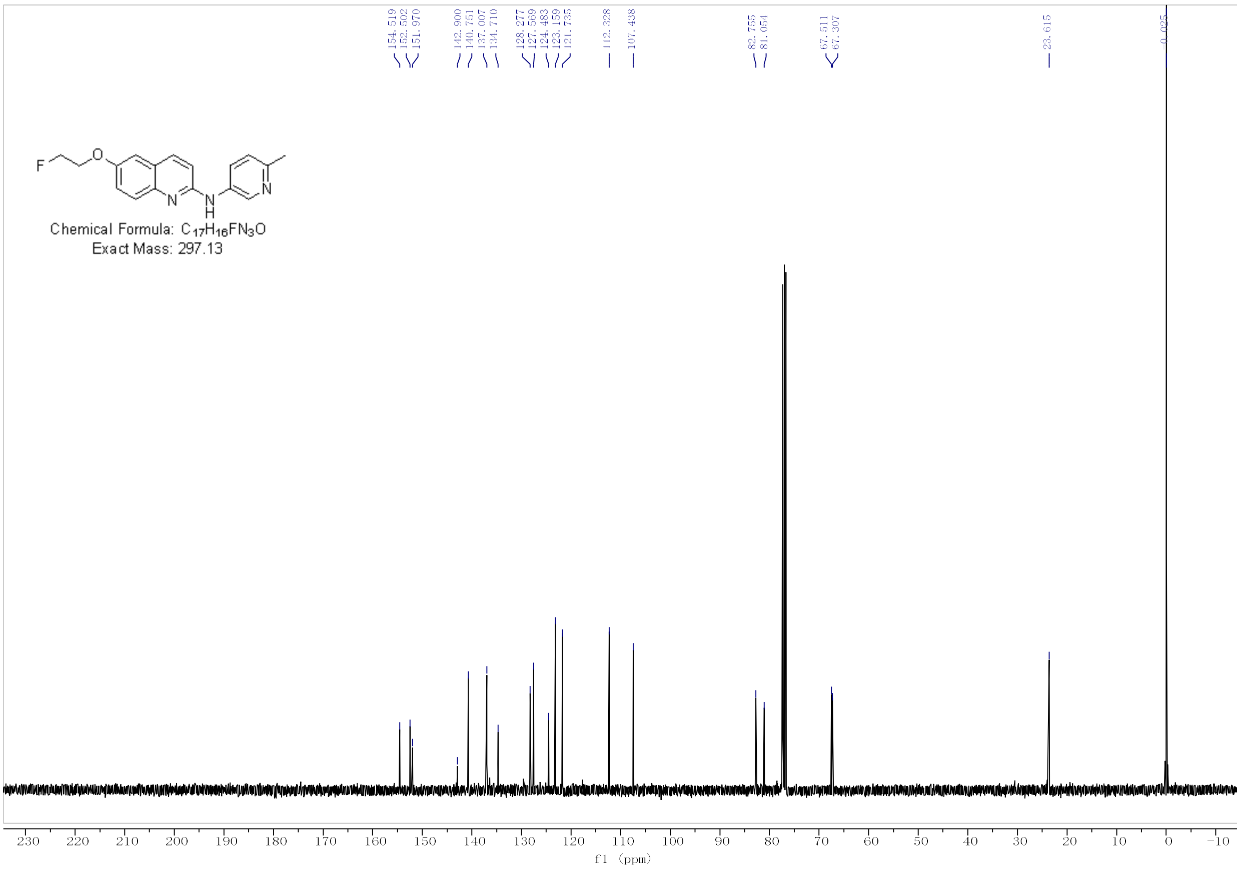

6-(2-Fluoroethoxy)-*N*-(pyridin-3-yl)quinolin-2-amine (7t, TZ80-53).

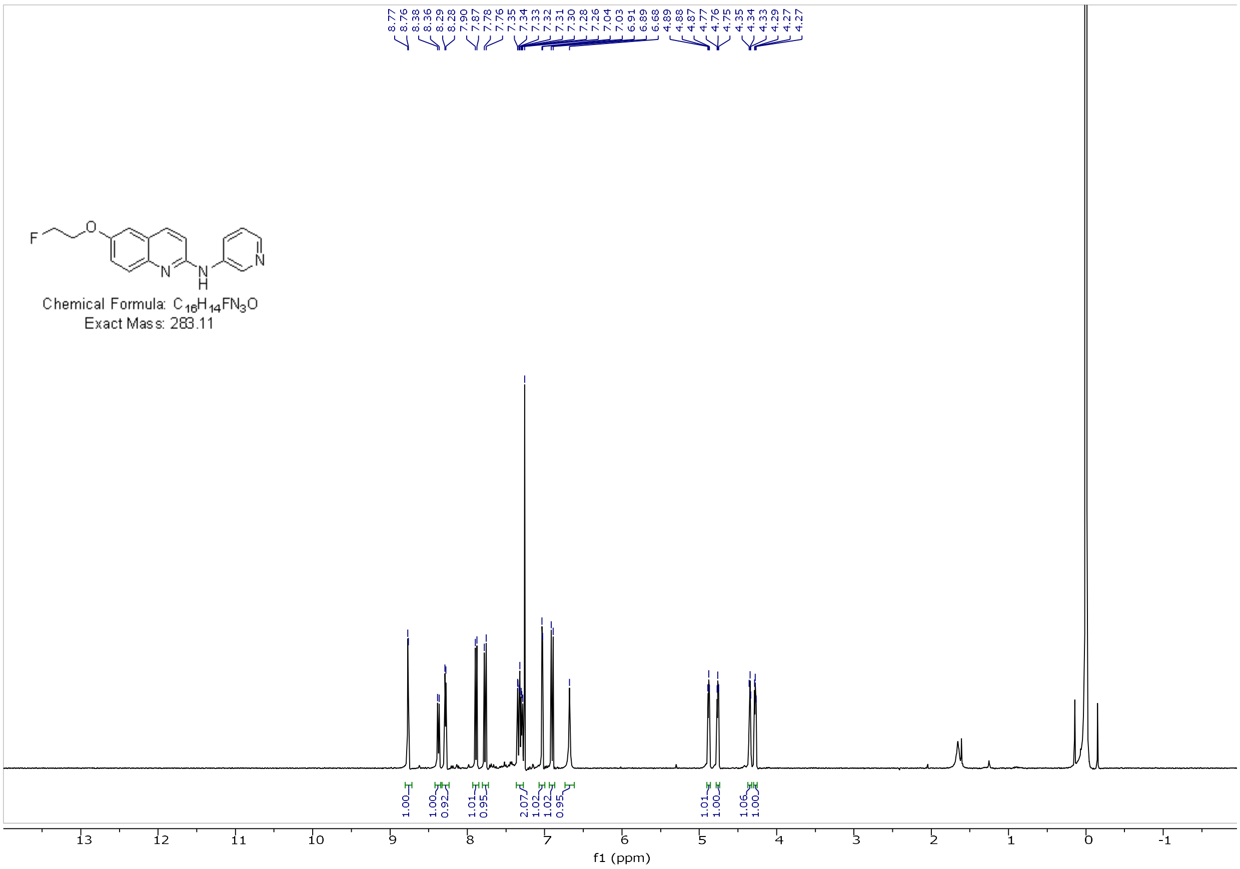

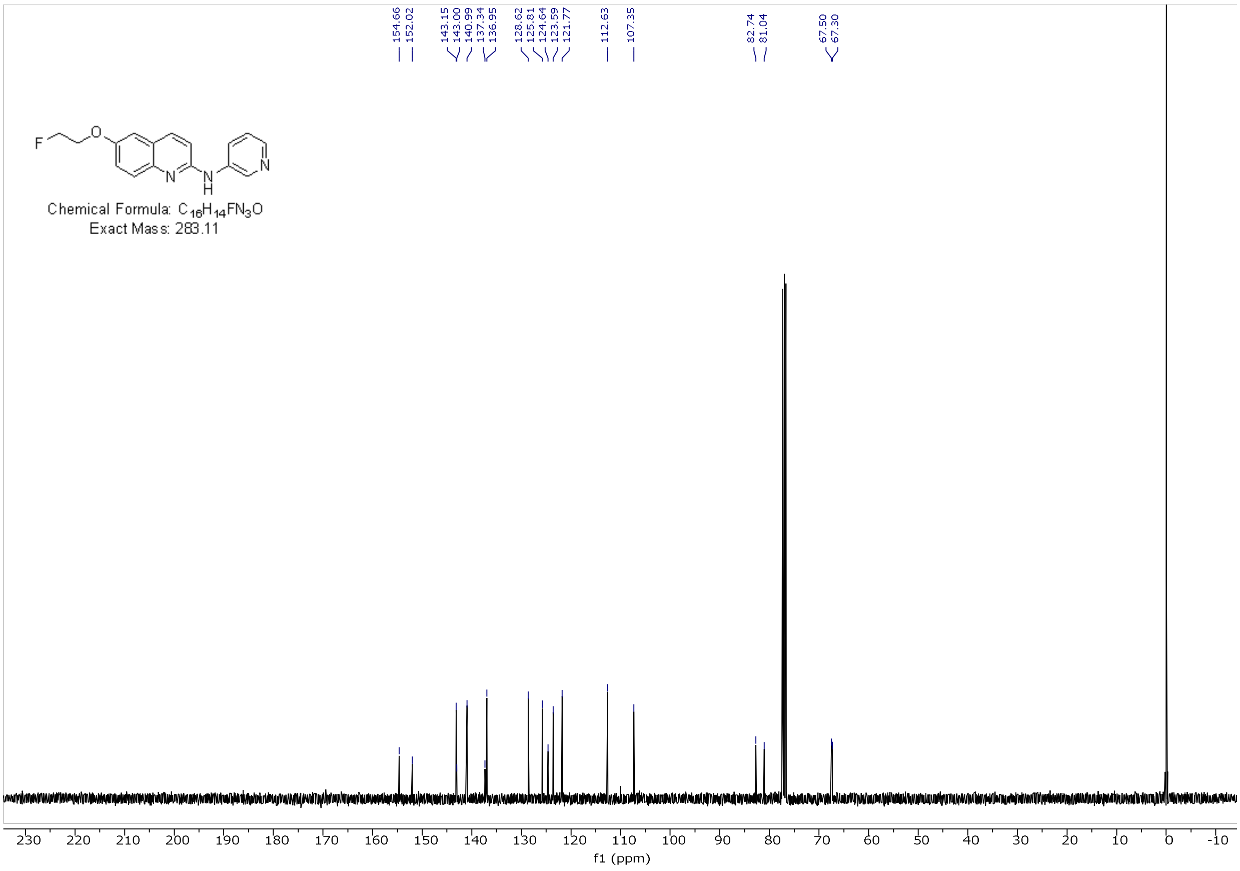

***N*-(6-Methoxypyridin-3-yl)-6-(2,2,2-trifluoroethoxy)quinolin-2-amine (7u, TZ80-58).**

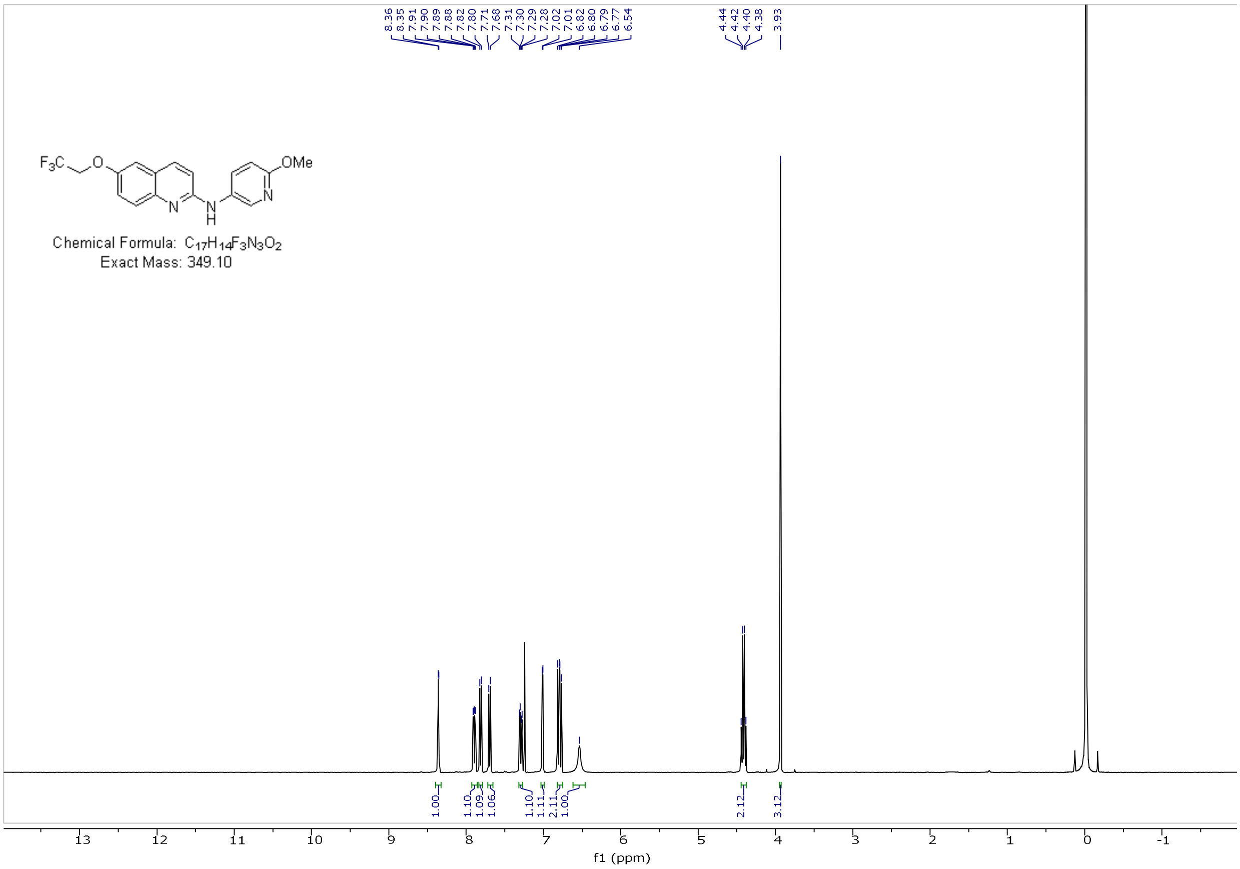

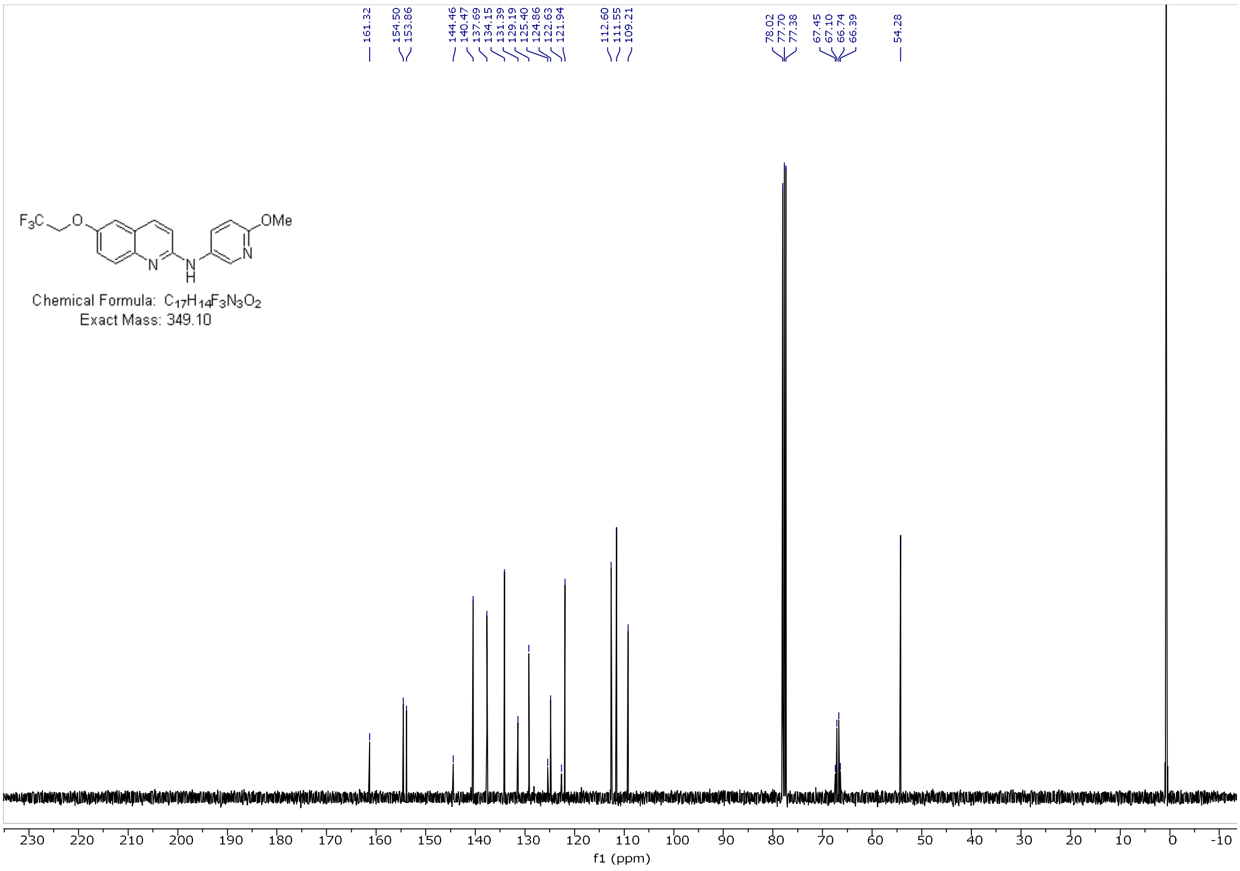

(*E*)-3-(tributylstannyl)prop-2-en-1-ol (7v, TZ61-83).

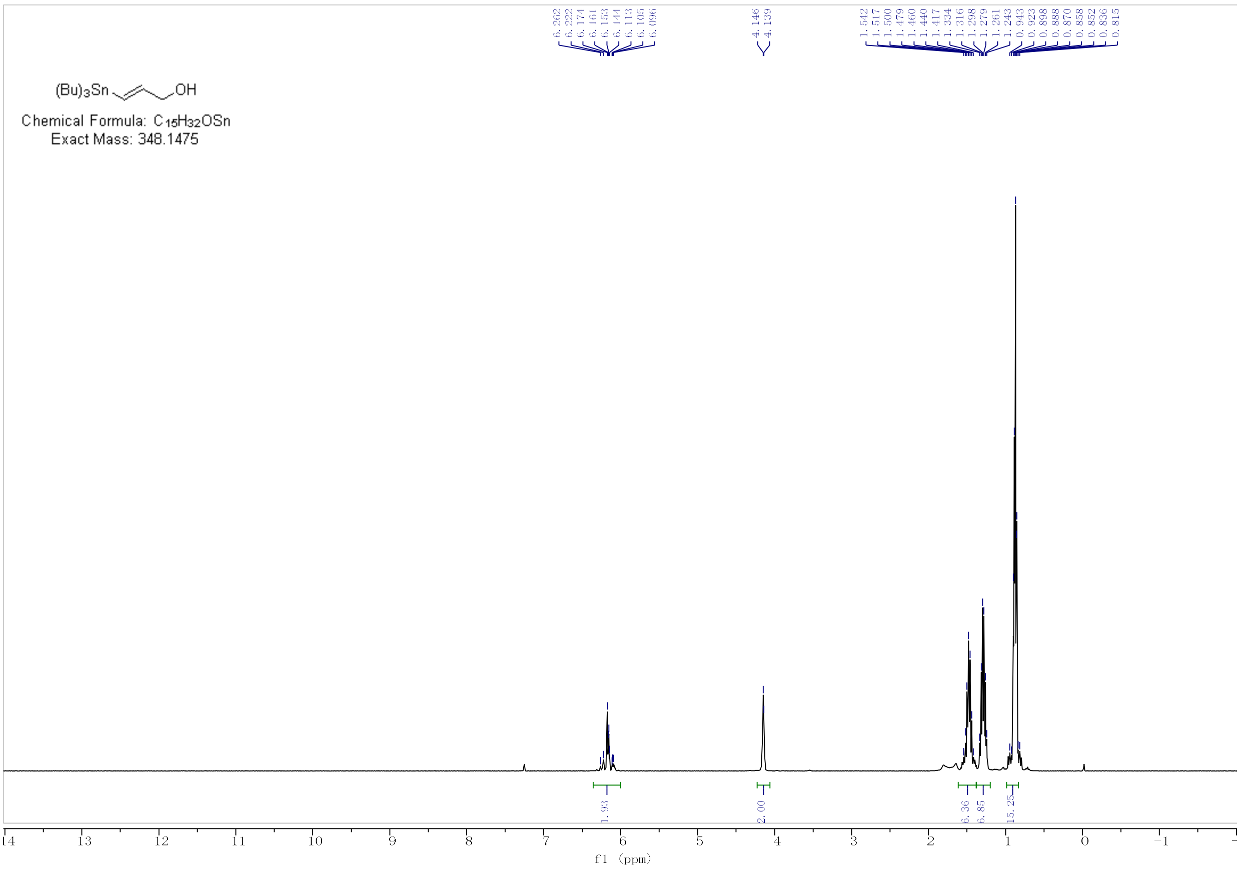

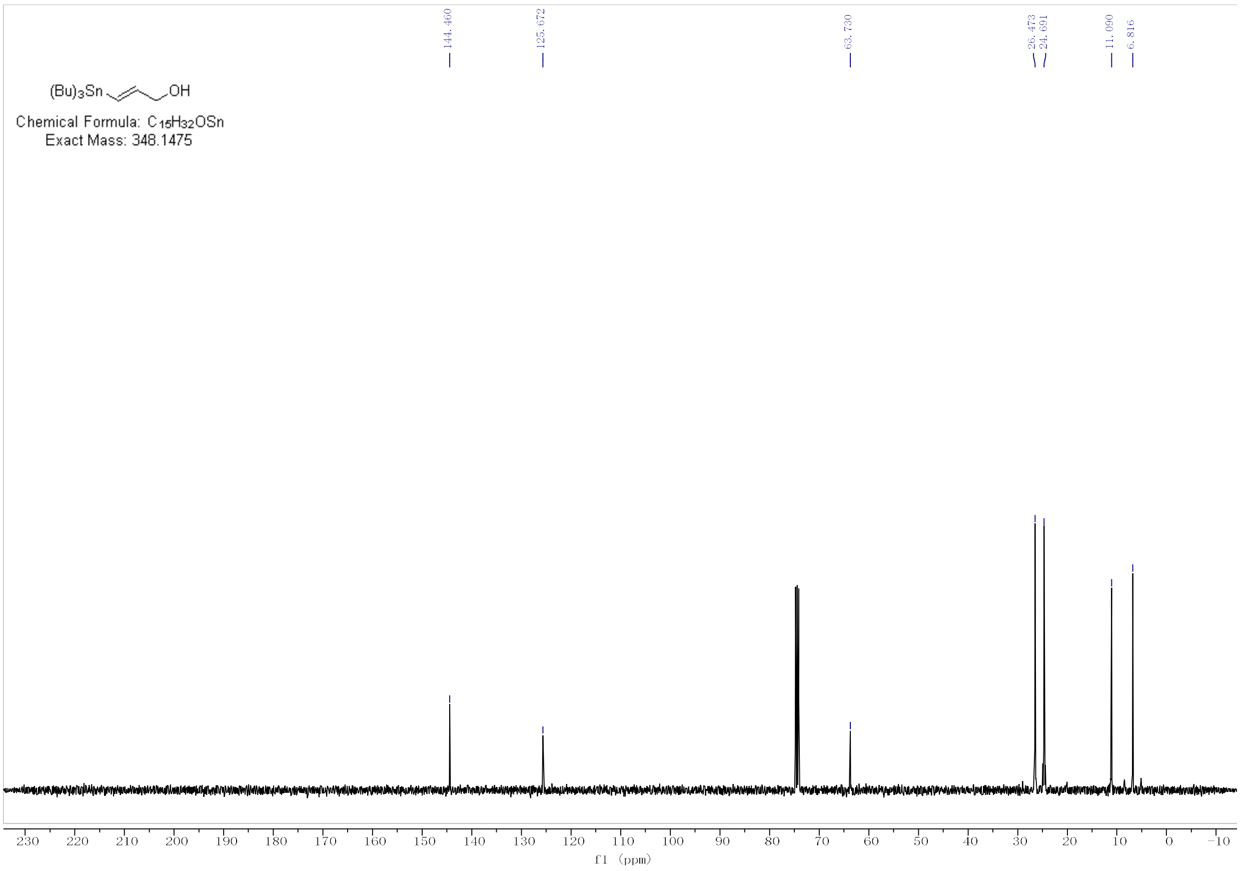

(*E*)-(3-bromoprop-1-en-1-yl)tributylstannane (7w, TZ61-79).

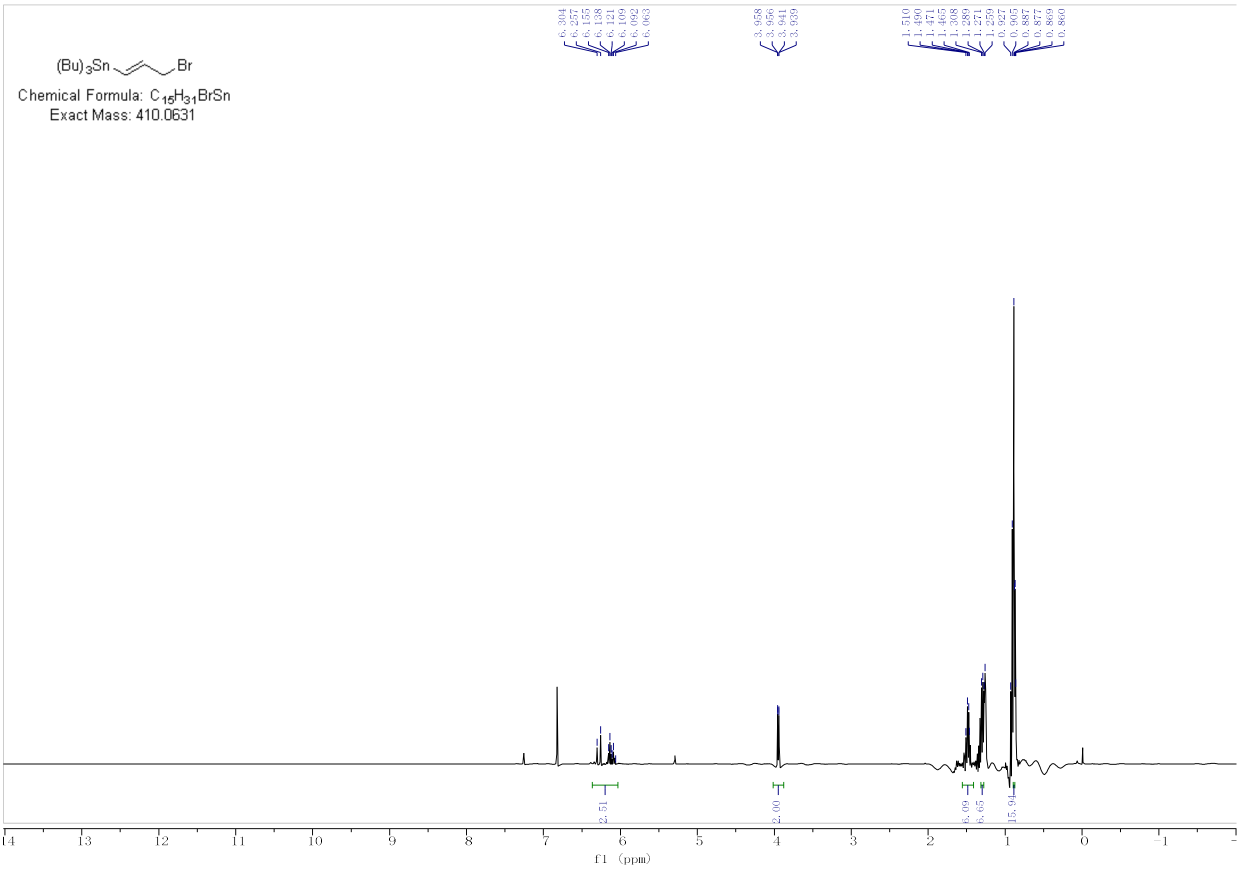

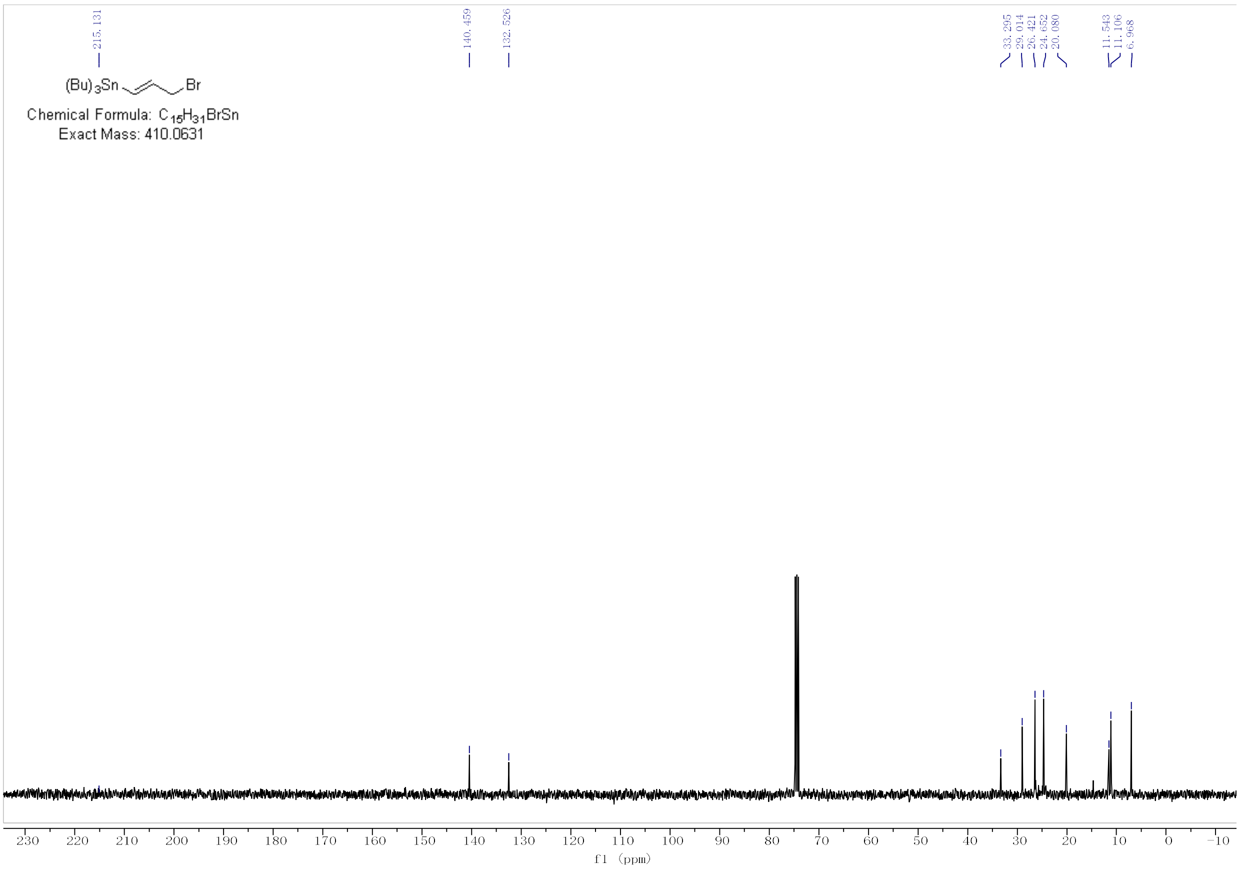

(*E*)-N-(6-methoxypyridin-3-yl)-6-((3-(tributylstannyl)allyl)oxy)quinolin-2-amine (7x, TZ61-80).

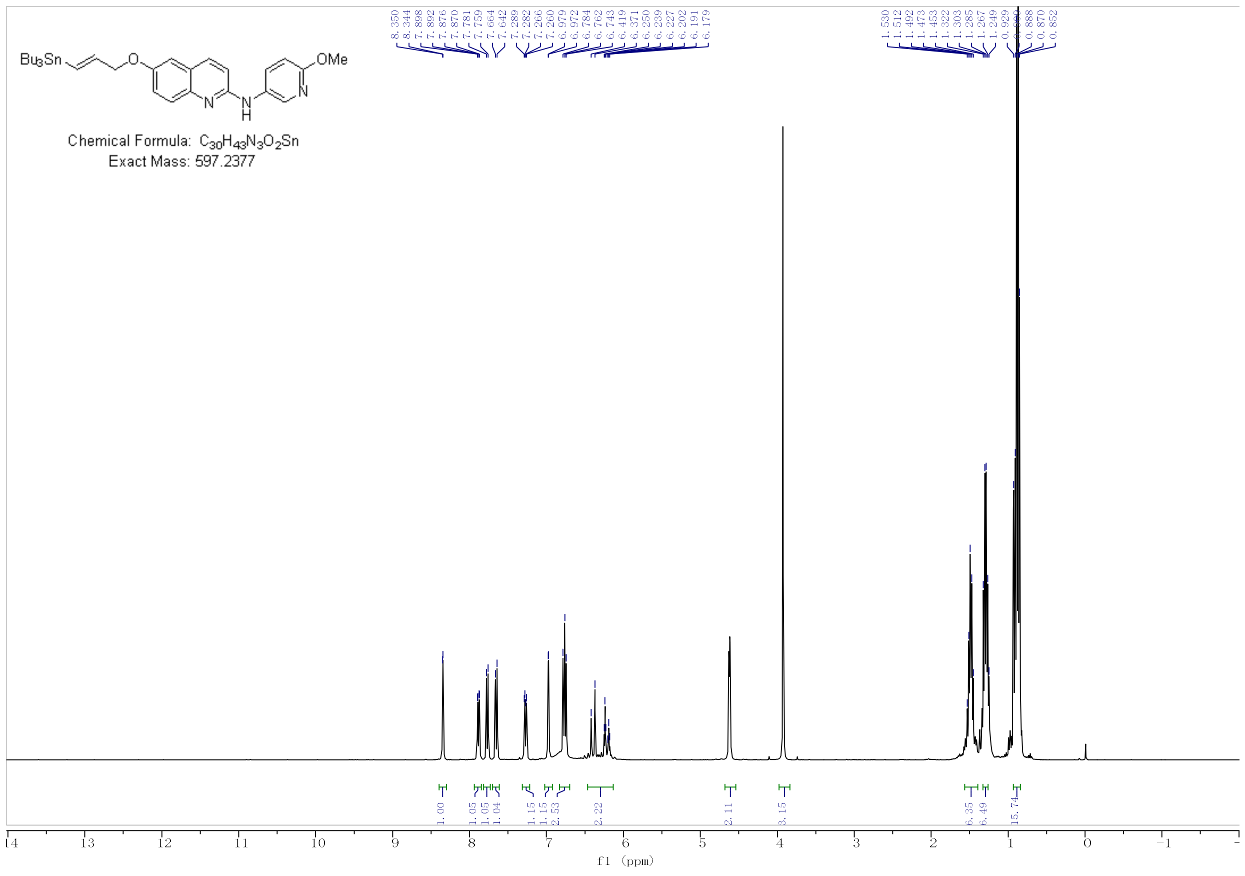

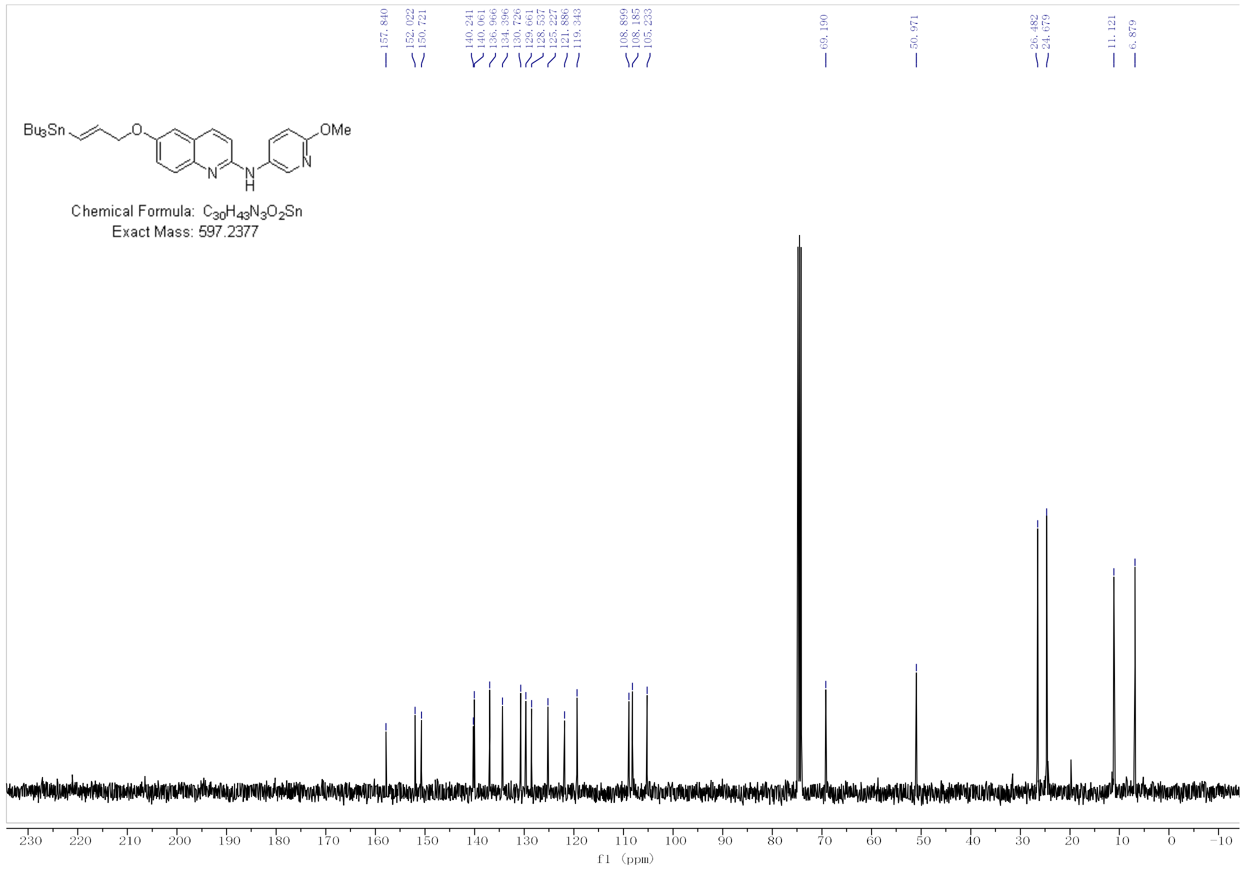

N-(6-Methoxypyridin-3-yl)-6-(oxetan-3-yloxy)quinolin-2-amine (8a, TZ80-54).

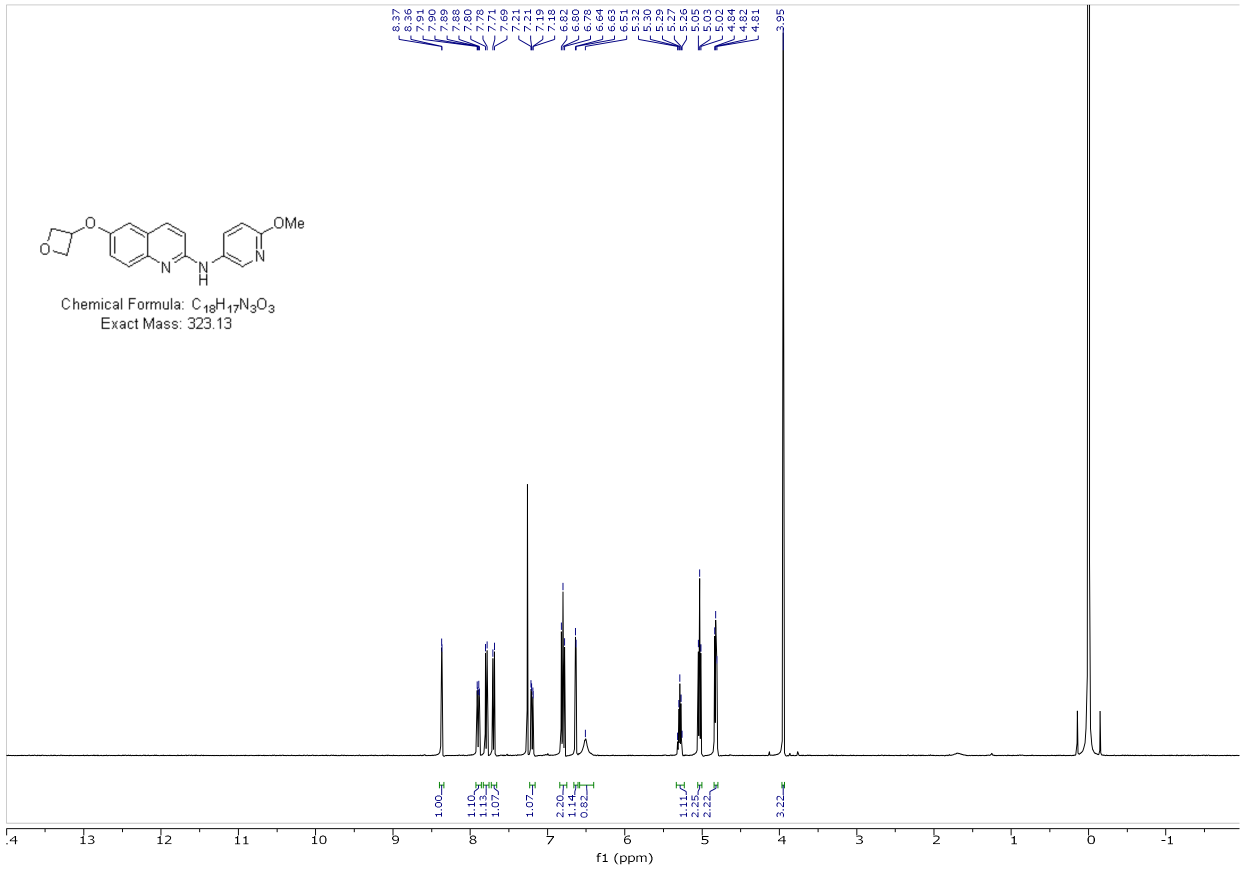

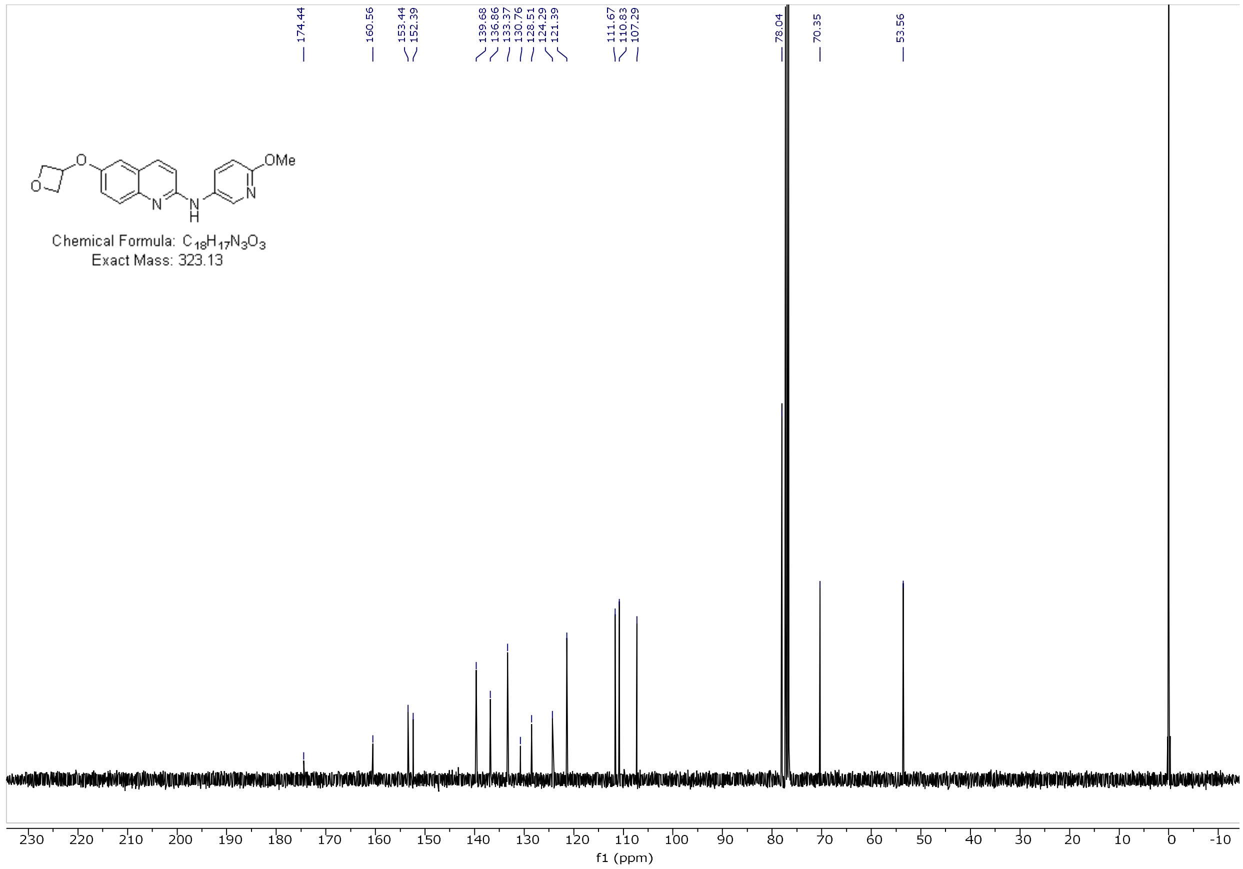

6-Cyclohexyloxy-*N*-(6-methoxypyridin-3-yl)quinolin-2-amine (8b, TZ80-52).

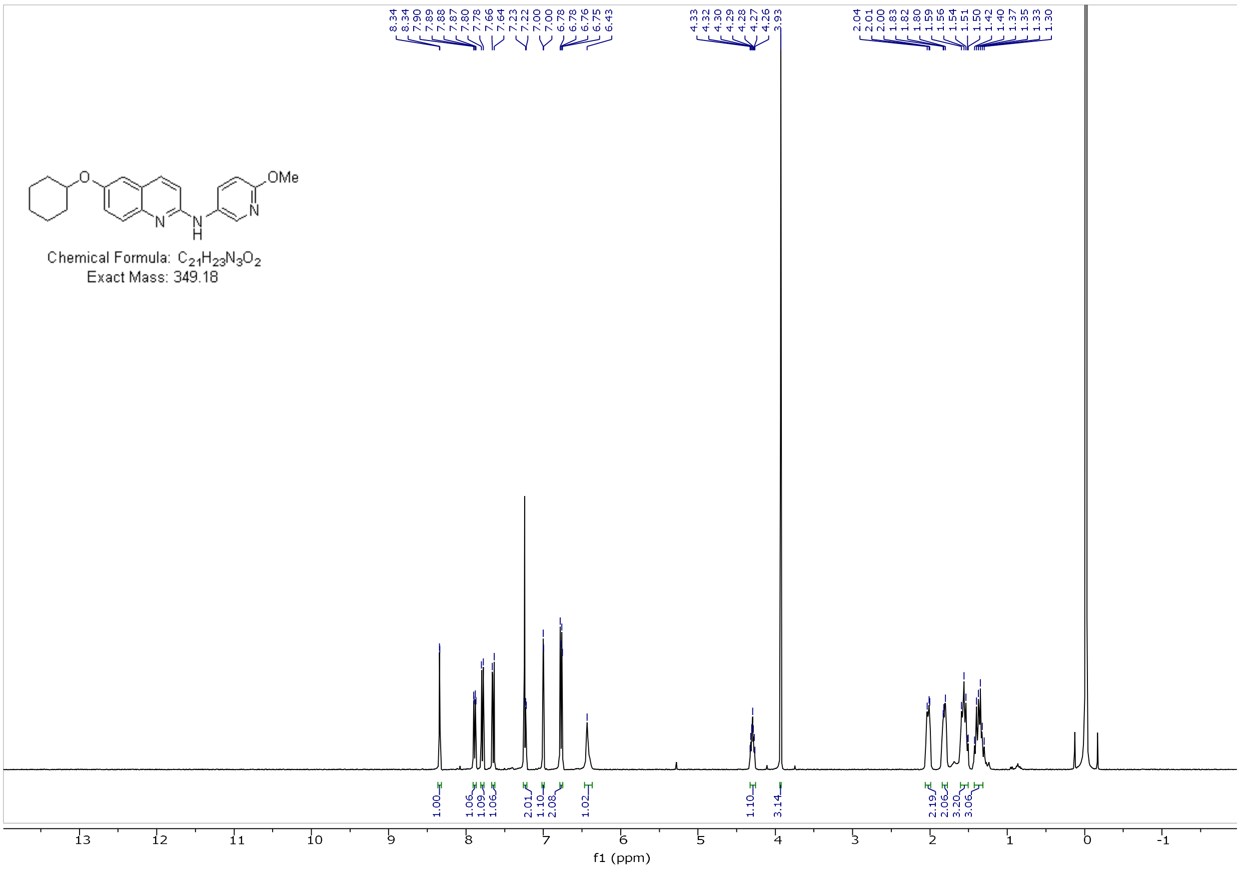

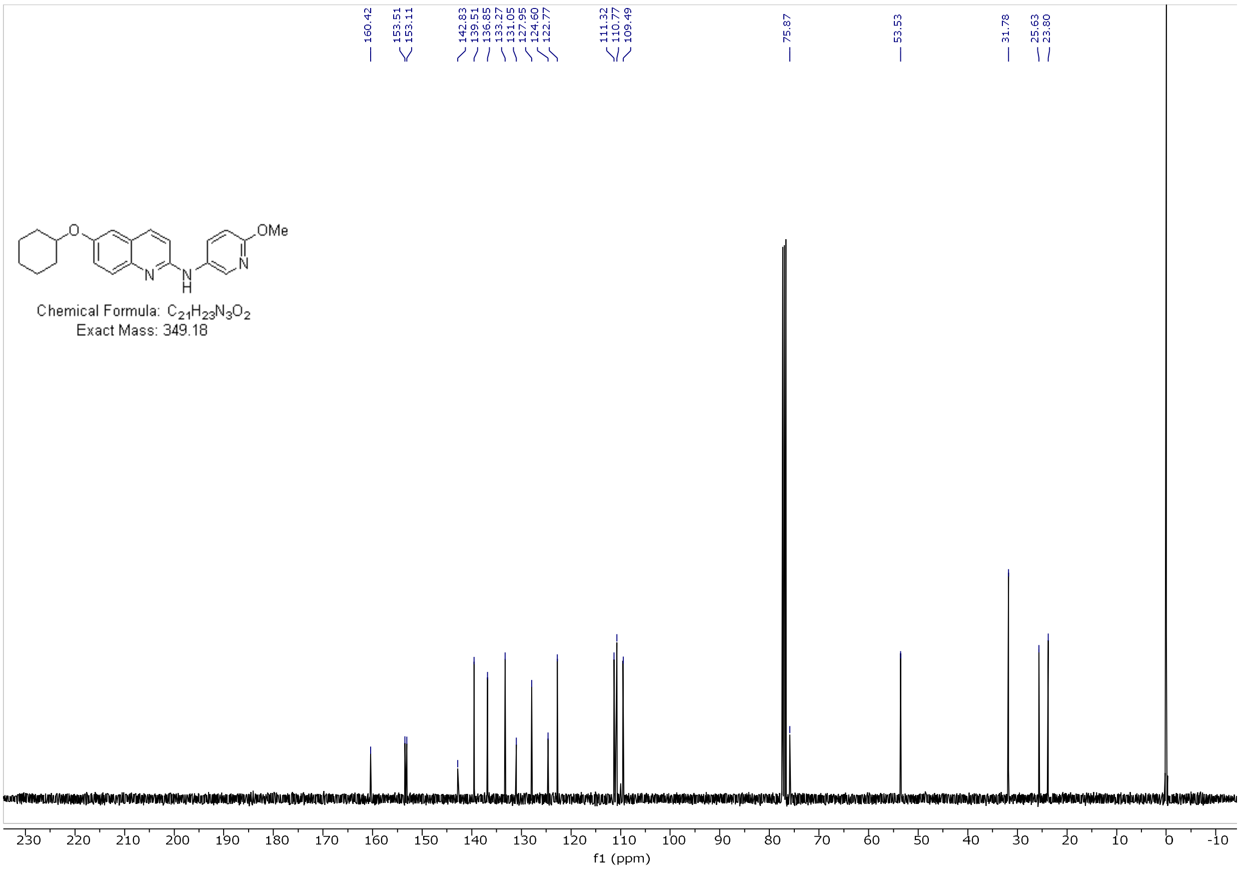

6-(Benzyloxy)-*N*-(6-methoxypyridin-3-yl)quinolin-2-amine (8c, TZ80-40).

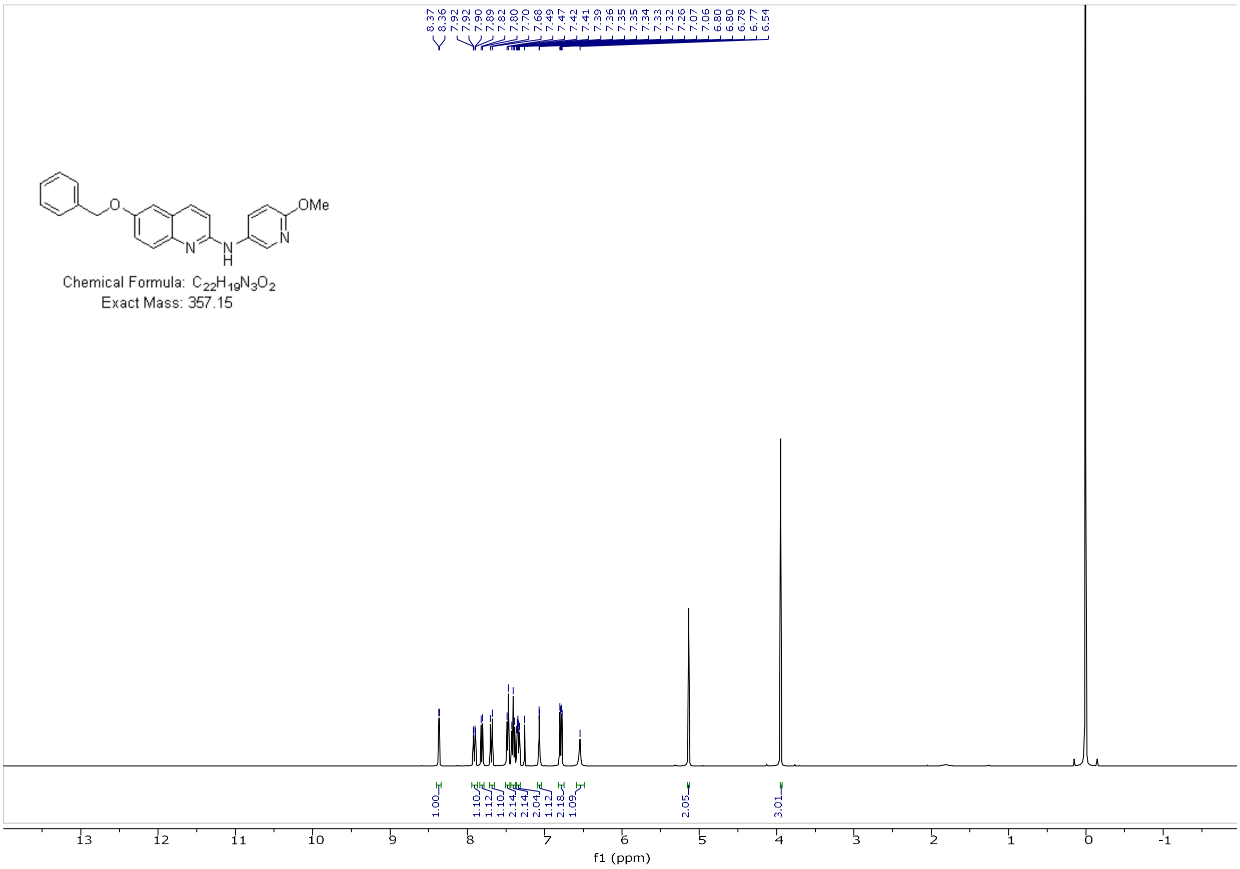

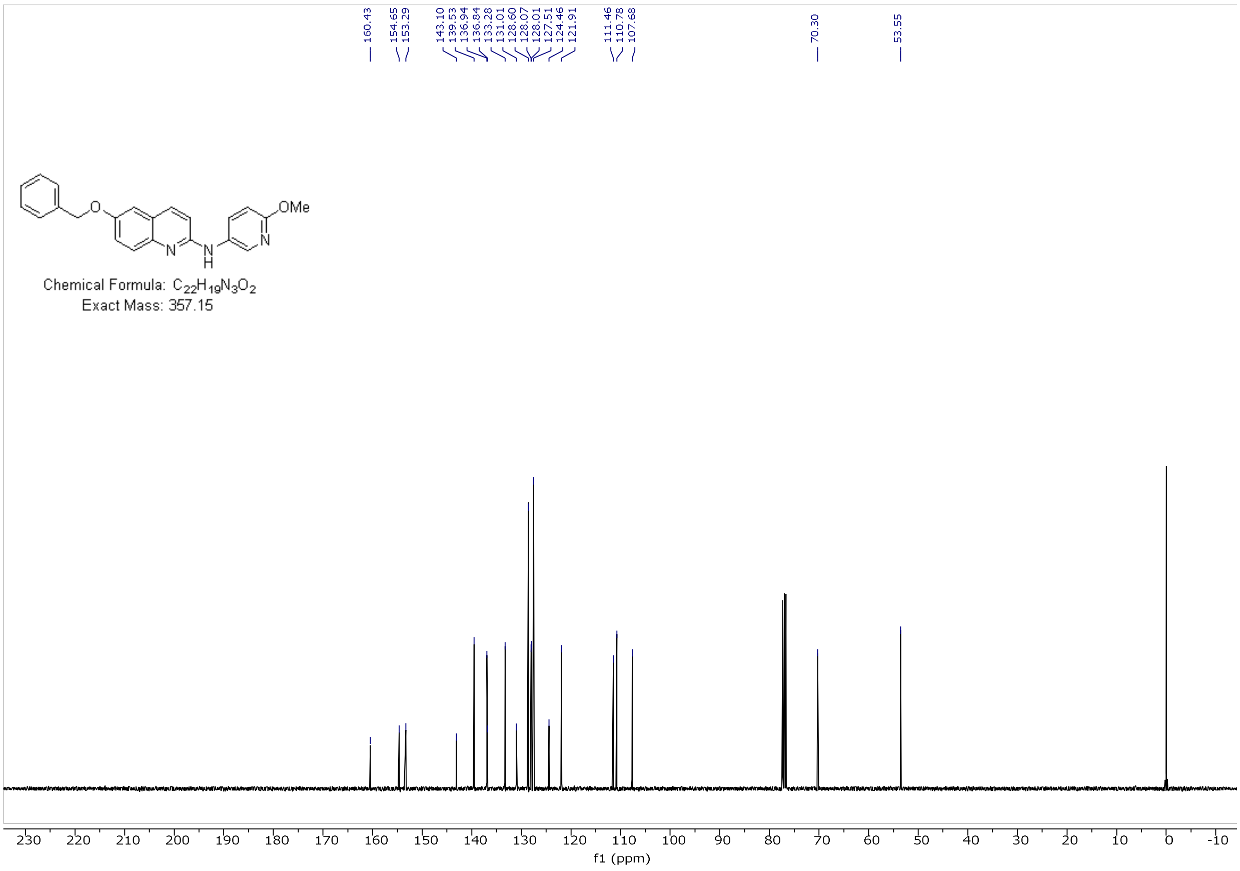

N-(6-Methoxypyridin-3-yl)-6-(pyridin-4-ylmethoxy)quinolin-2-amine (8d, TZ80-42).

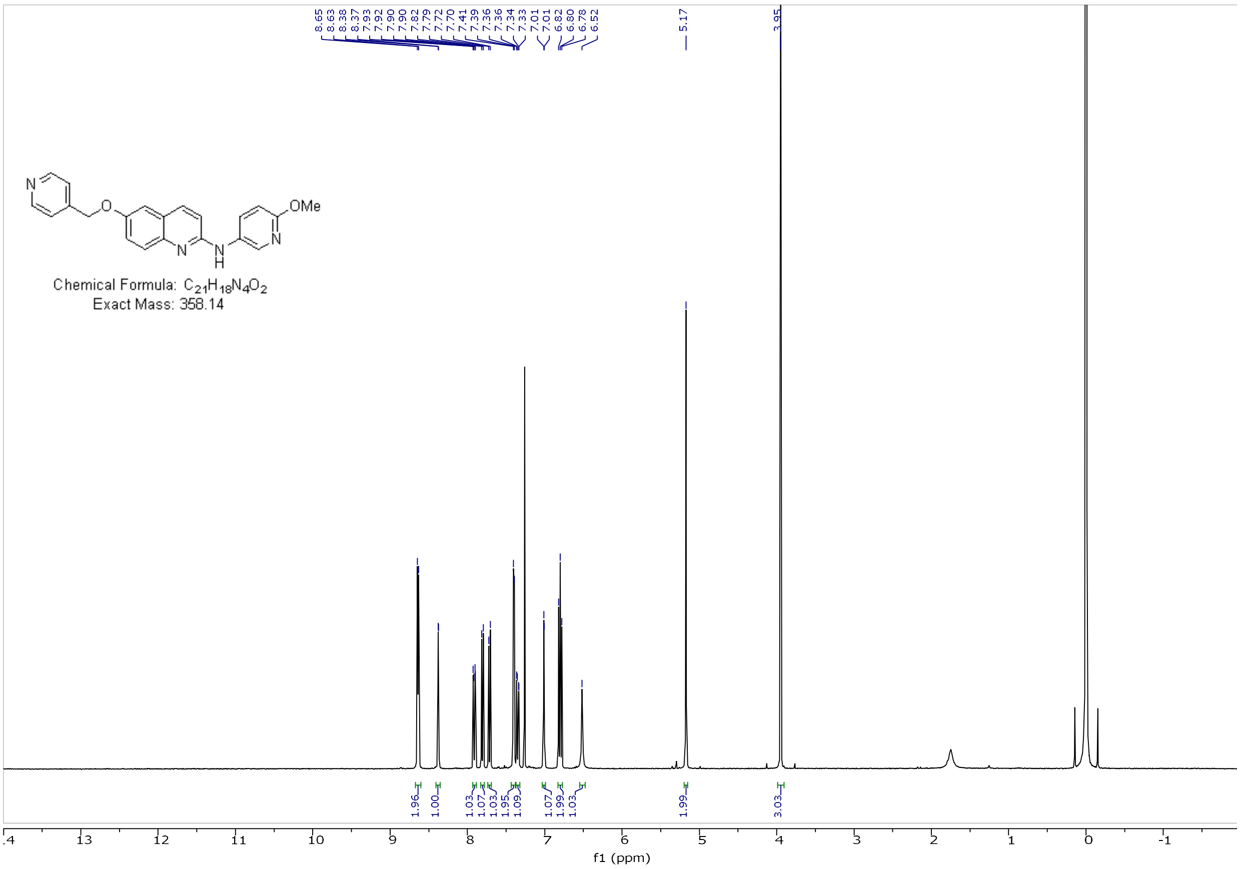

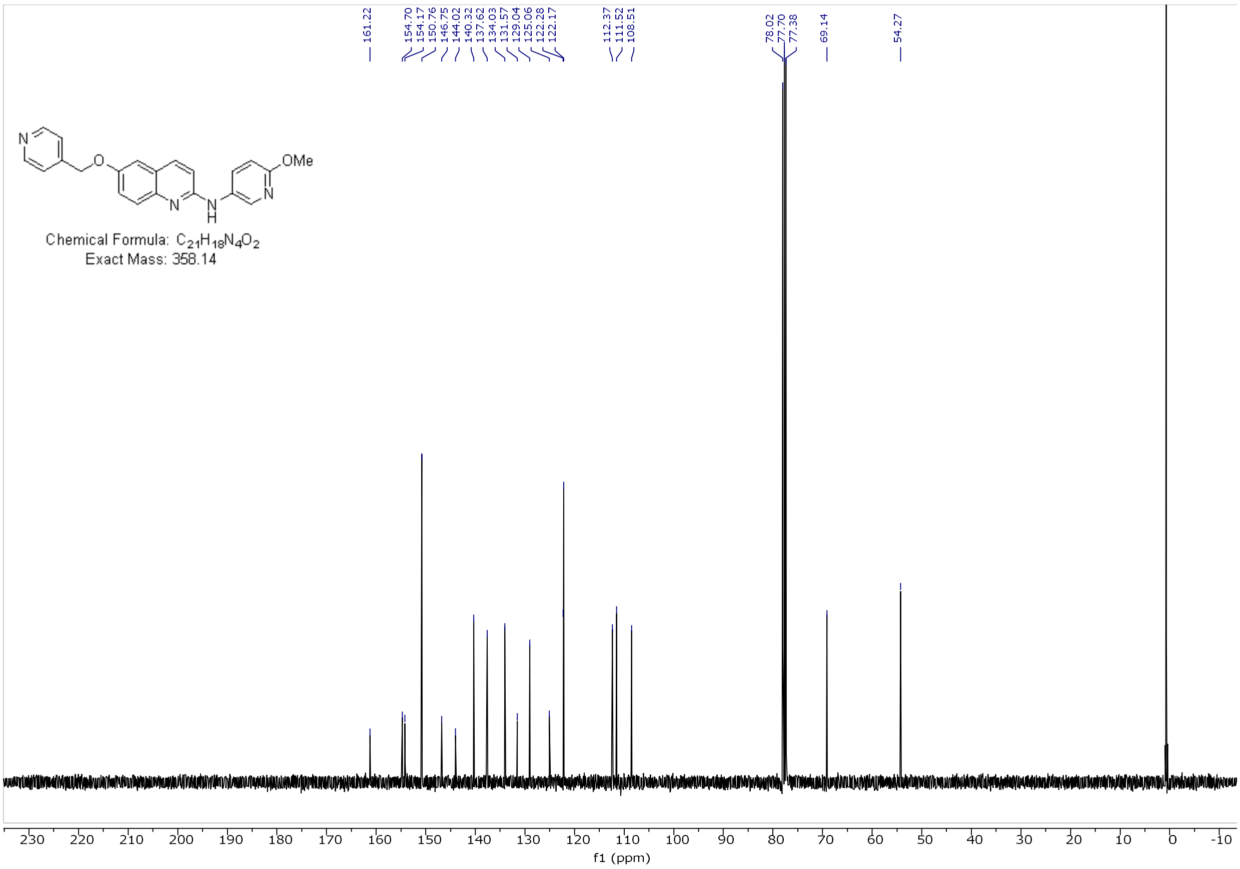

_N_-(6-Methoxypyridin-3-yl)-6-(pyridin-3-ylmethoxy)quinolin-2-amine (8e, TZ80-66-B).

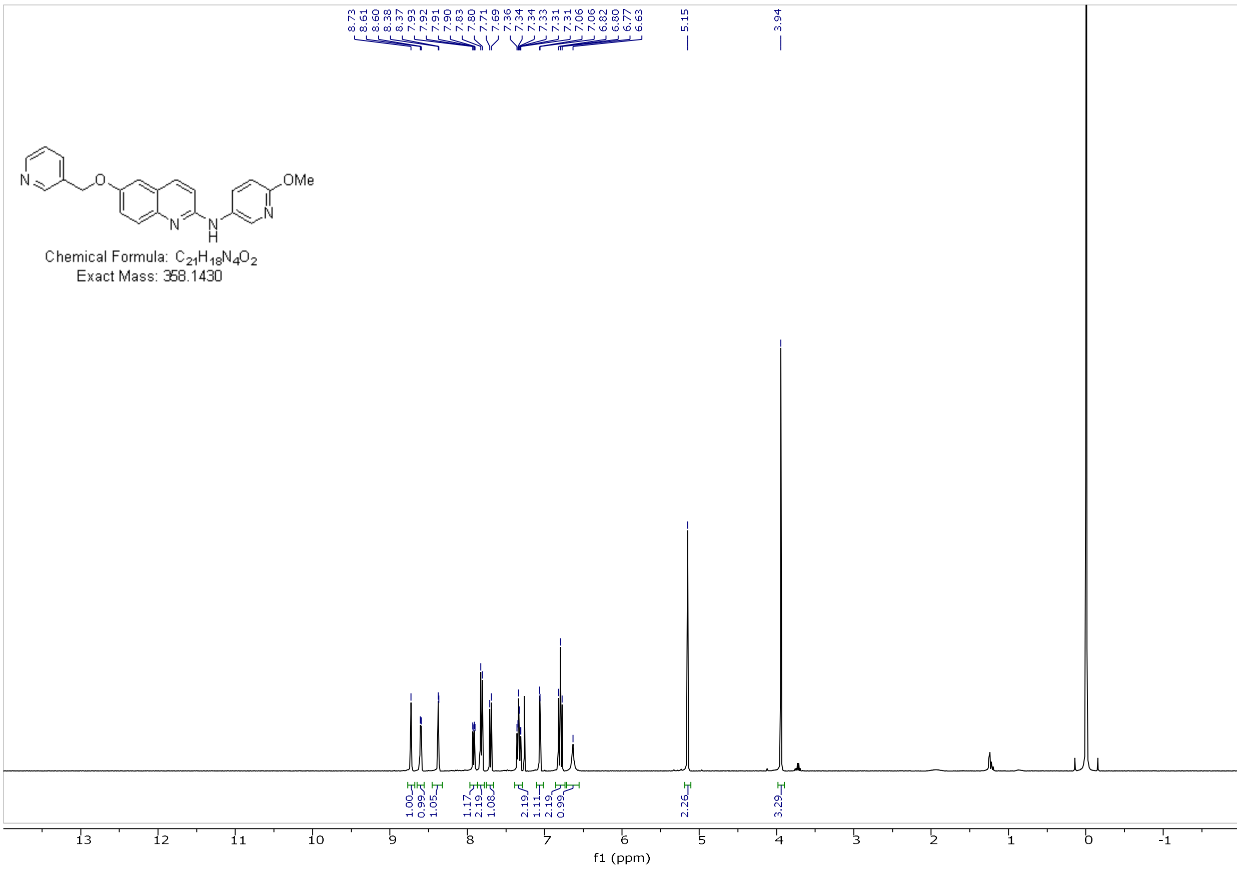

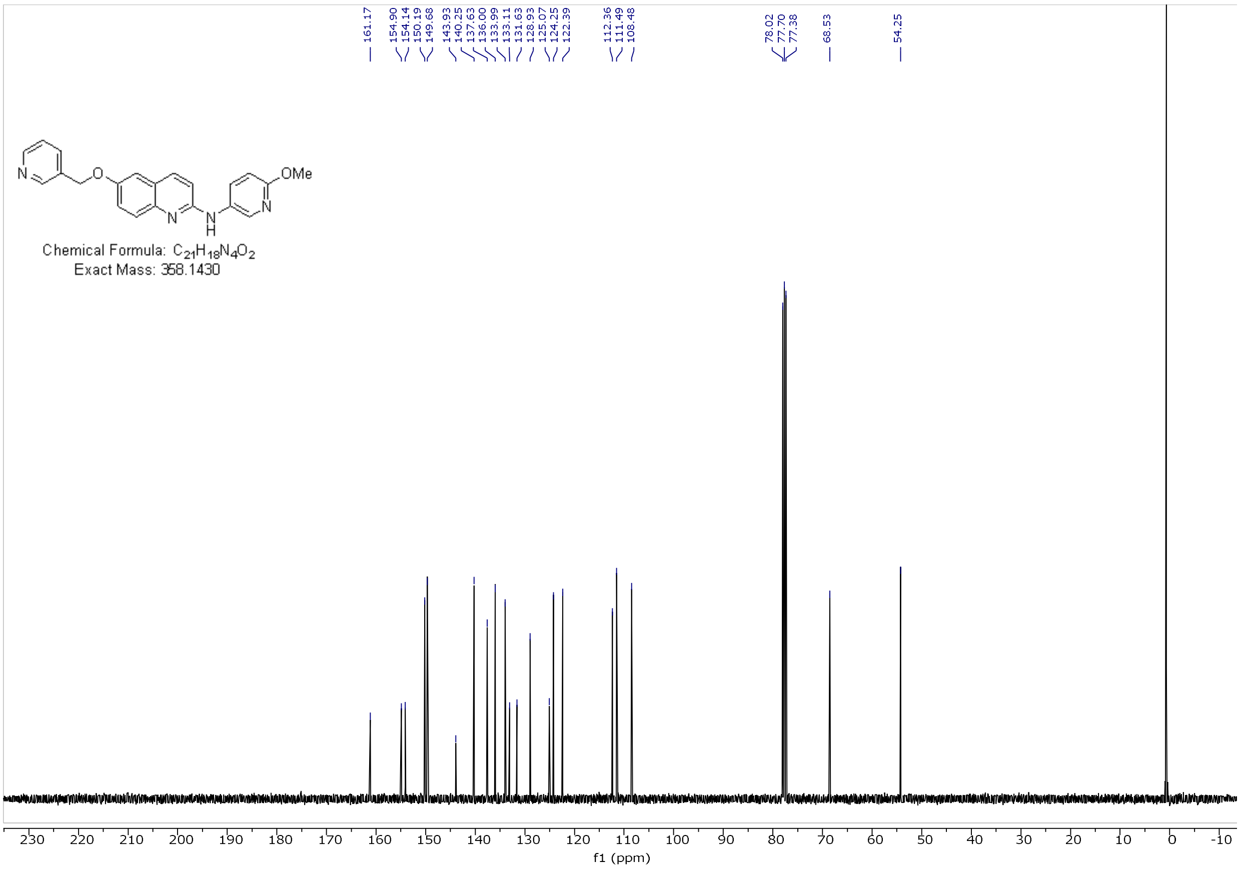

6-((4-Methoxybenzyl)oxy)-*N*-(6-methoxypyridin-3-yl)quinolin-2-amine (8f, TZ80-76).

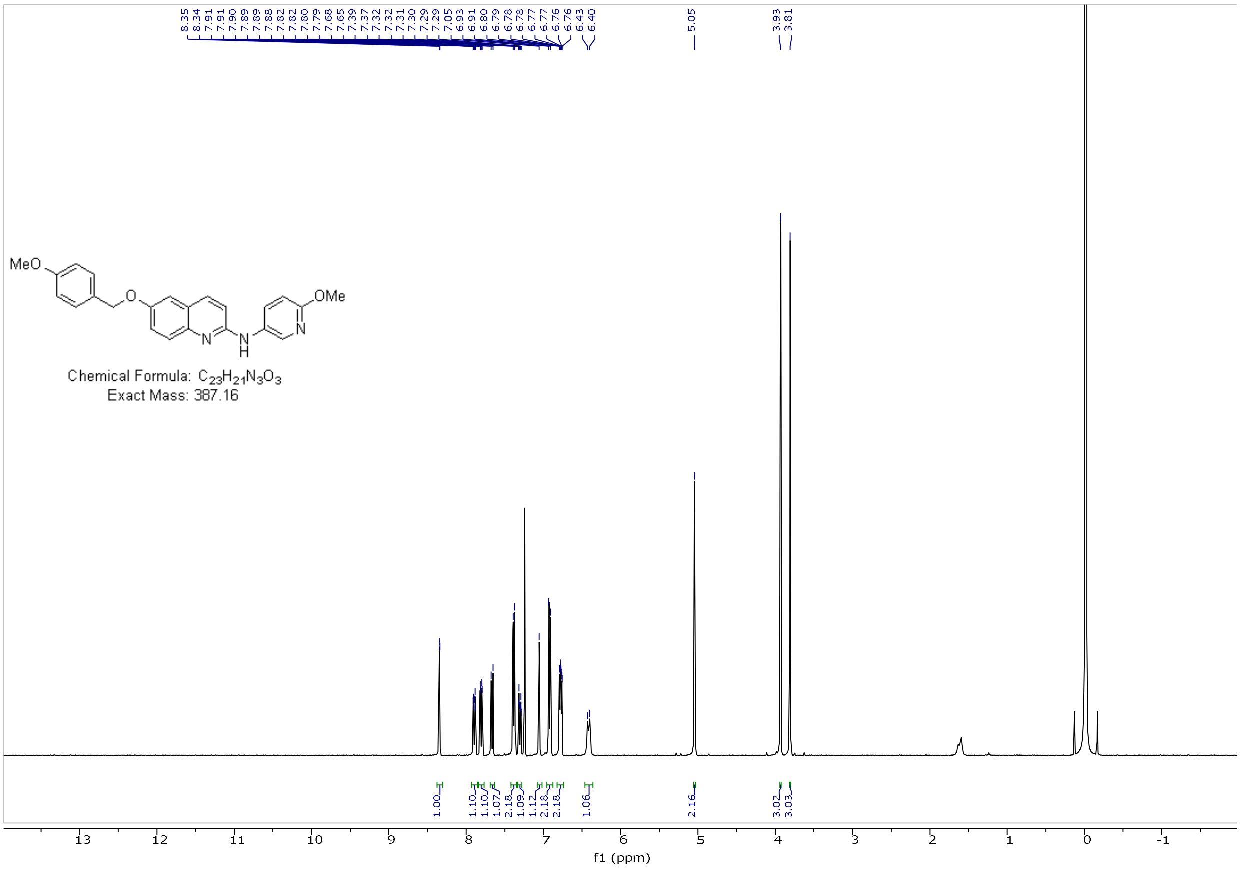

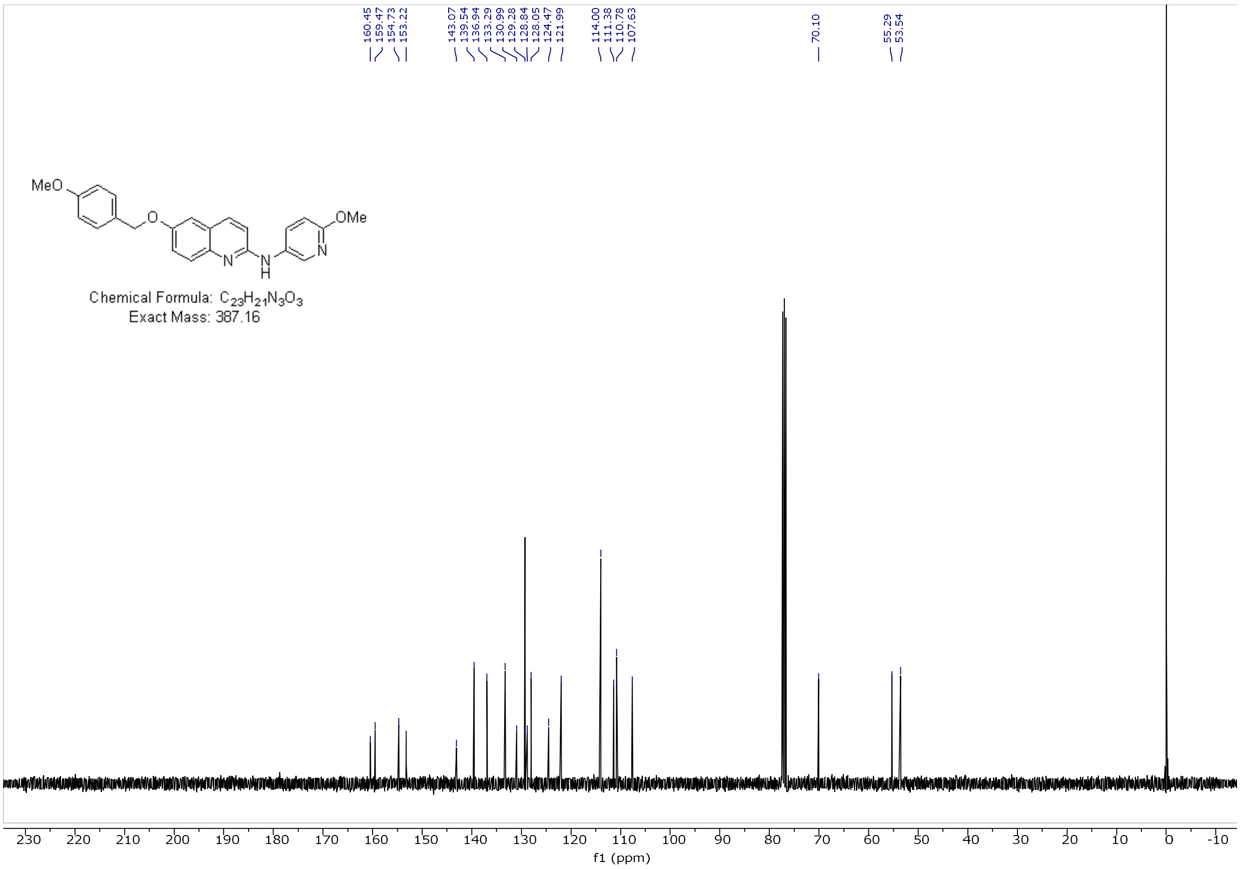

6-((3-Methoxybenzyl)oxy)-*N*-(6-methoxypyridin-3-yl)quinolin-2-amine (8g, TZ80-77).

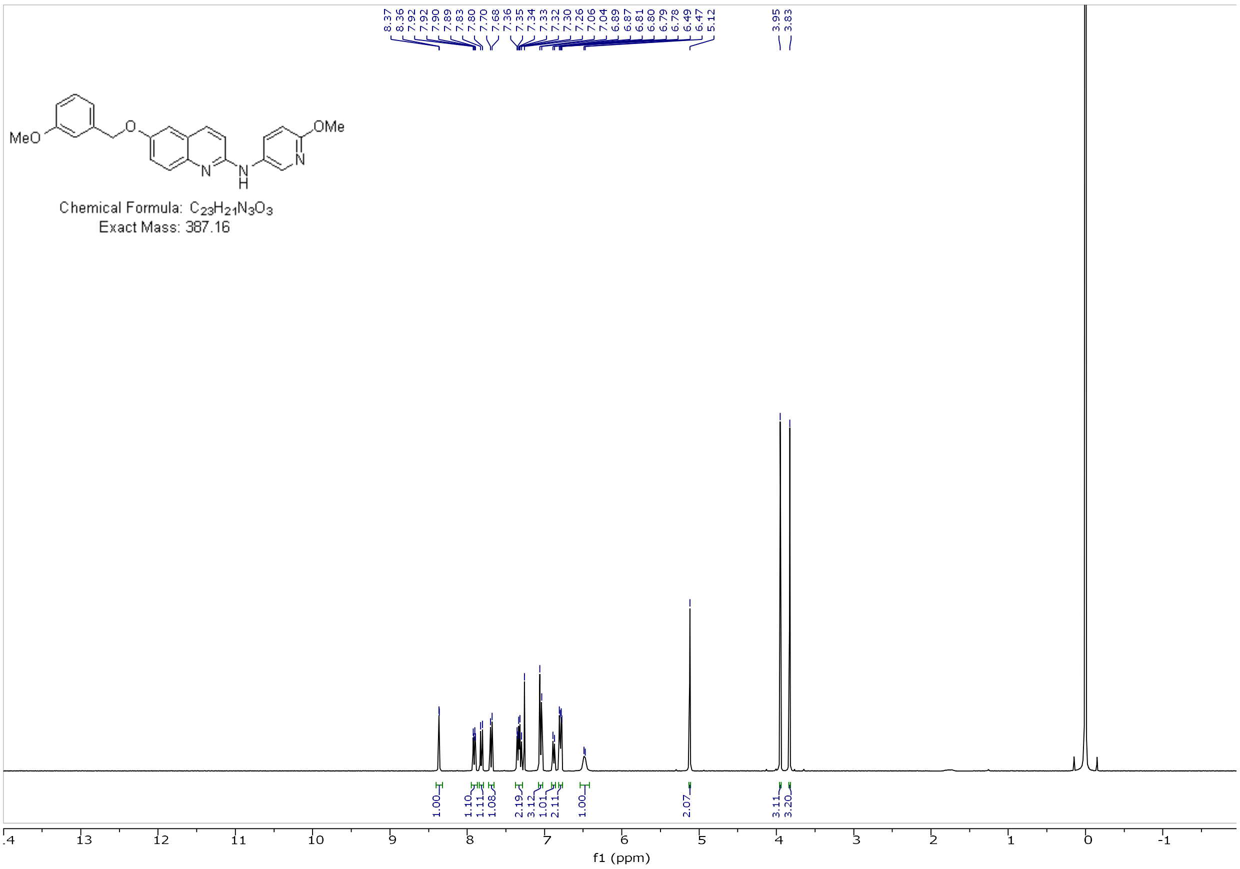

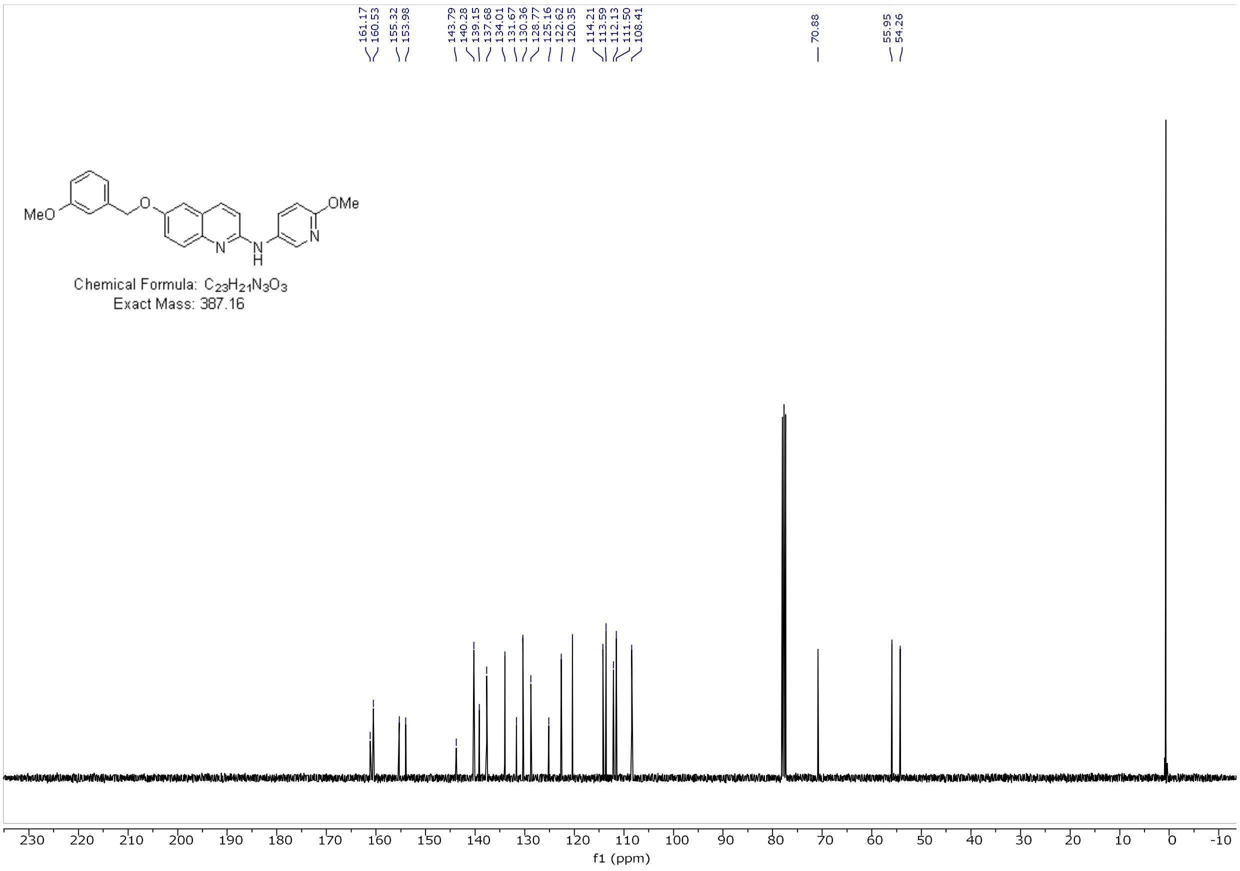

6-((4-Fluorobenzyl)oxy)-*N*-(6-methoxypyridin-3-yl)quinolin-2-amine (8h, TZ80-44).

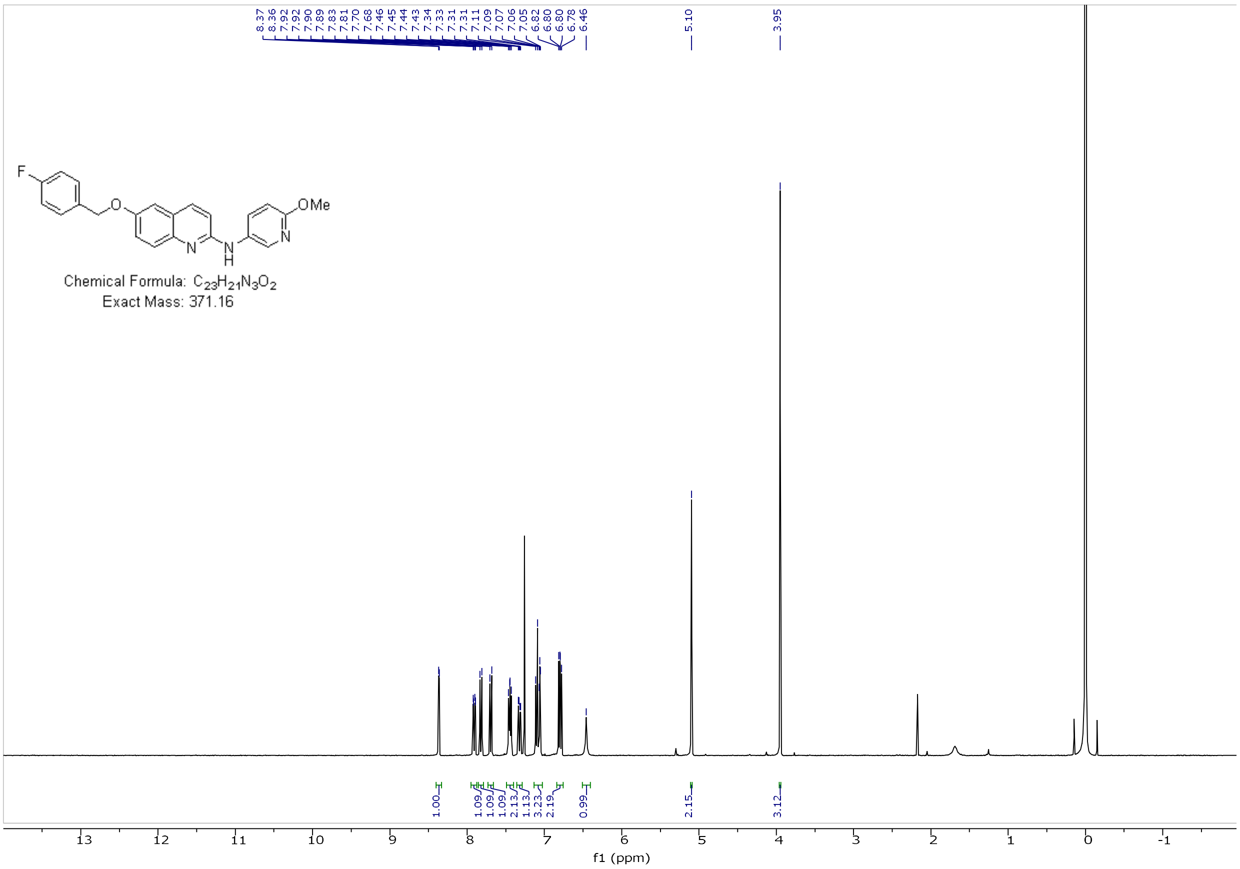

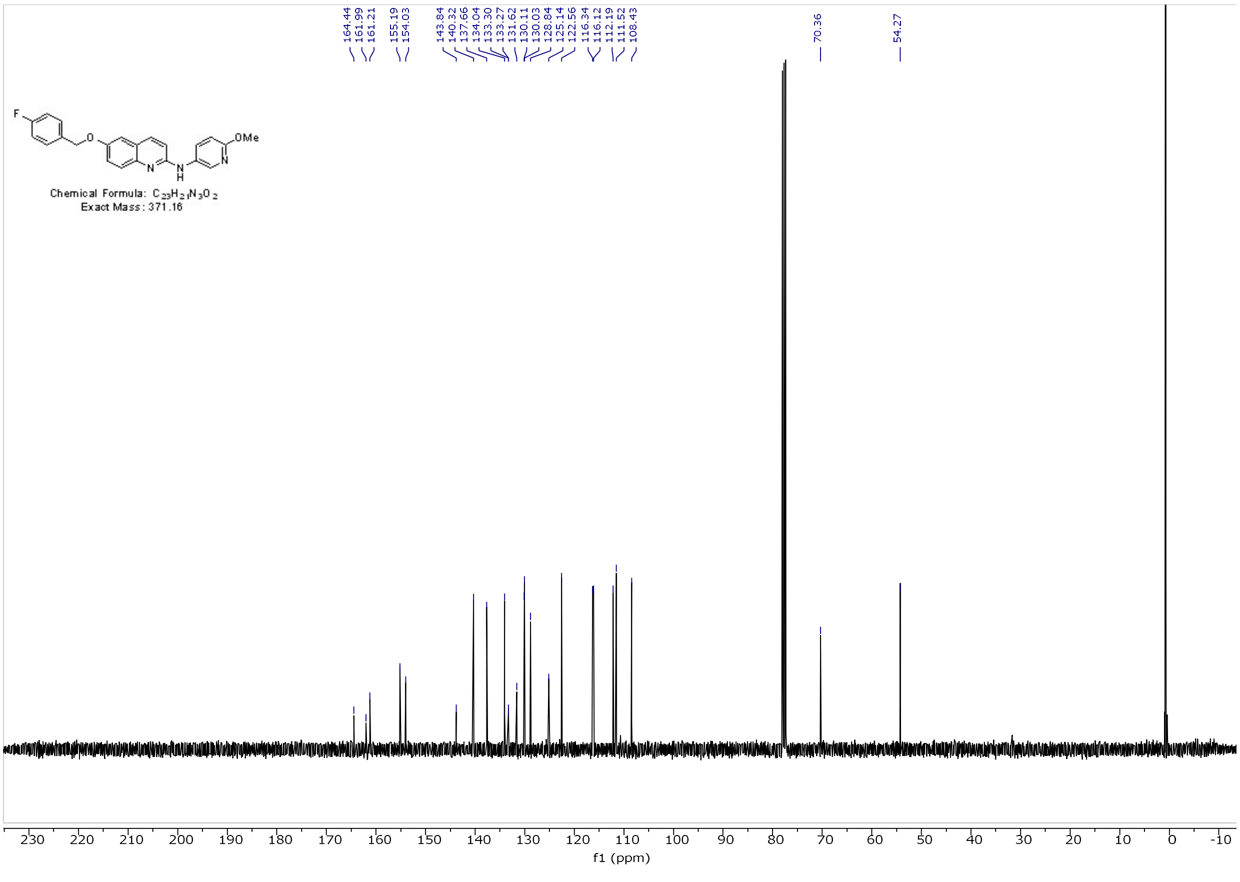

(*E*)-6-((3-Iodoallyl)oxy)-*N*-(6-methoxypyridin-3-yl)quinolin-2-amine (8i, TZ90-13).

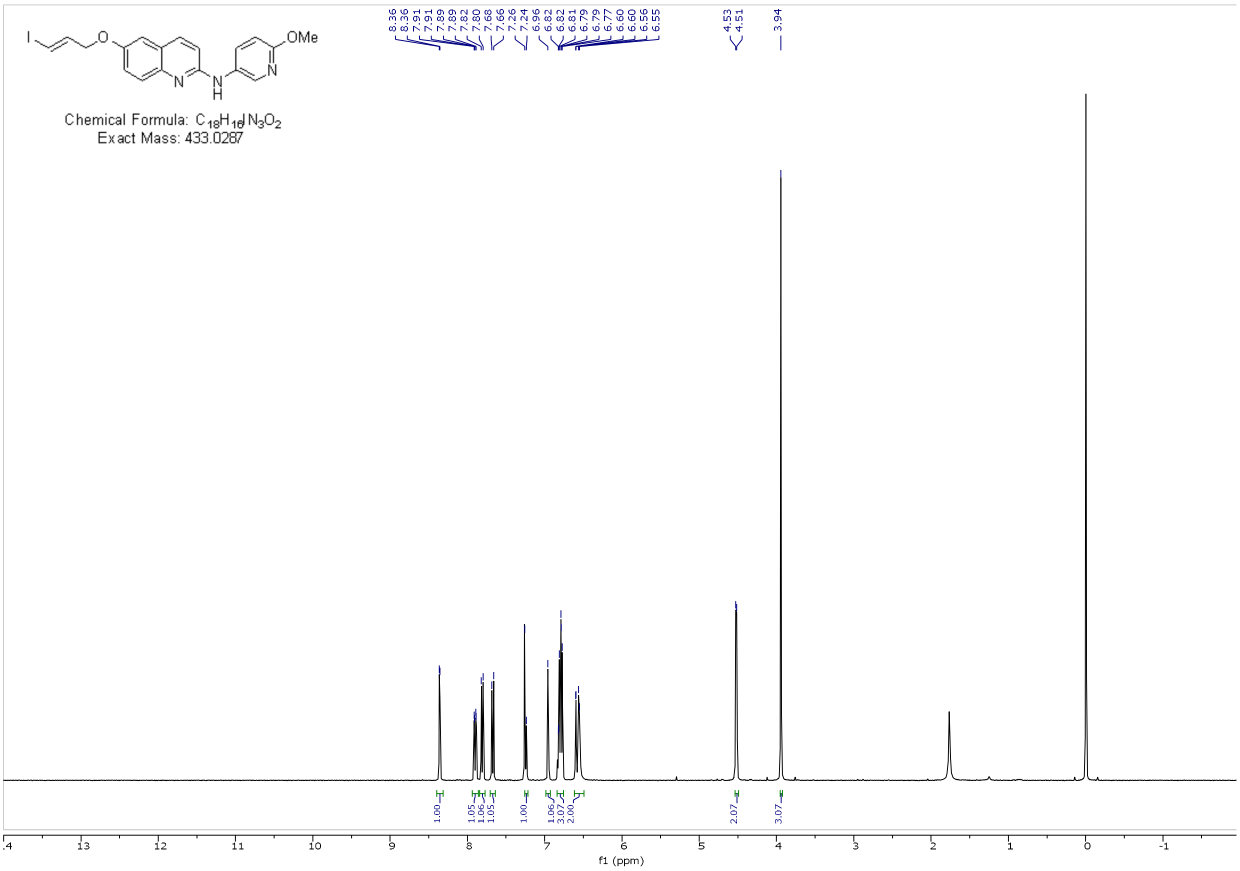

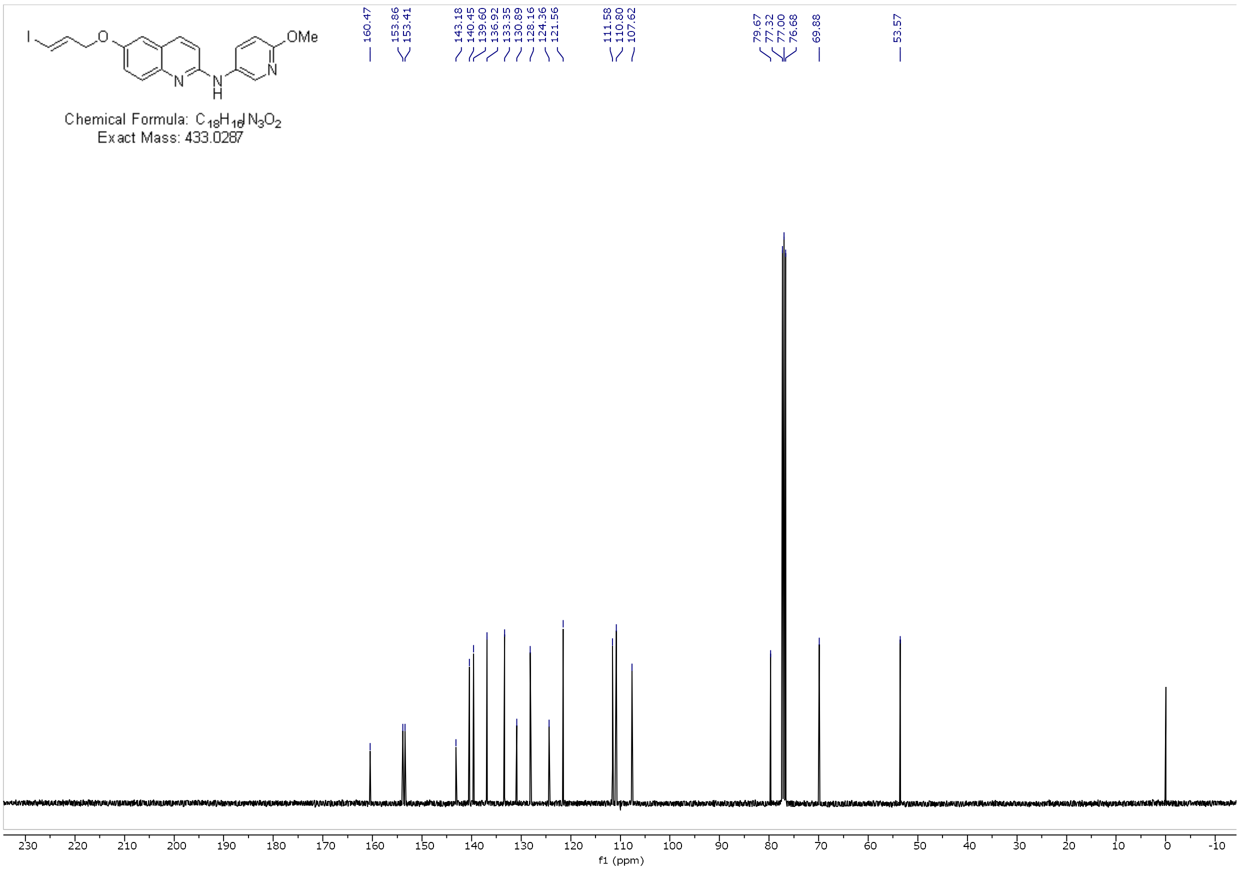

N^2,N^6-bis(6-methoxypyridin-3-yl)quinoline-2,6-diamine (8j, TZ80-102).

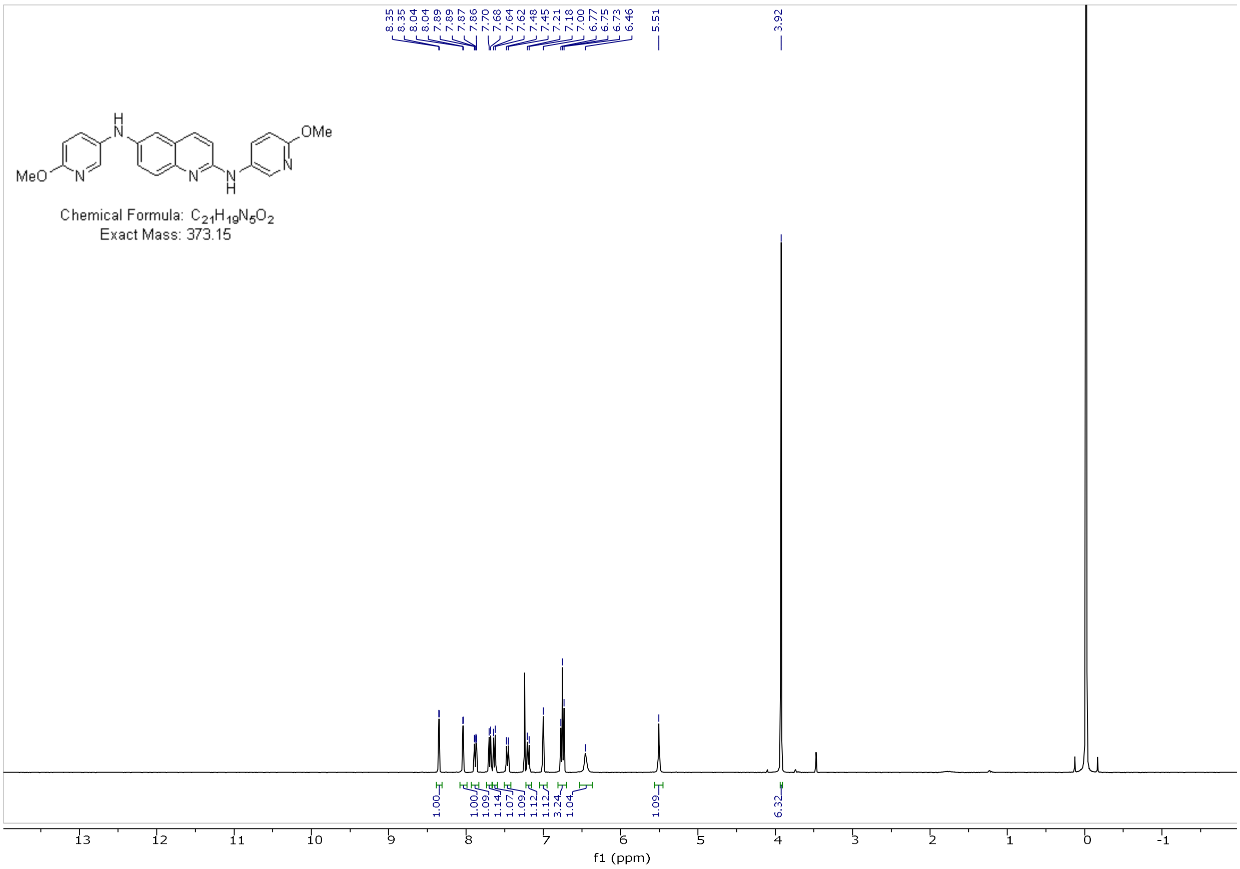

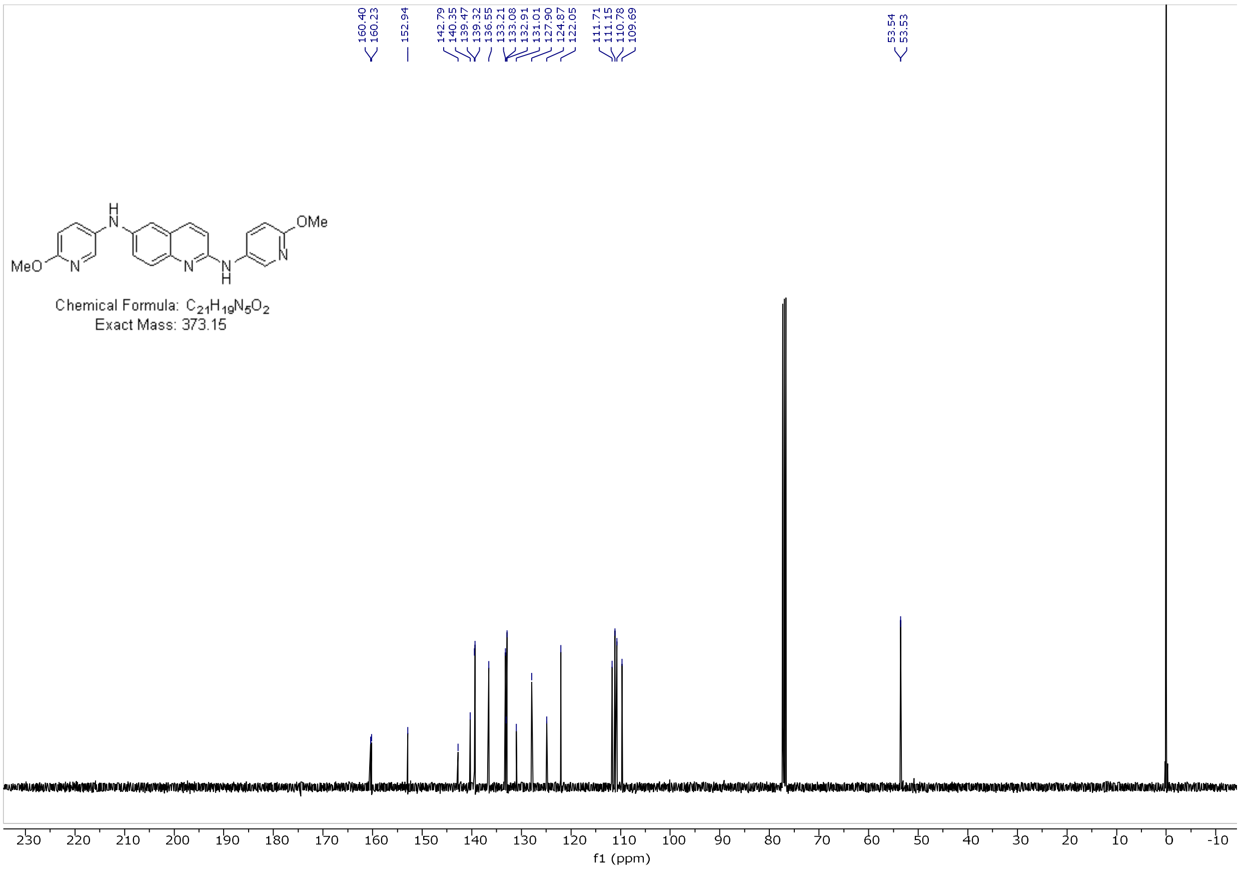

2-((6-Hydroxypyridin-3-yl)amino)quinolin-6-ol (9, TZ90-23).

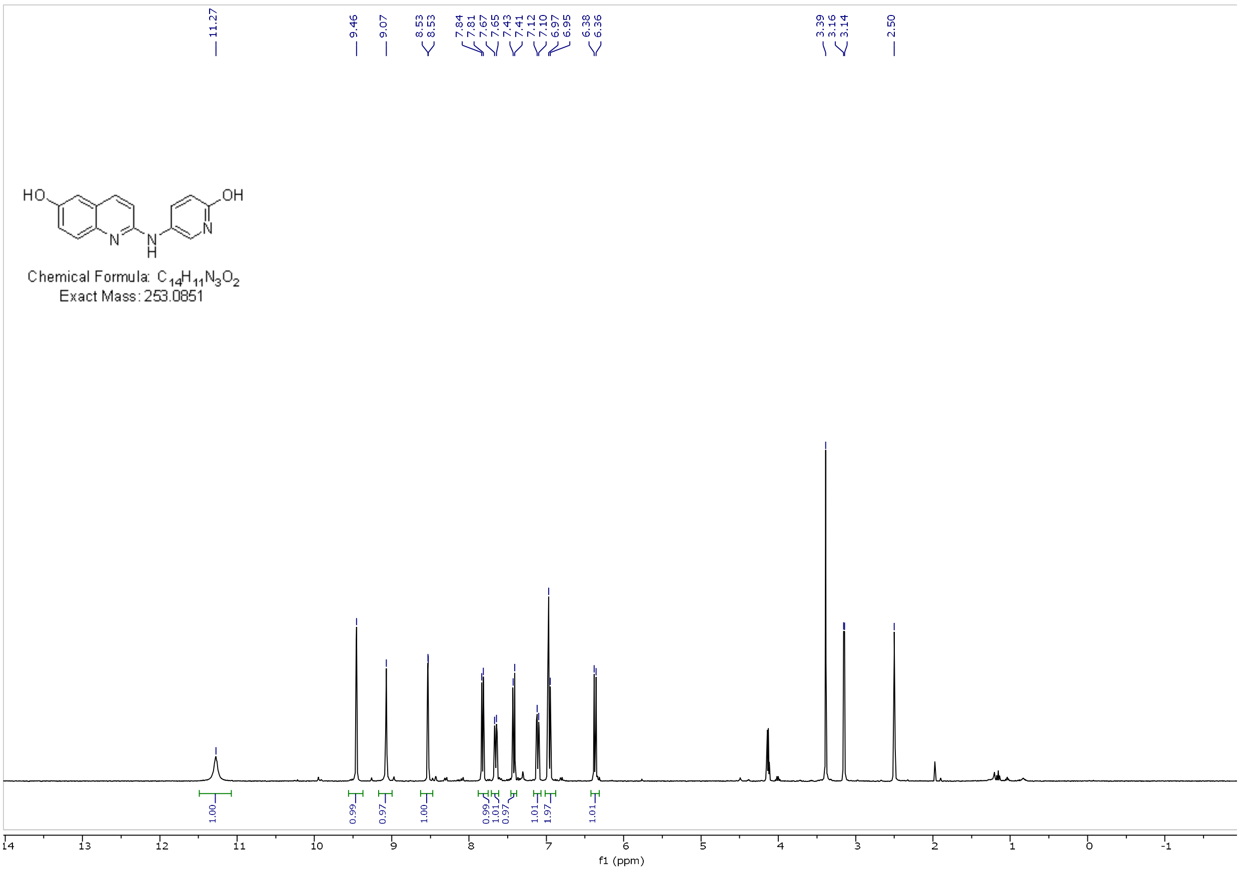
11.27
9.46
9.07
8.53
8.53
7.84
7.81
7.67
7.65
7.43
7.41
7.12
7.10
6.97
6.95
6.38
6.36
3.39
3.16
3.14
2.50
HO
OH
N
N
H
Chemical Formula: $C_{14}H_{11}N_3O_2$
Exact Mass: 253.0851
1.00
0.99
0.97
1.00
0.99
1.01
0.97
1.01
1.97
1.01
f1 (ppm)

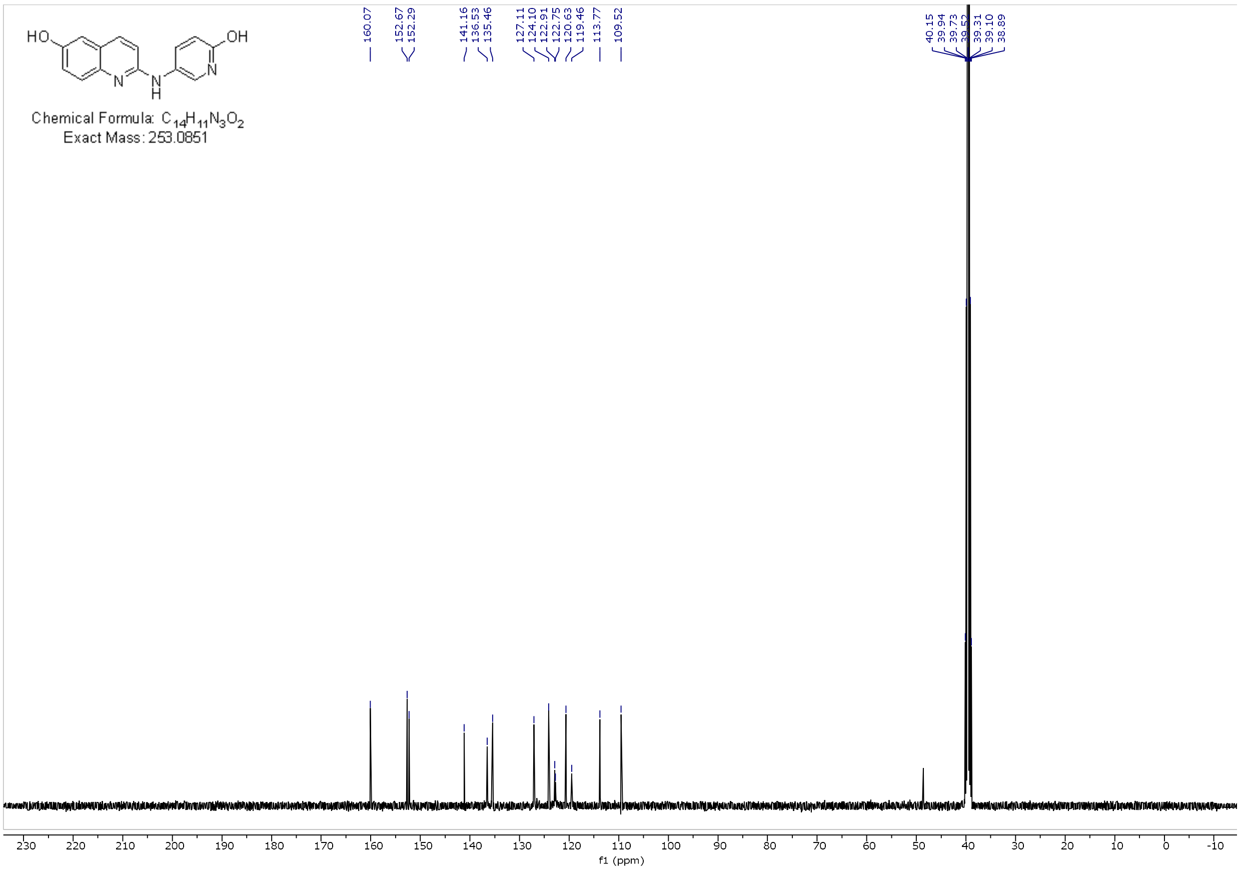
160.07
152.67
152.29
141.16
136.53
135.46
127.11
124.10
122.91
122.75
120.63
119.46
113.77
109.52
40.15
39.94
39.73
39.52
39.31
39.10
38.89
HO
OH
N
N
H
Chemical Formula: $C_{14}H_{11}N_3O_2$
Exact Mass: 253.0851
f1 (ppm)

(*E*)-5-((6-((3-iodoallyl)oxy)quinolin-2-yl)amino)pyridin-2-ol (10).

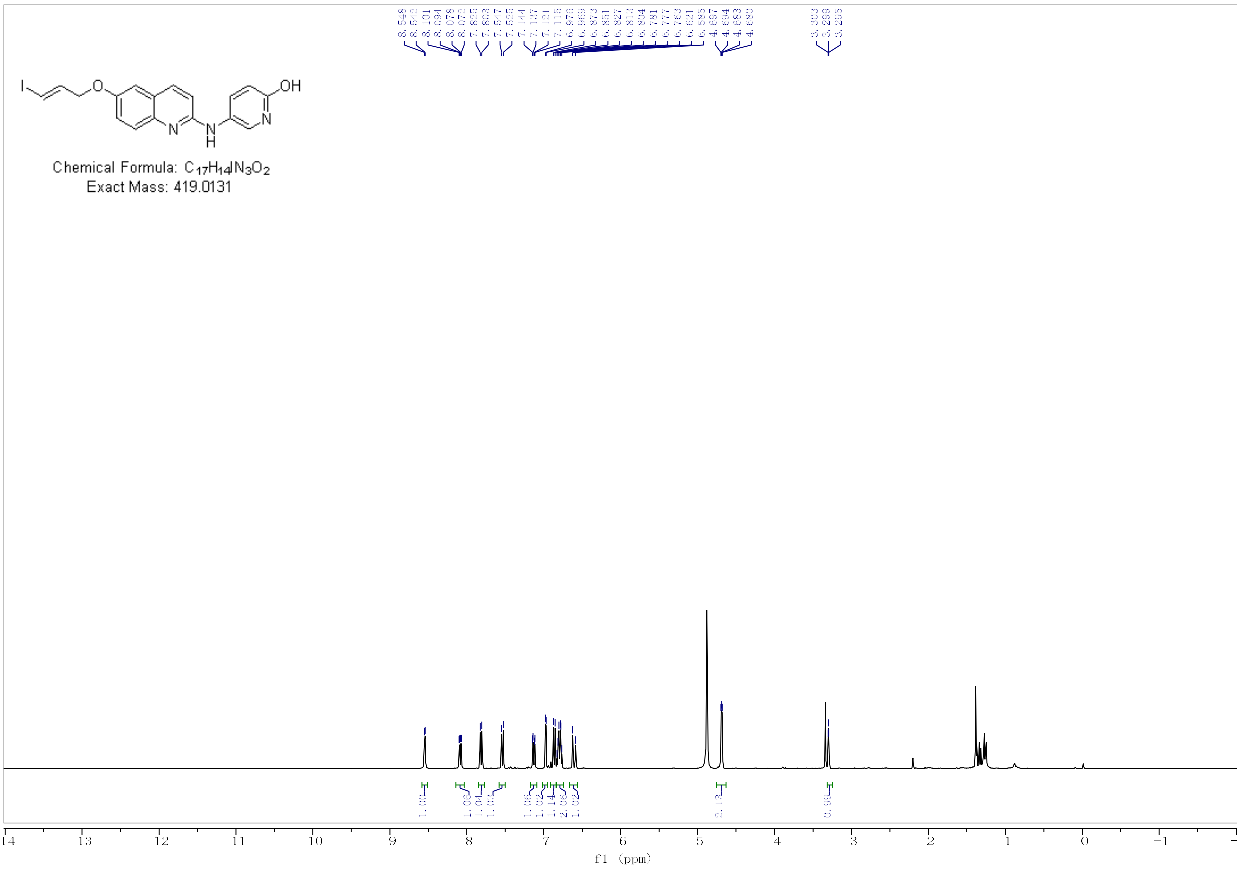

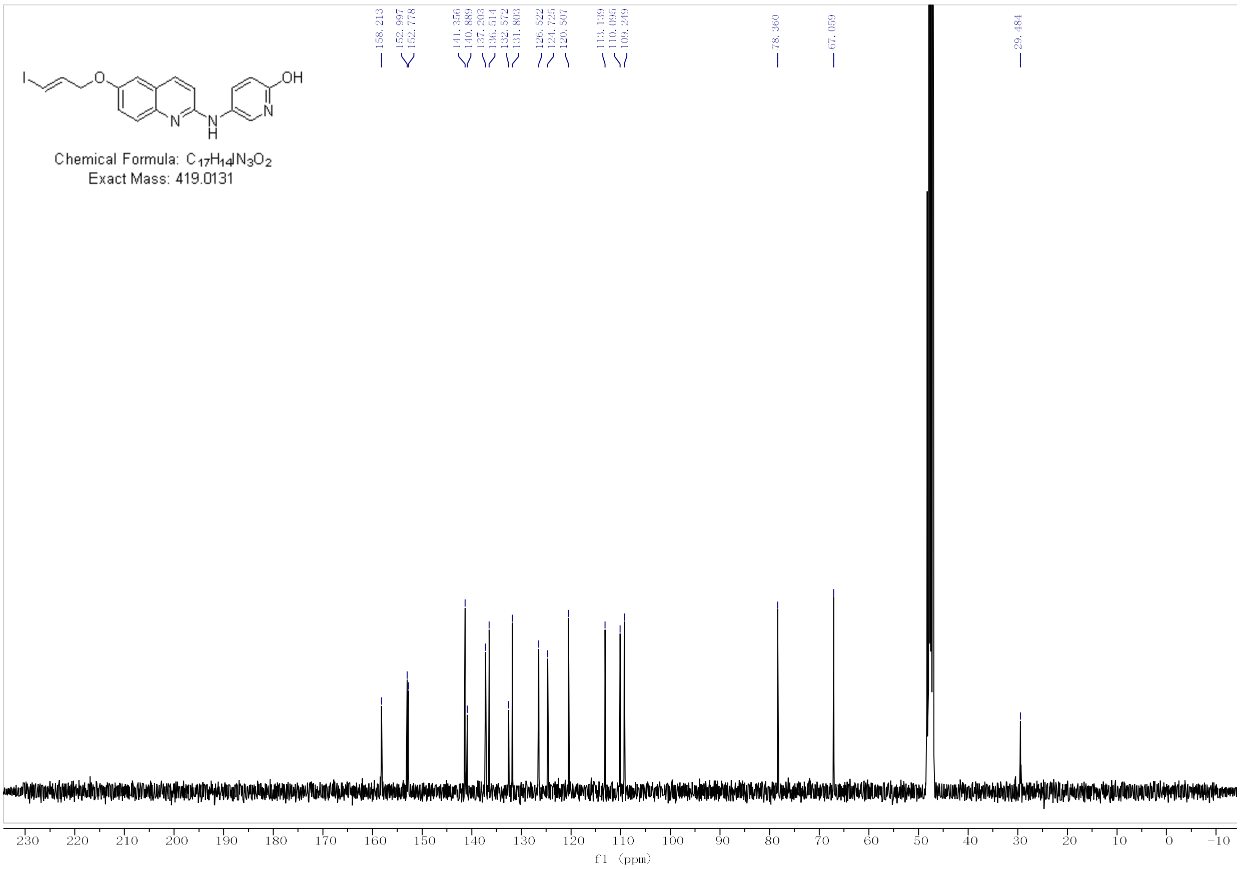

Supplement: Supplementary file 1 [file cells-14-01108-s001.zip › cells-3668023-supplementary.pdf]
